# Annelids of the eastern Australian abyss collected by the 2017 RV ‘Investigator’ voyage

**DOI:** 10.3897/zookeys.1020.57921

**Published:** 2021-02-24

**Authors:** Laetitia M. Gunton, Elena K. Kupriyanova, Tom Alvestad, Lynda Avery, James A. Blake, Olga Biriukova, Markus Böggemann, Polina Borisova, Nataliya Budaeva, Ingo Burghardt, Maria Capa, Magdalena N. Georgieva, Christopher J. Glasby, Pan-Wen Hsueh, Pat Hutchings, Naoto Jimi, Jon A. Kongsrud, Joachim Langeneck, Karin Meißner, Anna Murray, Mark Nikolic, Hannelore Paxton, Dino Ramos, Anja Schulze, Robert Sobczyk, Charlotte Watson, Helena Wiklund, Robin S. Wilson, Anna Zhadan, Jinghuai Zhang

**Affiliations:** 1 Australian Museum Research Institute, Sydney, Australia; 2 Macquarie University, Sydney, Australia; 3 Department of Natural History, University Museum of Bergen, University of Bergen, Bergen, Norway; 4 Museums Victoria, Melbourne, Australia; 5 Aquatic Research & Consulting, Duxbury, Massachusetts, USA; 6 Museum and Art Gallery of the Northern Territory, Darwin, Australia; 7 University of Vechta, Vechta, Germany; 8 P.P. Shirshov Institute of Oceanology, Russian Academy of Sciences, Moscow, Russia; 9 Department of Biology, University of the Balearic Islands, Palma, Spain; 10 Natural History Museum, London, UK; 11 Department of Life Sciences, National Chung Hsing University, Taichung City, China; 12 National Institute of Polar Research, Tachikawa, Tokyo, Japan; 13 Department of Biology, University of Pisa, Pisa, Italy; 14 Forschungsinstitut Senckenberg, DZMB, Hamburg, Germany; 15 Texas A&M University at Galveston, Galveston, TX, USA; 16 Department of Zoology of Invertebrates and Hydrobiology, University of Lodz, Lodz, Poland; 17 Gothenburg Global Biodiversity Centre and University of Gothenburg, Gothenburg, Sweden; 18 Biological Faculty, Lomonosov Moscow State University, Moscow, Russia; 19 South China Sea Environmental Monitoring Centre, State Oceanic Administration, Guangzhou, China

**Keywords:** Biodiversity, Biogeography, deep sea, Echiura, lower-bathyal, Marine Parks, Polychaeta, Sipuncula, Tasman Sea

## Abstract

In Australia, the deep-water (bathyal and abyssal) benthic invertebrate fauna is poorly known in comparison with that of shallow (subtidal and shelf) habitats. Benthic fauna from the deep eastern Australian margin was sampled systematically for the first time during 2017 RV ‘Investigator’ voyage ‘Sampling the Abyss’. Box core, Brenke sledge, and beam trawl samples were collected at one-degree intervals from Tasmania, 42°S, to southern Queensland, 24°S, from 900 to 4800 m depth. Annelids collected were identified by taxonomic experts on individual families around the world. A complete list of all identified species is presented, accompanied with brief morphological diagnoses, taxonomic remarks, and colour images. A total of more than 6000 annelid specimens consisting of 50 families (47 Polychaeta, one Echiura, two Sipuncula) and 214 species were recovered. Twenty-seven species were given valid names, 45 were assigned the qualifier cf., 87 the qualifier sp., and 55 species were considered new to science. Geographical ranges of 16 morphospecies extended along the eastern Australian margin to the Great Australian Bight, South Australia; however, these ranges need to be confirmed with genetic data. This work providing critical baseline biodiversity data on an important group of benthic invertebrates from a virtually unknown region of the world’s ocean will act as a springboard for future taxonomic and biogeographic studies in the area.

## Introduction

The deep sea (> 200 m depth) is the least explored environment on our planet, where most species have not been sampled and remain undiscovered. The vast sediments of the deep sea cover approximately 65% of the Earth’s surface, and it is a unique environment characterised by darkness, low temperatures and low currents, high hydrostatic pressure, and well oxygenated oligotrophic waters (Gage and Tyler 1991). Unfortunately, the deep-sea environment is increasingly affected by anthropogenic impact such as overfishing (Bailey et al. 2009), oil and gas exploration and extraction, waste disposal and pollution (reviewed in Glover and Smith 2003; Ramirez-Llodra et al. 2011; Mengerink et al. 2014). Thus, accurate baseline data on species is essential for monitoring, protecting and managing biological communities.

In Australia, the abyssal plain (3000 to 6000 m depth) and deep ocean floor covers ~ 2.8 million km^2^, or 30% of Australia’s marine territory (Heap and Harris 2008). The abyssal plain is a major part of Australia’s ~ 10 million km^2^ Exclusive Economic Zone (**EEZ**), which is the third largest EEZ in the world. The deeper areas of Australia’s EEZ including Marine Parks (**MP**) have been extremely poorly sampled for fauna compared with the intertidal and shallow sublittoral waters (Ponder et al. 2002). While the subtidal and shelf fauna of eastern Australian coasts is the best studied in the continent due to research capacity and high population density in the area, the deep-water fauna beyond the narrow shelf was virtually unknown. Until recently more was known about deep-water benthic fauna off the sparsely populated western Australian coast (McEnnulty et al. 2011; Poore et al. 2015) than off eastern Australia.

Earlier sampling of the eastern Australian abyss was performed as part of research expeditions to the area organised by non-Australian institutions. These include expeditions dating back to the H.M.S. ‘Challenger’ expedition (1874, the UK), the ‘Galathea’ expedition (1951–52, Denmark), the research vessel (**RV**) ‘Dmitry Mendeleev’ (1975–76, USSR), and RV ‘Tangaroa’ voyages (1982, New Zealand) (reviewed in O’Hara 2019). This is because Australia lacked the required capacity to consistently collect biological material from the seafloor at great depths. Surveys of the lower shelf to abyssal depths (200–3150 m) off the south-eastern margin of Australia were conducted from the Australian oceanographic research vessel (**ORV**) ‘Franklin’ in 1986 and 1988 (Poore et al. 1994) along four transects, but the report focused on isopods only.

A new era for deep-sea biological exploration in Australia began in 2014 with the launch of the Marine National Facility’s RV ‘Investigator’, the first Australian research vessel equipped to routinely perform biological sampling to depths of 5000 m. The systematic biological study of abyssal depths in Australia on board RV ‘Investigator’ started with the Great Australian Bight (**GAB**) Research Program. This programme conducted six surveys off the southern coastline of Australia during 2013, 2015, and 2017, sampling epifauna from soft substrates, rocky outcrops in canyons and seamounts from depths of 200–5000 m (MacIntosh et al. 2018).

The significant gap in knowledge about the eastern abyss was addressed by the 2017 ‘Sampling the Abyss’ research project supported by the Marine National Facility, the Commonwealth Scientific and Industrial Research Organisation (**CSIRO**) and Museums Victoria. This was the first dedicated deep-sea cruise to perform a systematic biological survey along the eastern Australian coast, from Tasmania to southern Queensland covering the lower bathyal (~ 2500 m) and abyssal environments (~ 4000 m). This was also the first expedition to collect biological samples from the deeper parts of the eastern network of Australian Marine Parks (O’Hara 2017). The present study focuses on the biodiversity of annelids collected during this voyage.

Annelids occur in all marine environments and they are typically a dominant macrofauna (> 300 µm) taxon in terms of abundance and species diversity in deep-sea soft sediments (Herring 2010; Rex and Etter 2010). They display a diverse range of life history strategies and feeding modes (Jumars et al. 2015) and play important roles in processing and burying organic matter, recycling nutrients and bioturbation of seafloor sediments (Hutchings 1998). More than 2000 annelid species are known from Australia (http://www.ala.org.au), yet only 15 species from six families had been described from below 1000 m and three species from below 2500 m (Table 1), the shallower depth limits of the present study. Of 158,400 records of annelids in Australia 770 are from below 1000 m and only 99 from below 2500 m depth (http://www.ala.org.au), suggesting deep-water biodiversity is severely underestimated.

This study reports an illustrated and annotated preliminary species-level checklist of the annelid fauna collected during the 2017 ‘Sampling the Abyss’ survey along with species diversity and distribution data. Morphospecies are compared with those collected from the GAB sampling programme where possible.

**Table 1.** Annelid species described below 1000 m in Australian waters (roughly corresponding to Exclusive Economic Zone, 12 nautical miles from the coast). Bold font indicates species from eastern Australian margin.

Family	Species	Depth (m)	Type Locality
** Polynoidae **	***Lepidasthenia australiensis* (Augener, 1927)**	**1000**	**Off eastern Victoria**
** Sabellidae **	***Potaspina australiensis* Capa, 2007**	**1000**	**South of Point Hicks, Victoria**
** Polynoidae **	***Brychionoe karenae* Hanley & Burke, 1991**	**1100**	**Cascade Plateau off Tasmania**
** Onuphidae **	***Paradiopatra imajimai* Paxton & Budaeva, 2013**	**1277**	**Off eastern Victoria**
Polynoidae	*Lagisca torbeni* Kirkegaard, 1995	1320–1340	Great Australian Bight, south of Adelaide
Polynoidae	*Harmothoe australis* Kirkegaard, 1995	1340	Great Australian Bight, south of Adelaide
Spionidae	*Laonice pectinata* Greaves, Meißner & Wilson, 2011	1440	Indian Ocean, west of Perth
** Onuphidae **	***Paradiopatra spinosa* Paxton & Budaeva, 2013**	**1600**	**Bass Canyon**
** Polynoidae **	***Eunoe ivantsovi* Averincev, 1978**	**1640**	**Lord Howe Island Rise**
Polynoidae	*Eunoe papillaris* Averincev, 1978	1800	Off southwestern Tasmania
** Nephtyidae **	***Aglaophamus profundus* Rainer & Hutchings, 1977**	**2195**	**Off northeastern Tasmania**
** Polynoidae **	***Parapolyeunoa flynni* (Benham, 1921)**	**2379**	**Off Maria Island, Tasmania**
Fauveliopsidae	*Fauveliopsis challengeriae* McIntosh, 1922	3566	South Indian Ocean, midway between Australia and Antarctica
Polynoidae	*Eunoe abyssorum* McIntosh, 1885	4755	South of Australia
Polynoidae	*Polynoe ascidioides* McIntosh, 1885 (now considered a nomen dubium)	4755	South of Australia

## Materials and methods

### Sampling area

The eastern Australian continental shelf is relatively narrow compared with the rest of the continent. The shelf break occurs ~ 15 km from the coast and the foot-of-slope and beginning of the abyssal plain can be as close as 60 km from the coast (Heap and Harris 2008). The eastern margin contains a range of geomorphological features including plateaus, basins, terraces, deeps/holes, and submarine canyons seamounts/guyots (Heap and Harris 2008).

The East Australian Current (**EAC**) is an important shallow water current carrying ~ 22–27 Sverdrups from north to south along the east coast of Australia. This counter-clockwise southern Pacific gyre circulates shallow water from the Coral Sea along the continental margin until 32–35°S before heading eastward to New Zealand. Part of the EAC is deflected offshore ~ 30°S along the Tasman Front, this divides the warm waters of the Coral Sea and the cooler waters of the Tasman Sea (Rintoul et al. 2017). Deeper currents (> 2000 m) have not been directly measured but have been inferred from sediment deposition and erosion patterns; these currents are thought to be weak with a western boundary undercurrent flowing northwards along the eastern Australian margin and an eastern boundary counter-flow along the eastern margin of the Tasman Sea (Jenkins 1984).

### Field collection and processing

**Figure 1.**
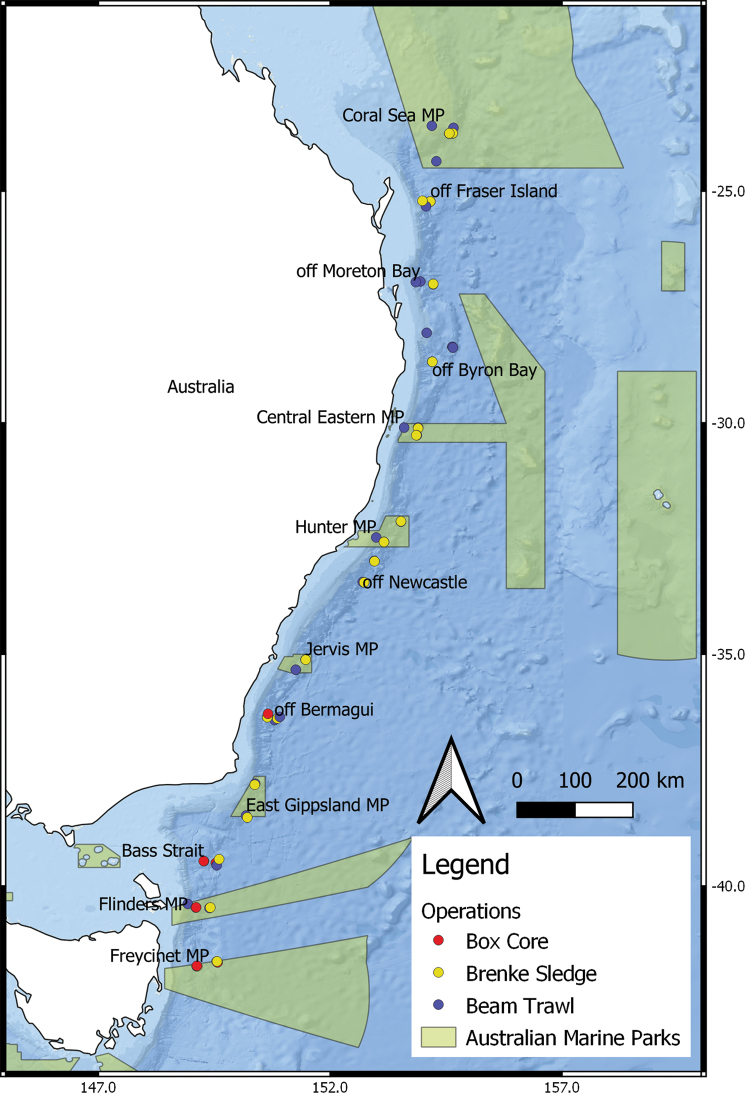
Map of sampling sites from expedition IN2017_V03 along eastern Australia. Blue dots represent beam trawl, yellow dots Brenke sledge, red dots box core sampling sites, green polygons represent areas of Australian Marine Parks.

Biological samples were collected from 13 sites at one-degree intervals of latitude from 42°S to 24°S along the east coast of Australia from Tasmania to Southern Queensland (Fig. 1, Table 2) on the RV ‘Investigator’ (voyage code IN2017_V03), from 15 May to 16 June 2017. Seven Marine Parks were included (Freycinet MP, Flinders MP, East Gippsland MP, Jervis MP, Hunter MP, Central Eastern MP, and Coral Sea MP). Benthic sampling was conducted at lower bathyal (~ 2500 m) and abyssal (~ 4000 m) depths, with some (seven operations) comparative samples taken at shallower mid-bathyal depths (~ 1000 m). The three types of sampling gear used were beam trawl (35 operations, ops.), Brenke sledge (28 ops.), and box core (8 ops.).

**Table 2.** Sample Sites. Beam trawl, Brenke sledge, and box core deployments on RV ‘Investigator’ cruise IN2017_V03 from the Australian eastern lower bathyal and abyssal environment. Abbreviations: Op., operation, BT, CSIRO 4-metre beam trawl; BS, Brenke sledge; BC, box core; *, unfired ; **, aborted.

Op.	Location	Gear	Date	Start latitude and longitude	End latitude and longitude	Trawling distance (km)	Start depth (m)	End depth (m)
004	Freycinet MP	BT	18/05/17	-41.731, 149.120	-41.791, 149.156	7.3	2820	2751
005	Freycinet MP	BS	18/05/17	-41.730, 149.135	-41.753, 149.147	2.8	2789	2779
006	Freycinet MP	BT	18/05/17	-41.626, 149.552	-41.689, 149.584	7.5	4022	4052
007	Freycinet MP	BC	18/05/17	-41.647, 149.570			4030	
008	Freycinet MP	BC	19/05/17	-41.647, 149.569			4012	
009	Freycinet MP	BS	19/05/17	-41.626, 149.560	-41.662, 149.574	4.2	4021	4035
011	Freycinet MP	BC	19/05/17	-41.721, 149.125			2793	
013	Flinders MP	BT	20/05/17	-40.386, 148.928	-40.383, 148.951	2.0	932	1151
014	Flinders MP	BT	20/05/17	-40.464, 149.102	-40.461, 149.147	3.8	2298	2486
015	Flinders MP	BT	20/05/17	-40.473, 149.397	-40.464, 149.426	2.6	4114	4139
016	Flinders MP	BS	21/05/17	-40.463, 149.415	-40.461, 149.364	4.3	4129	4131
017	Flinders MP	BC	21/05/17	-40.460, 149.109		s	2331	
022	Bass Strait	BT	22/05/17	-39.462, 149.276	-39.465, 149.242	2.9	2760	2692
023	Bass Strait	BS	22/05/17	-39.462, 149.277	-39.465, 149.246	2.7	2774	2694
027	Bass Strait	BC	22/05/17	-39.462, 149.271			2741	
028	Bass Strait	BC	22/05/17	-39.500, 149.535			4147	
030	Bass Strait	BT	23/05/17	-39.552, 149.553	-39.496, 149.598	7.3	4197	4133
031	Bass Strait	BS	23/05/17	-39.422, 149.604	-39.391, 149.597	3.5	4150	4170
032	East Gippsland MP	BT	24/05/17	-38.479, 150.185	-38.453, 150.186	2.9	3850	3853
033	East Gippsland MP	BS	24/05/17	-38.521, 150.213	-38.498, 150.207	2.6	4107	4064
035	East Gippsland MP	BT	25/05/17	-37.792, 150.382	-37.818, 150.353	3.9	2338	2581
040	East Gippsland MP	BS	25/05/17	-37.815, 150.373	-37.818, 150.356	1.5	2746	2600
041	off Bermagui	BT	26/05/17	-36.418, 150.800			3980	
042	off Bermagui	BS	26/05/17	-36.385, 150.863	-36.434, 150.863	5.4	4744	4716
043	off Bermagui	BT	27/05/17	-36.351, 150.914	-36.384, 150.913	3.7	4800	4800
044	off Bermagui	BT	27/05/17	-36.355, 150.644	-36.315, 150.651	4.5	2821	2687
045	off Bermagui	BS	27/05/17	-36.360, 150.644	-36.323, 150.650	4.1	2835	2739
046	off Bermagui	BC	27/05/17	-36.284, 150.658			2643	
053	Jervis MP	BT	28/05/17	-35.114, 151.469	-35.084, 151.441	4.2	3952	4011
054	Jervis MP	BS	28/05/17	-35.117, 151.473	-35.099, 151.455	2.6	4026	3881
055	Jervis MP	BS	28/05/17	-35.335, 151.259	-35.334, 151.219	3.6	2667	2665
056	Jervis MP	BT	29/05/17	-35.333, 151.258	-35.332, 151.214	4.0	2650	2636
57*	Jervis MP	BC						
065	off Newcastle	BT	30/05/17	-33.441, 152.702	-33.435, 152.665	3.5	4280	4173
066	off Newcastle	BS	30/05/17	-33.448, 152.733	-33.437, 152.674	5.6	4378	4195
067	off Newcastle	BT	31/05/17	-32.985, 152.952	-33.015, 152.913	4.9	2704	2902
068	off Newcastle	BS	31/05/17	-32.993, 152.957	-33.023, 152.943	3.6	2745	2963
069	Hunter MP	BT	03/06/17	-32.479, 152.994	-32.507, 152.991	3.1	1006	1036
070	Hunter MP	BT	03/06/17	-32.575, 153.162	-32.632, 153.142	6.6	2595	2474
076	Hunter MP	BS	03/06/17	-32.577, 153.161	-32.613, 153.149	4.2	2534	2480
078	Hunter MP	BT	04/06/17	-32.138, 153.527	-32.182, 153.524	4.9	3980	4029
079	Hunter MP	BS	04/06/17	-32.131, 153.527	-32.163, 153.524	3.6		4031
080	Central Eastern MP	BT	05/06/17	-30.099, 153.596	-30.128, 153.571	4.0	1257	1194
086	Central Eastern MP	BT	05/06/17	-30.098, 153.899	-30.119, 153.875	3.3	2429	2518
087	Central Eastern MP	BS	06/06/17	-30.113, 153.898	-30.116, 153.867	3.0	2634	2324
088	Central Eastern MP	BT	06/06/17	-30.264, 153.870	-30.287, 153.830	4.6	4481	4401
089	Central Eastern MP	BS	06/06/17	-30.263, 153.859	-30.289, 153.844	3.2	4436	4414
090	off Byron Bay	BT	07/06/17	-28.677, 154.203	-28.709, 154.190	3.8	2587	2562
096	off Byron Bay	BS	07/06/17	-28.678, 154.204	-28.716, 154.189	4.5	2591	2566
097	off Byron Bay	BT	08/06/17	-28.355, 154.636	-28.414, 154.615	6.9	3762	3803
098	off Byron Bay	BS	08/06/17	-28.371, 154.647	-28.389, 154.612	4.0	3811	3754
099	off Byron Bay	BT	09/06/17	-28.371, 154.649	-28.388, 154.617	3.7	3825	3754
100	off Byron Bay	BT	09/06/17	-28.054, 154.083	-28.097, 154.081	4.8	999	1013
101	off Moreton Bay	BT	09/06/17	-26.946, 153.945	-26.971, 153.951	2.8	2520	2576
102	off Moreton Bay	BT	10/06/17	-27.008, 154.223	-27.049, 154.224	4.6	4274	4264
103	off Moreton Bay	BS	10/06/17	-27.000, 154.223	-27.061, 154.223	6.8	4260	4280
104	off Moreton Bay	BT	10/06/17	-26.961, 153.848	-26.991, 153.847	3.3	1071	1138
109	off Fraser Island	BT	11/06/17	-25.221, 154.164	-25.253, 154.192	4.5	4006	4005
110	off Fraser Island	BS	11/06/17	-25.220, 154.160	-25.261, 154.200	6.1	4005	4010
115	off Fraser Island	BT	11/06/17	-25.325, 154.068	-25.351, 154.076	3.0	2350	2342
118**	off Fraser Island	BS						
119	off Fraser Island	BS	12/06/17	-25.206, 153.991	-25.178, 153.979	3.3	2247	2369
121	Coral Sea MP	BT	13/06/17	-23.587, 154.194	-23.617, 154.195	3.3	1013	1093
122	Coral Sea MP	BT	13/06/17	-23.751, 154.639	-23.773, 154.616	3.4	2369	2329
123	Coral Sea MP	BS	13/06/17	-23.749, 154.641	-23.774, 154.617	3.7	2271	2339
128	Coral Sea MP	BT	13/06/17	-23.631, 154.660	-23.659, 154.644	3.5	1770	1761
131	Coral Sea MP	BS	14/06/17	-23.748, 154.643	-23.778, 154.613	4.5	2297	2358
132	Coral Sea MP	BS	14/06/17	-23.756, 154.568	-23.780, 154.540	3.9	2181	2132
134	Coral Sea MP	BS	14/06/17	-23.750, 154.572	-23.774, 154.546	3.8	2093	2156
135	Coral Sea MP	BT	15/06/17	-24.352, 154.291	-24.384, 154.325	5.0	3968	4034

The CSIRO 4 m wide by 0.5 m high beam trawl used to collect megafaunal invertebrates had a net mesh size of 12 mm in the forward section, and 10 mm in the cod end (Lewis 2010). From the time samples were brought aboard the ship to preservation, a ‘cold-chain’ method (Glover et al. 2016) was employed to ensure specimens could be used for future morphological and DNA taxonomy. Substrate and larger specimens from the beam trawl catch were transferred to the wet laboratory on board in containers filled with chilled seawater (5 °C) and material was roughly sorted on ice made from seawater into higher taxonomic categories. Larger clumps of substrate were broken with a hammer and smaller animals were picked from the pieces. Selected specimens were photographed. The majority of the catch was preserved in 95% ethanol, the remainder in 10% buffered formalin, while selected specimens were fixed in RNAlater. When time allowed, for ~ 20% of the catch, tissue samples from selected specimens were taken and fixed in ethanol, while the voucher specimens were fixed in formalin. Larger samples containing numerous annelid tubes were split, half was fixed in ethanol and half in formalin. Operation 100 included a dead pilot whale skull and vertebrae, annelids were picked off the bones and preserved in 95% ethanol.

The Brenke sledge (mesh size 1 mm) was used to collect microbenthic infauna living near the sediment-water interface and more mobile epibenthic fauna (Brenke 2005). Both cod-ends of the Brenke sledge nets were emptied into chilled seawater and visible animals were picked out. The remainder of the sample was elutriated with chilled seawater to separate small animals from sediment, and gently sieved using a 300 µm sieve. Sediment residue was fixed in 95% ethanol and the remaining specimens were hand-picked from residues and sorted under microscopes in the dry lab on board. Selected preserved specimens were also photographed on board. The box core (Hessler and Jumars 1974) was used to collect infaunal invertebrates. Box core sampling was less successful than the trawl and sledge sampling: of five deployments, one deployment resulted in a full sample, the other four resulted in only partial samples or none at all. For successful samples, the top 2 cm sediment layer of the core was elutriated in chilled seawater, sieved using a 300 µm mesh sieve and treated as samples collected by Brenke sledge.

Prior to fixation, all specimens were weighed and registered on board and assigned labels with operation (op) and accession numbers (acc).

Annelid specimens collected during the voyage were shipped to the Australian Museum, Sydney (**AM**), Museums Victoria, Melbourne (**MV**), and the Natural History Museum, London (**NHMUK**) where they were registered and assigned permanent registration numbers of the respective institutions.

### Laboratory identification of annelids

At the respective institutions, annelids fixed in formalin were soaked in water, preserved with 80% ethanol and sorted in 80% ethanol, while ethanol-fixed annelids were sorted in 95% ethanol. Mixed lots of annelids were sorted to families at the AM and MV. Annelid families for which no taxonomic expertise is available in Australia (Acrocirridae, some Ampharetidae, Cirratulidae, Dorvilleidae, Flabelligeridae, Glyceridae, Goniadidae, Lumbrineridae, Maldanidae, some Melinnidae, Opheliidae, Orbiniidae, Paraonidae, Scalibregmatidae, Sphaerodoridae, Spionidae, Sternaspidae, Echiura, and Sipuncula) were sent internationally to taxonomic authorities for species-level identification.

All beam trawl specimens were identified. Brenke sledge and box core material was identified past family level when specimens were large enough (considered adult) and/or complete. Annelids were assigned Latin binomial names where possible or determined in open nomenclature following Sigovini et al. (2016). Species were designated cf. qualifiers to the closest morphological match and not the full species name following the reasons stated in Neal et al. (2020); i) specimens were too damaged or incomplete, ii) the original description was not detailed enough, iii) the described species had a type locality from shallow water or in a different region from Australia (another ocean basin). Some deep-sea species are known to be widespread and in these cases we follow the individual authors of each section as to whether the species is thought to be widespread and thus whether cf. is designated or not. It is important to stress that no formal descriptions of the species are given here, only preliminary identifications and diagnoses (a short written description of the species, which allows that species to be distinguish from other species with which it is likely to be confused). Subsequent taxonomic papers will describe the species and compare them genetically.

The matrix of all annelid species-level abundance and presence data (including beam trawl, box core, and Brenke sledge material) from voyage IN2017_V03 was constructed in MS Excel in standardised Darwin Core format.

## Results

### Taxonomic overview

#### Family Acoetidae Kinberg, 1856

A. Murray

This family of scale worms is characterised by the presence of internal ‘spinning’ glands which produce fibres used to construct their tough fibrous permanent tubes. These fibres often appear as golden strings emerging from the notopodia. Acoetidae are active carnivores and predators, and most frequently collected by fishers on baited lines, in shallow to deep waters (1–200 m). There are currently nine valid genera with 58 nominal species worldwide (Read and Fauchald 2020). In Australian waters they have been collected rarely, and usually only single individuals are found, often as fragments, possibly due to their ability to actively avoid grabs and dredges by rapidly withdrawing into their tubes when detecting vibration in benthic sediments. The few Acoetidae specimens recorded from Australia have all been collected from shallow waters, with the deepest species being *Polyodontes australiensis* (McIntosh, 1885) reported from 120 m off Tasmania (as *Eupompe australiensis*) by Benham (1915), and a specimen of *Euarche* sp., reported as *Eupanthalis* sp., from off Cronulla, New South Wales by Hutchings (2000a) from < 100 m depth. In this study we report one species.

##### Panthalis

Taxon classificationAnimaliaPhyllodocidaAcoetidae

sp.

Fig. 2A

###### Diagnosis.

One damaged specimen, with 24 anterior segments measuring 1.2 cm long, 0.6 mm wide. Head region badly damaged, but some features recognisable: low rounded ommatophores without necks and colourless, a single long median antenna attached mid-prostomium, longer than prostomium length; lateral antennae and palps missing, however; tentaculophores with a few chaetae, styles missing; elytra present on segments 2, 4, 5, 7 and alternating segments thereafter, delicate, transparent. All chaetae simple. Acicular neurochaetae starting from chaetiger 3, notochaetae absent from chaetiger 4 and on all parapodia thereafter. Notopodia with notoaciculum and spinning glands internally, golden ‘spinning’ fibres emergent from the inner surface of the notopodial bract. Superior group of neurochaetae from chaetiger 9 onwards, of two types: long, with plumose (brush) tips, and shorter chaetae with few whorls of short widely spaced hairs along shafts; middle group of neurochaetae stout, acicular chaetae with hairy aristate tips; inferior group of neurochaetae curved, lanceolate, with many transverse rows of overlapping spines along shaft.

###### Remarks.

This specimen possesses brush-tipped neurochaetae typical of the genera *Acoetes* and *Panthalis*, but lacks notochaetae in all middle segments, a feature which distinguishes it as a species of *Panthalis*. The genus *Panthalis* has not yet been reported from Australian waters; however, specimens have been collected previously from deep water in the Arafura Sea off Western Australia and Northern Territory (Murray and Hutchings in prep.).

###### Records.

1 specimen. Suppl. material 1: op. 104 (AM).

**Figure 2.**
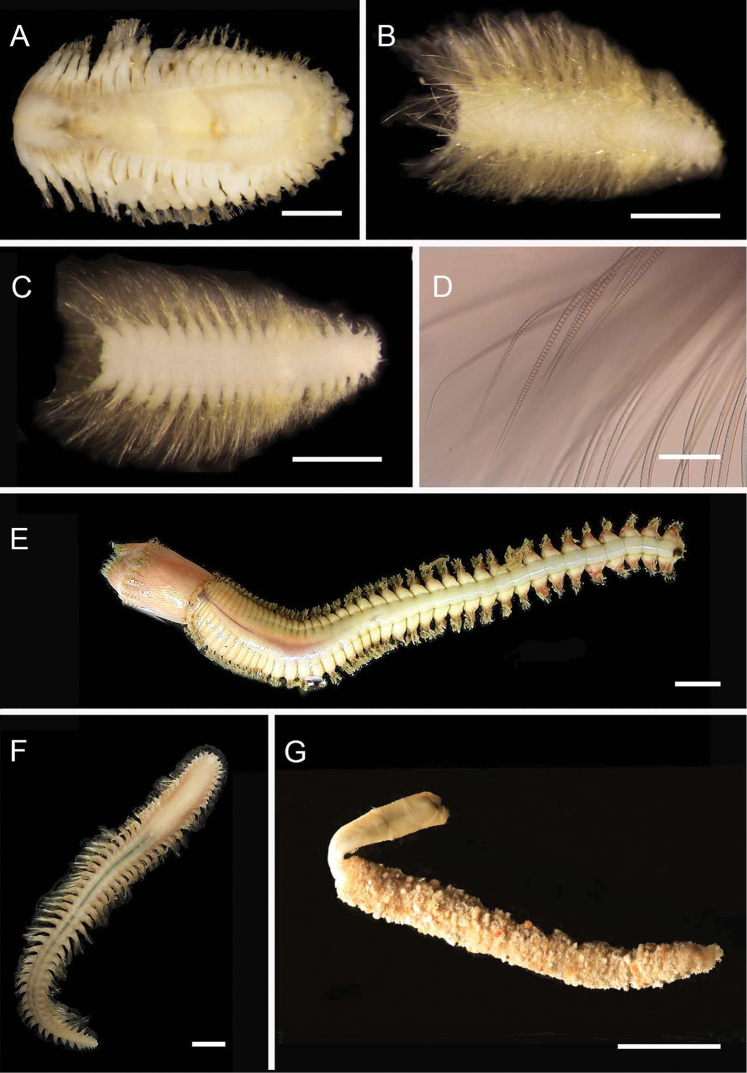
Acoetidae, Chrysopetalidae, Nephtyidae, Oweniidae**A**Acoetidae, *Panthalis* sp., dorsal view (AM W.50321) **B**Chrysopetalidae, Dysponetus cf. caecus, dorsal view **C**Dysponetus cf. caecus, ventral view **D**Nephtyidae, *Aglaophamus* sp. pre- and post-acicular chaetae **E**Nephtyidae, Nephtys cf. paradoxa (AM W.52750) **F**Nephtyidae, *Aglaophamus* sp. **G**Oweniidae, *Myriowenia* sp. in tube (AM W.51842). Scale bars: 2 mm (**A**); 1 mm (**B, C**); 50 µm (**D**); 5 mm (**E, G**); 3 mm (**F**).

#### Family Acrocirridae Banse, 1969

N. Jimi

Acrocirridae are generally small, thread-like or maggot-shaped worms, which are predominantly benthic. There are currently nine valid genera with 43 nominal species (Read and Fauchald 2020). Small deep-sea acrocirrids are very rare and resemble flabelligerids in having a retractile head (Salazar-Vallejo and Gillet 2007; Martínez et al. 2019). The genus *Flabelligena* consists of six species and has been reported from bathyal to abyssal depths. The genus can be distinguished by having 1–3 short branchiae, spinulose notochaetae, and compound neurochaetae (Aguirrezabalaga and Ceberio 2006). *Chauvinelia* consists of two species, both have been recorded from abyssal depths. The genus can be identified by having well-developed cephalic hood, simple notochaetae, and compound neurochaetae (Salazar-Vallejo and Gillet 2007; Martínez et al. 2019). In Australia, only two intertidal species have been described from two genera (*Acrocirrus* and *Macrochaeta*) (Kudenov 1976). Here we report at least one species of *Chauvinelia*, one species of *Flabelligella*, four species of *Flabelligena* and one species of *Swima*. This is the first time these genera have been recorded from Australian waters. Four species of *Flabelligena* are new to science.

##### Chauvinelia

Taxon classificationAnimaliaPhyllodocidaAcoetidae

sp.

Fig. 3E

###### Diagnosis.

Length 1.5 mm, width 0.4 mm, 19 chaetigers, two pairs of branchiae, palps lost. Large ventral papillae present in anterior achaetous segments. Notochaetae elongated, simple, spinous in the tip. Neurochaetae elongated, compound, spinous in the tip.

###### Records.

5 specimens. Suppl. material 1: ops. 23, 98, 110 (AM).

##### Flabelligella

Taxon classificationAnimaliaPhyllodocidaAcoetidae

sp.

###### Records.

1 specimen: Suppl. material 1: op. 40 (NHMUK).

##### Flabelligena

Taxon classificationAnimaliaPhyllodocidaAcoetidae

sp. nov. 1

Fig. 3A

###### Diagnosis.

Length ~ 15 mm, width 1–2 mm, 31–35 chaetigers, prostomium subpentagonal, three pairs of branchiae, two or three spinous notochaetae, one or two composite neurochaetae, short lateral cirri. Body papillae short, with sediment particles. Large ventral papillae absent.

###### Records.

12 specimens. Suppl. material 1: ops. 31, 33, 46, 54, 87 (AM).

**Figure 3.**
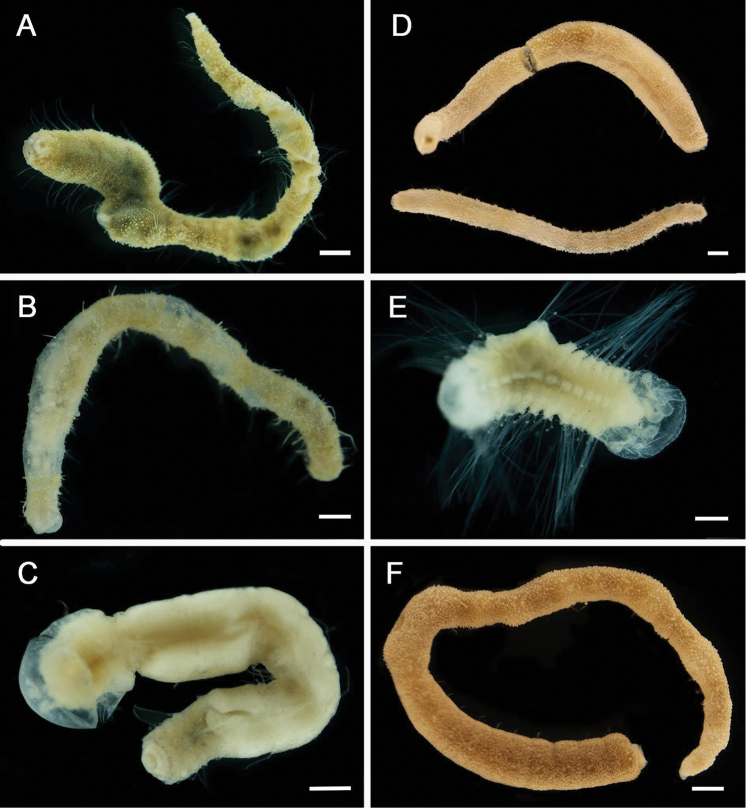
Acrocirridae**A***Flabelligena* sp. nov. 1 (AM W.52559) **B***Flabelligena* sp. nov. 2. (AM W.52561) **C***Flabelligena* sp. nov. 3. (AM W.52560) **D***Flabelligena* sp. nov. 4. (AM W.52828) **E***Chauvinelia* sp. (AM W.52552) **F**Acrocirridae gen. sp. 2 (AM W.52833). Scale bars: 1 mm (**A**); 500 µm (**B–F**).

##### Flabelligena

Taxon classificationAnimaliaPhyllodocidaAcoetidae

sp. nov. 2

Fig. 3B

###### Diagnosis.

Incomplete, length ~ 7 mm, width 0.5 mm, ~ 18 chaetigers, prostomium subpentagonal, three pairs of branchiae, one or two spinous notochaetae, one composite neurochaetae, long lateral cirri in posterior chaetigers. Body papillae short, without attached sediment particles. Large ventral papillae present.

###### Records.

5 specimens. Suppl. material 1: ops. 5, 40, 76 (AM).

##### Flabelligena

Taxon classificationAnimaliaPhyllodocidaAcoetidae

sp. nov. 3

Fig. 3C

###### Diagnosis.

Length ~ 7 mm, width 0.5 mm, 13 chaetigers, prostomium subpentagonal, two pairs of branchiae, one or two spinous notochaetae, one or two composite neurochaetae, short lateral cirri. Body papillae very short, without attached sediment particles. Large ventral papillae absent.

###### Records.

4 specimens. Suppl. material 1: ops. 33, 89 (AM).

##### Flabelligena

Taxon classificationAnimaliaPhyllodocidaAcoetidae

sp. nov. 4

Fig. 3D

###### Diagnosis.

Length ~ 7 mm, width 0.5 mm, 40 chaetigers, prostomium subpentagonal, two pairs of branchiae, three or four spinous notochaetae, 2–4 composite neurochaetae, pair of short lateral cirri. Body papillae very short, with attached sediment particles. Large ventral papillae present.

###### Records.

6 specimens. Suppl. material 1: ops. 23, 119 (AM).

##### Flabelligena spp.

Taxon classificationAnimaliaPhyllodocidaAcoetidae

###### Records.

6 specimens. Suppl. material 1: ops. 16, 54, 98, 110 (NHMUK).

##### Swima

Taxon classificationAnimaliaPhyllodocidaAcoetidae

sp.

###### Records.

2 specimens. Suppl. material 1: op. 134 (NHMUK).

##### Acrocirridae

Taxon classificationAnimaliaPhyllodocidaAcoetidae

gen. sp. 1

###### Diagnosis.

Incomplete, length ~ 7 mm, width 0.4 mm, ~ 17 chaetigers, prostomium subpentagonal, ~ four pairs of branchiae, two or three notochaetae, three or four composite neurochaetae. Body papillae short, without sediment particles. Large ventral papillae absent.

###### Records.

14 specimens. Suppl. material 1: ops. 9, 54, 79, 96, 98 (AM).

##### Acrocirridae

Taxon classificationAnimaliaPhyllodocidaAcoetidae

gen. sp. 2

Fig. 3F

###### Diagnosis.

Incomplete (posterior fragment), length ~ 10 mm, width 0.7 mm, 25 chaetigers, 1–2 spinous notochaetae, one composite short neurochaetae. Body papillae short, with sediment particles. Large ventral papillae absent. Similar to *Flabelligena* sp. 1, but different in neurochaetal shape.

###### Records.

1 specimen. Suppl. material 1: op. 87 (AM).

##### Acrocirridae

Taxon classificationAnimaliaPhyllodocidaAcoetidae

gen. sp. 3

###### Diagnosis.

Incomplete, length ~ 4 mm, width 0.4 mm, 12 chaetigers, 2–3 notochaetae, 2–3 composite neurochaetae. Body papillae short, without sediment particles. Large ventral papillae absent.

###### Records.

1 specimen. Suppl. material 1: op. 79 (AM).

##### Acrocirridae gen. spp.

Taxon classificationAnimaliaPhyllodocidaAcoetidae

###### Remarks.

Samples were identified to family level only or individuals were too fragmented for further analysis.

###### Records.

16 specimens. Suppl. material 1: ops. 31, 54, 55, 56, 76, 87, 96, 98, 119, 134 (AM).

#### Family Ampharetidae Malmgren, 1866

T. Alvestad, L. M. Gunton

Ampharetidae are tubicolous annelids, with a body divided into a distinct thorax and abdomen, unlike the closely related Terebellidae, species of Ampharetidae are able to fully retract buccal tentacles into the mouth. The family Ampharetidae is composed of 64 accepted genera and > 300 species (Ebbe and Purschke 2021). Thirty-two of these genera are monospecific making the taxonomy complex (Eilertsen et al. 2017). Ampharetids are found from intertidal to abyssal depths (Aguirrezabalaga and Parapar 2014; Bonifácio et al. 2015). Deep-water ampharetids are found in high abundance on abyssal plains (Böggemann 2009) and chemosynthesis-based environments such as hydrothermal vents and cold seeps (Reuscher et al. 2009; Eilertsen et al. 2017). The ampharetid fauna of Australia has been poorly studied. To date, five genera (*Amphicteis*, *Auchenoplax*, *Phyllamphicteis*, *Pseudoamphicteis*, and *Neosabellides*) and nine species (Day and Hutchings 1979; Hutchings and Rainer 1979; Hartmann-Schröder 1981; Alvestad and Budaeva 2015) have been recorded from Australian waters. These Australian records are primarily from shallow waters (< 100 m); however, ampharetids are known to be well-represented in deep-sea benthic samples, indicating that a high number of Australian ampharetid species are yet to be described. In this study > 300 ampharetid specimens belonging to more than six species were recovered from the Australian lower bathyal and abyssal environment, at least four are new to science.

##### Amage

Taxon classificationAnimaliaPhyllodocidaAmpharetidae

sp. nov. 1

Fig. 4A

###### Diagnosis.

Length 12 mm, width 4 mm. Body short, thick, with a short abdomen. Prostomium complex; central part drawn out into two lateral horns, lateral parts form large lobes while front part forms a ‘lip’. No glandular ridges or eyes. Approximately three pairs of branchiae in a transverse line in two widely separate groups. No paleae. Fourteen thoracic segments with notopodia with chaetae. First three pairs of notopodia and chaetae small. Thoracic uncini from segment VI. Eleven thoracic uncinigers. Nine abdominal uncinigers. Abdomen with rudimentary notopodia. Pygidium without lateral cirri.

###### Records.

1 specimen. Suppl. material 1: op. 53 (AM).

**Figure 4.**
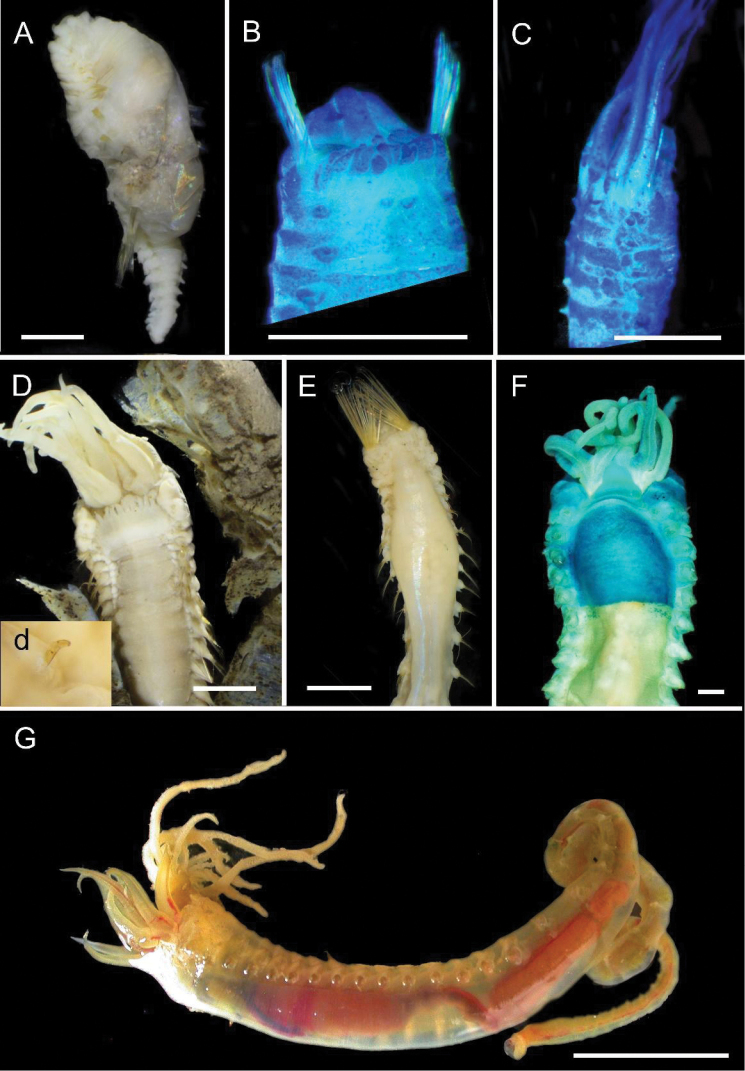
Ampharetidae and Melinnidae. Ampharetidae**A***Amage* sp. nov. 1 **B***Anobothrus* sp. nov. 1, anterior end methyl blue staining **C***Anobothrus* sp. nov. 2 anterior end methyl blue staining. Melinnidae**D**Melinna cf. armandi and tube (AM W.50354), **d** dorsal hook. Ampharetidae**E***Amphicteis* sp. (AM W. 50429). Melinnidae**F***Melinnopsis gardelli* anterior dorsal view (AM W.50735) **G***Melinnopsis* sp. nov., lateral view (AM W.50397). Scale bars: 2 mm (**A**); 1 mm (**B, C**); 2.5 mm (**D**); 1 cm (**E, G**); 1 mm (**F**).

##### Amage tasmanensis

Taxon classificationAnimaliaPhyllodocidaAmpharetidae

(Holthe, 2000)

###### Diagnosis.

Length 16–30 mm, width 3–5 mm. Widest at branchial region. Thorax long and cylindrical, not tapering towards abdomen. Abdomen short; half length of thorax tapering towards pygidium. Prostomium without glandular ridges or eyes. Distal part of prostomium with longitudinal folds. Ventral surface of buccal segment with longitudinal folds. Four pairs of branchiae arranged as three middle pairs, almost in a transverse line, and one outer pair behind the outermost of the inner branchiae. Right and left branchial group separated by a space more or less equal to width of one branchia. Large lateral lobes on segment II. No paleae. Third segment with rudimentary notopodia with a few extremely small chaetae. Fourth and fifth segment with small notopodia with a few very short chaetae. Sixth to 16^th^ segment with normal sized notopodia and notochaetae. Fourteen thoracic segments with notochaetae. Thoracic uncini from segment VI. Eleven thoracic uncinigers. Approximately 12 abdominal uncinigers. Abdomen with rudimentary notopodia. Pygidium with a pair of lateral cirri with thick bases and slender tips.

###### Remarks.

The holotype of *Amage tasmanensis* was collected from 3830 m in the Tasman Sea. Due to the matching morphology and close proximity of the specimens from this study to the collection location of the holotype, we assign the name *Amage tasmanensis*.

###### Records.

29 specimens. Suppl. material 1: ops. 32, 35, 53 (AM).

##### Amphicteis sp. nov.

Taxon classificationAnimaliaPhyllodocidaAmpharetidae

Fig. 4E

###### Diagnosis.

Length 20–30 mm, 3 mm at widest section. Paired longitudinal glandular ridges curving slightly sidewise anteriorly. Paired transverse nuchal ridges separated by median gap, ridges at right angle to each other. Buccal tentacles and branchiae missing on both specimens. Chaetae on segment II modified to golden paleae extending past prostomium. Seventeen thoracic chaetigers including paleae, 15 abdominal chaetigers including pygidium. Anal cirri absent.

###### Records.

2 specimens. Suppl. material 1: ops. 67, 78 (AM).

##### Anobothrus

Taxon classificationAnimaliaPhyllodocidaAmpharetidae

sp. nov. 1

###### Diagnosis.

Length 14 mm, width 1 mm. Prostomium trilobed. Median lobe, narrow and protruding, delimited by deep lateral grooves. Eye spots present. Three pairs of branchiae. Branchiae arranged in transverse row without median gap. Branchiophores fused at base, forming a characteristic and well-marked edge/fold above head. Long filiform paleae. Thorax and abdomen of similar length. Fifteen thoracic segments with notopodia and capillary chaetae. Last 12 chaetigers of thorax with neuropodia and uncini. Notopodia on thoracic unciniger 8 slightly elevated and connected with a ciliated band. Tube a thin layer of secretion loosely incrusted with mud and foraminifera.

###### Records.

1 specimen. Suppl. material 1: op. 22 (AM).

##### Anobothrus

Taxon classificationAnimaliaPhyllodocidaAmpharetidae

sp. nov. 2

Fig. 4C

###### Diagnosis.

Incomplete, 9 mm length, 1 mm width. Specimens not in a good condition. Not possible to discern characters on the prostomium or count segments. Conical prostomium. Long filiform paleae. Space between the two groups of branchiae similar to width of one branchia. Tube a thin layer of secretion loosely incrusted with mud and foraminifera.

###### Records.

2 specimens. Suppl. material 1: op. 56 (AM).

##### Jugamphicteis galatheae

Taxon classificationAnimaliaPhyllodocidaAmpharetidae

Holthe, 2000

###### Diagnosis.

Length 25–40 mm, width 2–3 mm. Prostomium with four curved nuchal arches. Body long tapering towards pygidium. Four pairs of branchiae. Paleae present, long golden extend past rim of prostomium. First abdominal segment with dorsal fan with large median notch.

###### Remarks.

The holotype of *Jugamphicteis galatheae* was collected from Kermadec Trench in the South Pacific Ocean ~ 4500 m; however, paratypes were recovered from both the Kermadec Trench and off the east coast of South Africa between Cape Town and Durban ~ 5000 m. The species is reported to have a wide distribution, which may indicate a species complex. Due to the matching morphology and close proximity of the specimens from this study to collection location of the holotype, we assign the name *Jugamphicteis galatheae*.

###### Records.

40 specimens. Suppl. material 1: ops. 6, 15, 30, 32, 43, 53, 65, 86, 121 (AM).

##### Ampharetidae gen. spp.

Taxon classificationAnimaliaPhyllodocidaAmpharetidae

###### Remarks.

Beam trawl specimens were incomplete which does not allow further identification, while Brenke sledge samples were identified to family level.

###### Records.

260 specimens. Suppl. material 1: ops. 9, 16, 22, 23, 30, 31, 33, 40, 42, 43, 45, 46, 54, 55, 56, 65, 76, 79, 88, 89, 90, 96, 98, 100, 101, 103, 110, 119, 123, 134 (AM). 7 specimens. Suppl. material 1: ops. 16, 31 (NHMUK).

#### Family Amphinomidae Lamarck, 1818

L. M. Gunton, D. Ramos, R. S. Wilson

The family Amphinomidae is characterised by simple calcareous chaetae, in some species these chaetae are very fragile breaking off if touched and causing a burning sensation giving them the common name, ‘fireworms’. The family is divided in to two subfamilies, Archinominae Kudenov, 1991 and Amphinominae Lamarck, 1818 based on the presence of accessory dorsal cirrus in the former and absence in the latter. Currently, there are 23 genera containing 148 valid species (Read and Fauchald 2020). Amphinomids occur worldwide from intertidal to abyssal depths, they are predominantly associated with shallow reefs, rocky and soft bottoms of intertidal and continental shelf habitats, comparatively few have been recorded from the deep sea (Rouse and Pleijel 2001), but some are associated with deep-sea chemosynthetic environments (Borda et al. 2012, 2013). There are > 1700 records of amphinomids in Australian waters but only nine species from seven genera have been listed (http://www.ala.org.au). In this study, at least four species were recovered, three may be new to science.

##### Bathychloeia cf. sibogae

Taxon classificationAnimaliaPhyllodocidaAmphinomidae

Horst, 1910

###### Diagnosis.

Body short, ovate, ~ 8 mm in length. 17–18 chaetigers bearing long (3–44 mm) furcate chaetae. Body pale colour, pair of purple spots dorsal on chaetiger 6, visible under skin. Dark blue-black colouration visible under skin on chaetigers 10–13, dorsal and ventral. Caruncle lobed, extending to chaetiger 3. Branchiae branched, only found on chaetiger 5. Parapodia short, but neuro-and notochaetae well separated, neurochaetae lateral. Notochaetae dorsal (remaining tuft on chaetiger 7). Parapodial cirri on all (?) chaetigers, longer on final five. Chaetae long, bifurcate. No serrations or harpoon chaetae. Neurochaetae shorter than notochaetae. Faint membrane/covering visible over the furcate tips of some chaetae. Pygidium with thick anal cirrus, may be part of a pair.

###### Remarks.

The type locality of *Bathychloeia sibogae* is in the Banda Sea, Malay Archipelago 1158 m depth. Böggemann (2009) redescribed the species using the type material, material from the Canaries (~ 2800 m) and material from the abyssal SE Atlantic (~5000 m). The species is also recorded from 12 stations (138–2074 m) in the GAB (MacIntosh et al. 2018: additional file 2). Due to the species broad distribution it is highly likely a species complex and thus we assign the name Bathychloeia cf. sibogae.

###### Records.

4 specimens: Suppl. material 1: ops. 96, 102, 103 (AM). 2 specimens Suppl. material 1: op. 110 (NHMUK).

##### Linopherus

Taxon classificationAnimaliaPhyllodocidaAmphinomidae

sp. 1

Fig. 5A, B

###### Diagnosis.

Prostomium divided into two. Posterior portion pentagonal with medial antennae on posterior edge, flanked laterally by the first chaetiger. Anterior section round with antennae and palps reduced to small bumps anterolaterally and laterally respectively. Body small, slightly wider anteriorly and tapering posteriorly. Eyes absent. First chaetiger reduced, not continuous dorsally. Papilliform notopodial postchaetal lobe present throughout. Bipinnate branchiae present on chaetigers 3–5.

###### Remarks.

*Linopherus* sp. 1 differs from a second species of *Linopherus* known from the GAB (MacIntosh et al. 2018: additional file 2) in having branchiae first present on chaetiger 3 rather than chaetiger 4 in GAB specimens.

###### Records.

1 specimen. Suppl. material 1: op. 100 (NHMUK).

**Figure 5.**
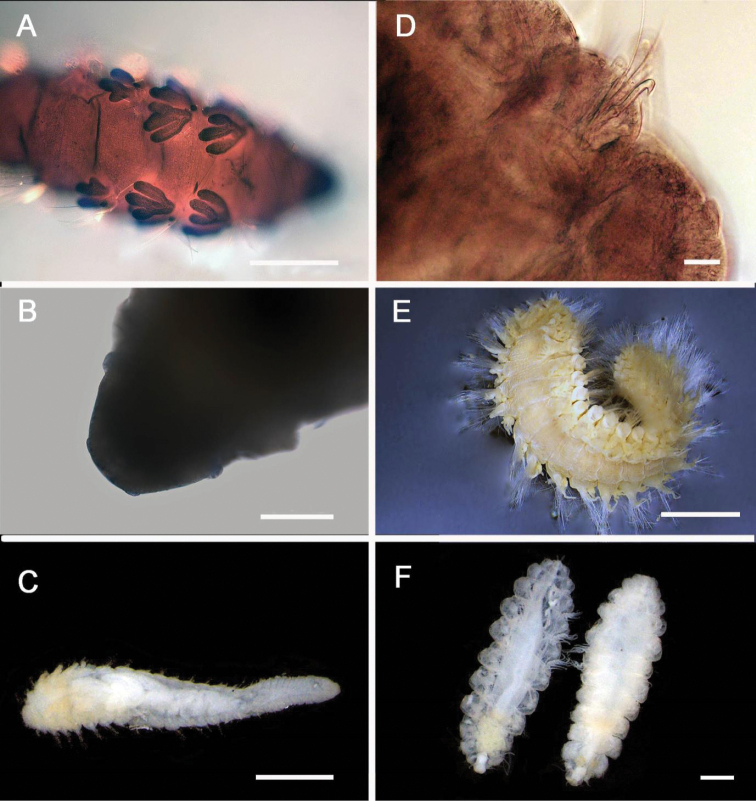
Amphinomidae**A***Linopherus* sp. 1, bipinnate branchiae **B***Linopherus* sp. 1, antennae and palps **C**Paramphinome cf. australis**D**Paramphinome cf. australis, hooks **E***Pareurythoe* sp. (AM W.52611) **F**Amphinomidae gen. sp. juveniles (AM W.52607). Scale bars: 250 µm (**A**); 100 µm (**B**); 1 mm (**C, F**); 50 µm (**D**); 3 mm (**E**).

##### Paramphinome cf. australis

Taxon classificationAnimaliaPhyllodocidaAmphinomidae

Monro, 1930

Fig. 5C, D

###### Diagnosis.

Body shape elongate ~ 3 mm length. Eyes absent. Prostomium rounded. One or two strongly curved hooks on chaetiger 1 (Fig. 5D) depending on body size (smaller individuals one hook, larger individuals two hooks). Arborescent branchiae beginning on chaetigers 4–7. Notochaetae capillary chaetae with step-like serrations and smooth unadorned spines. Notoacicula two per fascicle. Neurochaetae long thin capillaries with basal spurs, long thin capillaries no basal spurs, subdistally inflated bifurcate chaetae serrated prongs. Neuroacicula two per fascicle. Pygidium unadorned.

###### Remarks.

A redescription of *Paramphinome australis* is given in Böggemann (2009). Current specimens differ from *Paramphinome australis* in the number of strongly curved hooks numbering one or two, not two or three as in Böggemann (2009). No difference in thickness of notochaetae spines, whereas in Böggemann (2009) notochaetal spines are thicker in outer and thinner in inner positions. The type locality of *Paramphinome australis* is Antarctic Ocean off South Orkney Islands 244–344 m, Böggemann’s (2009) redescription was based on samples from Angola Basin 3945–3992 m, the distribution is recorded as from Antarctic and Subantarctic regions from subtidal to abyssal depths. This broad distribution suggests a species complex.

###### Records.

40 specimens. Suppl. material 1: op. 100 (AM).

##### Pareurythoe

Taxon classificationAnimaliaPhyllodocidaAmphinomidae

sp.

Fig. 5E

###### Diagnosis.

Body shape elongate (with parallel sides). Notochaetae in dorsal tufts. Caruncle inconspicuous. Caruncle median ridge absent. Branchiae as tufts from chaetiger 3.

###### Remarks.

Also known from six stations (189–2867 m) in the GAB (MacIntosh et al. 2018: additional file 2).

###### Records.

2 specimens. Suppl. material 1: op. 69 (AM).

##### Amphinomidae gen. spp.

Taxon classificationAnimaliaPhyllodocidaAmphinomidae

Fig. 5F

###### Remarks.

Samples identified to family level only as individuals too damaged for further analysis, or juveniles (Fig. 5F). Specimens from op. 110 may be juveniles, size 5 mm length, 2 mm width.

###### Records.

5 specimens. ops. 16, 110 (AM).

#### Family Aphroditidae Malmgren, 1867

A. Murray, R. S. Wilson

Aphroditidae is a family of scale-worms commonly referred to as ‘sea mice’ due to their hairy appearance. Currently, there are seven genera containing 104 species (Read and Fauchald 2020). The family is well-represented in both the deep sea and in shallow waters. They tend to be large animals often caught in trawls and grabs. Although 18 species in five genera have been recorded from Australian waters in a revision by Hutchings and McRae (1993), only four of these species have been reported from depths > 400 m: *Laetmonice producta* Grube, 1877; *Laetmonice yarramba* Hutchings & McRae, 1993, *Aphrodita goolmarris* Hutchings & McRae, 1993, and *Aphrodita malkaris* Hutchings & McRae, 1993. In this study five species from two genera (*Aphrodita* and *Laetmonice*) are reported, one species is believed to be undescribed.

##### Aphrodita cf. talpa

Taxon classificationAnimaliaPhyllodocidaAphroditidae

Quatrefages, 1866

Fig. 6A, B

###### Diagnosis.

Body shape ovate, length less than twice maximum width. Specimens with dorsal felt of fine notochaetae covering and obscuring elytra; 15 pairs elytra, elytral surface with micropapillae. Prostomium rounded, without ocular peduncles, eye pigment absent (may be present), nuchal flaps absent; facial tubercle well-developed, ~ same length as prostomium, papillate. Median antenna long, thin, as long as prostomium, with ceratophore ~ one third the length of style; palps long, minute papillae present. Notochaetae of three kinds: capillary chaetae forming matted dorsal felt; iridescent capillary chaetae projecting laterally; and stout, golden acicular spines with fine tubercles and hairs and with fine curved/hooked tips. Neurochaetae stout, superior tier thicker, brown with pilose margin and smooth slightly curved naked tip, inferior tier similar but golden brown and thinner than upper neurochaetae, with thickly pilose margin and slightly curved naked tips.

###### Remarks.

This species may be undescribed; it differs from *Aphrodita talpa* Quatrefages, 1866 (described from New Zealand) in having an elongate median antenna, hirsute notochaetae, iridescent capillary notochaetae, and lacking hastate neurochaetae. It has previously been reported from a number of locations around Australia at depths of 17–171 m as *Aphrodita talpa* by Hutchings and McRae (1993), who also suggest that it may be a complex of species due to its morphological variability. It is not clear if material reported from the Tasman Sea from depths of 186–526 m by Averincev (1978) represents *Aphrodita talpa* Quatrefages, 1866 or *Aphrodita talpa* sensu Hutchings & McRae, 1993 and so we prefer to use Aphrodita cf. talpa.

###### Records.

10 specimens. Suppl. material 1: ops. 4, 22, 35, 44, 56 (AM). 4 specimens. Suppl. material 1: op. 4 (MV).

**Figure 6.**
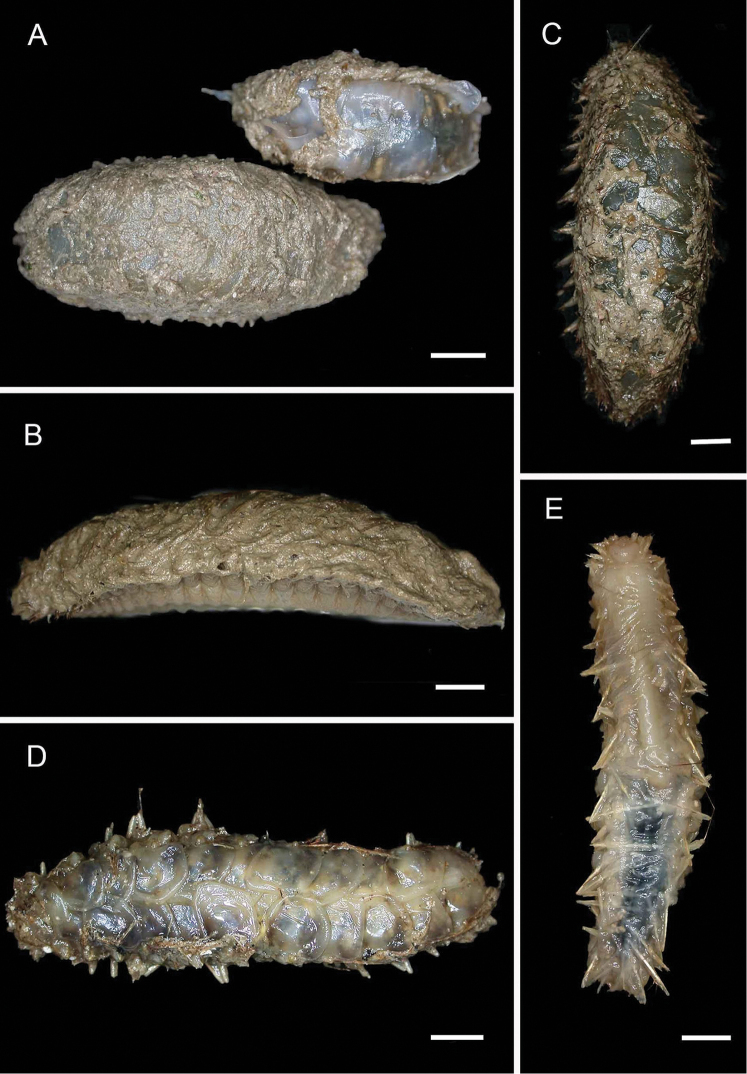
Aphroditidae**A**Aphrodita cf. talpa (NMV F.293294) dorsal view **B**Aphrodita cf. talpa (AM W.49509) lateral view **C***Aphrodita goolmarris* (AM W.52604) dorsal view **D***Laetmonice yarramba* dorsal view (AM W.49499) **E***Laetmonice yarramba* ventral view (AM W.49500). Scale bars: 1 mm (**C**); 5 mm (**A, B, D, E**).

##### Aphrodita goolmarris

Taxon classificationAnimaliaPhyllodocidaAphroditidae

Hutchings & McRae, 1993

Fig. 6C

###### Diagnosis.

Large-bodied specimens, body shape ovate, length less than twice maximum width. Thin dorsal felt of fine notochaetae covering elytra; 15 pairs elytra, elytral surface with micropapillae. Prostomium rounded, without ocular peduncles, with raised ocular areas, pigment absent; facial tubercle well-developed, inflated, with small papillae. Palps extending to segment 11 with minutely papillated margins. Median antenna rod-shaped, fifth length of prostomium. Notochaetae of three kinds: capillary chaetae forming matted dorsal felt; stout, golden-brown, smooth chaetae curving over dorsum; and lateral short tuft of faintly iridescent capillary notochaetae, not forming a fringe. Neuropodia with three tiers of chaetae: stout, superior tier with two stout dark, pilose-tipped acicular neurochaetae, middle tier with 4–9 similar chaetae, inferior tier with 8–15 similar chaetae. Some anterior segments with non-pilose acicular neurochaetae with smooth margins and tips. Numerous golden-yellow, bipinnate neurochaetae present in chaetigers 2 and 3 in inferior position.

###### Remarks.

This species is recorded from Western Australia (WA), New South Wales (NSW) and Queensland (QLD) (Cape York) in depths of 353–3058 m (Hutchings and McRae 1993). It has also been recently collected from the GAB voyages (MacIntosh et al. 2018: additional file 2).

###### Records.

4 specimens. Suppl. material 1: ops. 4, 14, 67 (AM).

##### Laetmonice benthaliana

Taxon classificationAnimaliaPhyllodocidaAphroditidae

McIntosh, 1885

###### Diagnosis.

Body shape elongate, > twice as long as maximum width. Number of chaetigers 33–34. Dorsal felt of fine notochaetae absent. Fifteen pairs of elytra, elytral surface smooth. Elytra comments: some inconspicuous brown pigmented spots in the middle. Prostomium rounded, facial tubercle large and visible dorsally. Facial tubercle papillate. Eye pigmentation absent. Eyes located on elongate ocular peduncles, longer than wide. Median antenna bi-articulate with basal ceratophore and elongate style. Median antenna ceratophore length ~ as long as prostomium. Median antenna ceratostyle length much longer than prostomium (3–6 × as long). Palp surface smooth, palps extending to segment 15. Ventrum with sparse cover of papillae, appearing almost smooth. Ventral cirri of mid-body chaetigers short, not reaching base of neurochaetae. Dorsal notochaetae harpoon-like, with several barb-like tips. Harpoon notochaetae (stout with barbed tips) present. Harpoon notochaetae shaft with fine granulations. Prominent basal spur on neurochaetae present. Spur-neurochaetae of (mid-body segments) subdistal teeth absent. Spur-neurochaetae subdistal hairs present. Marginal hairs not reaching the tip of the spur but leaving a significant basal gap.

###### Remarks.

In this study the species was recorded from 2751–2820 m; other records from the Australian region include RV ‘Dmitry Mendeleev’ voyage 16 stations 1372 and 1373 in the GAB (700–1976 m) by Averincev (1978) and RV ‘Galathea’ stations 601, 697 in the Tasman Sea (3580–4400 m) by Kirkegaard (1995).

###### Records.

4 specimens. Suppl. material 1: op. 4 (MV).

##### Laetmonice yarramba

Taxon classificationAnimaliaPhyllodocidaAphroditidae

Hutchings & McRae, 1993

Fig. 6D, E

###### Diagnosis.

Specimens dorsally with debris entangled in felted notochaetae, sometimes obscuring elytra, but dorsal felt not covering elytra, elytra 12–15 pairs. Prostomium with short ocular peduncles, eye pigment absent; facial tubercle well-developed, papillate; nuchal flaps absent. Palps extending to segments 13 and 14, margins finely papillate. Median antenna with ceratophore half length of prostomium; antennal style long thin, > 3 × length of prostomium. Notochaetae of three kinds: golden, curved, smooth, acicular chaetae arched over dorsum; stout, long harpoon chaetae with three recurved fangs below tips, shafts tuberculate on some specimens; tuft of fine mud-covered chaetae ventrally. Neurochaetae in two tiers: superior tier of 2–4 yellow acicular chaetae with basal spur and subdistal fringe of hairs, inferior tier with numerous yellow bipinnate chaetae. Ventrum with small papillae present, or may be absent.

###### Remarks.

Specimens range from 0.2 mm to 7 cm in length. Some individuals are badly damaged, with chaetae missing, and other intact specimens show some morphological variability from the original description by Hutchings and McRae (1993) in the longer palps, and some with fewer than 13 pairs of elytra. However, they most resemble *L. yarramba*, but share some similarities with *L. producta* Grube, 1877 (which is also considered to be species complex by Hutchings and McRae 1993), such as tuberculate shafts of the harpoon chaetae, but from which it can be distinguished by the fewer pairs of elytra and the absence of nuchal flaps on the prostomium. Widespread around Australia in depths of 60–3950 m, including records from the GAB (MacIntosh et al. 2018: additional file 2).

###### Records.

354 specimens. Suppl. material 1: ops. 4, 13, 15, 22, 23, 30, 31, 32, 33, 35, 42, 43, 44, 53, 56, 65, 70, 86, 115 (AM). 4 specimens. Suppl. material 1: op. 4 (MV).

##### Laetmonice cf. producta

Taxon classificationAnimaliaPhyllodocidaAphroditidae

sensu Hutchings & McRae, 1993

###### Diagnosis.

Large-bodied specimen, body shape elongate, > twice as long as maximum width. Dorsal felt of fine notochaetae absent, 18 pairs elytra with purple colouration on inner halves. Prostomium with pair of large ocular peduncles, without eye pigment; facial tubercle well-developed, with long conical papillae; small nuchal flaps present. Palps extending to segment 11, margins finely papillate. Median antenna with ceratophore half the length of the prostomium; antennal ceratostyle longer than prostomium, slender, clavate-tipped, 3 × length of prostomium. Notochaetae of three kinds: ~ 15 smooth, golden, unidentate acicular chaetae; ~ ten long, stout, yellow brown harpoon-like chaetae with 3–5 recurved fangs below tips, shafts smooth or tuberculate; tuft of short, fine mud-covered capillary chaetae ventrally. Neurochaetae in two tiers: superior tier of yellow acicular chaetae with basal spur and subdistal fringe of long hairs and bare tips, inferior tier with numerous golden bipinnate neurochaetae. Ventrum covered with small papillae.

###### Remarks.

This specimen agrees well with the description of other Australian specimens assigned to *Laetmonice producta* Grube, 1877 by Hutchings & McRae (1993), who stated that the species displayed much morphological variability and had a broad distribution. Grube originally described *L. producta* from the area of the Kerguelen Islands in the Southern Ocean. McIntosh (1885, 1900) described several “varieties” of *L. producta*, ranging from the Azores to Antarctic waters, most of which were subsequently raised to species level by Chamberlin (1919). However, Hartman (1965) suggested that the variety represented by Grube’s original species (now known as the subspecies *L. producta producta*, see Read and Fauchald 2020) was restricted in distribution to the area of Kerguelen Islands, the South Georgia Islands and the Antarctic Peninsula. The records from southeastern Australian waters were assigned to *L. producta* by Hutchings and McRae (1993) because they were unable to examine the type specimens of McIntosh’s “varieties”, and they suggest that it may possibly belong to another new species. The specimen examined herein differs from *L. producta producta* Grube, 1877 by the number of elytra (18, not 20 pairs), the lack of eye pigment, smaller nuchal flaps, and the shafts of the harpoon notochaetae which may be smooth or finely granulated. Live specimens are usually pale, with a longitudinal purple stripe mid-dorsally.

###### Records.

1 specimen. Suppl. material 1: op. 14 (AM).

#### Family Capitellidae Grube, 1862

L. M. Gunton

Capitellids resemble terrestrial earthworms due to their simple cylindrical body shape, lack of head appendages and often reduced parapodia. The family contains 44 genera and ~ 186 species (Magalhães and Blake 2019). Capitellids are common and widespread annelids that occur in every marine habitat from intertidal to hadal depths. In Australia, Capitellidae fauna comprises of at least 37 species in 18 genera (Hutchings 2000b). Unfortunately, the capitellids collected from the present study were damaged. Complete or nearly complete specimens are needed for generic diagnosis, furthermore, no taxonomic expertise in the family was available. Two genera (*Capitella* and *Notomastus*) are reported here.

##### Capitella spp.

Taxon classificationAnimaliaPhyllodocidaCapitellidae

###### Records.

7 specimens. Suppl. material 1: op. 88 (AM).

##### Notomastus spp.

Taxon classificationAnimaliaPhyllodocidaCapitellidae

###### Remarks.

A single OTU provisionally referred to *Notomastus* was recorded from six stations (437–3771 m) in the GAB (MacIntosh et al. 2018: additional file 2), further investigation is needed to understand if the species from the present study are the same as the GAB specimens.

###### Records.

2 specimens. Suppl. material 1: ops. 30, 33 (AM).

##### Capitellidae gen. spp.

Taxon classificationAnimaliaPhyllodocidaCapitellidae

###### Remarks.

Specimens not identified beyond family level.

###### Records.

19 specimens. Suppl. material 1: ops. 16, 27, 31, 33, 40, 46, 66, 76, 134 (AM).

#### Family Chaetopteridae Audouin & Milne Edwards, 1833

L. M. Gunton, J. Zhang

Chaetopterids are characterised by a pair of long palps and a body divided into three distinct regions. Some species produce bright blue luminescent mucus. Currently there are five genera and 73 accepted species (Read and Fauchald 2020). Chaetopterids are found from intertidal to abyssal depths. The chaetopterid fauna is poorly studied in Australian waters, with only eight named species from four genera (*Chaetopterus*, *Mesochaetopterus*, *Phyllochaetopterus* and *Spiochaetopterus*) reported (http://www.ala.org.au). The material from the present study was in poor condition, only one genus, *Phyllochaetopterus*, was recognised.

##### Phyllochaetopterus spp.

Taxon classificationAnimaliaPhyllodocidaChaetopteridae

###### Records.

7 specimens. Suppl. material 1: ops. 6, 41, 53, 78 (AM).

##### Chaetopteridae gen. spp.

Taxon classificationAnimaliaPhyllodocidaChaetopteridae

###### Remarks.

The specimens were too fragmented to be identified further.

###### Records.

2 specimens. Suppl. material 1: ops. 88, 134 (AM). Tubes only. Suppl. material 1: ops. 65, 104 (NHMUK).

#### Family Chrysopetalidae Ehlers, 1864

C. Watson

Chrysopetalids are distinguished by broad notochaetal, leaf-like paleae and/or notochaetal spines in fans covering the dorsum. Chrysopetalidae currently contains 29 genera and ~ 110 species (Watson 2020). There are three subfamilies, paleate Chrysopetalinae, spinous Dysponetinae, and putatively lacking spinous notochaetae or notochaetae Calamyzinae. Abyssal Chrysopetalinae taxa have been described from hydrothermal vents (e.g., *Thrausmatos* Watson, 2001) and wood falls (e.g., *Strepternos* Watson Russell, 1991). Dysponetinae taxa have been described from abyssal plains of the East Atlantic (Böggemann 2009). Free-living Calamyzinae taxa occur in seep and whale fall communities, e.g., *Micospina auribohnorum*Watson et al. 2016 from the East Pacific, and symbiotic Calamyzinae taxa from vent bivalve hosts, e.g., *Nautilina calyptogenicola* Miura & Laubier, 1989 from the West Pacific. No Calamyzinae taxa have yet been recorded from Australian waters. Chrysopetalinae taxa from southern Australia, shelf to ~1000 m, are currently being described (Watson in prep.). In this abyssal study we report one species, Dysponetus cf. caecus.

##### Dysponetus cf. caecus

Taxon classificationAnimaliaPhyllodocidaChrysopetalidae

(Langerhans, 1880)

Fig. 2B, C

###### Diagnosis.

Prostomium truncate, with three short antennae and two short palps. No eyes. Large mouth cirrus and pair of rod-like stylets. Two pairs of cirriform tentacular cirri on segments 1 and 2; segments one and two fused; dorsal cirri segment 1 very long, notopodia 2 with notochaetae. Mid-body notopodia with two lengths of semi-erect, golden-coloured notochaetal fascicles: short, broad and long, slender, spines with two rows of spinelets; elongate cirrophores and long dorsal styles. Neuropodia with fascicle of very slender falcigerous neurochaetae with very long shafts; ventral cirri longer than neuropodia.

###### Remarks.

Similar to, but differs from abyssal East Atlantic Dysponetus cf. caecus (sensu Böggemann 2009; Watson et al. 2014) in possession of broad short spines with less spinulation. *Dysponetus caecus* is currently considered a global species complex.

###### Records.

48 specimens. Suppl. material 1: ops. 11, 23, 40, 76, 98, 110, 134 (AM). 2 specimens. Suppl. material 1: op. 100 (NHMUK).

#### Family Cirratulidae Ryckholt, 1851

J. A. Blake

Cirratulids possess many anterior tentacular filaments and numerous long filamentous branchiae along their body which gives them a frilly appearance. Currently there are 11 genera and ~ 277 accepted species (Blake and Magalhães 2019). Although abundant in deep-water (Blake et al. 2009), cirratulids are not well known from abyssal depths. Blake (2019a) provides the first known account of an abyssal cirratulid fauna from the equatorial Pacific Ocean where 12 endemic species are described.

Cirratulids are divided into (1) bitentaculate genera, having a narrow head consisting of a distinct prostomium and peristomium, a pair of long dorsal tentacles and branchiae along most of the body, and (2) multitentaculate genera having a wedge-shaped head and numerous dorsal tentacles arising from anterior segments (Blake and Magalhães 2019). Nearly all deep-sea cirratulids are bitentaculates, except at some vent and seep sites where multitentaculates are found. In Australian waters 15 species from ten genera have been recorded (http://www.ala.org.au). Most cirratulid material in this study was in poor condition, few were complete, with most specimens having lost their posterior ends. The posterior segments and the anterior end morphology are critical in cirratulid systematics to identify not only the species, but also the genus. In this study we report five genera and at least seven new species: undescribed species include *Aphelochaeta* (three species), *Chaetocirratulus* (one species), *Chaetozone* (two species), and *Kirkegaardia* (one species).

##### Aphelochaeta spp. nov.

Taxon classificationAnimaliaPhyllodocidaCirratulidae

###### Remarks.

At least three new species of *Aphelochaeta* are present among these samples. Four OTUs of the genus *Aphelochaeta* were recorded from the GAB (189–2867 m) (MacIntosh et al. 2018, additional file 2). Further investigation is needed to understand if any of the GAB species match those found in this study. None agree with six new species reported by Blake (2019a) from the abyssal Pacific Ocean.

###### Records.

16 specimens. Suppl. material 1: ops. 16, 31, 33, 40, 54, 98 (AM).

##### Chaetocirratulus sp. nov.

Taxon classificationAnimaliaPhyllodocidaCirratulidae

###### Remarks.

A single specimen, believed to be a new species, is similar to *Chaetocirratulus pinguis* (Hartman, 1978) from Weddell Sea, Antarctica re-described by Blake (2018).

###### Records.

1 specimen. Suppl. material 1: op. 87 (AM).

##### Chaetozone spp. nov.

Taxon classificationAnimaliaPhyllodocidaCirratulidae

###### Remarks.

One distinct new species is similar to *Chaetozone brunnea* described by Blake (2006) from deep water off California. However, this new species has distinctive abdominal moniliform segments. Another more typical *Chaetozone* species is also present. Two *Chaetozone* OUTs were recorded from the GAB (141–2012 m) (MacIntosh et al. 2018: additional file 2). Further investigation is needed to understand if any of the GAB species match those found in this study.

###### Records.

13 specimens. Suppl. material 1: ops. 31, 33, 54, 79, 134 (AM).

##### Kirkegaardia sp. nov.

Taxon classificationAnimaliaPhyllodocidaCirratulidae

###### Remarks.

At least one new species of *Kirkegaardia* is present. The specimens have a long smooth peristomium, typical of several species of this genus (Blake 2016). The characteristic serrated capillary chaetae found in *Kirkegaardia* are largely sheared off on the available specimens.

###### Records.

3 specimens. Suppl. material 1: ops. 16, 28, 33 (AM).

##### Cirratulidae gen. spp.

Taxon classificationAnimaliaPhyllodocidaCirratulidae

###### Remarks.

Brenke sledge samples were identified to family level.

###### Records.

7 specimens. Suppl. material 1: ops. 11, 16, 27, 31, 33 (AM). 3 specimens. Suppl. material 1: ops. 9, 16, 40 (NHMUK).

#### Family Dorvilleidae Chamberlin, 1919

H. Wiklund

Dorvilleids contain some of the smallest described annelids and are the only extant group with ctenognath jaws. The family Dorvilleidae consists of ~ 200 species arranged in 32 genera (Read and Fauchald 2020), with more than a third of the genera containing only one species. The most speciose dorvilleid genus is *Ophryotrocha* Claparède & Mecznikow, 1869 with ~ 75 described species. The first *Ophryotrocha* species were described from shallow water, but with advancing deep-sea sampling, now more species are known from the deep sea than shallow water. Worms in the genus are opportunistic feeders and thrive in organically enriched habitats both in deep-sea and shallow waters, for example in polluted harbours, sewer outlets, beneath fish farms and on hydrothermal vents, cold seeps and whale falls. One species of *Ophryotrocha* has been described from Australian waters, *O. shieldsi* Paxton & Davey, 2010, that was found in large numbers beneath a shallow fish farm in Tasmania. In this study we report eight species of *Ophryotrocha*. Some species were far more abundant than others, with *Ophryotrocha* sp. 2 and *Ophryotrocha* sp. 3 being the most common. Several of these species are likely new to science.

**Remarks.** Species were preliminary separated on the basis of the forms of mandibles, shape of head and appendages, and shape of chaetae. The species vary slightly in size, with the smallest species being just 1 mm long (*Ophryotrocha* sp. 2) and the largest being 3.2 mm long (*Ophryotrocha* sp. 5).

##### Ophryotrocha

Taxon classificationAnimaliaPhyllodocidaDorvilleidae

sp. 1

Fig. 7A

###### Records.

55 specimens. Suppl. material 1: op. 100 (NHMUK).

**Figure 7.**
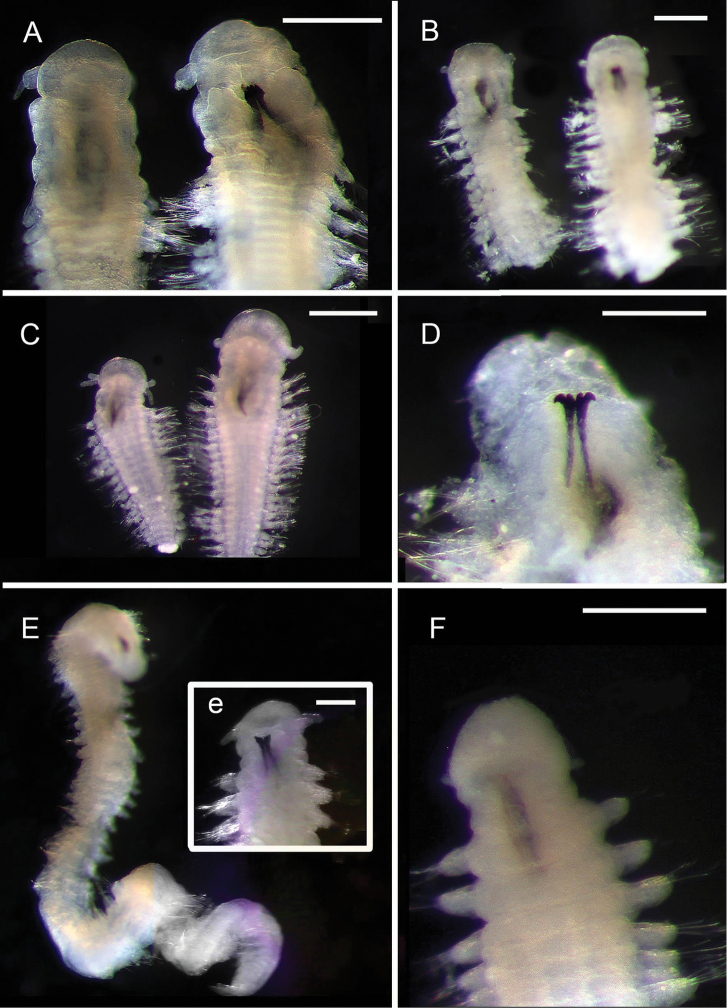
Dorvilleidae**A***Ophryotrocha* sp. 1 **B***Ophryotrocha* sp. 2 **C***Ophryotrocha* sp. 3 **D***Ophryotrocha* sp. 4 **E***Ophryotrocha* sp. 5 **e** same, anterior view **F***Ophryotrocha* sp. 6. Scale bars: 200 µm.

##### Ophryotrocha

Taxon classificationAnimaliaPhyllodocidaDorvilleidae

sp. 2

Fig. 7B

###### Records.

137 specimens. Suppl. material 1: op. 100 (NHMUK).

##### Ophryotrocha

Taxon classificationAnimaliaPhyllodocidaDorvilleidae

sp. 3

Fig. 7C

###### Records.

136 specimens. Suppl. material 1: op. 100 (NHMUK).

##### Ophryotrocha

Taxon classificationAnimaliaPhyllodocidaDorvilleidae

sp. 4

Fig. 7D

###### Records.

29 specimens. Suppl. material 1: op. 100 (NHMUK).

##### Ophryotrocha

Taxon classificationAnimaliaPhyllodocidaDorvilleidae

sp. 5

Fig. 7E, e

###### Records.

17 specimens. Suppl. material 1: op. 100 (NHMUK).

##### Ophryotrocha

Taxon classificationAnimaliaPhyllodocidaDorvilleidae

sp. 6

Fig. 7F

###### Records.

17 specimens. Suppl. material 1: op. 100 (NHMUK).

##### Ophryotrocha

Taxon classificationAnimaliaPhyllodocidaDorvilleidae

sp. 7

###### Records.

1 specimen. Suppl. material 1: op. 100 (NHMUK).

##### Ophryotrocha

Taxon classificationAnimaliaPhyllodocidaDorvilleidae

sp. 8

###### Records.

3 specimens. Suppl. material 1: op. 100 (NHMUK).

##### Dorvilleidae gen. spp.

Taxon classificationAnimaliaPhyllodocidaDorvilleidae

###### Remarks.

Specimens from whale fall (op. 100) were too damaged or dried out to be identified. A single specimen provisionally referred to *Schistomeringos* was recorded from 932 m in the GAB (MacIntosh et al. 2018: additional file 2). Further investigation is needed to understand if any of the GAB species match those found in this study.

###### Records.

75 specimens. Table 1: op. 100 (NHMUK).

#### Family Eunicidae Berthold, 1827

R. S. Wilson

Along with other members of the order Eunicida, species of Eunicidae possess a ventral muscular pharynx with mineralized or sclerotized jaws. Eunicidae are recognisable by possessing a prostomium with one to three antennae which lack ringed ceratophores. The family consists of 11 extant genera and 453 currently accepted species (Read and Fauchald 2020). They are not dominant members of abyssal benthic communities. Shallow water species from Australia are comparatively well known (Zanol et al. 2020) and historical records include about 32 species from six genera from Australian waters, all from <100 m water depth (http://www.ala.org.au). This study reports two species from depths of ~ 1000–2800 m off the east coast of Australia; neither species is previously known from Australia and both appear to be undescribed.

##### Eunice sp. nov.

Taxon classificationAnimaliaPhyllodocidaEunicidae

Fig. 8A, B

###### Diagnosis.

No pigmentation on preserved specimens. Prostomium bilobed, slightly notched. Eyes present, behind bases of palps. Prostomial appendages with widest gap separating palps from lateral antennae. Palpostyles, antennal styles, and peristomial cirri with irregular articulations. Peristomial rings distinct dorsally and ventrally but continuous laterally. Maxillae dentition: Mx I left 1, right 1. Mx II left 7, right 6. Mx III left 6. Mx IV left 5, right 9. Mx V left 1, right 1.

Branchiae absent. Lateral black dot between posterior parapodia absent. Dorsal cirri length short, at most as long as two body segments. Dorsal cirri of anterior chaetigers tapering, median chaetigers tapering, posterior chaetigers tapering, smooth, without articulations. Digitiform ventral cirri, basally inflated, commence chaetiger 3.

Pectinate chaetae absent. Compound falcigers present, appendages distally bidentate, hoods without mucros (rounded). Compound spinigers absent. Aciculae dark honey-coloured to black, distally bluntly pointed. Subacicular hooks dark honey-coloured to black, bidentate, distal tooth directed distally, subdistal tooth directed laterally. Subacicular hooks first present from chaetiger 24–27.

###### Remarks.

Although referred to here as ‘*Eunice* sp.’, the above combination of characters cannot be accommodated in any currently known eunicid genus. The species is here treated as a member of *Eunice* since that genus remains poorly defined and already contains species of uncertain relationships (Zanol et al. 2020).

###### Records.

4 specimens. Suppl. material 1: ops. 69, 104 (AM).

**Figure 8.**
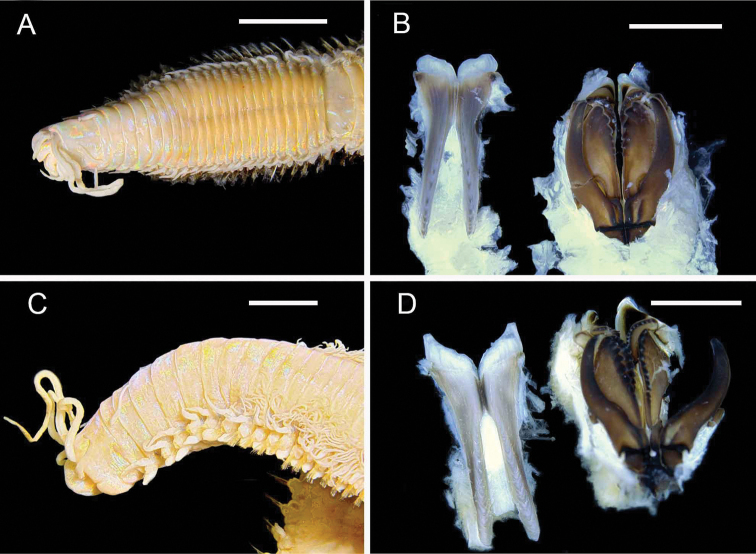
Eunicidae**A***Eunice* sp. nov., anterior view (AM W.50148) **B** Maxillae of *Eunice* sp. nov. (AM W.50148) **C***Leodice* sp. nov., anterior view (AM W.50146) **D** Maxillae of *Leodice* sp. nov. (AM W.50152). Scale bars: 2 mm (**A, C**); 1 mm (**B, D**).

##### Leodice sp. nov.

Taxon classificationAnimaliaPhyllodocidaEunicidae

Fig. 8C, D

###### Diagnosis.

Prostomial lobes frontally rounded, bilobed, slightly notched. Eyes behind bases of palps. Prostomial appendages evenly spaced, with palps slightly thinner than antennae. Antennal styles and palpostyles without articulations. Peristomial rings distinct dorsally and ventrally, continuous laterally. Peristomial (tentacular) cirri present, reach anterior region of peristomium, styles tapering, without articulations.

Maxillae dentition: Mx I left 1, right 1. Mx II left 7, right 9. Mx III left 12 (right absent). Mx IV left 6, right 11. Mx V left 1, right 1.

Branchiae present from chaetiger 4. Branchiae distinctly longer than dorsal cirri. Maximum number of branchial filaments 12–13 (at ~ chaetiger 10–12); five or six anterior chaetigers with single branchial filaments, no posterior chaetigers with single branchial filaments. Branchiae continuing until chaetiger 32–34. Ventral cirri of anterior segments digitiform.

Compound falcigers present, appendages bidentate, hoods without mucros (rounded). Aciculae light yellow, translucent. Subacicular hooks colour light yellow, translucent, bidentate.

###### Variation.

A number of very small specimens (< ~ 1.5 mm maximum width) differ from the above description in having at most three or four branchial filaments, and a single specimen much larger than the remaining material has < 18 branchial filaments. These specimens apparently do not differ in other respects and all are assumed to represent a single species.

###### Remarks.

This species clearly belongs in the genus *Leodice* following the generic concept of Zanol et al. (2020), but cannot be referred to any of the species known from Australia.

###### Records.

28 specimens. Suppl. material 1: ops. 13, 22, 44, 69, 70, 86, 100, 104, 115 (MV).

#### Family Euphrosinidae Williams, 1852

D. Ramos

Euphrosinidae have short, wide bodies bearing many chaetae. There are ~ 62 species in four genera now considered valid (Read and Fauchald 2020). It has been suggested that they are better represented in deep waters than the closely related Amphinomidae (Fauchald and Jumars 1979). Deep-water Euphrosinidae have been recorded off the east coast of Japan at ~ 400–450 m water depth (Imajima 2009) and around Antarctic regions (Kudenov 1993). Two species, *Euphrosine longesetosa* Horst, 1903 and *Euphrosine superba* Marenzeller, 1879, have been recorded from Australian waters (*Euphrosine longesetosa* from 9 m to 33 m, NSW and QLD; *Euphrosine superba* from shallow waters unknown depth, NSW) (http://www.ala.org.au). In this study we report one species.

##### Euphrosinopsis cf. horsti

Taxon classificationAnimaliaPhyllodocidaEuphrosinidae

Kudenov, 1993

###### Diagnosis.

Narrow prostomium, flanked by first three chaetigers, with a broader ventral pad. Pair of large eyes dorsally. Oval body dorsoventrally flattened. Abundant chaetae densely covering the dorsum. Notochaetae in five tiers: first and fifth tiers with small furcate chaetae, second and fourth tiers with large furcate chaetae, and third tier with category IIB ringent chaetae. Neurochaetae furcate. Paired inflated anal cirri with unfused bases present.

###### Remarks.

Observed specimens differ from described *Euphrosinopsis* species in having five tiers of notochaetae instead of two in *E. antarctica*, three in *E. crassiseta*, and four in *E. horsti* (Kudenov 1993). The category IIB ringent chaetae are similar to those of *E. antarctica* and *E. horsti* but with a smooth shaft. This is the first record of *Euphrosinopsis* outside the Southern Ocean.

###### Records.

3 specimens. Suppl. material 1: ops. 76, 98, 110 (NHMUK).

##### Euphrosinidae

Taxon classificationAnimaliaPhyllodocidaEuphrosinidae

gen. sp.

###### Records.

1 specimen. op. 9 (NHMUK).

#### Family Fabriciidae Rioja, 1923

A. Murray

Fabriciids are small (0.85–10 mm long) fanworms. Approximately 80 species in 17 valid genera are now considered to be in the family Fabriciidae (Read and Fauchald 2020), which until 2008 had been classified as a subfamily of Sabellidae (see Kupriyanova and Rouse 2008; Capa et al. 2010). Most species are intertidal or shallow-dwelling in fresh- or marine waters; however, there are a few genera such as *Raficiba*, *Pseudofabriciola*, and *Fabriciola*, which are represented in deep waters up to 100 m, with *Raficiba* known from continental shelf waters of ~ 300 m (Fitzhugh 1996; Fitzhugh 2001; Huang et al. 2011). A few undescribed species assigned provisionally to *Novafabricia* and *Fabriciola* have also been reported in southwest Atlantic Ocean deepsea basins at depths of 5000 m (Bick 2020). In Australian waters, the family has only been recorded from intertidal or subtidal depths (Hartmann-Schröder 1981, 1986, 1991; Hutchings et al. 1981; Fitzhugh 1990, 1992, 2002; Huang et al. 2011). In this study we report at least one species.

##### Fabriciidae

Taxon classificationAnimaliaPhyllodocidaFabriciidae

indet.

###### Diagnosis.

One small complete specimen, ~ 2 mm long. Branchial crown with three pairs of radioles with long pinnules terminating at same height as radioles. Eight thoracic and three abdominal chaetigers. Peristomial collar low, membranous, entire ventrally, mid-dorsally incised, anterior margin with shallow lateral notches (ventral conical flap or lobe absent). Dorsal lips as low rounded structures, ventral filamentous appendages absent. Conical structure above mouth absent. Peristomial glandular patches present. All thoracic notochaetae of two lengths: superior elongate, narrowly hooded; inferior short, narrowly hooded. Notochaetae of segments 3–8 similar to those of 1 and 2, pseudospatulate notochaetae absent. Thoracic uncini acicular with few rows of similar-sized teeth above main fang, hood present. Abdominal uncini with rasp-shaped teeth and long manubrium. Pigmented pygidial eyespots absent.

###### Remarks.

This single specimen has been preserved in 95% ethanol and may have lost pigmentation of the peristomial and pygidial eyes. There are many similarities with the genus *Fabriciola* but the absence of ventral filamentous appendages is not typical for that genus, so the identification is tentative. More complete and well-preserved specimens would be required to provide a positive identification. This specimen also does not match the diagnosis for *Pseudofabriciola*, with which it is also similar. This specimen is also similar to that found in the deep GAB surveys of 2015 and 2017, recorded as ‘? *Fabriciola* sp.’ (MacIntosh et al. 2018: additional file 2).

###### Records.

1 specimen. Suppl. material 1: op. 54 (AM).

##### Fabriciidae

Taxon classificationAnimaliaPhyllodocidaFabriciidae

gen. sp.

###### Records.

1 specimen. Suppl. material 1: op. 89 (AM).

#### Family Fauveliopsidae Hartman, 1971

A. Murray, D. Ramos

Fauveliopsids may be cylindrical or have swellings along the body, they can be free-living or occupy gastropod shells, foraminifera tests, or tubes (Salazar-Vallejo et al. 2019b). The family Fauveliopsidae currently consists of 27 species in three genera (Read and Fauchald 2020). Members of family Fauveliopsidae have mostly been described from deep waters < 6835 m (Salazar-Vallejo et al. 2019b). There are recent reports of this family in Australia (MacIntosh et al. 2018; Salazar-Vallejo et al. 2019b), with only one species, *Fauveliopsis challengeriae* McIntosh, 1922, currently described midway between Australia and Antarctica in 3510 m depth (Salazar-Vallejo et al. 2019b). In this study we report at least three species, one likely new to science.

##### Fauveliopsis cf. challengeriae

Taxon classificationAnimaliaPhyllodocidaFauveliopsidae

McIntosh, 1922

###### Diagnosis.

Specimens complete, 14.5 mm long, 1.5 mm wide at widest point. Body integument rugose and opaque, with scattered small papillae, tapered, posteriorly swollen, with segments in posterior region short (2–4 × wider than long), and 33 chaetigers. Anterior chaetigers with 2–4 chaetae per ramus, capillary and acicular (sigmoid or falcate) chaetae; middle and posterior chaetigers with 2–3 chaetae per notopodium and 3–5 chaetae per neuropodium, including falcate acicular chaetae and capillary chaetae. Interramal papillae distinct, somewhat stalked. Genital papillae not seen. Living in cemented sediment foraminifera tubes.

###### Remarks.

The type locality for this species is in the Southern Ocean between Antarctica and Australia in 3510 m depth. It has not previously been recorded in Australian waters, but was recently redescribed from specimens from Antarctic waters and Eastern Pacific Ocean (Salazar-Vallejo et al. 2019b) as the holotype is badly damaged. These specimens from off the east coast of Australia differ from those earlier descriptions in number and types of chaetae along the body. Also, because genital papillae were not observed, the identification remains tentative but may represent a new species.

###### Records.

3 specimens. Suppl. material 1: op. 43 (AM).

##### Laubieriopsis hartmanae

Taxon classificationAnimaliaPhyllodocidaFauveliopsidae

(Levenstein, 1970)

Fig. 9A–C

###### Diagnosis.

Prostomium retracted. Body linear, blunt on both ends with 16 chaetigers. Chaetigers 1–4 shorter with 2–3 large acicular chaetae and 2–3 small acicular chaetae per parapodia. Chaetigers 5–16 with one acicular and one capillary per ramus, longest on chaetiger 16. Granular genital papillae on boundary of chaetigers 6 and 7.

###### Remarks.

Similar in appearance to *L. brevis* from the Atlantic Ocean, but differs in the tips of the aciculars (bidentate in *L. brevis*) and genital papilla (smooth in *L. brevis*) (Salazar-Vallejo et al. 2019b). One species of *Laubieriopsis* was recorded from five stations (932–4068 m) in the GAB (MacIntosh et al. 2018: additional file 2); however, further investigation is required to determine if the species in the present study are the same as at the GAB.

###### Records.

22 specimens. Suppl. material 1: ops. 9, 16, 23, 40, 42, 45, 76, 79, 96, 110, 134 (NHMUK). 2 specimens. Suppl. material 1: op. 79.

**Figure 9.**
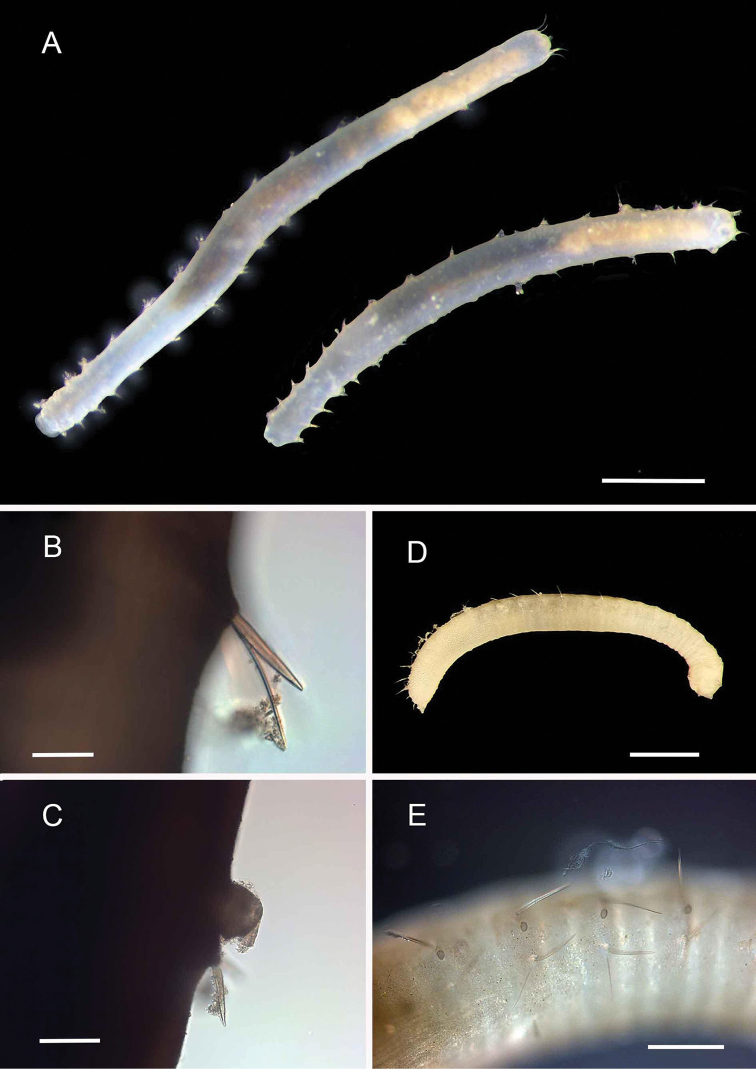
Fauveliopsidae**A***Laubieriopsis hartmanae***B***L. hartmanae*, chaetae **C***L. hartmanae*, genital papilla **D**Riseriopsis cf. santosae**E**Riseriopsis cf. santosae, chaetae. Scale bars: 1 mm (**A, D**); 50 µm (**B**); 100 µm (**C**); 250 µm (**E**).

##### Riseriopsis cf. santosae

Taxon classificationAnimaliaPhyllodocidaFauveliopsidae

Salazar-Vallejo, Zhadan & Rizzo, 2019b

Fig. 9D, E

###### Diagnosis.

Prostomium retracted. Body linear with annulations, slightly inflated terminally with 27 chaetigers. Chaetigers 1–3 shorter, parapodia with one acicular and one capillary per ramus and an interramal papillae. Following chaetigers with one acicular and one capillary in the neuropodia and one larger acicular in the notopodia.

###### Remarks.

*Riseriopsis santosae* resembles this specimen but differs in having more chaetigers (37–88). *Riseriopsis santosae* is known from shallower depths (410–415 m), while the only described deep-sea species in the genus, *R. confusa* (Thiel, Purschke & Böggemann, 2011), has a similar number of chaetigers as this specimen, but with an acicular and capillary in the medial notopodia. Both of these species are recorded from the South Atlantic.

###### Rec﻿ords.

1 specimen. Suppl. material 1: op. 96 (NHMUK).

##### Fauveliopsidae gen. spp.

Taxon classificationAnimaliaPhyllodocidaFauveliopsidae

###### Remarks.

Brenke sledge samples identified to family level.

###### Records.

60 specimens. Suppl. material 1: ops. 16, 23, 31, 33, 40, 42, 54, 79, 110, 134 (AM).

#### Family Flabelligeridae de Saint-Joseph, 1894

N. Jimi

The family Flabelligeridae is a group of sedentary annelids living in soft sediments or on hard substrates, except for two pelagic genera (Salazar-Vallejo and Zhadan 2007; Salazar-Vallejo 2008). The family is composed of 24 accepted genera and > 200 species (Salazar-Vallejo 2017). Flabelligerids are found from intertidal to abyssal depths world-wide but some genera (e.g., *Ilyphagus*) are restricted to abyssal depths. Approximately 20 flabelligerid species have been described or recorded from Australia (Haswell 1886, 1892; Day and Hutchings 1979; Salazar-Vallejo 2011a, b, 2012a, b, c; Salazar-Vallejo and Buzhinskaja 2011). However, all these records are from shallow waters (< 40 m) except for *Flabelligera affinis* reported from deep sea (101–500 m) by Day and Hutchings (1979), and thus, flabelligerid fauna of deep Australian waters is unknown. In this study, we report nine species of Flabelligeridae from the lower bathyal and abyssal Australian waters, four species are new to science.

##### Bradabyssa cf. kirkegaardi

Taxon classificationAnimaliaPhyllodocidaFlabelligeridae

Salazar-Vallejo, 2017

Fig. 10A

###### Diagnosis.

Length 3 mm, width 0.5 mm, body papillae very long, thin, abundant. Cephalic cage not developed. One capillary notochaeta, one anchylosed neurochaeta. Sediment particles present on base of papillae.

###### Remarks.

Salazar-Vallejo (2017) indicates this species contained some cryptic species because of the extensive bathymetric pattern of the species and for this reason we prefer to use the term Bradabyssa cf. kirkegaardi.

###### Records.

1 specimen. Suppl. material 1: op. 42 (AM).

**Figure 10.**
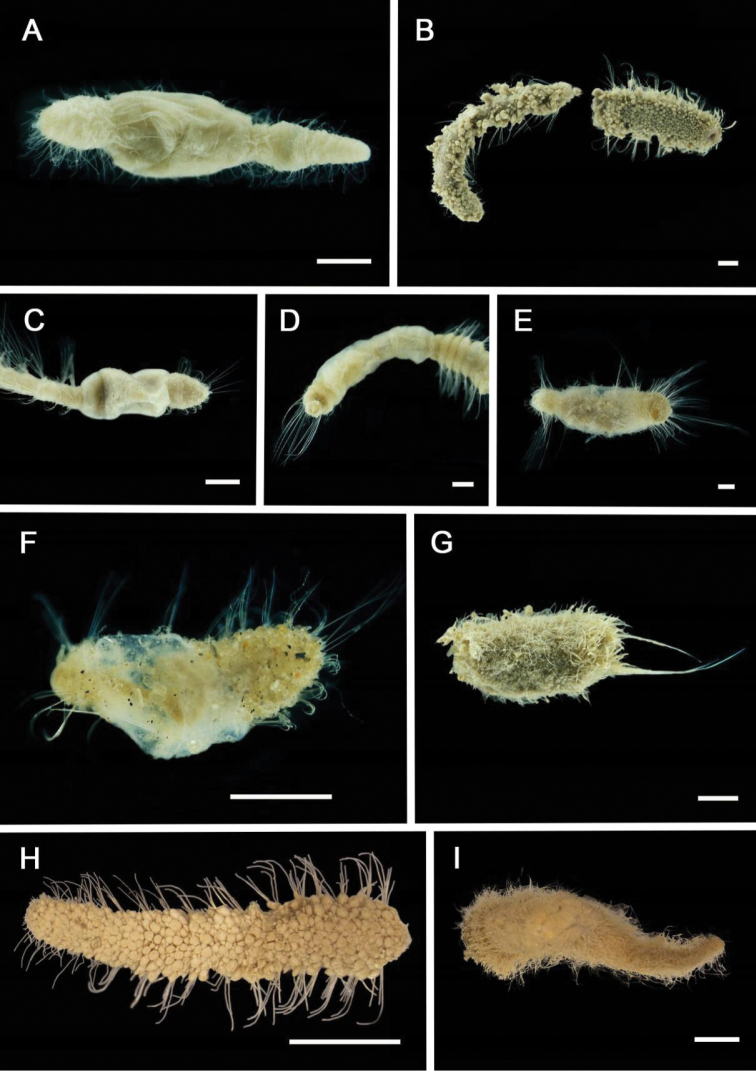
Flabelligeridae**A***Bradabyssa cf. kirkegaardi* (AM W.52573) **B***Bradabyssa* sp.1 (AM W.52551) **C***Diplocirrus* sp. nov. 1 (AM W.52559) **D***Diplocirrus* sp. nov. 2 (AM W.52563) **E***Diplocirrus* sp. nov. 4 (AM W.52551) **F***Diplocirrus* sp. 5 (AM W.52562) **G***Ilyphagus* sp. (AM W.52561) **H**Flabelligeridae sp. (AM W.52554) **I**Flabelligeridae sp. Scale bars: 500 µm (**A, B, D, E**); 1 mm (**C, F, G, I**); 2 mm (**H**).

##### Bradabyssa

Taxon classificationAnimaliaPhyllodocidaFlabelligeridae

sp. 1

Fig. 10B

###### Diagnosis.

Incomplete, length 9 mm, width 1 mm, 26 chaetigers, body papillae long, thin, abundant, with mud. Cephalic cage not developed. 2–4 capillary notochaetae, 3–4 anchylosed neurochaetae. Sediment particles present on body surface.

###### Remarks.

This species belongs to group ‘villosa’ described in Salazar-Vallejo (2017).

###### Records.

2 specimens. Suppl. material 1: ops. 16, 31 (AM).

##### Bradabyssa

Taxon classificationAnimaliaPhyllodocidaFlabelligeridae

sp. 2

###### Diagnosis.

Incomplete, length 25 mm, width 3 mm, 28 chaetigers, body tubercles short, without sediment particles, abundant. Cephalic cage not developed. 4–5 capillary notochaetae, 3–4 anchylosed neurochaetae. Sediment particles present on body surface.

###### Remarks.

This species belongs to group ‘verrucosa’ described in Salazar-Vallejo (2017).

###### Records.

1 specimen. Suppl. material 1: op. 16 (AM).

##### Diplocirrus

Taxon classificationAnimaliaPhyllodocidaFlabelligeridae

sp. nov. 1

Fig. 10C

###### Diagnosis.

Incomplete, length 5.4 mm, width 1 mm, 16 chaetigers. Body with first ten chaetigers swollen, thereafter cylindrical. Cephalic cage (chaetiger 1) developed. Lateral papillae and chaetae well developed in posterior chaetigers. Sand particles restricted on body wall. Body papillae few, short, thin.

###### Records.

7 specimens. Suppl. material 1: ops. 9, 31, 33 (AM).

##### Diplocirrus

Taxon classificationAnimaliaPhyllodocidaFlabelligeridae

sp. nov. 2

Fig. 10D

###### Diagnosis.

Incomplete, length ~ 5–8 mm, width 1 mm, ~ 17 chaetigers. Body with first nine chaetigers swollen, thereafter cylindrical. Cephalic cage (chaetigers 1 and 2) developed. Lateral papillae and chaetae well developed in posterior chaetigers. Sand particles on body wall, absent on body papillae. Body papillae abundant, short, thin.

###### Records.

2 specimens. Suppl. material 1: op. 45 (AM).

##### Diplocirrus

Taxon classificationAnimaliaPhyllodocidaFlabelligeridae

sp. nov. 3

###### Diagnosis.

Incomplete. Length 4 mm, width 0.5 mm, ~ 16 chaetigers. Body with first eight chaetigers swollen, thereafter cylindrical. Cephalic cage (chaetigers 1–3) developed. Lateral papillae not developed in posterior chaetigers, chaetae developed in posterior chaetigers. Attached sand particles absent. Body papillae scarce, very short, thin.

###### Records.

2 specimens. Suppl. material 1: op. 96 (AM).

##### Diplocirrus

Taxon classificationAnimaliaPhyllodocidaFlabelligeridae

sp. nov. 4

Fig. 10E

###### Diagnosis.

Incomplete. Length 5 mm, width 1.5 mm, ~ 13 chaetigers. Body with first eight chaetigers swollen, thereafter cylindrical. Cephalic cage (chaetigers 1–4) developed. Lateral papillae not developed in posterior chaetigers, chaetae developed along entire body. Sand particles present on body surface. Body papillae abundant, very short, thin, without sand particles.

###### Records.

5 specimens. Suppl. material 1: ops. 9, 16, 31, 87, 110 (AM).

##### Diplocirrus

Taxon classificationAnimaliaPhyllodocidaFlabelligeridae

sp. 5

Fig. 10F

###### Diagnosis.

Incomplete, only anterior fragments. Cephalic cage (chaetiger 1) developed. Large sand particles present on body surface. Body papillae few, short, thin, with large sediment particles on the base.

###### Records.

3 specimens. Suppl. material 1: op. 42 (AM).

##### Ilyphagus

Taxon classificationAnimaliaPhyllodocidaFlabelligeridae

sp.

Fig. 10G

###### Diagnosis.

Incomplete, damaged. Only anterior chaetigers. Body papillae very long, thin, abundant, with sediment particles at base of papillae. Cephalic cage developed. 1–2 capillary notochaetae, 4–5 anchylosed neurochaetae.

###### Records.

4 specimens. Suppl. material 1: ops. 40, 45, 55 (AM).

##### Flabelligeridae gen. spp.

Taxon classificationAnimaliaPhyllodocidaFlabelligeridae

Fig. 10H, I

###### Remarks.

Specimens were too damaged to identify further and Brenke sledge samples were identified only to family level.

###### Records.

1 specimen. Suppl. material 1: 16 (AM). 28 specimens. Suppl. material 1 ops. 9, 16, 31, 33, 40, 42, 45, 66, 96, 98, 103, 110, 119, 134 (NHMUK).

#### Family Glyceridae Grube, 1850

M. Böggemann, R. Sobczyk

Cylindrical and long-bodied worms, widely distributed in soft bottom sediments from intertidal zone to abyssal depths (Wilson 2000; Böggemann 2002). Glyceridae and Goniadidae constitute the Glycerimorpha group. Glycerids may be easily recognized by having annulated prostomium with two pairs of terminal appendages and four cross-arranged jaws on anterior end of eversible pharynx. The family consist of 46 valid species grouped in three genera (Böggemann 2014a). Sixteen species from three genera have been found in intertidal to upper abyssal zones of Australian waters (Hartman 1964; Day and Hutchings 1979; Hutchings and Murray 1984; Böggemann 2002; Böggemann and Wilson 2003; Böggemann 2015). This study reports at least two glycerid species, one may be new to science.

##### Glycera cf. capitata

Taxon classificationAnimaliaPhyllodocidaGlyceridae

Örsted, 1842

Fig. 11C

###### Diagnosis.

**Figure 11.**
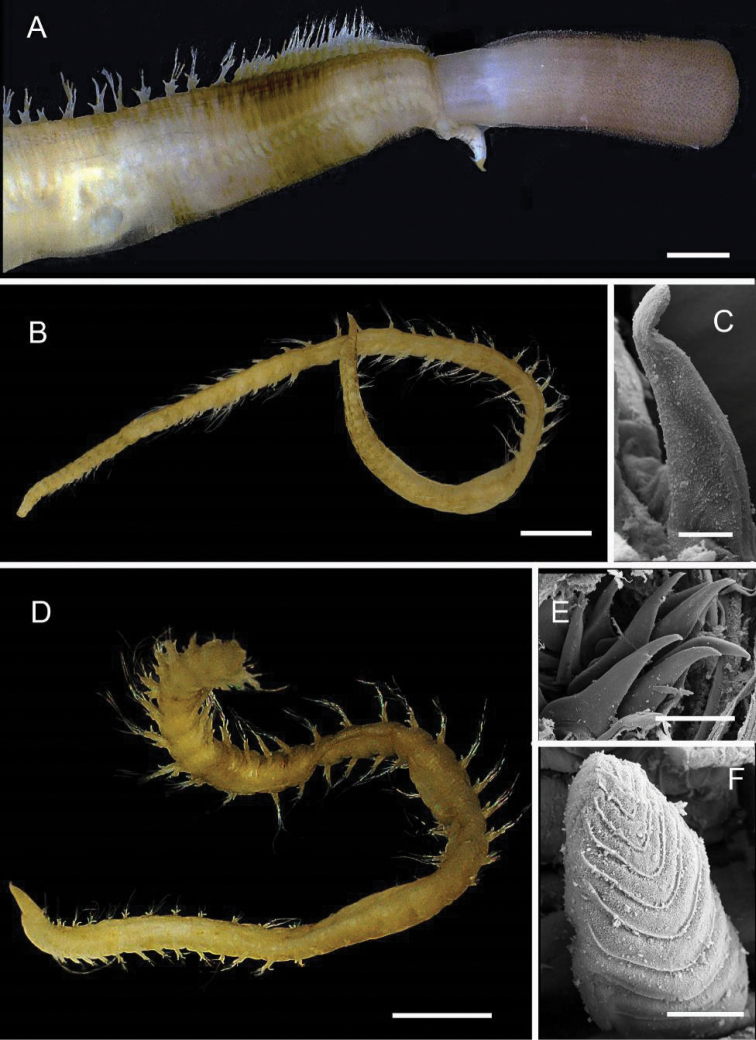
Glyceridae and Goniadidae. **A**Glyceridae, *Glycera* sp. anterior body with everted pharynx, ventrolateral view (AM W.52668) **B**Goniadidae, *Bathyglycinde profunda*, whole specimen (AM W.52667) **C**Glyceridae, Glycera cf. capitata, digitiform proboscideal papilla, lateral view (AM W.52672) **D**Goniadidae, *Bathyglycinde profunda*, anterior fragment (AM W.52667) **E***Bathyglycinde profunda*, proboscideal papillae of area II, posterior view (AM W.52669) **F**Glyceridae, Glycera cf. russa, conical proboscideal papilla with eight ridges, posterior view (AM W.52605). Scale bars: 1 mm (**A, B, D**); 10 µm (**C**); 50 µm (**E**); 10 µm (**F**).

Specimens < 7 mm long, 1.2 mm wide. Prostomium with ~ ten rings. Parapodia of mid-body with longer neuropodial than notopodial prechaetal lobes and one rounded postchaetal lobe, dorsal cirri inserted on body wall far above parapodial base. Proboscideal papillae of two types, long digitiform (Fig. 11C) and shorter oval to globular ones. Jaw ailerons with pointed triangular bases. Branchiae absent.

###### Remarks.

The identification to species level is tentative because the type locality of *Glycera capitata* is in the Atlantic Ocean off Greenland and there are no molecular data.

###### Records.

4 specimens. Suppl. material 1: ops. 79, 96, 98 (AM).

##### Glycera cf. russa

Taxon classificationAnimaliaPhyllodocidaGlyceridae

Grube, 1870

Fig. 11F

###### Diagnosis.

Specimen 74 mm long, 6 mm wide. Prostomium with only nine rings. Postchaetal lobes of mid-parapodia both triangular and similar in length along most of the body, posteriorly more acute. Proboscideal papillae of two types, long conical ones (Fig. 11F) and shorter oval ones, both with 6–10 transverse U-shaped ridges (and V-shaped apically). Jaw ailerons with triangular bases, not pointed nor deeply incised. Branchiae completely absent along body.

###### Remarks.

This specimen most resembles *Glycera russa* Grube, 1870. However, the prostomium consists of nine rings and the proboscideal papillae have only up to ten ridges, therefore, this may be a new species.

###### Records.

1 specimen. Suppl. material 1: op. 22 (AM).

##### Glycera spp.

Taxon classificationAnimaliaPhyllodocidaGlyceridae

Fig. 11A

###### Diagnosis.

Specimens with various lengths of the body and number of segments. Specimens dried up or missing the anterior parts of body. Prostomium when present prolonged, distinctly annulated with two pairs of appendages situated on anterior margin; eyes absent. Cylindrical proboscis, if present, covered by papillae; terminal part with four ailerons. Parapodia biramous.

###### Records.

4 specimens. Suppl. material 1: op. 31 (AM).

##### Glyceridae gen. spp.

Taxon classificationAnimaliaPhyllodocidaGlyceridae

###### Remarks.

Brenke sledge samples were identified to family level.

In addition to the taxa recorded from this study, *Glycera lapidum* Quatrefages, 1866 (13 stations, 212–2063 m) and *Glycerella magellanica* (McIntosh, 1885) (a single specimen, 2503 m) were recorded from the GAB (MacIntosh et al. 2018: additional file 2). However, we are unable to confirm if these are the same species until we compare material from both localities.

###### Records.

17 specimens. Suppl. material 1: ops. 9, 16, 31, 33, 79, 98, 110 (NHMUK).

#### Family Goniadidae Kinberg, 1865

M. Böggemann, R. Sobczyk

As the sister group to glycerids, goniadids are cylindrical, long-bodied annelids. The family is easily distinguished from glycerids by presence of usually one pair of macrognaths and variable number of ventral and dorsal micrognaths instead of two pairs of jaws on the anterior end of pharynx (Böggemann 2005). In addition, some genera have longitudinally arranged rows of V-shaped chevrons on each side of the proboscis. The family consist of 64 valid species in eight genera, two of which are monotypic (Böggemann 2014b). Goniadids are widely distributed from intertidal to abyssal depths (Böggemann 2005, 2009). In Australia, 22 species from six genera have been recorded (Day and Hutchings 1979; Walker-Smith and Wilson 2003; Böggemann 2005, 2015). In this study we report one named goniadid.

##### Bathyglycinde profunda

Taxon classificationAnimaliaPhyllodocidaGoniadidae

(Hartman & Fauchald, 1971)

Fig. 11B, D, E

###### Diagnosis.

Up to 24 mm length and 69 segments; anterior 36–37 parapodia uniramous; prostomium indistinctly annulated with bi-articulated terminal appendages; eyes absent; chevrons not present; papillae on pharynx arranged in rows (Fig. 11E); parapodia biramous at least at posterior part of body; notochaetae capillaries; one posterior neuropodial lobe.

###### Remarks.

A Depth range of 350–5500 m has been reported (Böggemann 2005).This species was previously recorded from a single station, 2063 m, in the GAB (MacIntosh et al. 2018: additional file 2). *Bathyglycinde profunda* was originally described from the equatorial region off northeast South America (4825 m) (Hartman and Fauchald 1971); however, molecular evidence suggests it is a widespread deep-water species (Böggemann 2009).

###### Records.

7 specimens. Suppl. material 1: ops. 16, 31, 33, 54 (AM). 3 specimens. Suppl. material 1: ops. 31, 103, 134 (NHMUK).

##### Goniadidae gen. spp.

Taxon classificationAnimaliaPhyllodocidaGoniadidae

###### Remarks.

Brenke sledge samples were identified to family level. *Goniada antipoda* (a single specimen, 2366 m), *Progoniada regularis* (a single specimen, 1486 m) and *Progoniada* sp. MoV7077 (5 stations, 932–4068 m) were recorded from the GAB (MacIntosh et al. 2018, additional file 2). Further investigation is required to determine if the species in the present study are the same as at the GAB.

###### Records.

1 specimen. Suppl. material 1: op. 76 (NHMUK).

#### Family Hesionidae Grube, 1850

C.J. Glasby, D. Ramos

Hesionids are a reasonably common and widespread group of polychaetes that have affinities with other nereidiforms, especially nereidids and syllids (Worsaae et al. 2005). There are currently 34 genera and ~ 450 valid species (Read and Fauchald 2020). They have been collected worldwide, from intertidal habitats to the deep sea. In Australian waters 13 genera and 21 species have been reported (http://www.ala.org.au). In this study we have treated *Microphthalmus* as a member of the family, as the change proposed by Salazar-Vallejo et al. (2019a) in elevating its subfamily Microphthalminae to family status has yet to be fully considered by the polychaete community and adopted in WoRMS (Read and Fauchald 2020) (Appendix I). We report at least five genera and six species, four species new to science, denoted using a species number.

##### Microphthalmus

Taxon classificationAnimaliaPhyllodocidaHesionidae

sp.

Fig. 12A–C

###### Diagnosis.

Prostomium round, anteriorly cleft, broader than long, with three antennae and two palps. Antennae twice the length of the palps. No eyes. Six pairs of cirriform tentacular cirri on segments 1–3, longer than dorsal cirri. Uniramous parapodia. Dorsal cirri shorter on segment 4 than those on segment 5 onwards. Neuropodia with a pointed prechaetal lobe longer than the blunt postchaetal lobe. Neurochaetae heterogomph falcigers with serrated edge. Pygidium with two anal cirri and a ventral anal plate. Colour in ethanol pale yellow.

###### Records.

10 specimens. Suppl. material 1: op. 100 (NHMUK). 50 specimens. Suppl. material 1: op. 100 (AM).

**Figure 12.**
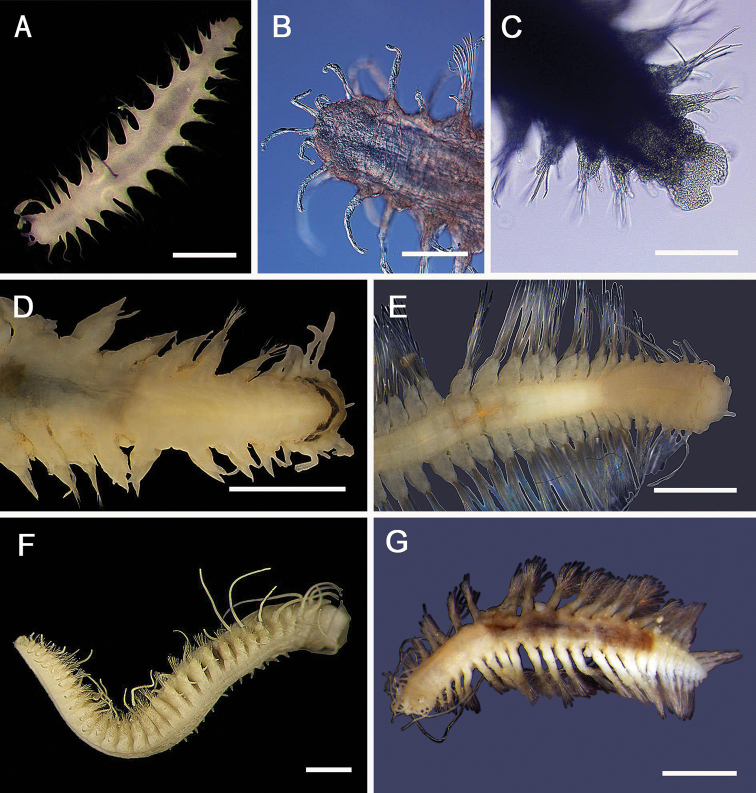
Hesionidae**A***Microphthalmus* sp. **B***Microphthalmus* sp., anterior end **C***Microphthalmus* sp., pygidium **D***Neogyptis* sp. nov. 1 (AM W.52327) **E***Neogyptis* sp. nov. 2 (AM W.52436) **F***Vrijenhoekia ketea***G** cf. *Vrijenhoekia* sp. nov. 1 (AM W.52453). Scale bars: 0.25 mm (**A**); 100 µm (**B**); 100 µm (**C**); 1 mm (**E, F, G**); 2 mm (**D**).

##### Neogyptis

Taxon classificationAnimaliaPhyllodocidaHesionidae

sp. nov. 1

Fig. 12D

###### Diagnosis.

Specimens all incomplete. Prostomium subrectangular (broader than long), posteriorly with deep mid-dorsal incision; one pair lateral antennae, tips well defined, extending two thirds to just short of length of palps; one short mid-prostomial antenna. Eyespots not visible. Palps bi-articulate, palpostyle cylindrical, twice length palpophore. Proboscis with many cylindrical papillae two or three rows deep, each papilla cilia-tipped. Nuchal organs very conspicuous, light brown, extending from anterolateral prostomium and coalescing mid-posterodorsally. First four segments tentacular, achaetous, bearing eight pairs tentacular cirri, longest extending back to chaetigers 5 or 6. Parapodia biramous throughout, dorsal cirri slender tapered, ~ two thirds length parapodial lobes anteriorly, half length in mid body; ventral cirri slender distally attached, tapered, extending less than half length parapodial lobes. Notopodia bearing capillaries only. Neuropodia compound spinigers. Colour in ethanol yellow-white base, with dark brown band dorsally and laterally on all tentacular segments, and lighter brown nuchal organs.

###### Remarks.

The present material clearly falls within the concept of the tribe Amphidurini Pleijel, Rouse, Sundkvist & Nygren, 2012, which includes *Amphiduros* Hartman, 1959, *Amphiduropsis* Pleijel, 2001, *Neogyptis* Pleijel, Rouse, Sundkvist & Nygren, 2012, and questionably *Parahesione* Pettibone, 1956. Of these three genera the present specimens are closest to *Neogyptis* because of the terminal ring of proboscideal papillae and presence of a median antenna. The fine-tipped lateral cirri and cilia-tipped cylindrical papillae were clearly visible only in the formalin-fixed specimen (AM W.52351, see Suppl. material 1) and not in the ethanol-fixed material, indicating the value of this preservative for morphological studies.

Of the 12 species in the genus the only species currently known from the West Pacific is *Neogyptis hinehina* Pleijel, Rouse, Sundkvist & Nygren, 2012 from the Lau Basin, south of Tonga. However, this species has twisted noto- and neurochaetae, which were not observed in the present material. Therefore, the present material probably represents an undescribed species.

###### Records.

11 specimens. Suppl. material 1: ops. 23, 31, 45, 54, 55, 76, 79 (AM).

##### Neogyptis

Taxon classificationAnimaliaPhyllodocidaHesionidae

sp. nov. 2

Fig. 12E

###### Diagnosis.

Specimens incomplete. Prostomium subrectangular (broader than long), posteriorly with slight mid-dorsal incision; antennae, presence implied from scars as follows: two lateral and one median antenna situated mid-posteriorly. Eyespots not visible. Palps bi-articulate, palpostyle cylindrical to conical, approximately equal in length to palpophore. Everted proboscis with many cushion-shaped distal papillae. First four segments tentacular, achaetous, bearing eight pairs tentacular cirri, slightly shorter than dorsal cirri of chaetiger 1. Nuchal organs, unpigmented, coalescing mid-dorsally. Parapodia biramous throughout, dorsal cirri slender tapered, ~ equal in length to parapodial lobes, except those on first chaetiger which are many times longer than parapodial lobe; ventral cirri distally attached, slender tapered, ~ half-length parapodial lobes throughout. Notopodia bearing serrated capillaries and one or two smooth spines. Neurochaetae all compound spinigers. Colour in ethanol yellow-white, unpigmented.

###### Remarks.

See above for discussion justifying placement of this material into *Neogyptis*, tribe Amphidurini Pleijel, Rouse, Sundkvist & Nygren, 2012, and reasons for considering it represents an undescribed species. *Neogyptis* sp. 2 differs from *Neogyptis* sp. 1 in having palpostyles approximately equal in length to the palpophore, and lacking brown pigmentation on tentacular segments and the nuchal organs.

###### Records.

2 specimens. Suppl. material 1: ops. 16, 110 (AM).

##### Parahesiocaeca

Taxon classificationAnimaliaPhyllodocidaHesionidae

sp. 1

###### Diagnosis.

Specimens in poor condition, incomplete. Prostomium sub-rectangular, with three antennae, all tapering rapidly and broader at base, extending just beyond tip of palps. Bi-articulated palps, palpostyle cylindrical. Eyes absent. Proboscis with marginal papillae. First two segments tentacular, achaetous, bearing four pairs of tentacular cirri (inferred from stubs). Tentacular and dorsal cirri appearing to be articulated. Parapodia sub-biramous. Neuropodia with long prechaetal lobe, short postchaetal lobe, and short ventral cirrus; all neurochaetae with long-bladed heterogomph falcigers with very fine tips.

###### Remarks.

The present specimens fit the description of the genus *Parahesiocaeca* Uchida, 2004, although it differs from the description of the only species, *P. japonica* Uchida, 2004, in lacking eyes, having brown-pigmented nuchal organs, and the heterogomph falcigers having very long blades with very fine tips. The specimens probably represent an undescribed species, but their condition is too poor to describe as a new species.

###### Records.

2 specimens. Suppl. material 1: op 96 (AM).

##### Pleijelius cf. longae

Taxon classificationAnimaliaPhyllodocidaHesionidae

Salazar-Vallejo & Orensanz, 2006

###### Diagnosis.

Prostomium ovoid, broader than long, with three short antennae and two short palps. No eyes. Six pairs of cirriform tentacular cirri on segments 1–3, longer than dorsal cirri. Dorsal cirri from segment 4 onwards multiarticulate, longer than body width. Notopodia low mounds with notochaetae spread out in a fan-like arrangement dorsally. Neuropodia elongated, with straight acicula and long acicular lobes. Neurochaetae heterogomph falcigers with serrated edge. Ventral cirri cirriform, much smaller than dorsal cirri. Three cirriform anal cirri on pygidium. Colour in ethanol white.

###### Remarks.

We use the cf. designation here in recognition that *Pleijelius longae*, the only representative of the genus and originally described from the Northwestern Atlantic Ocean at 3500 m, is unlikely to be the same species as ours. *Pleijelius* is represented by a single species, *P. longae* Salazar-Vallejo & Orensanz, 2006. Observed specimens differ from *P. longae* in having notochaetal capillaries with denticles along the entire length instead of just near the tip, as well as possessing three anal cirri instead of six. This is the first report of this genus in the Pacific Ocean, occurring on whale fall, and at a depth of 1000 m.

###### Records.

2 specimens. Suppl. material 1: op. 100 (NHMUK).

##### Vrijenhoekia ketea

Taxon classificationAnimaliaPhyllodocidaHesionidae

Summers, Pleijel & Rouse, 2015 species complex

Fig. 12F

###### Diagnosis.

Prostomium rectangular, with two lateral antennae, a very small median antenna, two palps, and a facial tubercle. Palpophores thicker than palpostyles, similar lengths. No eyes. Everted proboscis lacking papilla. Three fused anterior segments. Parapodia uniramous. Dorsal cirri long, especially in segments 1–5. Ventral cirri the same length as neuropodia after segments 1–3, digitiform, and inserted subterminally. Colour in ethanol pale yellow.

###### Remarks.

The specimen differs from other specimens of the *V. ketea* species complex in having a larger body, being closer to the size range observed for *V. balaenophila* (Pleijel et al. 2008).The *Vrijenhoekia ketea* species complex includes *V. ketea* Summers, Pleijel & Rouse, 2015, *V. falenothiras* Summers, Pleijel & Rouse, 2015 and *V. ahabi* Summers, Pleijel & Rouse, 2015, all from Monterey Canyon off California; they can only be separated based on molecular data (Summers et al. 2015).

###### Records.

1 specimen. Suppl. material 1: op. 100 (NHMUK).

##### cf. Vrijenhoekia

Taxon classificationAnimaliaPhyllodocidaLacydoniidae

sp. nov. 1

Fig. 12G

###### Diagnosis.

Two complete specimens, 22 chaetigers (i.e., appears to be fixed growth of maximum 22 chaetigers). Prostomium subrectangular (broader than long), one pair lateral antennae, long, tapered, extending to tip of palps or 2 × longer; median antenna absent. Small red eyespots present, two or three pairs. Palps bi-articulate, palpostyle oval to globulose, slightly longer than palpophore. Proboscis with ten digitate terminal papillae and micro-papillae on surface; jaws absent. Facial tubercle absent. First three segments tentacular, achaetous, bearing six pairs tentacular cirri, slender, longest almost half length of body. Parapodia uniramous throughout, bearing digitate prechaetal lobe; dorsal and ventral cirri slender, tapered, similar in length throughout: ~ 0.5–1 × length of parapodial lobes, except for first and second pairs which are many times longer than parapodial lobe (similar in length to tentacular cirri). Ventral cirri inserted subterminally. Fan-like supra- and sub-neuropodial fascicles, bearing compound spinigers only, all with similar-length blades. Colour in ethanol yellow-white, unpigmented.

###### Remarks.

The present specimens are closer to *Vrijenhoekia* than any other described hesionid genus, but differ from its type species, *Vrijenhoekia balaenophila*, and from V. cf. ketea as described above, as follows: a mediodorsal prostomial process (tubercle or antenna) was not observed (present in other members of the genus though minute and probably only observable clearly with scanning electron microscopy); palpostyles are globulose in the present material vs. tapered; compound chaetae blades are relatively longer in the present material; and the present specimens appear to have a maximum of 22 chaetigers, whereas there are 35 in the type species (this character is not reported in other species of the genus). On the other hand, the present material resembles more closely the type species than V. cf. ketea in having ten digitate proboscideal papillae (absent in the latter). The globulose (= ovoid) palpostyles are the most distinctive feature of the species.

###### Records.

13 specimens. Suppl. material 1: ops. 11, 23, 31, 40, 42, 45, 46, 54, 96 (AM).

##### Hesionidae gen. spp.

Taxon classificationAnimaliaPhyllodocidaLacydoniidae

###### Remarks.

Twelve specimens of Hesionidae could not be identified beyond family because key features were lacking or the specimens were damaged (missing posterior segments, tentacles, antennae etc.).

Material provisionally referred to Leocrates cf. chinensis (four stations, 987–1402 m), *Hesiolyra* sp. (one specimen, 996 m), *Nereimyra* sp. (one specimen, 1256 m), and *Parahesione* sp. MoV6858 (three stations, 203–236 m) was recorded from the GAB (MacIntosh et al. 2018: additional file 2), further investigation is required to determine if any of these species are the same as from the present study.

###### Records.

11 specimens. Suppl. material 1: ops. 40, 42, 45, 54, 76, 79, 110, 134 (AM). 1 specimen. Suppl. material 1: op. 45 (NHMUK).

#### Family Lacydoniidae Bergström, 1914

A. Murray

Lacydoniidae are an uncommon, but widespread group of polychaetes that have affinities with phyllodocids (Rizzo and Magalhães 2019; Rouse and Fauchald 1997). Currently there are 12 described valid species in one genus, *Lacydonia* (Read and Fauchald 2020). They have been collected worldwide, from intertidal habitats to depths of 5600 m (Rouse and Pleijel 2001). Lacydoniids have previously been recorded from abyssal depths off the west coast of Africa (Böggemann 2009) and in the Campos Basin off Brazil in the South Atlantic Ocean (Rizzo et al. 2016). Although lacydoniids have been recorded from shallow water Antarctic locations (Ehlers 1913; Hartmann-Schröder 1993; Hartmann-Schröder and Rosenfeldt 1988), to date there have not yet been published reports from mainland Australia, with only one record of Lacydoniidae from shallow northern waters in the Timor Sea at 49–62 m depth, as ‘*Lacydonia* sp.’ (Przeslawski et al. 2018). However, other unreported lacydoniid specimens from shallow waters around Australian coasts (Western Australia, South Australia, Tasmania and Queensland) and deeper waters from Tasmanian seamounts are also held in the Australian Museum collections (Murray, pers. obs.). In this study we report one species.

##### Lacydonia cf. laureci

Taxon classificationAnimaliaPhyllodocidaLacydoniidae

Laubier, 1975

###### Diagnosis.

Specimen incomplete, ~ 2 mm wide excluding chaetae, 3 mm long for head plus 12 anterior segments. Body dorsoventrally flattened. Prostomium approximately as wide as long, somewhat indented anteriorly, with conspicuous lateral lobes present on posterior margin of prostomium, giving the appearance of a much wider than long prostomium. Eyes absent, median antenna missing. Pair of short digitiform to filiform lateral antennae located in slight incisions mid-prostomium; pair of similar-sized/shaped palps arising ventral to prostomial anterior margin. Faded pale brown pigment present on prostomium, dorsally and ventrally, and dorsally as transverse bands on tentacular segment and some other segments, and as spots on dorsal cirri and parapodia. Tentacular segment short, achaetous, with pair of ventrolateral cirri. Chaetigers 1–3 uniramous, with compound spinigerous chaetae, subsequent parapodia biramous, rami elongate and widely separated, with elongate supracicular lobes. Notochaetae simple capillary chaetae, finely spinulose distally; neurochaetae compound spinigers with heterogomph shaft-heads and long, finely spinulose blades. Dorsal cirri short, thick, digitiform, glandular, inserted basally on first three chaetigers, thereafter medially to distally on notopodia. Ventral cirri of similar size and shape, inserted distally on neuropodia. Posterior segments, pygidium and pygidial cirri, all missing and therefore unknown.

###### Remarks.

*Lacydonia laureci* Laubier, 1975 is the only currently described species that possesses conspicuous lateral lobes on the posterior margin of the prostomium. This specimen bears some similarity to *L. laureci*, because of these lobes, as well as the absence of eyes, but there are a few differences also apparent: Rizzo et al. (2016) report that *L. laureci* possesses capillary notochaetae that have coarse serrations on the distal part of the chaetae, but Böggemann (2009), however, describes fine serrations over the entire length of the notochaetae for that species, whereas this specimen from Australian waters appears to possess fine serrations only on the distal portion of the notochaetae. *L. laureci* has also been reported from several widely distributed locations in 1001–5497 m depths: the type locality, Matapan Trench, Mediterranean Sea (Laubier 1975); Angola, Cape and Guinea Basin, SE Atlantic Ocean (Böggemann 2009), and the Campos Basin off Brazil, South Atlantic Ocean (Rizzo et al. 2016). Because this specimen consists of an anterior end only, this can only be a tentative identification.

###### Records.

1 specimen (incomplete). Suppl. material 1: op. 110 (AM).

#### Family Lumbrineridae Schmarda, 1861

P. Borisova, N. Budaeva, D. Ramos

Lumbrineridae is a family of jaw-bearing annelids from the large monophyletic group Eunicida. Lumbrinerids have a simple external morphology, with uniform elongated body, simple uniramous parapodia, and conical prostomium lacking distinct appendages or eyes. No appendages are present on the peristomium consisting of two rings, and only few species have branchiae associated with parapodia. In contrast, the diversity of jaw morphology is remarkable, and morphology of maxillary plates is used as diagnostic characters at genus and species levels. The family comprises 19 genera and ~ 300 species and has world-wide distribution (Carrera-Parra 2006). Lumbrineridae are very common in deep waters being the fifth most diverse annelid family found below 2000 m (Paterson et al. 2009). Australian lumbrinerids are poorly studied with two shallow water species described and one presumably cosmopolitan species reported from the region (Hutchings and Murray 1984; Hartmann-Schröder 1987). In this study we report at least three species from three genera.

##### Cenogenus sp. nov.

Taxon classificationAnimaliaPhyllodocidaLumbrineridae

Fig. 13A, B, C

###### Diagnosis.

Body width: 1–2 mm. Prostomium conical, elongated, shorter than peristomium. Nuchal antenna not observed. All parapodia well developed, first four pairs of parapodia smaller than remaining parapodia. Parapodia with inconspicuous prechaetal and postchaetal lobes equal in size. Anterior parapodia with simple digitate elongate (length to width ratio near 2:3) branchia attached dorsally and posteriorly to parapodial lobes, in middle parapodia branchiae decrease in length becoming more rounded.

Anterior parapodia with long fragile limbate chaetae only, middle parapodia with limbate chaetae and simple multidentate hooded hooks with six to seven small teeth and long blade. All chaetae dark, black or dark brown in colour, becoming translucent near tip. Acicula dark, two in median parapodia.

Maxillary apparatus dark, stout, with four pairs of maxillae. Maxillary carriers shorter than MI. MI forceps-like with attachment lamellae, without connecting plates. MII as long as MI, with two large teeth. MIII unidentate, completely pigmented, dark in colour. MIV large, unidentate round-square plates, completely pigmented.

###### Remarks.

The specimen AM W.50137 (op. 33, Suppl. material 1) differs from others by being smaller (width 1 mm, other three specimens near 2 mm wide) and having asymmetrical MII with three teeth on left MII and two teeth on right MII.

###### Records.

4 specimens. Suppl. material 1: ops. 30, 33, 53 (AM).

**Figure 13.**
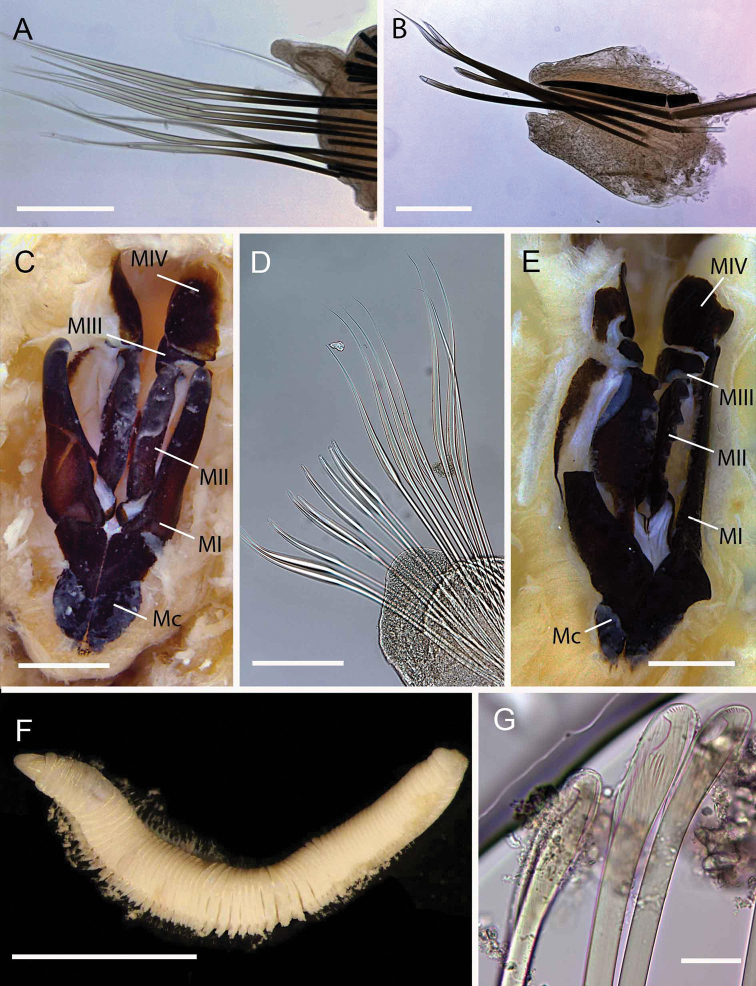
Lumbrineridae**A***Cenogenus* sp. nov. (AM W.50139), parapodium from chaetiger 7 **B***Cenogenus* sp. nov., posterior parapodium **C***Cenogenus* sp. nov., maxillae **D***Eranno* sp. nov., AM W.50140, parapodium from chaetiger 11 **E***Eranno* sp. nov., maxillae **F***Lumbrineris* sp., op. 4 (NHMUK), anterior part of the body, dorsal view **G***Lumbrineris* sp., simple hooded hooks. Abbreviations: Mc, maxillary carriers; MI–MIV, maxillary plates. Scale bars: 200 µm (**A, B, D**); 500 µm (**C, E**); 5 mm (**F**); 25 µm (**G**).

##### Eranno sp. nov.

Taxon classificationAnimaliaPhyllodocidaLumbrineridae

Fig. 13D, E

###### Diagnosis.

Prostomium conical, as long as wide, shorter than peristomium. Nuchal antenna absent. All parapodia well developed, first four to five pairs of parapodia smaller than remaining parapodia. Prechaetal lobes inconspicuous in all parapodia, postchaetal lobes auricular in anterior parapodia (1–40), becoming small and rounded posteriorly, always longer than prechaetal lobes.

Anterior parapodia with limbate chaetae and limbate simple hooded hooks. Clear simple multidentate hooded hooks present after chaetiger 25, with seven to eight teeth and long blades, all of similar size. In median chaetigers (27–40) part of limbate chaetae exceedingly longer than in the remaining chaetigers and ~ twice as long as hooded hooks.

Maxillary apparatus dark, with five pairs of maxillae, elongated. Maxillary carriers shorter than MI. MI forceps-like with attachment lamellae, with narrow connecting plates. MII shorter than MI, with five teeth. MIII unidentate, completely pigmented, dark in colour. MIV unidentate, large, completely pigmented. MV reduced to attachment lamella, partly fused with MIV.

###### Remarks.

Limbate simple hooded hooks are not typical for *Eranno* and similar to those reported for *Abyssoninoe*, however, maxillary apparatus is of typical *Eranno* shape with narrow connecting plates and MII significantly shorter than MI. There is not enough information to consider this a new genus, but we suggest it is a new species.

###### Records.

1 specimen. Suppl. material 1: op. 56 (AM).

##### Lumbrineris

Taxon classificationAnimaliaPhyllodocidaLumbrineridae

sp.

Fig. 13F, G

###### Diagnosis.

Bluntly conical prostomium without appendages. Two peristomial rings of similar sizes. Parapodia uniramous, neuropodia bearing yellow acicula, limbate chaetae compound (until chaetiger 13) and simple (chaetiger 14 onwards) hooded hooks with up to nine teeth. Maxillary apparatus: MII quadridentate, almost as long as MI; MIII and MIV unidentate. Colour in ethanol pale yellow.

###### Records.

1 specimen. op. 4 (NHMUK).

##### Lumbrineridae gen. spp.

Taxon classificationAnimaliaPhyllodocidaLumbrineridae

###### Remarks.

Brenke sledge samples were identified to family level.

###### Records.

5 specimens: Suppl. material 1: ops. 16, 31, 43 (AM). 4 specimens. Suppl. material 1: ops. 31, 54, 66, 79 (NHMUK).

#### Family Maldanidae Malmgren, 1867

J.A. Kongsrud

The family Maldanidae, commonly known as bamboo worms, are infaunal burrowers inhabiting tubes made of sediments consolidated by mucus. The family comprises ~ 240 valid species in 38 genera and five subfamilies (Read and Fauchald 2020). Maldanids are common members of deep-sea soft bottom communities, with ~ 50 species known from deeper than 2000 m depth (Paterson et al. 2009). The family is poorly studied in Australian waters with only ~ 20 species recorded, mostly from coastal areas (http://www.ala.org.au). In this study we report ten species from six genera, with four species likely new to science.

##### Boguea sp. nov.

Taxon classificationAnimaliaPhyllodocidaMaldanidae

Fig. 14A

###### Diagnosis.

Complete specimens with ~ 30 chaetigers, < 20 mm long and 0.2 mm wide. Head rounded without a cephalic plate. Cephalic keel well developed. Nuchal slits curved, parallel on each side of the cephalic keel. Neuropodia with avicular uncini present from chaetiger 5. Avicular uncini in single row in chaetiger 5–8, in double rows from chaetiger 9 and onwards. Pygidium simple, without papillae. Anus terminal. Tube cylindrical, straight, thick and solid, consisting of a thin inner organic layer incrusted with a thick layer of densely packed mud. Ventral glandular pads on anterior part of chaetigers 4–6 with reddish-brown pigmentation.

###### Remarks.

At present, only two species of *Boguea* have been described, *B. enigmatica* Hartman, 1945 from North Carolina, USA and *B. panwaensis* Meyer & Westheide, 1997 from Phuket, Thailand. This is the first record of the genus in the deep sea.

###### Records.

10 specimens. Suppl. material 1: ops. 44, 56 (AM).

**Figure 14.**
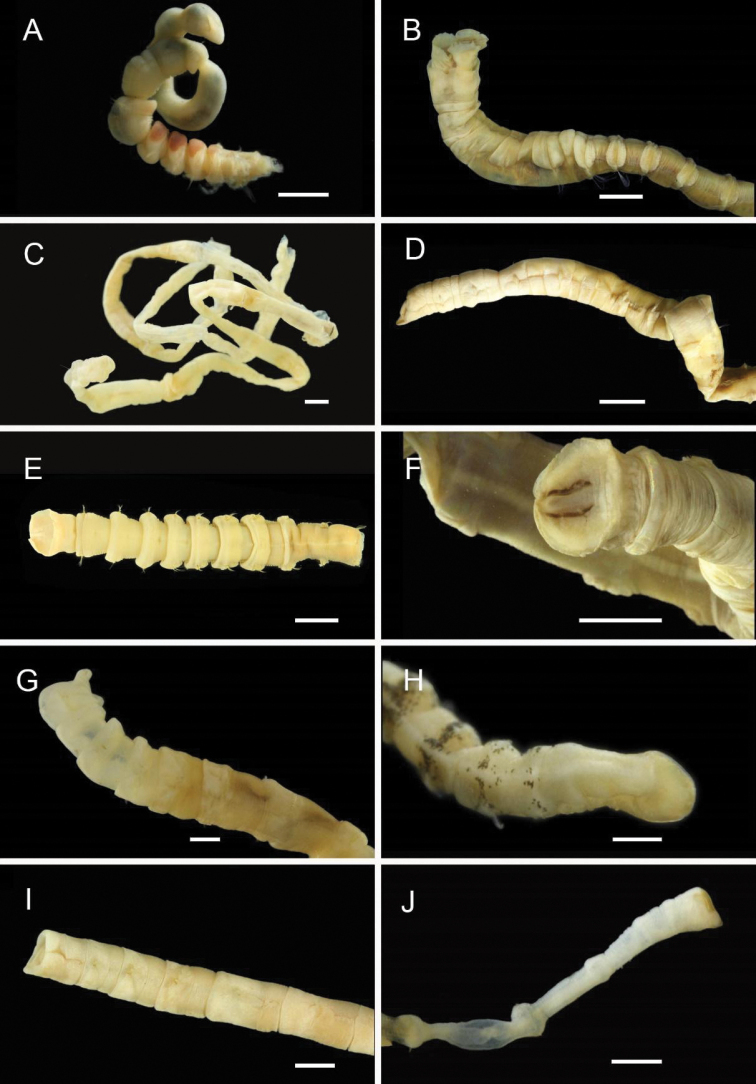
Maldanidae**A***Boguea* sp. nov. **B***Chirimia* sp. nov. **C***Lumbriclymene* sp. **D***Maldane* sp. **E***Maldanella* sp. 1 **F***Maldanella* sp. 2 **G***Notoproctus* sp. nov. 1 **H**Notoproctus cf. scutiferus**I***Notoproctus oculatus antarcticus***J***Notoproctus* sp. nov. 2. Scale bars: 5 mm (**A, D, F**); 1 mm (**B, C, G–J**).

##### Chirimia sp. nov.

Taxon classificationAnimaliaPhyllodocidaMaldanidae

Fig. 14B

###### Diagnosis.

Largest specimen available, anterior fragment with 14 chaetigers, 43 mm long and 3 mm wide. Head with cephalic plate bordered with well-developed rim. Cephalic rim divided in lateral and posterior lobes by deep lateral incisions; lateral lobes with five elongated, triangular cirri. Posterior lobe with eight triangular cirri. Nuchal slits long, U-shaped. No visible cephalic keel between nuchal slits. Palpode wide, rounded. Chaetiger 1 with distinct collar with deep lateral notches. Neurochaeta as rostrate hooks, starting on chaetiger 2. Posterior part of body and pygidium unknown. Tube unknown. Specimen in alcohol uniformly pale.

###### Remarks.

In general, this species is similar to *C. fauchaldi* Light, 1991, described from 2070 m depth in the East Pacific, off Panama, but differs in the development of the cephalic rim.

###### Records.

2 specimens. Suppl. material 1: ops. 80, 104 (AM).

##### Lumbriclymene

Taxon classificationAnimaliaPhyllodocidaMaldanidae

sp.

Fig. 14C

###### Diagnosis.

Incomplete, anterior fragment with 11 chaetigers, 90 mm long and 1.5 mm wide. Head well defined, rounded, ~ as long as wide, with distinct cephalic keel. Nuchal slits long and curved on each side of the cephalic keel. All chaetigers elongated: chaetiger 1 approximately as long as wide, chaetiger 2 and chaetiger 3 ~ 4 × longer than wide, chaetigers 4–11 ~ 6 × longer than wide. Anterior four chaetigers with a single straight acicular spine per neuropodium. Neuropodia of remaining chaetigers with single row of rostrate hooks. Posterior part of body and pygidium unknown. Tube unknown. Specimen in alcohol uniformly pale.

###### Records.

1 specimen. op. 70 (AM).

##### Maldane

Taxon classificationAnimaliaPhyllodocidaMaldanidae

sp.

Fig. 14D

###### Diagnosis.

Incomplete, anterior fragment with ten chaetigers, 50 mm long and 4 mm wide. Head with oval cephalic plate and a wide, rounded palpode. Cephalic rim divided in lateral and posterior lobes by distinct lateral incisions; posterior rim forming small pocket covering posterior part of the cephalic plate. Cephalic keel prominent; nuchal slits long and parallel, on each side of the cephalic keel. Anterior four chaetigers distinctly biannulate, with parapodia placed on anterior annulus. Epidermal glands distinct in anterior chaetigers. Neurochaetae as rostrate hooks, starting on chaetiger 2.

###### Records.

1 specimen. Suppl. material 1: op. 43 (AM).

##### Maldanella

Taxon classificationAnimaliaPhyllodocidaMaldanidae

sp. 1

Fig. 14E

###### Diagnosis.

Single complete specimen with 19 chaetigers and three preanal achaetous segments, 80 mm long and 4 mm wide. Additional numerous anterior fragments and a few posterior fragments available. Head with oval cephalic plate. Cephalic rim well developed with minute lateral incisions. Cephalic palpode very small, rounded. Cephalic keel not visible. Nuchal slits straight and parallel, not outward-curved anteriorly, on anterior one third of the cephalic plate. Neurochaetae as rostrate hooks, in single rows from chaetiger two onwards. Notochaetae as simple capillaries in two rows in all chaetigers. Posterior achaetous region with three achaetous segments with rudimentary parapodia, and a well-developed pygidial funnel. Posterior rim of anal funnel rimmed with triangular cirri. Anus on small cone inside anal funnel. Tube greyish, thin and flexible, loosely incrusted by fine sand particles. Specimens in alcohol uniformly pale.

###### Remarks.

Species of *Maldanella* (including *Abyssoclymene* as synonym) are common members of abyssal soft bottom fauna (McIntosh 1885; Detinova 1982; Kongsrud et al. 2013). However, the taxonomy of the genus is confused with several species being poorly characterized. The present material includes two species, here reported as *Maldanella* sp. 1 and *Maldanella* sp. 2 (see below). A single specimen, provisionally referred to the genus *Maldanella*, was recorded from 376 m depth in the GAB (MacIntosh et al. 2018: additional file 2).

###### Records.

107 specimens. Suppl. material 1: ops. 4, 6, 22, 35, 44, 56, 128 (AM).

##### Maldanella

Taxon classificationAnimaliaPhyllodocidaMaldanidae

sp. 2

Fig. 14F

###### Diagnosis.

Incomplete, anterior fragment with 14 chaetigers, 65 mm long and 5 mm wide. In general, similar to *Maldanella* sp.1, but differing in details of the head. The cephalic rim is comparatively low and the cephalic keel is distinct. Two pigmented lines run parallel on each side of the cephalic keel. Nuchal slits straight, parallel on each side of the cephalic keel. Palpode minute. Neurochaetae as rostrate hooks, in single rows starting on chaetiger 2. Notochaetae as simple capillaries in two rows. Tube not known. Colour in alcohol: body uniformly pale, with distinct pigmented parallel lines along cephalic keel.

###### Records.

2 specimens: Suppl. material 1: op. 32 (AM).

##### Notoproctus oculatus antarcticus

Taxon classificationAnimaliaPhyllodocidaMaldanidae

Arwidsson, 1911

Fig. 14I

###### Diagnosis.

Complete specimens with 19 chaetigers and two preanal achaetous segments, < 27 mm long and 1 mm wide. Cephalic plate with wide anterior palpode and bordered by a thickened low rim with distinct postero-lateral incisions; nuchal slits strongly curved, located centrally on cephalic plate, transversely oriented. No visible cephalic keel between nuchal organs. Ocelli not observed. Head and anterior four chaetigers biannulate. Anterior four chaetigers with single, straight acicular spine per neuropodium. Neuropodia of remaining chaetigers with single row of rostrate hooks. Notochaetae as simple capillaries. Anal plate slightly oval without distinct rim.

###### Records.

15 specimens. Suppl. material 1: ops. 4, 43, 44, 56 (AM).

##### Notoproctus

Taxon classificationAnimaliaPhyllodocidaMaldanidae

sp. nov. 1

Fig. 14G

###### Diagnosis.

Complete specimens with 19 chaetigers and three achaetous preanal segments, < 29 mm long and 1.5 mm wide. Head rounded, cephalic plate without distinct border. Anterior part of cephalic plate (palpode) distinctly set-off in an angle from the plate. Cephalic keel indistinct. Nuchal slits slightly curved, transversally oriented. Ocelli as numerous small reddish dots in two groups located antero-laterally on palpode. Anterior four chaetigers with single, straight acicular spine per neuropodium. Neuropodia of remaining chaetigers with single row of rostrate hooks. Anal plate more or less circular, slightly pointed dorsally, with thickened rim.

###### Records.

3 specimens. Suppl. material 1: op. 70 (AM).

##### Notoproctus cf. scutiferus

Taxon classificationAnimaliaPhyllodocidaMaldanidae

Wesenberg-Lund, 1948

Fig. 14H

###### Diagnosis.

Only two posterior fragments available, largest 26 mm long and 0.8 mm wide for 13 chaetigers and two achaetous preanal segments. Anal plate oval, without distinct rim. A characteristic quadrangular pad/ridge present ventrally on preanal chaetigers, slightly overhanging ventral part of anal plate. Tube relatively robust, incrusted with small stones and shell fragments. Brown/black pigmentation on tori.

###### Remarks.

The species is similar to *N. scutiferus* Wesenberg-Lund, 1948, described from abyssal depths in the NW Atlantic, in the presence of a quadrangular pad/ridge ventrally on preanal achaetigerous segments.

###### Records.

2 specimens. Suppl. material 1: op. 32 (AM).

##### Notoproctus

Taxon classificationAnimaliaPhyllodocidaMaldanidae

sp. nov. 2

Fig. 14J

###### Diagnosis.

Several anterior and posterior fragments present. Largest anterior fragment with ten chaetigers, 15 mm long and 0.4 mm wide. Cephalic plate more or less circular. Nuchal slits slightly curved, transversely oriented. No cephalic keel visible between nuchal slits. Ocelli absent. Anterior four chaetigers with one or two straight acicular spine(s) per neuropodium. Neuropodia of remaining chaetigers with a single row of rostrate hooks. Two preanal achaetigerous segments. Anal plate more or less circular with distinct lateral incisions. Anal opening dorsally to plate.

###### Remarks.

Presence of an anal plate with distinct lateral incisions, similar to what is seen in species of *Maldane*, is unique within the genus *Notoproctus*.

###### Records.

14 specimens, Suppl. material 1: op. 56 (AM).

##### Maldanidae gen. spp.

Taxon classificationAnimaliaPhyllodocidaMaldanidae

###### Remarks.

Brenke sledge samples were identified to family level or material was too damaged to identify further.

###### Records.

19 specimens. Suppl. material 1: ops. 31, 33, 42, 55, 66, 89, 110 (AM). 9 specimens. Suppl. material 1: ops. 4, 11, 33, 40, 42, 66 (NHMUK).

#### Family Melinnidae Chamberlin, 1919

T. Alvestad, L. M. Gunton

Melinnidae are tubicolous annelids that often have dorsal hooks. Recently the subfamily Melinninae Chamberlin, 1919 within Ampharetidae was raised to the family Melinnidae (Stiller et al. 2020). The family Melinnidae is composed of five accepted genera and 49 species (Read and Fauchald 2020). Solis-Weiss (1993) suggested that Melinnidae are generally restricted to deeper waters, this appears to only hold true for the genera *Melinnopsis* McIntosh, 1885 and *Melinantipoda* Hartman, 1967a of which all species are described from 50–5600 m. Prior to this study, only two species of Melinnidae were reported from Australian waters, *Isolda pulchella* Müller in Grube 1858 and *Isolda warnbroensis* Augener, 1914 (Day and Hutchings 1979). Here we report at least five species from two genera. Material from the present study was used to describe two deep-sea Melinnidae species *Melinnopsis chadwicki* Gunton, Kupriyanova & Alvestad 2020 and *Melinnopsis gardelli* Gunton, Kupriyanova & Alvestad 2020 , reported here.

##### Melinna cf. armandi

Taxon classificationAnimaliaPhyllodocidaMelinnidae

McIntosh, 1885

Fig. 4D, d

###### Diagnosis.

No complete specimens, large, robust worm > 30 mm length, 5 mm width. Abdominal segments very badly preserved and/or missing on all specimens. Body long, widest in postbranchial region. Thorax with 18 chaetigers; neurochaetae as small acicular spines on first four chaetigers and uncini on remaining 14 chaetigers. Prostomium with well-defined anterior and posterior parts, separated by a pair of deep transverse nuchal that almost meeting mid-dorsally. Anterior part distally trilobed. No eyespots. Many smooth buccal tentacles. Chaetiger 1 collar-like, laterally and ventrally encompassing head region; anterior margin not crenulated. Branchiae in two basally fused groups of four. Inner and anterior most branchia of each group only fused at base. Branchiae all long, circular in cross section, tapering evenly to narrow tips. Postbranchial hooks (Fig. 4d) with sharply pointed and gently curved tips. Dorsal end of neurochaetal row on chaetiger 1–3 on elevated lobe. Chaetiger 3 with a few notochaetal capillaries. Chaetiger 4 with small, but well developed notopodia. Serrated brim/fold behind the hooks (dorsal membrane) with ~ 14 equally sized lanceolate projections.

###### Remarks.

*Melinna armandi* was originally described from west of North Island, New Zealand. Specimens here have 14 lanceolate projections on transverse dorsal membrane whereas *M. armandi* have eight.

###### Records.

21 specimens. Suppl. material 1: ops. 4, 30 (AM).

##### Melinnopsis chadwicki

Taxon classificationAnimaliaPhyllodocidaMelinnidae

Gunton, Kupriyanova & Alvestad, 2020

###### Diagnosis.

Neurochaetae small acicular spines with lanceolate tips on segments 2–5. Neuropodial uncini from chaetiger 5 (segment VI), present in 12 thoracic uncinigers. Postbranchial dorsal membrane low inconspicuous, located on chaetiger 4. Branchiae emerging together on dorsal branchial ridge at level of segments II and III, arranged in two basally fused groups of four. Uncini of thoracic uncinigers with two teeth in one vertical row over rostral tooth, subrostral process and basal prow.

###### Remarks.

Type locality is eastern Australia at 1006–1257 m. For detailed description see *Melinnopsis chadwicki*Gunton et al. (2020).

###### Records.

27 specimens: Suppl. material 1: ops. 69, 80, 104, 121 (AM).

##### Melinnopsis gardelli

Taxon classificationAnimaliaPhyllodocidaMelinnidae

Gunton, Kupriyanova & Alvestad, 2020

Fig. 4F

###### Diagnosis.

Neurochaetae small acicular spines with lanceolate tips on segment II–V. Neuropodial uncini from chaetiger 5 (segment VI), present in 12 thoracic uncinigers. Postbranchial dorsal membrane low inconspicuous, located on chaetiger 4. Branchiae emerging together on dorsal branchial ridge at level of segments II–III, arranged in two basally fused groups of four. Conspicuous stained band immediately behind dorsal fold ending between chaetigers 9 and 10. Uncini of thoracic uncinigers with three teeth in one vertical row over rostral tooth, subrostral process and basal prow.

###### Remarks.

Type locality is eastern Australia at 2520–2821 m. For detailed description see *Melinnopsis gardelli*Gunton et al. (2020).

###### Records.

62 specimens. Suppl. material 1: ops. 4, 22, 44, 54, 56, 90, 101, 122 (AM).

##### Melinnopsis spp. nov.

Taxon classificationAnimaliaPhyllodocidaMelinnidae

Fig. 4G

###### Diagnosis.

Minute acicular chaetae present on segments II–V. One long buccal tentacle present, diagnostic of genus. Four pairs of branchiae. Colour in ethanol pale yellow. Many specimens, but usually in poor shape.

###### Remarks.

At least two species of *Melinnopsis* are present. Further molecular investigation is required to delineate species. Some specimens are near to *Melinnopsis tetradentata* (Imajima, 2001) described from 621–622 m depth in Tosa Bay, Japan, but further investigation of type material is required to confirm their identity.

###### Records.

112 specimens. Suppl. material 1: ops. 4, 6, 14, 22, 30, 43, 44, 56, 69, 80, 90, 101, 104, 121, 122 (AM).

##### Melinnidae gen. spp.

Taxon classificationAnimaliaPhyllodocidaMelinnidae

###### Remarks.

Specimens were incomplete which does not allow further identification.

###### Records.

2 specimens. Suppl. material 1: op. 128 (AM).

#### Family Nephtyidae Grube, 1850

A. Murray, D. Ramos

The family Nephtyidae is distinguished by the presence of an interramal branchia attached to the ventral notopodial margin and a single median pygidial cirrus. The family is composed of > 140 species in four genera (Read and Fauchald 2020). Nephtyids are most abundant in shallow sandy and muddy environments but can be found at all depths (Ravara et al. 2017a). *Nephtys* and *Aglaophamus* are the most diverse nephtyid genera globally and in Australia, and some species of *Micronephthys* and *Inermonephtys* have also been recorded in Australian waters (Dixon-Bridges et al. 2014). To date, 22 nephtyid species have been documented in Australia (Murray et al. 2015). Australian nephtyids have been recorded mostly from shallow waters and seem to have a high degree of endemicity (Dixon-Bridges et al. 2014), though several species have been reported in deep waters: *Aglaophamus profundus* Rainer & Hutchings, 1977 from Bass Strait in 2195 m (Paxton 1974; Rainer and Hutchings 1977) and also from the GAB in 3714 m (MacIntosh et al. 2018: additional file 2); *Nephtys paradoxa* Malm, 1874, from 860 m off Green Cape, NSW (Fauchald 1963); *Aglaophamus* MoV7086 (one specimen, 3465 m); and *Micronephthys* MoV6847 (4 stations, 203–1521 m) from the GAB (MacIntosh et al. 2018: additional file 2). In this study we report at least four nephtyid species.

##### Aglaophamus spp.

Taxon classificationAnimaliaPhyllodocidaNephtyidae

Fig. 2D, F

###### Diagnosis.

Prostomium rectangular with anteriorly-projecting lateral antennae and ventro-laterally-projecting palps. Nuchal glands present in posterior corners of prostomium. No eyes observed. Body tapering posteriorly. Parapodia of first chaetiger directed anteriorly, neuropodia projecting forward beside the prostomium while the notopodia shorter and without dorsal cirri. Following parapodia distinctly biramous, with dorsal and ventral cirri. Involute interramal cirri present from chaetiger 8 or 12–15, emerging from notopodia only. Preacicular chaetae barred (Fig. 2D), postacicular chaetae spinulose, neuropodial chaetae of chaetiger 1 simple capillaries.

###### Remarks.

Preliminary molecular work using 16S gene on these specimens has separated them into three clades, each clade restricted to either lower bathyal or abyssal depths (Ramos 2019). *Aglaophamus gippslandicus* Rainer & Hutchings, 1977 (six stations, 138–141 m), *A. profundus* Rainer & Hutchings, 1977 (one specimen, 3714 m), and *Aglaophamus* sp. MoV7086 (one specimen, 3465 m) were recorded from the GAB (MacIntosh et al. 2018: additional file 2). Further investigation is needed to confirm if any of these GAB species are the same as the ones from this study.

###### Records.

17 specimens. Suppl. material 1: ops. 5, 16, 31, 33, 40, 45, 54, 66, 76, 79, 110, 119 (NHMUK). 65 specimens. Suppl. material 1: ops. 16, 23, 31, 33, 40, 45, 46, 54, 55, 76, 134 (AM).

##### Micronephthys

Taxon classificationAnimaliaPhyllodocidaNephtyidae

sp. 1

###### Diagnosis.

Small bodied specimens, 4–8 mm long for < 30 chaetigers. Branchiae (or interramal cirri) absent. Prostomium with straight to slightly convex anterior margin, subpentagonal to round in shape; short, conical antennae inserted on distal margin slightly medial to anterolateral corners; palps single, short, conical, inserted and directed ventrally. Subdermal eyespots not visible. First chaetiger similar in size to following chaetigers, not reduced. Parapodial acicular lobes conical, aciculae with curved tips. Neuropodial superior lobes absent. Parapodial rami with four types of chaetae: barred chaetae present in preacicular position, spinose chaetae present in postacicular position; capillary chaetae present in neuropodia of chaetiger 1; and some finely spinulose (almost smooth) long capillary-like chaetae in postacicular position of notopodia on following chaetigers. Furcate (lyrate) chaetae absent; thick dentate chaetae absent from chaetiger 1. Some specimens with very long spinulose chaetae in mid and posterior chaetigers, so specimens perhaps in swimming phase. Small papilla-like dorsal cirrus present on inner posteroventral face of all notopodia. Dissected pharynx with pair of conical jaws, 20 bifid terminal papillae (none enlarged more than others), plus 20–22 longitudinal rows of subterminal papillae with more than eight long papillae per row, diminishing in size proximally, single elongate middorsal and midventral papillae absent.

###### Remarks.

These specimens agree with the emended diagnosis for the genus by Murray et al. (2015). There are currently 15 valid nominal species of *Micronephthys* (Read and Fauchald 2020), of which only two species possess the combination of absence of both branchiae and lyrate chaetae (see Dnestrovskaya and Jirkov 2010, 2019): *Micronephthys abranchiata* Ehlers, 1913 and *M. ambrizettana* Augener, 1918. These species, however, have fewer papillae in the subterminal rows of pharyngeal papillae (4–6, cf. > 8 for IN2017_V03 specimens), and the latter species also possesses eyespots which the specimens herein do not. Without examination of type material of all *Micronephthys* species, the identity of specimens described above must remain unknown. *Micronephthys* MoV6847 (four stations, 203–1521 m) were recorded from the GAB (MacIntosh et al. 2018: additional file 2) and further investigation is required to determine if the species from the GAB are the same as those from the present study.

###### Records.

71 specimens. Suppl. material 1: ops. 9, 16, 23, 27, 31, 33, 40, 54, 55, 79 (AM).

##### Nephtys cf. paradoxa

Taxon classificationAnimaliaPhyllodocidaNephtyidae

Malm, 1874

Fig. 2E

###### Diagnosis.

Body stout, wider anteriorly and tapering from middle chaetigers to posterior. Prostomium subrectangular, eyes not visible, pharynx when everted with ten pairs of terminal bifid papillae, and 22 rows of subterminal papillae (rows with 4–6 similar conical papillae) extending only one third of pharyngeal length, median dorsal and ventral papillae not elongated, proximal region smooth. Antennae and palps conical, short, nuchal organs conspicuous, rounded. Parapodia biramous with well-separated rami. Interramal branchiae present, somewhat recurved, from chaetiger 11, becoming foliaceous from ~ chaetiger 16 and appearing membranous from chaetiger 14–16, ciliated and fully developed with membranous expansions (or ‘foliaceous lamellae’) ~ chaetiger 20, becoming small and rudimentary from ~ chaetiger 38 to posterior chaetigers. Acicular lobes obliquely rounded, notopodia with rudimentary preacicular and low postacicular lobes; neuropodia with rudimentary preacicular lobe, postacicular lamella longer than acicular lobe. Chaetae short and ‘spiky’, of three kinds: barred chaetae in preacicular position, spinulose chaetae in postacicular position and capillary chaetae present in neuropodia of chaetiger 1.

###### Remarks.

This species has previously been recorded from off the east Australian coast by Paxton (1974), collected in 1912 from 860 m, and has also been collected from 200 m northeast of Coffs Harbour, NSW in 1993 (unpublished AM records). However, it is a deep-water species widely distributed in the Arctic, the northern Atlantic Ocean, the Mediterranean Sea and the Pacific Ocean (Ravara et al. 2010a), originally described from the North Sea. It is surmised that it may be a species complex and that examination of global material as well as molecular analyses can only resolve its taxonomic status (Ravara et al. 2010b). Therefore, this is a tentative identification.

###### Records.

2 specimens Suppl. material 1: ops. 80, 101 (AM).

##### Nephtyidae gen. spp.

Taxon classificationAnimaliaPhyllodocidaNephtyidae

###### Remarks.

Brenke sledge samples were identified to family level.

###### Records.

7 specimens. ops. 16, 31, 40, 54 (NHMUK).

#### Family Nereididae Blainville, 1818

D. Ramos, R. S. Wilson

Nereididae are commonly found in intertidal areas worldwide, as a result the family has been extensively studied and used by physiologists for laboratory experiments and as bait by fishermen. They possess an eversible pharynx with one pair of jaws and often have accessory papillae or denticles in a regular pattern. There are 48 currently accepted genera and ~ 708 extant marine species (Read and Fauchald 2020). Abyssal Nereididae faunas are typically dominated by species of *Ceratocephale* (e.g., see Hilbig 1997; Böggemann 2009). In Australian waters there are 100 species reported from 24 genera (http://www.ala.org.au). In this study we report at least eight species from four genera.

##### Ceratocephale

Taxon classificationAnimaliaPhyllodocidaNereididae

sp. 1

###### Diagnosis.

Eyes absent; tentacular cirri all very short, longest extending just beyond peristomium; cirrophore of dorsal cirri not significantly expanded; double ventral cirri from chaetiger 3, posterior one twice length of anterior one. Specimens too small to dissect to observe paragnath/papillae arrangement.

###### Remarks.

Specimens do not fit descriptions of any named species. Members of the genus are typically common in deep-sea samples, however, in this study they were rare.

###### Records.

3 specimens. Suppl. material 1: ops. 79, 89, 110 (AM).

##### Neanthes cf. bassi

Taxon classificationAnimaliaPhyllodocidaNereididae

Wilson, 1984

###### Diagnosis.

Area I = 0–4; II = 6–27; III = 1–14; IV = 1–18 conical paragnaths + 2–7 smooth bars; V = 0–1; VI = 2–16; VII–VIII = 5–30. Dorsal notopodial ligule similar size to acicular ligule throughout (not markedly reduced or expanded on posterior chaetigers). Prechaetal notopodial lobe absent. Neuropodial postchaetal lobe present on chaetigers 1– ~ 12. Ventral neuropodial ligule on posterior chaetigers reduced, up to half length of acicular neuropodial ligule. Notochaetae homogomph spinigers only (homogomph falcigers absent). Neuropodial dorsal fascicle fused falcigers absent.

###### Remarks.

Prior to the collection of the first abyssal samples in Australian waters, *Neanthes bassi* was only known from 0–147 m. The most similar species is *Neanthes tasmani* Bakken, 2002 (known from slightly deeper locations, 75–220 m). However, *Neanthes* specimens from this study and recent GAB voyages are very close to *N. bassi*, yet they have been recorded from 200–4800 m. This taxon is referred to here as Neanthes cf. bassi, a hypothesis to be tested when molecular data are available. Two species other than *N. tasmani* are similar enough to be confused with *N. bassi* (until the pharynx is dissected), *Neanthes kerguelensis* (which has fewer maxillary ring paragnaths and lacks oral ring paragnaths or has at most VI = 1 and VII–VIII = 8) and *Nicon maculata* (which is also described here and lacks paragnaths completely yet is otherwise strikingly similar to *N. kerguelensis*).

This species was also recorded from eight stations (199–4518 m) in the GAB (MacIntosh et al. 2018: additional file 2).

###### Records.

15 specimens. Suppl. material 1: ops. 15, 30, 32, 43, 65 (AM).

##### Neanthes cricognatha

Taxon classificationAnimaliaPhyllodocidaNereididae

(Ehlers, 1904)

Fig. 15A

###### Diagnosis.

Prostomium with entire anterior margin. Longest tentacular cirri extend back to chaetiger 4. Maxillary ring of pharynx without papillae. Area I = 9–16; II = 22–45; III = 23–45 paragnaths; IV = 29–54; V, VI, VII–VIII forming a dorsally and ventrally continuous ring.

Notopodial prechaetal lobe present, well developed, so that notopodium made up of two ligules and one lobe similar and triangular. Dorsal cirrus length ~ 1 × ventral notopodial ligule at chaetiger 10–20. Neuropodial postchaetal lobe present, at least on some anterior chaetigers. Ventral neuropodial ligule on posterior chaetigers similar to length of acicular neuropodial ligule. Ventral cirri single.

Notopodial homogomph spinigers present; sesquigomph spinigers absent. Notopodial homogomph falcigers absent. Neuropodial dorsal fascicle fused falcigers absent.

###### Remarks.

At 1194–1257 m, this is the deepest record of this species which is widely distributed in Australia and New Zealand from the intertidal to 253 m, suggesting this may be a species complex.

###### Records.

1 specimen. Suppl. material 1: op. 80 (AM).

**Figure 15.**
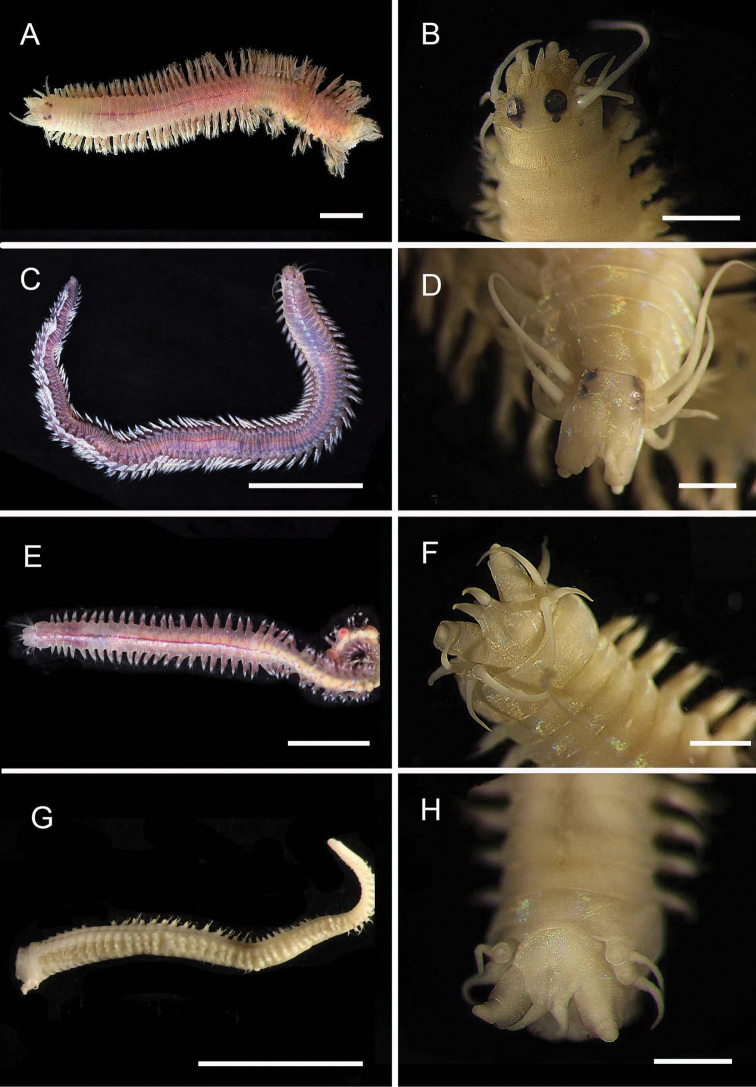
Nereididae**A***Neanthes cricognatha* (op. 80) **B***Neanthes heteroculata*, prostomium **C***Neanthes* sp. 1, live specimen **D***Neanthes* sp. 1, prostomium **E***Neanthes* sp. 2, live specimen **F***Neanthes* sp. 2, prostomium **G***Nereis* sp. 1 **H***Nereis* sp. 1, prostomium. Scale bars: 5 mm (**A**); 1 mm (**B, D, F**); 10 mm (**C, E**); 5 mm (**G**); 0.5 mm (**H**).

##### Neanthes heteroculata

Taxon classificationAnimaliaPhyllodocidaNereididae

(Hartmann-Schröder, 1981)

Fig. 15B

###### Diagnosis.

Prostomium slightly wider than long, one anterior pair of very large eyes and one very small pair posteriorly. Jaws with dentate cutting edge, translucent yellow to brown with six teeth. Maxillary ring of pharynx with paragnaths as follows: Area I absent; II absent; III absent; IV 1–3 conical paragnaths; V absent; VI 2 conical paragnaths; VII–VIII 7 conical paragnaths in a ventral band.

Longest tentacular cirri extend back to chaetiger 6. Dorsal notopodial ligule of posterior chaetigers similar to those on anterior chaetigers. Prechaetal notopodial lobe present; smaller than dorsal notopodial ligule on anterior chaetigers, reduced and ultimately absent posteriorly. Acicular process absent. Dorsal cirrus basally attached throughout, ~ 1 × acicular notopodial ligule at chaetiger 10–20. Neuropodial prechaetal and postchaetal lobes absent. Ventral neuropodial ligule of anterior chaetigers present, ~ as long as acicular neuropodial ligule throughout.

Notoaciculae absent from segments 1 and 2. Notopodial homogomph spinigers present. Neurochaetae: dorsal fascicle heterogomph spinigers absent. Neuropodial dorsal fascicle homogomph spinigers present. Neuropodial dorsal fascicle with heterogomph falcigers and homogomph falcigers on anterior chaetigers present. Neurochaetae, ventral fascicle: homogomph spinigers; ventral fascicle heterogomph falcigers.

###### Remarks.

Although previously only known from the type material in the North Atlantic, Bay of Biscay, 4700 m, the present material from 3980–4280 m is indistinguishable on morphological characters. This is the first record from Australia and the first record since the original description.

###### Records.

4 specimens. Suppl. material 1: ops. 54, 65, 78 (NHMUK).

##### Neanthes

Taxon classificationAnimaliaPhyllodocidaNereididae

sp. 1

Fig. 15C, D

###### Diagnosis.

Antennae ~ one quarter length of prostomium. Longest tentacular cirri extending back to chaetiger 5–6. Area I = 13; II ≥ 30; III ≥ 30; IV > 30; V absent; VI = 6; VII–VIII = 51. Prechaetal notopodial lobe present; approximately equal to length of dorsal notopodial ligule at least on anterior chaetigers (thus notopodium of three similar sized ligules/lobes); present throughout all chaetigers. Dorsal cirrus length ~ 1 × acicular notopodial ligule at chaetiger 10–20. Neuropodial prechaetal and postchaetal lobes absent. Neuropodial postchaetal lobe present, at least on some anterior chaetigers; projecting strongly beyond end of acicular ligule throughout all chaetigers. Ventral neuropodial ligule of anterior chaetigers ~ as long as acicular neuropodial ligule. Notopodia larger than neuropodia, dorsal notopodial ligule prominent triangular, largest structure in the parapodia.

Notopodial homogomph spinigers present. Neurochaetae: dorsal fascicle homogomph spinigers only (heterogomph falcigers absent). Neurochaetae, ventral fascicle: heterogomph spinigers absent. Homogomph spinigers present, heterogomph falcigers absent.

###### Records.

1 specimen. Suppl. material 1: op. 100 (NHMUK).

##### Neanthes

Taxon classificationAnimaliaPhyllodocidaNereididae

sp. 2

Fig. 15E, F

###### Diagnosis.

Eyes absent. Antennae ~ half length of prostomium. Longest tentacular cirri extend back to chaetiger 3 and 4.

Area I = 3; II = 16–17; III = > 30; IV = > 60; V absent; VI = 7–9; VII–VIII = > 120. Dorsal cirrus length ~ 1.5 × acicular notopodial ligule at chaetiger 10–20.

Neuropodial prechaetal lobe absent. Neuropodial postchaetal lobe absent. Ventral neuropodial ligule of anterior chaetigers ~ as long as acicular neuropodial ligule. Notopodia larger than neuropodia, dorsal notopodial ligule triangular, pointed.

Notopodial homogomph spinigers present. Neurochaetae: dorsal fascicle homogomph spinigers and heterogomph falcigers. Neurochaetae, ventral fascicle: heterogomph spinigers and heterogomph falcigers.

###### Records.

1 specimen: Suppl. material 1: op. 100 (NHMUK).

##### Nereis

Taxon classificationAnimaliaPhyllodocidaNereididae

sp. 1

Fig. 15G, H

###### Diagnosis.

Eyes absent. Paragnath counts: Area I = 2; II = 14–16; III = 0–3 (unclear, possibly damaged during dissection); IV = 9; V absent; VI = 6; VII–VIII = 30–40. Dorsal notopodial ligule markedly broader and elongate on posterior chaetigers. Prechaetal notopodial lobe absent. Dorsal cirrus length ~ twice acicular notopodial ligule at chaetiger 10–20. Dorsal cirrus on posterior chaetigers terminally attached to dorsal notopodial ligule. Neuropodial prechaetal and postchaetal lobes absent. Ventral neuropodial ligule ~ as long as acicular neuropodial ligule. Notopodial homogomph spinigers present. Notopodial homogomph falcigers present. Notopodial homogomph falciger blades very long. Notopodial homogomph falcigers multidentate, with two or more small lateral teeth, first and subsequent lateral teeth much smaller than terminal tooth. Neurochaetae: dorsal fascicle heterogomph spinigers absent. Neuropodial dorsal fascicle homogomph spinigers and heterogomph falcigers. Neurochaetae, ventral fascicle: heterogomph spinigers and heterogomph falcigers.

###### Records.

1 specimen. Suppl. material 1: op. 100 (NHMUK).

##### Nicon maculata

Taxon classificationAnimaliaPhyllodocidaNereididae

Kinberg, 1865

###### Diagnosis.

Longest tentacular cirri extending back to chaetiger 5–9. Maxillary and oral rings of pharynx entirely bare of papillae and paragnaths. Dorsal notopodial ligule similar size to acicular ligule throughout (not markedly reduced or expanded on posterior chaetigers). Prechaetal notopodial lobe absent. Neuropodial postchaetal lobe present on chaetigers 1– ~ 12. Ventral neuropodial ligule on posterior chaetigers reduced, up to half length of acicular neuropodial ligule. Notochaetae homogomph spinigers only (homogomph falcigers absent). Neuropodial dorsal fascicle fused falcigers absent.

###### Remarks.

Until the pharynx is dissected and found to be bare, this species is strikingly similar to *Neanthes bassi* and *N. kerguelensis* (McIntosh, 1885). Although *Nicon maculata* does have slightly longer tentacular cirri and the postchaetal neuropodial lobe (which is present in all three species) seems slightly longer and appears articulated or constricted in *Nicon maculata* where it meets the neuropodial lobe (see further comments for *N. bassi*, above). These taxa are otherwise very similar, and it is difficult to sustain the placement of *Nicon maculata* in a different genus as no such revision has yet been undertaken. Hutchings and Reid (1991) were the first to record *Nicon maculata* from Australia.

This record here at 2687–2821 m is the deepest record of this species that we are aware of. The species is also known from a single specimen (1391 m) in the GAB (MacIntosh et al. 2018: additional file 2) and widely recorded in southeastern Australia, the vicinity of Heard Island, and on the Antarctic continental margin at depths 145–1650 m.

###### Records.

1 specimen. Suppl. material 1: op. 44 (AM).

##### Nereididae gen. spp.

Taxon classificationAnimaliaPhyllodocidaNereididae

###### Remarks.

Specimens were too small to dissect to observe paragnath arrangement. Brenke sledge samples were identified to family level.

###### Records.

3 specimens. Suppl. material 1: ops. 33, 54, 128 (NHMUK).

#### Family Onuphidae Kinberg, 1865

H. Paxton, N. Budaeva

The family Onuphidae, a member of the jaw-bearing order Eunicida, consists of the subfamilies Onuphinae comprising 18 genera and Hyalinoeciinae Paxton, 1986 comprising five genera. Most onuphids are tubicolous; while the Onuphinae are sediment dwellers, well represented in intertidal to shelf depths, the Hyalinoeciinae are often found as epibenthic crawlers in deeper environments, making the onuphids the fourth most diverse polychaete family in the deep sea (Paterson et al. 2009). Onuphinae are much better studied than the Hyalinoeciinae as a result of their depth distribution and the scarcity of bathyal and abyssal sampling (Paxton 2000). In Australian waters there are 31 species reported from 13 genera (http://www.ala.org.au). The present study recovered 161 onuphid specimens, almost exclusively made up of hyalinoeciines. Ten species from four hyalinoeciine genera were identified, the most diverse being *Nothria* with five species. The only onuphine genus collected was *Paradiopatra*, with three species reported. A key to genera and their definitions can be found in Paxton (1986).

##### Anchinothria cf. pycnobranchiata

Taxon classificationAnimaliaPhyllodocidaOnuphidae

(McIntosh, 1885)

Fig. 16A

###### Diagnosis.

Peristomial cirri present. Parapodia 1 enlarged; parapodia 1–3 with bi- to trilobed prechaetal lobes; subulate ventral cirri on chaetiger 1 and 2. Bidentate simple to pseudocompound hooks on first three parapodia; pectinate chaetae scoop-shaped. Simple branchiae from chaetiger 16–19. Round tubes of inner parchment-like lining and outer muddy layer with embedded foreign objects such as spines or spicules.

###### Remarks.

The species is widely distributed in great depths of southern oceans, perhaps a species complex. It is new to Australian waters, also collected from six stations at the GAB (990–1790 m depth) reported as *Anchinothria* sp. 1 (MacIntosh et al. 2018: additional file 2).

###### Records.

6 specimens. Suppl. material 1: ops. 56, 69, 115, 122 (AM).

**Figure 16.**
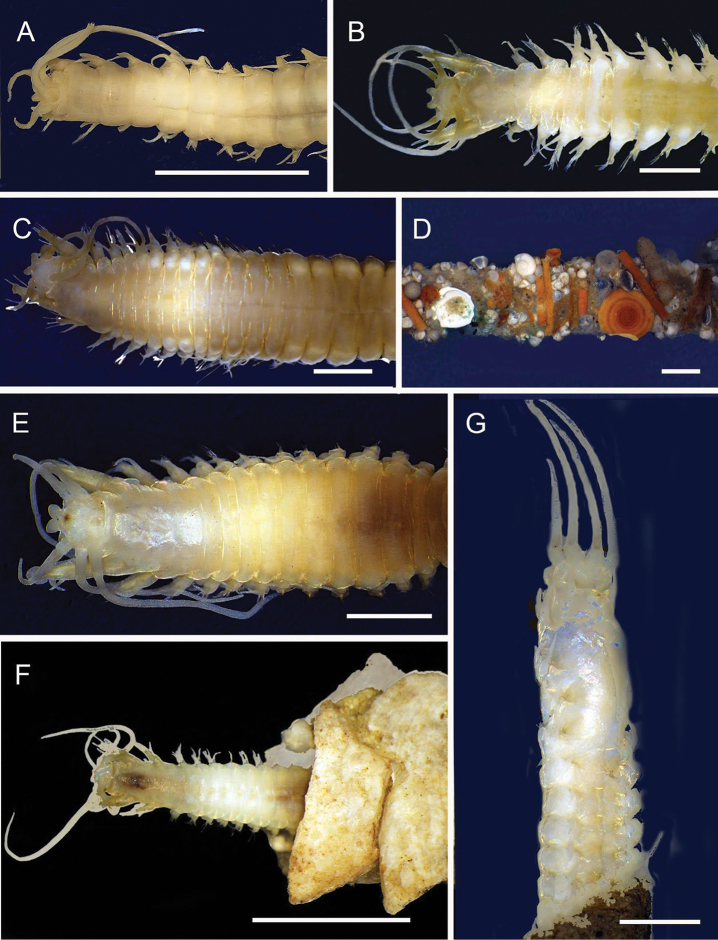
Onuphidae**A**Anchinothria cf. pycnobranchiata (op. 56) **B**Nothria cf. paxtonae (op. 56) **C***Nothria* sp. nov. 1 (AM W.49940) **D***Nothria* sp. nov. 1, tube (AM W.49940) **E***Nothria* sp. nov. 2 (AM W.49933) **F***Nothria* sp. nov. 3 (AM W.49934) **G***Paradiopatra* sp. nov. 2 (AM W.49950). Scale bars: 5 mm (**A**); 1 mm (**B–E, G**); 4 mm (**F**).

##### Hyalinoecia abranchiata

Taxon classificationAnimaliaPhyllodocidaOnuphidae

Lechapt, 1997

###### Diagnosis.

Frontal lips fused; palps subulate, with brown median patch. Eyes absent; peristomial cirri absent. Parapodium 1 enlarged; chaetigers 1 and 2 with bidentate simple hooks; limbate and scoop-shaped pectinate chaetae from chaetiger 2. Branchiae absent. Clear, quill-like tubes.

###### Remarks.

The species was originally described from abyssal zones off New Caledonia. It is new to Australian waters.

###### Records.

7 specimens. Suppl. material 1: ops. 80, 128 (AM).

##### Hyalinoecia

Taxon classificationAnimaliaPhyllodocidaOnuphidae

sp. nov. 2

###### Diagnosis.

Frontal lips fused; palps subulate, with brown median patch. Eyes absent; peristomial cirri absent. Parapodium 1 enlarged. Only chaetiger 1 with bidentate simple hooks; limbate and scoop-shaped pectinate chaetae from chaetiger 2. Branchiae absent. Clear, quill-like tubes.

###### Records.

43 specimens. Suppl. material 1: ops. 69, 100, 121 (AM).

##### Hyalinoecia

Taxon classificationAnimaliaPhyllodocidaOnuphidae

sp. 3

###### Diagnosis.

Frontal lips subulate; palps cirriform. Eyes absent; peristomial cirri absent. Parapodium 1 enlarged, with weakly bidentate simple hooks. Scoop-shaped pectinate chaetae and limbate chaetae from chaetiger 2. Single branchial filament from chaetiger 26. Clear, quill-like tubes.

###### Remarks.

Specimen is perhaps a juvenile *Hyalinoecia longibranchiata* (McIntosh, 1885).

###### Records.

1 specimen. Suppl. material 1: op. 69 (AM).

##### Leptoecia ultraabyssalis

Taxon classificationAnimaliaPhyllodocidaOnuphidae

(Kucheruk, 1977)

###### Diagnosis.

Frontal lips, peristomial cirri and branchiae absent. Chaetiger 1 enlarged. Parapodia 1 and 2 with bidentate simple and pseudocompound hooks. Limbate and pectinate chaetae from chaetiger 2. Subacicular hooks from chaetiger 13. Tube transparent, very delicate, quill-like.

###### Remarks.

The species was originally described from Philippine Trench in 6290–6330 m. It is new to Australian waters.

###### Records.

5 specimens. Suppl. material 1: ops. 42, 76 (AM). 12 specimens. Suppl. material 1: op. 9 (MV).

##### Nothria cf. paxtonae

Taxon classificationAnimaliaPhyllodocidaOnuphidae

Imajima, 1999

Fig. 16B

###### Diagnosis.

No eyes visible; peristomial cirri present. Chaetiger 1 enlarged; with auricular prechaetal lobes. Branchiae absent. Bidentate pseudocompound hooks on chaetigers 1 and 2. Chaetiger 3 with limbate chaetae only. Flat pectinate chaetae present from chaetiger 8–9. Subacicular hooks from chaetiger 8–9. Flattened tube, thin lining, covered with foraminifera.

###### Remarks.

This species was originally described from Japan, in 150 m depth. Japanese species is with eight papillae surrounding the anus, which cannot be confirmed for Australian specimens as all are incomplete. This species was also collected from the GAB reported as *Nothria* sp. (MacIntosh et al. 2018: additional file 2).

###### Records.

2 specimens. Suppl. material 1: op. 56 (AM).

##### Nothria

Taxon classificationAnimaliaPhyllodocidaOnuphidae

sp. nov. 1

Fig. 16C, D

###### Diagnosis.

Eyes absent; peristomial cirri present. Chaetiger 1 greatly enlarged; with auricular prechaetal lobes. Branchiae absent. Dorsal cirri absent from ~ chaetiger 30. Bidentate simple and pseudocompound hooks on chaetiger 1; bidentate compound hooks on chaetiger 2 and 3. ‘Scoop-shaped’ pectinate and limbate chaetae from chaetiger 2. Subacicular hooks from chaetiger 11–13. Flattened tube, clear inner layer, covered with pieces of shells and pebbles, elongate fragments placed transversely.

###### Remarks.

This species was also collected from the GAB reported as *Nothria* sp. (MacIntosh et al. 2018: additional file 2).

###### Records.

3 specimens. Suppl. material 1: ops. 4, 56 (AM).

##### Nothria

Taxon classificationAnimaliaPhyllodocidaOnuphidae

sp. nov. 2

Fig. 16E

###### Diagnosis.

Eyes absent; peristomial cirri present. Chaetiger 1 greatly enlarged; with auricular prechaetal lobes. Simple, short branchiae from chaetiger 10–12. Uni- to bidentate simple and pseudocompound hooks on chaetiger 1 and 2. Bidentate compound hooks on chaetiger 3. ‘Scoop-shaped’ pectinate and limbate chaetae from chaetiger 2. Subacicular hooks from chaetiger 11–14. Flattened tube, transparent lining, covered with pieces of shells and foraminifera.

###### Remarks.

This species was also collected from the GAB reported as *Nothria* sp. (MacIntosh et al. 2018: additional file 2).

###### Records.

2 specimens. Suppl. material 1: ops. 56, 90 (AM).

##### Nothria

Taxon classificationAnimaliaPhyllodocidaOnuphidae

sp. nov. 3

Fig. 16F

###### Diagnosis.

Eyes present; peristomial cirri present. Chaetiger 1 greatly enlarged; with auricular prechaetal lobes. Simple, short branchiae from chaetiger 11–14. Bidentate simple and pseudocompound hooks on chaetiger 1 and 2. Bidentate compound hooks on chaetiger 3. ‘Scooped-shaped’ pectinate and limbate chaetae from chaetiger 3. Subacicular hooks from chaetiger 9–12. Flattened tube with transparent inner layer, covered on outside with shell pieces, some larger than diameter of tube, spaces filled in with small particles.

###### Records.

40 specimens. Suppl. material 1: ops. 100, 121 (AM).

##### Nothria

Taxon classificationAnimaliaPhyllodocidaOnuphidae

sp. nov. 4

###### Diagnosis.

Eyes absent; peristomial cirri present. Chaetiger 1 enlarged; with auricular prechaetal lobes. Simple branchiae from chaetiger 10. Bidentate simple and pseudocompound hooks on chaetiger 1 and 2. Chaetiger 3 with limbate chaetae and ‘scoop-shaped’ pectinate chaetae only. Subacicular hooks from chaetiger 14–16. Flattened tube with transparent inner layer, covered on outside with foraminifera and small shell pieces.

###### Records.

3 specimens. Suppl. material 1: ops. 86, 115 (AM).

##### Paradiopatra ehlersi

Taxon classificationAnimaliaPhyllodocidaOnuphidae

(McIntosh, 1885)

###### Diagnosis.

Ceratophores without lateral projections. Peristomial cirri present. Almost unidentate and bidentate pseudocompound hooks with long pointed hoods on first three chaetigers. Subacicular hooks from chaetiger 10. Branchiae with single filaments from chaetiger 17–22, becoming pectinate. Tube with tough lining, outside muddy.

###### Remarks.

This species was previously reported NE of Sydney, collected from 4530 m during RV ‘Galathea’ expedition (Kirkegaard 1994).

###### Records.

3 specimens. Suppl. material 1: ops. 30, 65 (AM).

##### Paradiopatra

Taxon classificationAnimaliaPhyllodocidaOnuphidae

sp. nov. 1

###### Diagnosis.

Ceratophores without lateral projections. Peristomial cirri present. Bidentate pseudocompound hooks with long pointed hoods on first three chaetigers. Subacicular hooks from chaetiger 9. Branchiae with single filaments throughout, starting on chaetiger 14–16, filaments becoming very long. Thick mud tube.

###### Records.

11 specimens. Suppl. material 1: ops. 4, 35, 56, 70 (AM).

##### Paradiopatra

Taxon classificationAnimaliaPhyllodocidaOnuphidae

sp. nov. 2

Fig. 16G

###### Diagnosis.

Ceratophores with lateral projections. Almost unidentate pseudocompound hooks with long pointed hoods on first three chaetigers. Subacicular hooks from chaetiger 14. Branchiae absent. Mud tube.

###### Remarks.

This species was also recorded from 3794 m at the GAB as *Paradiopatra* sp. nov.

###### Records.

29 specimens. Suppl. material 1: ops. 33, 53, 54, 56, 78 (AM).

#### Family Opheliidae Malmgren, 1867

D. Ramos

Opheliids are usually elongate, tapering at both ends, with ventral grooves and reduced parapodial lobes (Magalhães et al. 2019). The family Opheliidae is composed of > 120 species in five genera (Magalhães et al. 2019). A comprehensive taxonomic revision of this family is needed given its confusing taxonomic history and evidence of paraphyly in *Ophelina*, the most speciose genus (Paul et al. 2010; Wiklund et al. 2019). *Travisia* was formerly included in Opheliidae, but has since been assigned its own family, which forms a sister group with Scalibregmatidae (Paul et al. 2010). Opheliids occupy intertidal to abyssal sandy and muddy environments (Hutchings 2000c). To date, there have been 30 species reported mostly in the shallow waters of Australia, with all currently accepted genera represented. These include 13 species of *Armandia*, six species each of *Ophelia* and *Ophelina*, one species of *Polyophthalmus*, and four species of *Thoracophelia* (Day and Hutchings 1979; Hutchings and Murray 1984; Hartmann-Schröder and Parker 1995; Neave and Glasby 2013; Parapar and Moreira 2015; Moreira and Parapar 2017). In this study we report at least six species from one genus, *Ophelina*.

##### Ophelina

Taxon classificationAnimaliaPhyllodocidaOpheliidae

sp.

###### Diagnosis.

Following synonymy of *Ammotrypanella* and *Ophelina* (Blake and Maciolek 2019a). Bluntly conical prostomium with oval palpode having an enlarged base. 32 chaetigers. Chaetiger 24–32 ventrally located and compressed. Chaetigers 1–6 shorter than midbody chaetigers, having more abundant chaetae. Branchial scars present from chaetigers 24–30. Anal funnel damaged, slightly longer than the last two posterior chaetigers, directed dorsally.

###### Records.

15 specimens. Suppl. material 1: ops. 9, 16, 27, 31, 33, 55, 79, 96 (AM). 46 specimens. Suppl. material 1: ops. 9, 16, 31, 33, 45, 54, 76, 79, 98, 110, 134 (NHMUK).

##### Ophelina cf. cirrosa

Taxon classificationAnimaliaPhyllodocidaOpheliidae

(Schüller, 2008)

Fig. 17A–C

###### Diagnosis.

Bluntly conical prostomium with slit-like nuchal organs. Eyes absent. Chaetigers 1–9 compressed, having more abundant chaetae. Midbody chaetigers longer than anterior and posterior chaetigers. Ventral and lateral grooves present along entire body. Fan-shaped parapodia. Branchiae from chaetiger 23 to chaetiger 30. Abranchiate chaetigers compressed, with the long chaetae directed dorsally. Anal funnel as long as last six posterior chaetigers, directed dorsally. Colour in ethanol white.

###### Remarks.

Observed specimens resemble *Ammotrypanella cirrosa* described from the Weddell Sea at 3050 m depth. These also possess an anal funnel with small cirri on the posterior margin but differ in having a large terminal ventral papilla instead of a ventral cirrus in the anal funnel. We followed the synonymy of *Ammotrypanella* and *Ophelina* as suggested by Blake and Maciolek (2019a).

###### Records.

76 specimens. Suppl. material 1: ops. 9, 11, 16, 23, 31, 76, 96, 134 (NHMUK).

**Figure 17.**
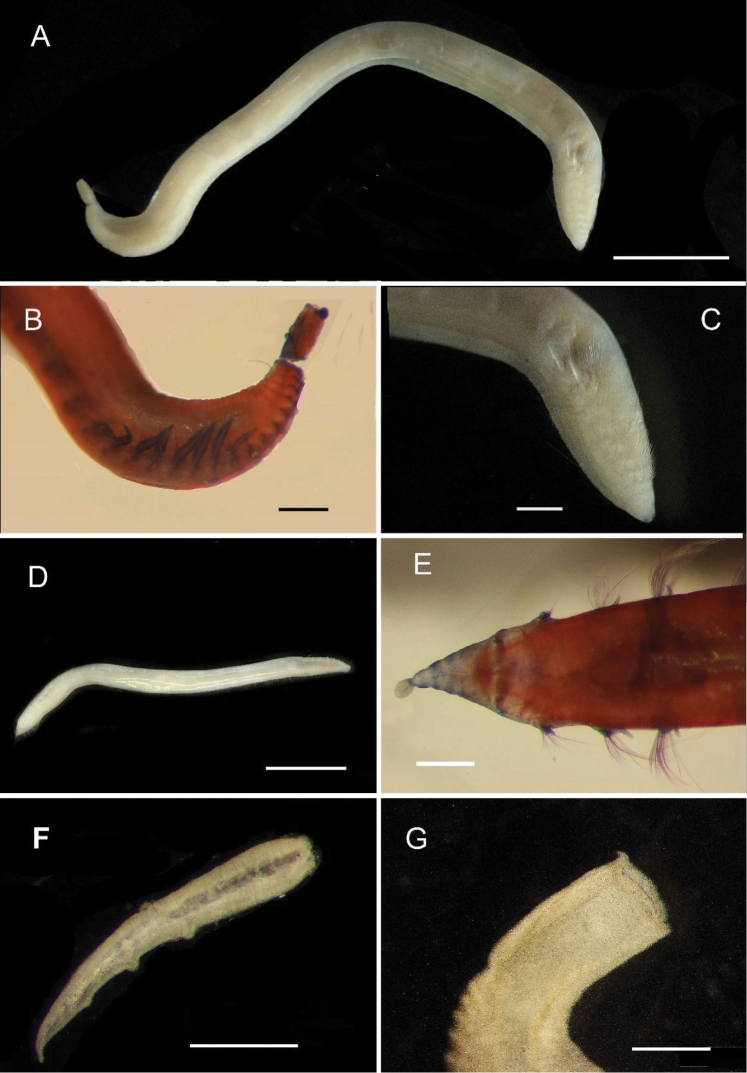
Opheliidae**A**Ophelina cf. cirrosa**B**Ophelina cf. cirrosa, posterior end **C**Ophelina cf. cirrosa, anterior end **D**Ophelina cf. helgolandiae**E**Ophelina cf. helgolandiae, prostomium **F**Ophelina cf. bowitzi, branchiae with blister-shaped bumps **G**Ophelina cf. bowitzi, anal funnel. Scale bars: 3 mm (**A**); 0.5 mm (**B, C**); 5 mm (**D**); 0.25 mm (**E, F**); 1 mm (**G**).

##### Ophelina cf. meyerae

Taxon classificationAnimaliaPhyllodocidaOpheliidae

Wiklund, Neal, Glover, Drennan, Rabone & Dahlgren, 2019

###### Diagnosis.

Bluntly conical prostomium with oval palpode. Eyes absent. 30 chaetigers. Midbody chaetigers longer than anterior and posterior chaetigers. Ventral and lateral grooves present along entire body. Wider space between parapodial rami on chaetigers 1–8. Chaetae all capillaries. Branchiae from chaetiger 2 to 29, largest posteriorly and smallest on midbody chaetigers. Anal funnel as long as last two posterior chaetigers, with thickened ventral keel and small terminal cirri. Colour in ethanol white.

###### Remarks.

Current specimens mostly conform to the description of *Ophelina meyerae*, the species was described from a single specimen from the Clarion-Clipperton Zone, Central Pacific at 4300 m depth. These specimens have their largest branchiae on the posterior end of the body unlike *O. meyerae*. They were inferred as the sister group to the latter using the 16S marker, with K2P distances of 2.15–2.40% (Ramos 2019).

###### Records.

138 specimens. Suppl. material 1: ops. 6, 9, 16, 31, 33, 45, 76, 79, 89, 96 (NHMUK).

##### Ophelina cf. helgolandiae

Taxon classificationAnimaliaPhyllodocidaOpheliidae

Augener, 1912

Fig. 17D, E

###### Diagnosis.

Prostomium triangular, longer than wide with oval palpode. Four annuli on prostomium revealed using Shirlastain. Eyes absent. 32 chaetigers. Chaetigers 2–7 having more abundant chaetae. Chaetigers 25–32 ventrally located and compressed. Branchiae on chaetigers 2–5, then on chaetigers 25–32. Anal funnel slightly longer than the last two posterior chaetigers, directed dorsally, with thickened ventral keel and small terminal cirri.

###### Remarks.

Observed specimens closely resemble *Ophelina helgolandiae* Augener, 1912, which is recorded from the Nordic Seas at depths of 600–1300 m (Kongsrud et al. 2011), but differ in having a pointed, annulated prostomium and lacking branchiae in the midbody chaetigers.

###### Records.

8 specimens. Suppl. material 1: ops. 23, 27, 40, 76 (NHMUK).

##### Ophelina cf. bowitzi

Taxon classificationAnimaliaPhyllodocidaOpheliidae

Parapar, Moreira & Helgason, 2011

Fig. 17F, G

###### Diagnosis.

Prostomium bluntly conical with distinct nuchal organs and an oval palpode. Eyes absent. Posterior chaetigers compressed. Deep ventral and lateral grooves. Branchiae starting from chaetiger 2. Chaetae all capillaries. Longest branchiae found posteriorly, the largest ones with blister-shaped bumps. Anal funnel bent dorsally, as long as last ten posterior chaetigers, becoming narrower from base to tip, and with short terminal cirri. Three specimens having a more rectangular anal tube with a thickened lip instead of terminal cirri. Colour in ethanol pale yellow.

###### Remarks.

Initial observations of these specimens show that they match the description of *Ophelina bowitzi*, which has previously been recorded only from the North Atlantic Ocean (Parapar et al. 2011; Kongsrud et al. 2013). Due to the distance between the collection and the type localities, we assign this species as Ophelina cf. bowitzi.

###### Records.

14 specimens. Suppl. material 1: ops. 9, 22, 23, 31, 45 (NHMUK).

##### Ophelinacf. juhazi

Taxon classificationAnimaliaPhyllodocidaOpheliidae

﻿Wiklund, Neal, Glover, Drennan, Rabone & Dahlgren, 2019

###### Diagnosis.

Prostomium conical (sunken in specimen) with teardrop-shaped palpode. Eyes absent. Deep ventral and lateral grooves. Parapodia small lobes with few chaetae. Chaetae all capillaries. Branchiae absent. Anal funnel cylindrical, length of last four chaetigers. Colour in ethanol pale yellow.

###### Remarks.

The specimen is morphologically similar to *O. juhaz*, but is found as a sister clade to it in initial COI and 16S phylogenies (Ramos 2019). Differs by having 30 chaetigers compared with 27 in *O. juhazi*.

###### Records.

1 specimen. Suppl. material 1: op. 42 (NHMUK).

#### Family Orbiniidae Hartman, 1942

A. Zhadan

Orbiniidae are deposit feeders burrowing in sediments, they range in size from a few millimetres to few centimetres and inhabit all depths from intertidal to abyssal. The body of the larger orbiniids is usually separated into a muscular dorsally flattened thorax and a more cylindrical abdomen; abdominal parapodia are shifted dorsally. Smaller species do not show such a separation of body regions. The parapodia are biramous; many genera bear notopodial and/or neuropodial postchaetal lobes. An autapomorphic character for Orbiniidae is the presence of camerated capillary chaetae with characteristic crenulations (Bleidorn and Helm 2019). There are 21 genera and ~ 240 species of Orbiniidae world-wide (Blake 2020) and 27 species from nine genera are known in Australian waters (http://www.ala.org.au). Australian Orbiniidae were studied by Day (1977), Hartmann-Schröder (1979, 1981, 1983, 1991), Mackie (1987) (genus *Leitoscoloplos* Day, 1977), Hutchings and Rainer (1979), Hutchings and Murray (1984), and Glasby (2000a), none were reported from abyssal depths. Eighteen species of Orbiniidae, 15 of which were new to science, have been reported from the deep-sea habitats (600–4880 m) in the Pacific Ocean and the South China Sea (Blake 2020). In total there are ~ 44 known species of Orbiniidae from deep-sea habitats: *Berkeleyia* (five), *Califia* (four), *Leitoscoloplos* (13), *Leodamas* (four), *Microrbinia* (one), *Naineris* (one), *Orbiniella* (ten), *Phylo* (one), and *Scoloplos* (five), of these *Berkeleyia*, *Califia*, *Microrbinia*, and *Orbiniella* are mainly composed of deep-water species (Blake 2020). In this study we report at least nine species from four genera.

##### Berkeleyia

Taxon classificationAnimaliaPhyllodocidaOrbiniidae

sp.

Fig. 18A

###### Diagnosis.

Posteriorly incomplete fragment ~ 4 mm long, 0.35 mm wide. Prostomium short, conical; one peristomial ring. Postchaetal lobes inconspicuous in anterior segments, becoming elongate digitiform in chaetiger 7. First seven chaetigers with tufts of long thin crenulated capillaries in both rami. Following chaetigers with smaller number of capillaries, neuropodia also bearing long thin slightly curved acicular spines with bidentate tips. Forked chaetae not observed.

###### Records.

1 specimen. Suppl. material 1: op. 79 (AM).

**Figure 18.**
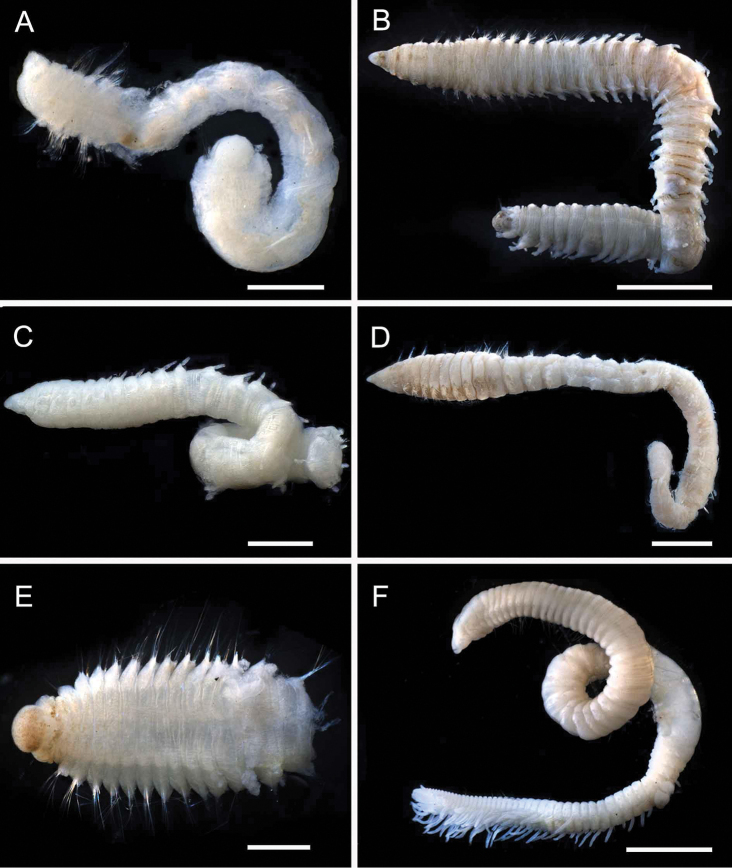
Orbiniidae**A***Berkeleyia* sp. (op. 79) **B**Leitoscoloplos cf. abranchiatus (op. 54) **C**Leitoscoloplos cf. kerguelensis (op. 96) **D***Leitoscoloplos* sp. 1 (op. 30) **E**Orbiniella cf. aciculata (op. 42) **F**Protoaricinae gen. sp. (op 100). Scale bars: 500 µm (**A, C, E**); 2 mm (**B, D**); 1 mm (**F**).

##### Leitoscoloplos cf. abranchiatus

Taxon classificationAnimaliaPhyllodocidaOrbiniidae

(Hartman, 1967a)

Fig. 18B

###### Diagnosis.

All specimens incomplete posteriorly. Longest fragment ~ 15 mm long, 1.3 mm wide, consisting of 41 chaetigerous segments. Thorax slightly flattened, abdomen cylindrical. Prostomium conical; one peristomial ring. Eleven thoracic chaetigers. Abdominal parapodia lateral in anterior abdomen, shifted dorsally on posteriorward segments. Branchiae from chaetiger 24, first short triangle, then becoming longer, strap-like. Thoracic postchaetal lobes conical in both rami; short in anterior thorax, becoming longer in middle and posterior parts; notopodial lobes longer. No subpodial or stomach papillae. Abdominal notopodia digitiform, same length as branchiae or shorter; abdominal neuropodia weakly bilobed, with short round lobes, inner slightly larger. Subpodial flange not developed, no flange papillae. All chaetae crenulated capillaries; forked chaetae not observed, probably broken. Colour in ethanol whitish-brown, with pigment spots on prostomium and in intersegmental furrows, or completely white.

###### Remarks.

*Leitoscoloplos abranchiatus* was described as entirely lacking branchiae, but all specimens studied were posteriorly incomplete (Hartman 1967a; Mackie 1987; Blake 2017). Specimens described here resemble *L. abranchiatus* by the number of thoracic chaetigers, shape of thoracic postchaetal lobes, shape and more lateral than dorsal position of abdominal parapodia.

###### Records.

4 specimens. Suppl. material 1: ops. 9, 16, 54 (AM).

##### Leitoscoloplos cf. kerguelensis

Taxon classificationAnimaliaPhyllodocidaOrbiniidae

(McIntosh, 1885)

Fig. 18C

###### Diagnosis.

Incomplete specimen, 5 mm long, 0.4 mm wide, consisting of 21 chaetigers. Body cylindrical, thoracic segments short, abdominal segments long. Prostomium conical with round tip, one peristomium ring. Nine thoracic chaetigers. Branchiae from chaetiger 17–19, exact position unknown. Thoracic postchaetal lobes short conical in anterior thorax, becoming long and oval in middle and posterior parts; notopodial lobes longer. No subpodial or stomach papillae. Abdominal parapodia small, with short lobes. Notopodia oval, shorter than in thorax; neuropodia weakly bilobed, with short subequal lobes. Chaetae supposedly all crenulated capillaries, but mostly broken; presence of uncini or forked chaetae unknown. Colour in ethanol white.

###### Remarks.

*Leitoscoloplos kerguelensis* is widespread in Antarctic and subantarctic seas, intertidal to 1400 m; it has 8–10 thoracic chaetigers and branchiae from chaetigers 13–17 (Blake 2017). The specimen studied here has close affinities with this species due to the number of thoracic chaetigers, late beginning of branchiae and the shape of postchaetal lobes. Due to the absence of accurate information on branchiae position, chaetal structure, the large distance from the species area, and bathymetric difference of material collected in this study we suggest the name Leitoscoloplos cf. kerguelensis.

###### Records.

1 specimen. Suppl. material 1: op. 96 (AM).

##### Leitoscoloplos cf. simplex

Taxon classificationAnimaliaPhyllodocidaOrbiniidae

Blake, 2017

###### Remarks.

Specimens resemble *Leitoscoloplos simplex* Blake, 2017 from Clarion-Clipperton Fracture Zone.

###### Records.

4 specimens. Suppl. material 1: 79 (AM).

##### Leitoscoloplos

Taxon classificationAnimaliaPhyllodocidaOrbiniidae

sp. 1

Fig. 18D

###### Diagnosis.

Incomplete specimen, 17 mm long, 1.9 mm wide. Thorax slightly flattened, abdomen cylindrical. First nine segments short, then becoming longer. Fourteen thoracic chaetigers, last one intermediate. Prostomial sharp conical; one peristomial ring. All thoracic segments and at least first three abdominal segments without branchiae. Thoracic postchaetal lobes short conical in both rami, notopodial lobes slightly longer. No subpodial or stomach papillae. Thoracic noto- and neurochaetae long crenulated capillaries. Abdominal region macerated; chaetae broken. Shape of abdominal parapodia and chaetae unknown. Colour in ethanol brown-yellowish.

###### Remarks.

This specimen is similar to *L. abranchiatus* but has more thoracic chaetigers and shorter thoracic postchaetal lobes. Two OTUs assigned to *Leitoscoloplos* (four stations, 486–2224 m) were recorded in the GAB (MacIntosh et al. 2018: additional file 2), but further investigation is required to determine if any of the species from this study match those from the GAB.

###### Records.

1 specimen. Suppl. material 1: op. 30 (AM).

##### ? Leitoscoloplos spp.

Taxon classificationAnimaliaPhyllodocidaOrbiniidae

###### Remarks.

Brenke sledge specimens were incomplete and poorly preserved which does not allow further identification.

###### Records.

3 specimens. Suppl. material 1: ops. 31, 79, 119 (AM).

##### Orbiniella cf. aciculata

Taxon classificationAnimaliaPhyllodocidaOrbiniidae

Blake, 1985

Fig. 18E

###### Diagnosis.

All specimens represented by short anterior fragments 1.2–2.5 mm long, 0.6–1 mm wide. Prostomium rounded, wider than long; two peristomial rings. No branchiae. Parapodial rami conical, prominent without postchaetal lobes. Both rami with thin crenulated capillaries and one or two short thick acicular spines. Colour in ethanol white with brown pigmentation on dorsal side of prostomium and peristomium, and on ventral side of anterior segments.

###### Records.

4 specimens. Suppl. material 1: op. 42 (AM).

##### Orbiniella sp. nov.

Taxon classificationAnimaliaPhyllodocidaOrbiniidae

###### Records.

2 specimens. Suppl. material 1: op. 31 (AM).

##### Protoaricinae gen. spp.

Taxon classificationAnimaliaPhyllodocidaOrbiniidae

Fig. 18F

###### Diagnosis.

Body long and thin, < 95 chaetigers, < 15 mm long and 0.5 mm wide; without clear division on thorax and abdomen; parapodia not shifting dorsally in posterior segments. Prostomium short, bluntly conical with round tip; two peristomial rings. Branchiae from chaetiger 15–30 as short lobes, becoming very long and prominent on posterior segments. Parapodia with widely arranged rami, chaetal tufts emerging from low tubercles. Anterior neuropodia without postchaetal lobes, short conical lobes appearing ~ chaetiger 7 or beyond, becoming long posteriorward, disappearing in posterior segments; some specimens without neuropodial lobes. No notopodial postchaetal lobes. Notopodia and neuropodia with crenulated capillaries and long thin acicular spines in all segments; spines absent in juveniles. Pygidium with two lobes. Colour in ethanol white.

###### Remarks.

Genus is uncertain, probably new. Two morphospecies: one with neuropodial postchaetal lobes and another without.

###### Records.

127 specimens. Suppl. material 1: op. 100 (AM).

##### Orbiniidae

Taxon classificationAnimaliaPhyllodocidaOrbiniidae

gen. sp.

###### Remarks.

Beam trawl specimens were incomplete and poorly preserved which does not allow further identification. Brenke sledge samples were identified to family level.

###### Records.

6 specimens. Suppl. material 1: ops. 31, 40, 42, 54 100 (AM). 2 specimens. Suppl. material 1: ops. 16, 42 (NHMUK).

#### Family Oweniidae Rioja, 1917

P. Hutchings

Oweniids are slender, fragile annelids which have a cylindrical body composed of relatively few segments and reduced parapodia. They live inside tightly fitting tubes made of cemented sand grains, shell fragments, or Foraminifera tests. The family is composed of four genera and ~ 60 species (Capa et al. 2019a). Common in the soft sediments of continental shelves, they are also found intertidally and in shallow subtidal habitats, including seagrass beds. Oweniids of the genera *Galathowenia* and *Myriochele* have been reported from deep waters (2770 m and 396 m respectively) (Blake 2000) and *Myriochele* sp. was reported from the Kermadec Trench down to 8300 m (Kirkegaard 1956). A study on Australian oweniids from intertidal to 70 m reported four genera and ten species, including two specimens only described to genus (Capa et al. 2012). There were no previous records of oweniids from Australian deep waters. All material from the present study was in poor condition because the animals were fixed inside the tubes, which makes extraction of intact specimens difficult. This study reports at least one species from one genus.

##### Myriowenia spp.

Taxon classificationAnimaliaPhyllodocidaOweniidae

Fig. 2G

###### Diagnosis.

Head with large grooved palps and bilobed prostomium, mouth anteroventral, with ventral pharyngeal organ. First three segments uniramous, with capillary notochaetae. Subsequent segments biramous with capillary notochaetae and neuropodial uncini with teeth arranged in a vertical position.

###### Remarks.

At least two species are present based on tubes, one very substantial tube, other fine, difficult to extract entire animal from tube but all are characterised by a pair of large grooved palps. The genus *Myriowenia* is represented by four species, one from California, two from the Gulf of Mexico, and an undescribed species from Australia (Capa et al. 2012). Capa et al. (2012), while providing a detailed description, did not formally describe the specimen as a new species because previously described species did not provide detailed information on intraspecific differences in diagnostic characters.

###### Records.

14 specimens. Suppl. material 1: ops. 43, 44, 56, 65; 78, 99, 135 (AM).

##### Oweniidae gen. spp.

Taxon classificationAnimaliaPhyllodocidaParaonidae

###### Remarks.

Material is too damaged to be identified further or Brenke sledge material was identified to family level only.

###### Records.

18 specimens. Suppl. material 1: ops. 27, 30, 40, 43, 54, 56, 78, 99 (AM).

#### Family Paraonidae Cerruti, 1909

J. Langeneck, D. Ramos

Paraonids are small, elongate worms. They have a well-defined prostomium on which some genera have one distinct median antenna. The family Paraonidae includes ~ 140 described species (Blake 2019b). The family is currently divided into eight genera, one of which is further split into four subgenera (Blake 2019b), but molecular data suggest that substantial rearrangements are needed (Langeneck et al. 2019a). Paraonidae typically occur in soft sediments and are especially abundant in shelf to slope depths, where they represent one of the most abundant groups (Blake 2019b), they also show high diversity in bathyal and abyssal environments (Aguirrezabalaga and Gil 2009; Langeneck et al. 2019b). The diversity of Paraonidae is likely largely underestimated even in the best studied geographic areas (Blake 2019b; Langeneck et al. 2019a). In Australian waters this group is poorly known, and published data only refer to shallow environments, whereas the majority of collection data are still unpublished, and presumably a high number of species remains undescribed (Glasby 2000b). This study yielded 23 specimens of Paraonidae belonging to four taxa. Although all specimens could be assigned, at least tentatively, to described species, the frequent occurrence of pseudocryptic taxa in this family and the type localities often far apart from the eastern Australia suggest that they may be undescribed, and that integrative taxonomy is needed to clarify the diversity of this family.

##### Aricidea

Taxon classificationAnimaliaPhyllodocidaParaonidae

sp.

Fig. 19A, B

###### Diagnosis.

Three-lobed prostomium with deep nuchal grooves flanking a cirriform median antenna reaching chaetiger 3. Narrow elongated body. Notopodial postchaetal lobe becoming distinctly cirriform on chaetiger 8. Papilliform neuropodial postchaetal lobe. Cirriform branchiae from chaetigers 4–7. Notopodia with simple capillaries, neuropodia with hooks and capillaries with long arista.

###### Records.

6 specimens. Suppl. material 1: ops. 11, 31, 33, 54 (NHMUK).

**Figure 19.**
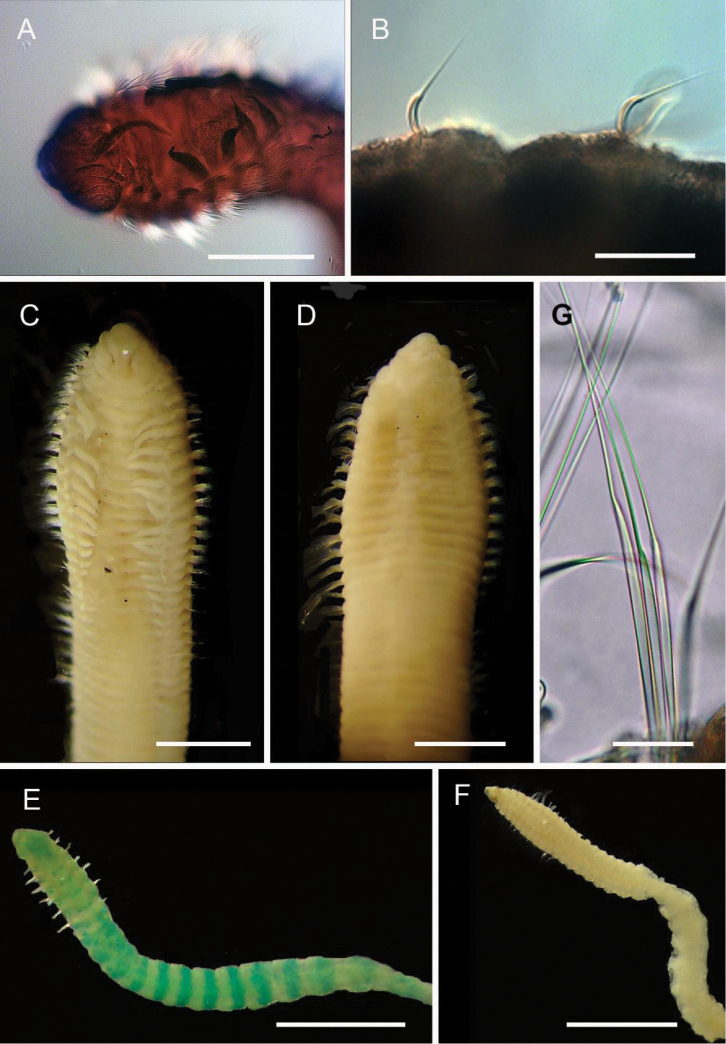
Paraonidae**A***Aricidea* sp., prostomium with median antennae **B** same, capillary with arista **C**Aricidea cf. simplex, dorsal view anterior (AM W.52307) **D** same, ventral view anterior (AM W.52307) **E***Levinsenia uncinata* (AM W.52320), methyl green stained dorsal view **F***Paraonella* sp. 1 (AM W.52310) **G**Paraonis cf. quadrilobata chaetae (AM W.52307). Scale bars: 250 µm (**A**); 50 µm (**B**); 1 mm (**C, D, E, F**); 10 µm (**G**).

##### Aricidea cf. simplex

Taxon classificationAnimaliaPhyllodocidaParaonidae

Day, 1963b

Fig. 19C, D

###### Diagnosis.

All specimens consist of anterior fragments; most complete with 56 chaetigers, 12.7 mm total length, 1.45 mm maximum width. Prostomium sub-trapezoidal, with large, conspicuous nuchal organs showing dark brown pigmentation. Antenna very short, clavate, reaching the anterior margin of the first chaetiger (absent, likely broken, in one individual). Three pre-branchial chaetigers, 14–17 pairs of acute, relatively short branchiae; last five or six pairs of branchiae gradually decreasing in size. Notopodial lobes tubercular in the first two chaetigers, spindle-shaped in chaetigers 3–15, gradually thinner and thread-like from chaetiger 16 to the end of the fragment. Neuropodial post-chaetal lobes inconspicuous. Notopodial modified chaetae absent. Neuropodial modified chaetae from chaetiger 32, first one or two, then up to five (possibly more in the posterior part of the body, missing in all examined specimens), strong, slightly reddish hooks with bent tip.

###### Remarks.

The examined specimens are very similar to each other and are clearly similar to ‘Aricidea’ simplex as described by Blake (1996a) on the basis of shelf specimens. However, molecular data showed that this taxon represents a species complex (Brasier et al. 2016; Langeneck et al. 2019a) and that interspecific differences are most likely concealed by its extremely simple anatomy and the absence of reliable diagnostic characters. *Aricidea neosuecica* Hartman, 1965 (with type locality in the West Atlantic) and *Aricidea neosuecica nipponica* Imajima, 1973 (with type locality off Japan) have been considered synonymous with *A. simplex*, but may also represent separate species. As *A. simplex* was described for relatively shallow environments in South Africa (Day 1963b), this present deep-sea species most likely does not correspond to the taxon sensu stricto.

###### Records.

3 specimens. Suppl. material 1: ops. 31, 40, 42 (AM).

##### Levinsenia uncinata

Taxon classificationAnimaliaPhyllodocidaParaonidae

(Hartman, 1965)

Fig. 19E

###### Diagnosis.

Body thread-like, posteriorly incomplete, ~ 12 mm for 35 chaetigers, 0.33 mm maximum width. Prostomium triangular, with apical organ, without eyes, without prostomial antenna, nuchal organs as thin slits on the posterior part of the prostomium. Branchiae absent. Notopodial post-chaetal lobes inconspicuous in the anterior part of the body, posterior part of the body too damaged to determine. Modified neuropodial chaetae after chaetiger 18, first two, then up to four or five, strong, thick and slightly curved hooks with well-developed sub-distal dorsal sheath. Methyl green staining pattern: a ventral median rectangular dot on chaetigers 1–5; ventrally complete bands (dorsally open) from chaetiger 6 to chaetiger 17; nuchal slits pigmented.

###### Remarks.

The examined specimen corresponds well to the original description by Hartman (1965) and the redescription by Strelzov (1973). This species seems to be widespread in bathyal to abyssal environments of the Atlantic and Pacific Ocean and may be a species complex.

###### Records.

1 specimen. Suppl. material 1: op. 56 (AM).

##### Paraonella

Taxon classificationAnimaliaPhyllodocidaParaonidae

sp. 1

Fig. 19F

###### Diagnosis.

Complete specimen with 72 chaetigers, 0.3 mm maximum width, 8 mm total length. Prostomium oval, without apical organ, without eyes, two large nuchal organs, often with rusty pigmentation. Branchiae absent, notopodial post-chaetal lobes finger-like from chaetiger 1 to chaetiger 9, triangular, short afterwards, increasing in length for last 15 chaetigers. Pygidium rounded with three cirri approximately of the same length. Chaetae all capillaries. Methyl green staining: no pattern. The only complete specimen partially in a brittle, mucous tube.

###### Remarks.

The absence of modified chaetae and prostomial antenna allows the assignation of these specimens to the genus *Paraonella* Strelzov, 1973. Currently the genus includes eight species, three of which are abranchiate, namely *Paraonella monilaris* (Hartman & Fauchald, 1971), *Paraonella myriamae* (Laubier & Ramos, 1974) and *Paraonella abranchiata* Fauchald & Hancock, 1981. Both *P. myriamae* and *P. abranchiata* are characterised by triangular prostomium, and can be readily distinguished from *Paraonella* sp. 1; *P. monilaris*, instead, has a rounded prostomium and a similar pattern of notopodial lobes, and might be closer to this species, even though the structure of nuchal organs is not clear from the original drawings. However, *P. monilaris* has moniliform segments, with clear constrictions in between, while *Paraonella* sp. has less pronounced constrictions and shorter segments. Moreover, although the size of the specimens examined by Hartman and Fauchald (1971) is similar (< 8 mm for 71 chaetigers), they are slenderer (0.23 mm vs. 0.27–0.35 mm maximum width). According to the original drawing, notopodial lobes are proportionally shorter in *P. monilaris*, but the pattern described by Hartman and Fauchald (1971) (lobes short, increasing until the eighth chaetiger, then shorter) is very similar to that observed in these specimens. Although the ecology of the two species is very similar and morphological features largely overlap, the difference between *P. monilaris* and *Paraonella* sp. is unclear as the original description of *P. monilaris* lacks detail.

*Paraonella* as currently described is most likely polyphyletic, including species close to *Paradoneis* Hartman, 1965 and to *Paraonis* Grube, 1873. The pattern of notopodial lobes observed in this species clearly resembles that occurring in *Paradoneis*, as in the majority of the known *Paraonella* species. However, there is the possibility that *Paradoneis*-like *Paraonella* also represent separate lineages that independently lost the modified notochaetae.

###### Records.

11 specimens. Suppl. material 1: ops. 33, 54 (AM).

##### Paraonis cf. quadrilobata

Taxon classificationAnimaliaPhyllodocidaParaonidae

(Webster & Benedict, 1887)

Fig. 19G

###### Diagnosis.

All specimens anterior fragments; most complete specimen with 47 chaetigers (into two pieces), ~ 10 mm length, 1.2 mm maximum width. Prostomium sub-triangular, wider than long, with two large, conspicuous nuchal organs, showing traces of dark pigmentation. Antenna slender, thread-like, reaching chaetiger 3–7 backwards (tip often broken). Three pre-branchial chaetigers, five to 12 pairs of flattened branchiae. Branchial region wider and slightly flattened. Notopodial post-chaetal lobes tubercular in the first three chaetigers, then slender, elongated, with bulbous base in chaetigers 4–15, thread-like from chaetiger 16 to the end of the body. Neuropodial post-chaetal lobes conical, well-developed, in the first 15–17 chaetigers. Notopodial modified chaetae absent. Neuropodial modified chaetae occuring after chaetiger 25 thickened capillaries, with abruptly tapered tips. Remains of thin, dark transverse bars on the dorsal side of the branchial region. The largest specimen showing oocytes (140 × 110 μm) in the coelom of the post-branchial region.

###### Remarks.

These specimens correspond well to material sampled in the sub-arctic Atlantic Ocean (Norway) in regard to size and number of branchiae. However, *P. quadrilobata* has been reported from all over the world and from different depths, and most likely represents a species complex (unpublished molecular data point at a separation at least between North Atlantic and Mediterranean specimens, the latter described as *Aricidea annae* Laubier, 1967 which is now *Paraonis annae*). Blake (1996a) suggested that the majority of Pacific specimens should be assigned to *Aricidea antennata* Annenkova, 1934 (now *Paraonis antennata*), but the examined specimens have simple notopodial post-chaetal lobes (instead of branched ones as in *A. antennata*).

###### Records.

8 specimens. Suppl. material 1: ops. 31, 33, 40; 42 (AM).

##### Paraonidae gen. spp.

Taxon classificationAnimaliaPhyllodocidaParaonidae

###### Remarks.

Brenke sledge samples were identified to family level. Seven OTUs not yet confidently assigned to genus (23 stations, 416–2850 m) were recorded in the GAB (MacIntosh et al. 2018: additional file 2), further investigation is required to determine if any of the species in this study are the same as those from the GAB.

###### Records.

1 specimen. Suppl. material 1: op. 23 (NHMUK). 2 specimens. Suppl. material 1: ops. 33, 40 (AM).

#### Family Pectinariidae Quatrefages, 1866

E. K. Kupriyanova, J. Zhang

Pectinariids are easily recognisable by their stout golden paleae, and distinctive tubes made of cemented sand grains that resemble an ice-cream cone. The family Pectinariidae is composed of five genera and 63 accepted species (Read and Fauchald 2020). Pectinariids are mostly found at subtidal and shelf depths, the only exception until recently was *Petta assimilis* McIntosh, 1885 collected from 2926 m. In Australia pectinariids have been collected from intertidal and subtidal habitats (Hartman 1966a; Day and Hutchings 1979; Hutchings and Peart 2002; Wong and Hutchings 2015). A recent review of Australian pectinariids (Zhang and Hutchings 2019) reported 13 species from three genera (*Amphictene* Savigny, 1822, *Lagis* Malmgren, 1866 and *Pectinaria* Lamarck, 1818). Material from the present study was used to describe two deep-sea *Petta* species, *P. investigatoris* Zhang, Hutchings & Kupriyanova, 2019 and *P. williamsonae* Zhang, Hutchings & Kupriyanova, 2019, reported here.

##### Petta investigatoris

Taxon classificationAnimaliaPhyllodocidaPectinariidae

Zhang, Hutchings & Kupriyanova, 2019

Fig. 20A–C

###### Diagnosis.

Cephalic veil completely free from operculum, with smooth or bearing several lappets (slightly raised mounds) anterior margin. Operculum semi-circular with smooth dorsal and lateral margins. Ventral margin of operculum with a transverse row of numerous stout notopodial paleae on each side. Two pairs of comb-like branchiae on segments 3 and 4, consisting of large basal hump and series of well separated free lamellae. Pair of dorso-lateral pads on segment 5. Ventro-lateral lobes with continuous row of papillae on segment 3. Notopodia with paleae on segment 1 and with notochaetae on segments 5–21 (17 pairs). Neuropodia present on segments 8–21, > 14 pairs with transverse tori, each with a row of uncini. Scaphe indistinctly separated from posterior segments.

###### Remarks.

Type locality is Jervis Marine Park, eastern Australia, 2650–2636 m.

###### Records.

6 specimens. Suppl. material 1: ops. 4, 11, 22, 35, 56 (AM).

**Figure 20.**
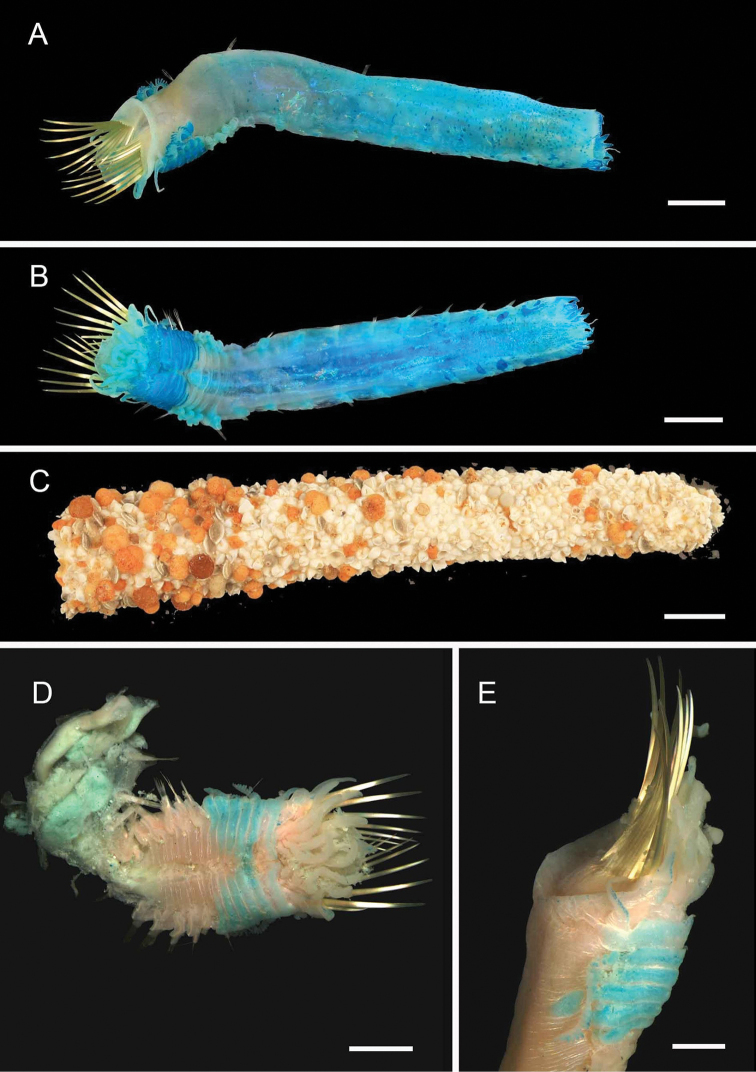
Pectinariidae*Petta investigatoris* (AM W.50672) **A** ventral view **B** dorsal view **C** tube. *Petta williamsonae* (AM W.50667) **D** ventral view **E** anterior end, lateral view. Scale bars: 2 mm (**A, B**); 5 mm (**C**); 1 mm (**D**); 0.5 mm (**E**).

##### Petta williamsonae

Taxon classificationAnimaliaPhyllodocidaPectinariidae

Zhang, Hutchings & Kupriyanova, 2019

Fig. 20D, E

###### Diagnosis.

Cephalic veil completely free from operculum, with smooth or bearing several lappets (slightly raised mounds) anterior margin. Operculum semi-circular with smooth dorsal and lateral margins. Ventral margin of operculum with a transverse row of numerous stout notopodial paleae on each side. Two pairs of comb-like branchiae on segments 3 and 4, consisting of large basal hump and series of well separated free lamellae. Pair of dorso-lateral pads on segment 5. Ventro-lateral lobes smooth on segment 3. Notopodia with paleae on segment 1 and with notochaetae on segments 5–21 (17 pairs). Neuropodia present on segments 8–21, > 14 pairs with transverse tori, each with a row of uncini. Scaphe distinctly separated from posterior segments.

###### Remarks.

Type locality is Bass Strait, eastern Australia, 2760–2692 m.

###### Records.

2 specimens. Suppl. material 1: op. 22 (AM).

##### Pectinariidae gen. spp.

Taxon classificationAnimaliaPhyllodocidaPectinariidae

###### Remarks.

Material is too damaged, no further identification is possible.

###### Records.

2 specimens. Suppl. material 1: op. 11 (AM). 5 specimens. Suppl. material 1: op. 89 (NHMUK).

#### Family Phyllodocidae Örsted, 1843

D. Ramos, R. S. Wilson

Phyllodocids are commonly known as ‘paddle-worms’ due to their large leaf-like dorsal cirri. There are currently 31 extant genera and 497 accepted species (Read and Fauchald 2020). Benthic Phyllodocidae (pelagic phyllodocids were not sampled in this study) are most common and diverse in shallow waters, especially associated with hard substrates, but some genera inhabit mud flats. However, most genera are also represented in bathyal and abyssal habitats (Blake 1997; Böggemann 2009; Paterson et al. 2009). In Australian waters 15 genera and 30 named species have been reported (http://www.ala.org.au). In this study we report at least five species from three genera.

##### Clavadoce

Taxon classificationAnimaliaPhyllodocidaPhyllodocidae

sp.

###### Records.

1 specimen. Suppl. material 1: op. 69 (AM).

##### Eumida

Taxon classificationAnimaliaPhyllodocidaPhyllodocidae

sp.

###### Records.

36 specimens. Suppl. material 1: ops. 13, 44, 88, 100 (AM).

##### Eumida cf. angolensis

Taxon classificationAnimaliaPhyllodocidaPhyllodocidae

Böggemann, 2009

Fig. 21A

###### Diagnosis.

Prostomium wider than long, with three antennae and two palps. Antennae and palps digitiform. Eyes absent. Tentacular cirri with broad base tapering to a fine tip, four pairs on anterior three segments (1–2–1 arrangement). First tentacular segment dorsally reduced. Everted proboscis barrel-shaped with round terminal papilla. Parapodia uniramous. Chaetae present from segment 2. Dorsal cirri lanceolate, ventral cirri conical, both approximately as long as the neuropodia. Colour in ethanol pale yellow.

###### Remarks.

This specimen closely resembles *Eumida angolensis* Böggemann, 2009, described from the Angola Basin at depths of 3950–5443 m. Alalykina (2018) reports the presence of Eumida cf. angolensis in the Sea of Okhotsk at 1676–3366 m, which would expand its range to the Pacific Ocean if confirmed. Unfortunately, no descriptions were provided for these specimens that would allow comparisons with the Australian sample. We currently consider this as a different species due to the distance from the type locality and some observed morphological differences such as having 18 instead of 16 terminal proboscideal papilla and bearing more neurochaetae per fascicle.

###### Records.

1 specimen, anterior fragment only. Suppl. material 1: op. 100 (NHMUK).

**Figure 21.**
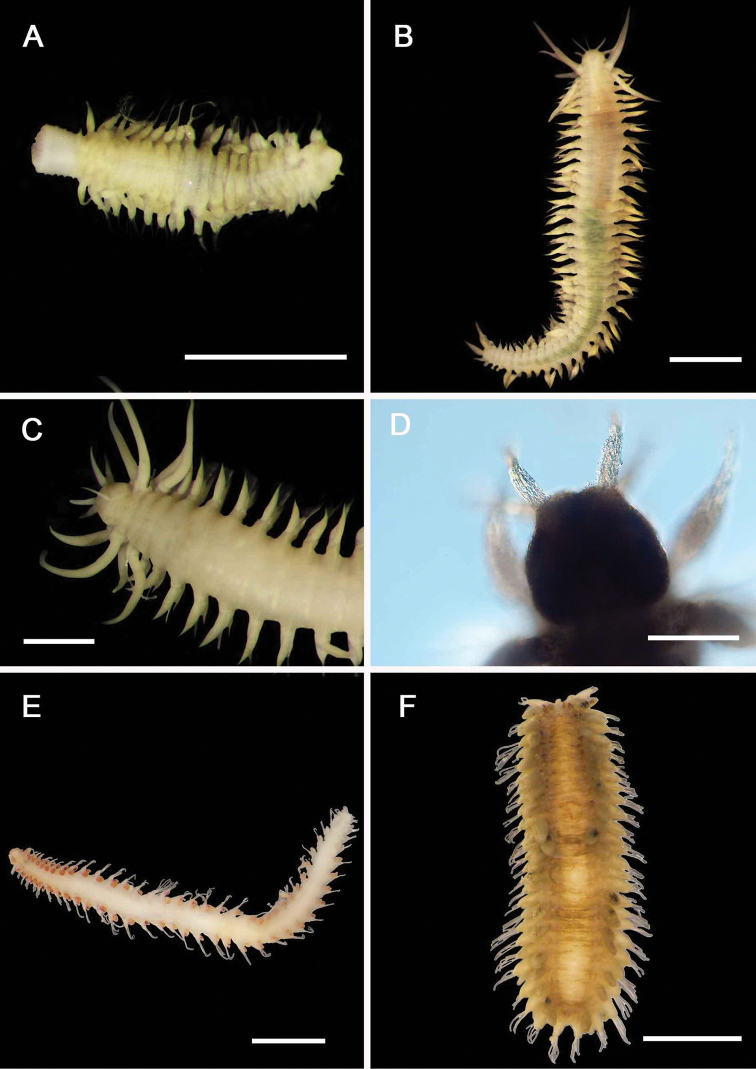
Phyllodocidae**A**Eumida cf. angolensis**B**Eumida cf. longicirrata, live specimen **C**Eumida cf. longicirrata, prostomium **D** ?*Pseudomystides* sp., prostomium **E**Phyllodocidae sp. (op. 79) **F**Phyllodocidae sp. (AM W.52332). Scale bars: 1 mm (**A, B, E, F**); 0.5 mm (**C**); 100 µm (**D**).

##### Eumida cf. longicirrata

Taxon classificationAnimaliaPhyllodocidaPhyllodocidae

Hartmann-Schröder, 1975

Fig. 21B, C

###### Diagnosis.

Broadly triangular prostomium (Fig. 21C) with three antennae and two palps. Antennae and palps digitiform. Eyes absent. Four pairs of cylindrical tentacular cirri on anterior three segments (1–2–1 arrangement). First tentacular segment dorsally reduced. Everted proboscis funnel-shaped with digitiform terminal papillae. Parapodia uniramous. Chaetae present from segment 2. Dorsal cirri lanceolate, ventral cirri conical. Raised postero-dorsal semi-circular structures not as distinct as in the description of Eibye-Jacobsen (1991). Colour in ethanol pale yellow.

###### Remarks.

Current specimens closely resemble the redescription of *Eumida longicirrata* (Eibye-Jacobsen 1991), although the raised postero-dorsal semi-circular structures were not as distinct, and in some cases not visible at all. Similar observations were noted by Ravara et al. (2017b) for *E. longicirrata* collected from the Gulf of Cadiz. Considering that the recorded distribution of *E. longicirrata* appears to be limited to the margins of the Iberian Peninsula (Ravara et al. 2017b), we tentatively regard this as a different species until further investigation.

###### Records.

23 specimens. Suppl. material 1: op. 100 (NHMUK).

##### ? Pseudomystides

Taxon classificationAnimaliaPhyllodocidaPhyllodocidae

sp.

Fig. 21D

###### Diagnosis.

Prostomium broadly triangular and cleft anteriorly, with three antennae and two palps. Antennae and palps digitiform, ~ as long as prostomium. Short bodies with few segments (11–14). Tentacular cirri with broad base tapering to a fine tip, three pairs on anterior two segments (1–2 arrangement). Dorsal cirri absent on segment 3. Parapodia uniramous. Dorsal cirri lanceolate, slightly shorter than neuropodia. Ventral cirri digitiform. A pair of tear-drop-shaped anal cirri and a small median papilla. Colour in ethanol pale yellow to brown.

###### Records.

13 specimens. Suppl. material 1: op. 100 (NHMUK). 145 specimens. Suppl. material 1: op. 100 (AM).

##### Phyllodocidae gen. spp.

Taxon classificationAnimaliaPhyllodocidaPhyllodocidae

Fig. 21E, F

###### Remarks.

Brenke sledge material is not identified past family level. *Phyllodoce duplex* (5 stations, 410–1836 m) and three specimens belonging to *Protomystides* and *Pseudomystides* (3 stations, 995–1154 m) were recorded in the GAB (MacIntosh et al. 2018: additional file 2). Further investigation is required to determine if the species in the present study are the same as those at the GAB.

###### Records.

20 specimens. Suppl. material 1: ops. 16, 31, 33, 40, 42, 43, 44, 79 (AM). 1 specimen. Suppl. material 1: op. 100 (NHMUK).

#### Family Pilargidae Saint-Joseph, 1899

C.J. Glasby

Pilargidae are free-living sediment dwellers with similarities to the nereidiforms (Fitzhugh and Wolf 1990; Glasby 1993; Dahlgren et al. 2002), although more recent molecular studies suggest a sister group relationship with Nephtyidae (Zrzavý et al. 2009; Struck and Halanych 2010). There are currently 11 valid genera and ~ 100 species (Read and Fauchald 2020). The family has a worldwide distribution in marine and estuarine soft sediments; they are most common at shelf, slope and deeper depths, but can be found at all depths (Parapar et al. 2004; Hocknull and Glasby 2009). In Australian waters, eight genera and 13 named species have been reported (http://www.ala.org.au). Although pilargids are seldom frequent or abundant, their representation in this study by only a few specimens is unusual and suggests under-sampling. Here we report two species, both of which appear to be new to science, from two genera.

##### Ancistrosyllis

Taxon classificationAnimaliaPhyllodocidaPilargidae

sp. 1

###### Diagnosis.

Small specimen, incomplete. Median antenna short, approximately same length as laterals. Dorsal cirri of chaetiger 1 ~ 1 × longer than following. Dorsal hooks starting from chaetiger 6. Ventral cirri starting on chaetiger 3. Verrucae present on body surface, short and sparse. Colour in ethanol white.

###### Remarks.

The genus is only known in Australia from a single named species, A. cf. hartmanae Pettibone, 1966 (Hocknull and Glasby 2009) from northern Australia, and *Ancistrosyllis* sp. from Dampier, WA (Hartmann-Schröder 1980). The present material does not resemble either of these species so probably represents a new species.

###### Records.

1 specimen. Suppl. material 1: op. 40 (AM).

##### Sigambra

Taxon classificationAnimaliaPhyllodocidaPilargidae

sp. 1

###### Diagnosis.

Small specimen, complete, 47 chaetigers. Pharynx with eight or nine terminal papillae. Dorsal cirri (except for first) slightly longer than ventral cirri. Dorsal hooks starting from chaetiger 3, extending to within a few segments from pygidium; accompanied by one or two small capillary chaetae. Neurochaetae smooth, broad-bladed capillaries of varying lengths. Chaetiger 2 with ventral cirri. Median antenna much longer than laterals, extending back to chaetiger 6; first dorsal cirri cirriform, several times longer than following ones which are slender, foliose. Colour in ethanol white.

###### Remarks.

The specimen is similar to *S. magnuncus* Paterson & Glover, 2000. It probably represents a new species.

###### Records.

1 specimen. Suppl. material 1: op. 96 (AM).

##### Pilargidae gen. spp.

Taxon classificationAnimaliaPhyllodocidaPilargidae

###### Records.

1 specimen. Suppl. material 1: op. 134 (AM).

#### Family Polynoidae Kinberg, 1856

A. Murray, R. S. Wilson

The Polynoidae is the most species-rich of the seven families of Aphroditiformia, commonly known as scale-worms (Read and Fauchald 2020). Although molecular studies have shown the family to be monophyletic, the character supporting the Polynoidae clade (presence of tubercles on the elytra) exhibits many subsequent reversals and there is no morphological diagnosis that separates Polynoidae from other scale worms, nor do all scale-worms have scales (elytra) (Wiklund et al. 2005; Norlinder et al. 2012; Gonzalez et al. 2018). The Polynoidae are widely distributed geographically and ecologically, occurring in all depths from intertidal waters to hadal trenches (Hutchings 2000d; Paterson et al. 2009). There are currently ~ 852 accepted species in 164 genera (Read and Fauchald 2020), some of which appear to be restricted to the deep sea, e.g., species in the subfamily Macellicephalinae (Bonifácio and Menot 2018). Polynoids are one of the dominant epifaunal annelid families in abyssal (> 2000 m) depths (Paterson et al. 2009) but their diversity in Australian waters is poorly known. Records of polynoids from deep water in Australia (> 1000 m) are from McIntosh (1885) who described *Eunoe abyssorum* from 4755 m depth from 800 km southwest of Victoria, as well as *Polynoe ascidioides*, which is now considered as nomen dubium (Read and Fauchald 2020); Benham (1921) described *Parapolyeunoa flynni* as *Hololepidella flynni* from 2379 m off Tasmania; and Augener (1927) described *Lepidasthenia australiensis* as *Nectochaeta australiensis* from 1000 m off the Victorian coast. Averincev (1978) described *Eunoe ivantsovi* from 1640 m from the Lord Howe Rise, *Eunoe papillaris* from 1800 m from the GAB, and *Harmothoe paxtoni* from 1800 m in southern Australian waters, although he also described other more shallowly-collected species, and recorded many polynoid species not previously recorded from deeper waters in the Australian and New Zealand region. More recently, Hanley and Burke (1991) described a new species and genus *Brychionoe karenae* from 1100 m from the Cascade Plateau in the Tasman Sea, and Kirkegaard (1995) described two new species, *Lagisca torbeni* from 1320 m and *Harmothoe australis* from 1340 m, both in the GAB.

From recent sampling voyages by RV ‘Investigator’ to the GAB in 2013–2017, 16 polynoid taxa were distinguished to species level OTUs (MacIntosh et al. 2018), some of which were also found in the 2017 Sampling the ‘Abyss’ voyage (present study material), an additional seven species not previously recorded were also discovered during this latter voyage.

In this study we report at least 11 genera and 15 species, some of which are likely undescribed.

##### Admetella cf. longipedata

Taxon classificationAnimaliaPhyllodocidaPolynoidae

(McIntosh, 1885)

Fig. 22A

###### Diagnosis.

Four long-bodied specimens with < ~ 62 segments, at least 24 pairs of elytrophores, all elytrae missing. Pharynx dark purple, with four plate-like falcigerous jaws, and ringed with 21 or 22 pairs of papillae. Prostomiums all badly degraded. Antennal styles all missing, median antenna ceratophore present, inserted in medial notch on prostomium, dorsal to scars of small lateral antennae inserted anteroterminally, antennal scales missing. Cephalic peaks absent. Palps long and robust, with longitudinal rows of very fine papillae. Facial tubercle present, rounded, located dorsal to ridged upper lip, between palp bases. Eyes absent. Low transverse nuchal fold present between first pair of elytrophores. Tentaculophores with chaetae. Parapodia subbiramous, well developed, flattened transversely, elongated (neuropodium 2 × longer than notopodium), with elongated acicular lobes and long cirriform subacicular processes, neuropodia not deeply split dorsally and ventrally, short notopodia arising from anterodorsal faces of parapodia. Neurochaetae flattened iridescent chaetae with pointed bare tips (often split) with rows of fine serrations along one side. Notochaetae all missing or absent.

###### Remarks.

Because of the poor state of prostomiums, it was not possible to determine the presence of antennal scales or sheaths between ceratophores of median and lateral antenna, which *A. longipedata* possesses. All specimens lacked notochaetae which were assumed to be broken off. In all other features, such as elongate parapodia with long acicular lobes and long cirriform subacicular processes, the position of antennae on prostomium, presence of a nuchal fold, numerous, long, flattened, transparent neurochaetae, > 20 pairs of elytra and > 50 body segments, the specimens resemble *Admetella* and are closer to *A. longipedata* than to *A. brevis* Levenstein, 1978, due to the presence of chaetae on the tentaculophore (see Fauchald 1972). Pettibone’s (1967) illustrations (and part of description) of *A. longipedata* are incorrect according to Fauchald (1972), who regards that description as belonging to *A. hastigerens* Chamberlin, 1919.

###### Records.

4 specimens. Suppl. material 1: op. 35 (AM).

**Figure 22.**
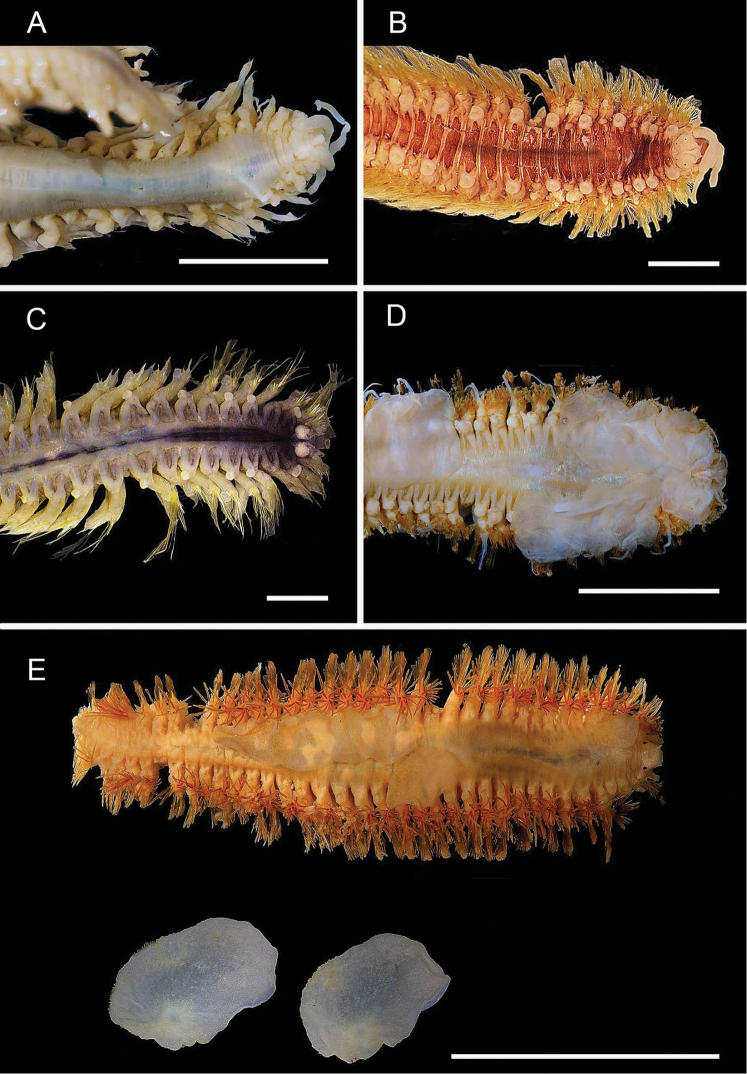
Polynoidae**A**Admetella cf. longipedata anterior end, dorsal view (AM W.51461) **B***Austrolaenilla* sp. anterior end, dorsal view (AM W.52215) **C***Bathyeliasona nigra* anterior end, dorsal view (AM W.52216) **D**Eunoe cf. abyssorum anterior end, dorsal view (AM W.51463) **E**Eunoe cf. opalina dorsal view and elytra (AM W.51464). Scale bars: 1 mm (**A, D, E**); 3 mm (**B**); 2 mm (**C**).

##### Austrolaenilla

Taxon classificationAnimaliaPhyllodocidaPolynoidae

sp.

Fig. 22B

###### Diagnosis.

Two complete specimens (AM W.52215; 1.5 cm long, 0.5 cm wide excluding chaetae; AM W.52016: 7 mm long, 3 mm wide excluding chaetae) with 30–35 segments, 14 or 15 pairs of elytrophores, on 2, 4, 5, 7, 9, 11, 13, 15, 17, 19, 21, 23, 26, 29, 32, all elytra missing. Dorsum with transverse red-brown pigment on every segment to posterior. Head with small cephalic peaks, two pairs of eyes of similar small size, anterior pair situated mediodorsally and level with widest part of prostomium, posterior pair more dorsal. Median antenna with ceratophores inserted in anteromedial notch, style missing, lateral antennae with ceratophores inserted ventral to prostomial lobes, bases touching, with short papillated styles, less than half length of prostomium. Palps long, with rows of small papillae. Tentaculophores with stout notochaetae present. Dorsal cirri present throughout body; dorsal tubercles low, nodular. Parapodia with notopodia shorter than neuropodia, both with elongate acicular lobes, neuropodial acicular lobe with extended papilla-like process. Notochaetae all stout, thicker than neurochaetae, with rows of spines and blunt tips. Neurochaetae all long, fine capillaries, with rows of long slender spines longer than the width of the neurochaeta, and more distally, long fine hairs terminating in a tuft, some with capillary tips and some with truncate tips. No other types of neurochaetae present.

###### Remarks.

The small size of the specimens (30–35 segments, ~ 40+ segments for *Austrolaenilla* species) indicates that they may be juvenile, but the identification is based on the diagnostic feature for the genus: the presence of neurochaetae much slenderer than notochaetae, and with capillary tips that terminate distally in tufts of fine hairs, which these specimens possess. There are ten currently valid *Austrolaenilla* species (Read and Fauchald 2020), some of which have been described and reported from southern Antarctic and New Zealand waters, but we are unwilling to attempt a more specific identification due to the small and probably juvenile nature of the specimens.

###### Records.

2 specimens. Suppl. material 1: ops. 4, 42 (AM).

##### Bathyedithia

Taxon classificationAnimaliaPhyllodocidaPolynoidae

sp.

###### Diagnosis.

Specimen small-bodied, short, with 23 segments and 10 pairs of elytrophores. All elytra missing. Prostomium bilobed, median and lateral antennae absent, frontal filaments absent, facial tubercle absent. Jaws with lateral serrations, pharynx not everted, thus number of terminal papillae not observed. Palps with small palpophores, palps of similar length to tentacular cirri. Tentaculophores achaetous. Notopodia shorter than neuropodia, with elongate acicular lobes. Notochaetae slenderer than neurochaetae; notochaetae with subdistal rows of fine spines, neurochaetae flattened to concave, serrated along both margins. Ventral cirri from segment 3 inserted medially on neuropodia. Nephridial papillae not observed. Ventral keel absent posteriorly.

###### Remarks.

The specimen is damaged and fragile, and most notochaetae and all elytra are missing. Two genera share the characters which distinguish our specimen (10 pairs of elytra, median and lateral antennae absent): *Bathyedithia* and *Polaruschakov*. Both genera also have similar neurochaetae, which are flattened and serrated on both margins. *Bathyedithia* has seven or nine pairs of terminal papillae on the pharynx compared with *Polaruschakov* which has 14 pairs (Pettibone 1976) but we have been unable to observe this character on our single fragile and damaged specimen. On the basis of the presence of lateral serrations of the jaws which were observed (and which are absent from *Polaruschakov* species) we assign this specimen to *Bathyedithia*.

###### Records.

1 specimen. Suppl. material 1: op. 31 (AM).

##### Bathyeliasona nigra

Taxon classificationAnimaliaPhyllodocidaPolynoidae

(Hartman, 1967b)

Fig. 22C

###### Diagnosis.

Specimens short-bodied, complete ones with 18 segments, eight pairs of elytra. Body colouration dark purple-black. Bilobed prostomium tapering anteriorly to subulate frontal filaments (lateral antennae absent). Median antenna on long ceratophore inserted mid-dorsally on prostomium, posterior to frontal filaments. Palps long, tapering, smooth. Tentaculophores with chaetae. Facial tubercle absent. Eyes absent. Dorsal tubercles indistinct/absent, dorsal cirri with papillated styles. Nephridial papillae large, wide, on segments 10–12, but small, cylindrical, on segments 5–9 and 13–18. Parapodia with distally elongate pre-acicular neuro- and notopodial lobes, neuropodial supra-acicular process absent. Notochaetae present, as thick as neurochaetae, stout, with numerous transverse spinous rows and blunt bare tips. Neurochaetae wide, flattened, with serrated lateral margins, tips bluntly pointed, unidentate.

###### Remarks.

Of the four species of *Bathyeliasona* (Pettibone 1976; Bonifácio and Menot 2018), only *Bathyeliasona nigra* has 18 segments, tentacular cirri with smooth styles and notochaetae as thick as neurochaetae. Our material conforms closely with the description of Pettibone (1976).

*Bathyeliasona nigra* was also recorded from the GAB surveys 2013/2015, albeit as ‘Lepidonotinae sp. 2’ (MacIntosh et al. 2018). Most specimens are missing chaetae (broken off), palps and styles of cirri and antenna. This species has been recorded previously from Antarctic waters in depths of ~ 2500 m (Hartman 1967b), as well as from the Indian Ocean, in 3300–4360 m depth (Kirkegaard 1995).

###### Records.

22 specimens. Suppl. material 1: ops. 33, 42, 53, 54, 65, 78, 89 (AM).

##### Bathypolaria magnicirrata

Taxon classificationAnimaliaPhyllodocidaPolynoidae

(Neal, Barnich, Wiklund & Glover, 2012)

###### Diagnosis.

Specimens with short bodies, 18–19 segments, nine pairs of small, reduced elytrophores (all elytra missing). Everted pharynx light brown, with seven pairs of similar-sized terminal papillae, two pairs of smooth amber-coloured jaws. Prostomium wider than long, bilobed, eyes absent, median antenna present on small cylindrical ceratophore, style long, tapering. Lateral antennae and frontal filaments absent. Long smooth palps present, inserted ventrolaterally. Tentacular segment fused to prostomium, tentaculophores achaetous, styles filiform, long, ventral style longer than dorsal style. Cirrophores on non-elytrigerous segments more prominent than elytrophores, large, cylindrical anteriorly. Parapodia biramous, elongate with notopodia almost as long as neuropodia, aciculae penetrating epidermis. Ventral cirri inserted subdistally from segment 3. Notopodia with long flattened, wide chaetae with fine serrations along one side, tips pointed, unidentate. Neurochaetae slenderer than notochaetae, flattened and with fine serrations along both sides of each chaeta. Posterior ventral keel present.

###### Remarks.

*Austropolaria* was originally described as a monotypic genus, based on *A. magnicirrata* described by Neal et al. (2012) from 1000–1500 m in the Amundsen Sea of the Antarctic region. These specimens agree closely with this species diagnosis including that the pharynx has seven pairs of terminal papillae, nine pairs of reduced elytrophores, large dorsal tubercles, and a posterior ventral keel.

Kolbasova et al. (2020) showed that the monotypic genus *Austropolaria* is a junior synonym of *Bathypolaria* Levenstein, 1981 so the combination of the type species *Bathypolaria magnicirrata* (Neal, Barnich, Wiklund & Glover, 2012) was implicit.

###### Records.

8 specimens. Suppl. material 1: ops. 23, 45, 110 (AM).

##### Bruunilla

Taxon classificationAnimaliaPhyllodocidaPolynoidae

sp.

###### Diagnosis.

Specimens small-bodied with 17 segments. All elytra missing, eight pairs of elytrophores present. Prostomium bilobed with small median antennal ceratophore (style missing), frontal filaments present, lateral antennae, eyes and facial tubercle absent. Palps smooth, short. Pair of large lamellate wing-like structures with blunt tips present ventrally, emergent from the lower lip. Tentaculophores achaetous, tentacular styles long. Notopodia reduced, much shorter than neuropodia, both neuropodia and notopodia with elongate acicular lobes. Notochaetae present, slenderer than neurochaetae, both distally flattened to concave with serrations along both sides. Ventral cirri from segment 3 inserted medially on neuropodia; ventral cirri on segment 2 longer than those on following segments.

###### Remarks.

These specimens possess a pair of large wing-like structures on the ventral surface of the lower lip (Bonifácio and Menot 2018: fig. 11B, G), a character so far unique to the genus *Bruunilla*, and specimens most resemble *Bruunilla nealae* Bonifácio & Menot, 2018 because of the blunt tips of these structures. However, because the posterior ends are all somewhat damaged, the presence of cirriform papillae on neuropodia 12–17, a character differentiating this species from *B. natalensis* Hartman, 1971, could not be confirmed. This former species is only known from a single specimen in the equatorial eastern Pacific Ocean, from 2979 m depth.

###### Records.

4 specimens. Suppl. material 1: ops. 79, 134, 110 (AM).

##### Eunoe cf. abyssorum

Taxon classificationAnimaliaPhyllodocidaPolynoidae

(McIntosh, 1885)

Fig. 22D

###### Diagnosis.

Short-bodied, 35–40 segments, 15 pairs of elytra. Elytra pale, with minute conical microtubercles around edges and on posterior half, margins without papillae or fimbriae. Prostomium (violet-coloured when newly preserved, but fading in ethanol) with small cephalic peaks. Eyes absent from most specimens, a few with small subdermal ones. Palps long, smooth. Short lateral antennae ventrally attached (sensu Barnich and Fiege 2009, 2010), bases not touching; median antenna dorsal to lateral antennae, with large ceratophore, inserted in anterodorsal notch. Tentacular segment with notochaetae. Dorsal cirri styles long, some small, sparse papillae present. Parapodia biramous, notopodia and neuropodia both with elongate prechaetal acicular lobes, neuropodial one with supra-acicular digitiform lobe papilla-like, and rounded postchaetal lobe. Notochaetae stout, thicker (but shorter) than neurochaetae; notochaetae with blunt tips and many distinct subdistal rows of spines. Neurochaetal falcigers with long unidentate tips and subdistal rows of short spines.

###### Remarks.

The type locality for *Eunoe abyssorum* McIntosh, 1885 is the GAB, from 4750 m depth. The only other records are by Benham (1921) as ‘*Harmothoe abyssorum*’ (three specimens from the Southern Ocean, south of Australia, in 650 m depth), Benham (1927) from Commonwealth Bay, Antarctica, in 82–91 m, and Knox and Cameron (1998) from McMurdo Sound, Antarctica. However, the descriptions by these authors of this species are inconsistent and may not be of the same species as McIntosh’s.

*Eunoe* includes 46 accepted species, of which at least 8 are known from southern Australia, New Zealand and adjacent regions of the Southern Ocean (Read and Fauchald 2020). Many species are incompletely known due to inconsistent descriptions; thus our species identifications are qualified pending a revision of the genus.

###### Records.

9 specimens. Suppl. material 1: ops. 6, 22, 30, 53 (AM).

##### Eunoe cf. opalina

Taxon classificationAnimaliaPhyllodocidaPolynoidae

McIntosh, 1885

Fig. 22E

###### Diagnosis.

Specimens with 38–42 segments. Elytra 15 pairs, far posterior part not covered by elytra, present on 2, 4, 5, 7, 9, 11, 13, 15, 17, 19, 21, 23, 26, 29, 32; margins with short fine papillae, also longer ones present internally on posterior section, microtubercles cylindrical with truncated flattened tips and few larger macrotubercles (soft, globular and bell-shaped) with similar truncated tips. Some faint small brown pigment spots on prostomium and dorsum. Prostomium ovate, wider than long. Cephalic peaks present, two pairs of eyes with anterior pair laterally at widest part of prostomium, posterior pair lateral, but closer together than anterior pair. Lateral antennae inserted ventrally, bases separate, antennal styles papillate and longer than prostomial width. Tentaculophores with notochaetae present, styles papillated. Dorsal cirri papillated. Noto- and neuropodia with elongate acicular lobes, supra-acicular digitiform process present on neuropodia. Aciculae penetrating epidermis. Notochaetae all spinous, with many rows of spines right up to the blunt tip, neurochaetae thinner with fine spinous rows, all unidentate with bare falcigerous tips. Notochaetal fascicles held erect dorsally but not orientated over dorsum.

###### Remarks.

These specimens most resemble *Eunoe opalina* McIntosh, 1885, because of the combination of unidentate neurochaetae, the form of spination of noto- and neurochaetae, presence of papillae on lateral antennae, presence of notochaetae on tentaculophores, and the forms of elytral ornamentation (papillae, small microtubercles and a few larger soft globular macrotubercles). However, because there are some differences to previous descriptions of *Eunoe opalina* (e.g., presence of papillae on dorsal cirri and presence of chaetae on tentaculophores), the identification remains tentative. *Eunoe opalina* has previously been recorded from the Southern Ocean at depths of 100–500 m.

###### Records.

2 specimens. Suppl. material 1: ops. 6, 22 (AM).

##### Eunoe

Taxon classificationAnimaliaPhyllodocidaPolynoidae

sp. 3

###### Diagnosis.

Specimens with 25–38 segments, 15 pairs of elytra. Some brown pigment present on prostomium and spots on anterior dorsum and ventrum. Elytra with small fine papillae marginally and sub-marginally scattered on surface, conical microtubercles also present, some curved distally, macrotubercles absent. Two pairs of eyes present, sometimes not visible, anterior pair at widest part of prostomium, oriented laterally, posterior pair located more dorsally. Cephalic peaks present. Facial tubercle present. Tentaculophores with several stout curved chaetae. Lateral antennae inserted ventrally, short, approximately half as long as prostomial width, styles papillate, bases almost touching, not fused. Median antenna ceratophore large, style long, papillate. Palps long, at least as long as eight anterior chaetigers, with minute papillae in rows along length. Dorsal cirri long, 1–2 × length of parapodia with chaetae, sparsely papillate. Parapodia long, as long as body width. Notochaetal fascicles held dorsally erect, but not joining mid-dorsally. Neuropodia with preacicular elongate lobe. Notochaetae slightly thicker than neurochaetae, with numerous spinous rows along chaetae, tapering to pointed tip. Neurochaetae of two types: superior ones elongate with numerous rows of small spines alternating along length, tapering to conical (broad) unidentate tips (not hooked); inferior ones shorter, with 6–10 rows short spines starting mid-length, somewhat curved and tapering to fine pointed unidentate tips.

###### Remarks.

These specimens do not exactly agree with any descriptions of the 46 valid species of *Eunoe*, particularly most of those that have been reported from southern Australian, New Zealand and Antarctic waters, i.e., *E. opalina*, *E. abyssorum*, *E. leiotentaculata* Averincev, 1978, *E. papillaris* Averincev, 1978, *E. ivantsovi* Averincev, 1978, *E. iphionoides* McIntosh, 1885, and *E. campbellica* Averincev, 1978. There are differences such as long papillate palps, elytral ornamentation, and the two distinctive types of neurochaetae. The most similar species is *E. etheridgei* Benham, 1915, with which our specimens share features such as type of elytral ornamentation, papillate antennal and dorsal cirri styles, ornamentation of chaetae, and notochaetae thicker than neurochaetae, but which differs from descriptions of *E. etheridgei* by the presence of two types of neurochaetae, and the presence of small papillae on long palps. *Eunoe etheridgei* was recorded from Bass Strait at 360 m. Polynoinae sp. 5 from the GAB surveys in 426–1027 m depth may be the same as *Eunoe* sp. 3 (MacIntosh et al. 2018: additional file 2).

###### Records.

31 specimens. Suppl. material 1: ops. 16, 31, 54 (AM).

##### Harmothoe

Taxon classificationAnimaliaPhyllodocidaPolynoidae

sp. 5

Fig. 23A

###### Diagnosis.

Short-bodied, brown pigment on anterior dorsum, < 32 segments, 15 pairs of elytra (elytra to posterior end). Elytra with microtubercles with numerous points like a crown, and large inflated cylindrical to globular macrotubercles with small mounds, present on lateral and posterior sections of elytra, short papillae present on posterior surface and lateral edges. Prostomium with cephalic peaks, brown spots present on posterior prostomium; two pairs of large eyes on prostomium, anterior pair dorsolateral on widest part of prostomium, posterior pair also lateral. Palps with minute papillae in short rows; lateral antennae short and papillate, attached ventrally with bases slightly separate; median antenna with large ceratophore. Frontal ridge of upper lip without papillae; facial tubercle absent; nuchal flap absent. Tentacular segment with notochaetae. Dorsal cirri styles papillate, with filiform tips, not subdistally inflated. Neuropodia with extended prechaetal acicular lobe and small cirriform supra-acicular lobe. Notopodial lobes low, not extended visibly. Notochaetae in spiky fascicles held vertically but not meeting dorsally, long, thicker than but not longer than neurochaetae, with rows of serrations along one side. Neurochaetae slenderer than notochaetae, bipinnate with rows of teeth and with fine bidentate tips. Single terminal pygidial cirrus present.

###### Remarks.

This species is different to all the GAB*Harmothoe* spp. 1–4, and other *Harmothoe* species reported from Australian waters.

###### Records.

16 specimens. Suppl. material 1: ops. 5, 9, 11, 14, 40, 45, 55, 56, 70, 76, 86 (AM).

**Figure 23.**
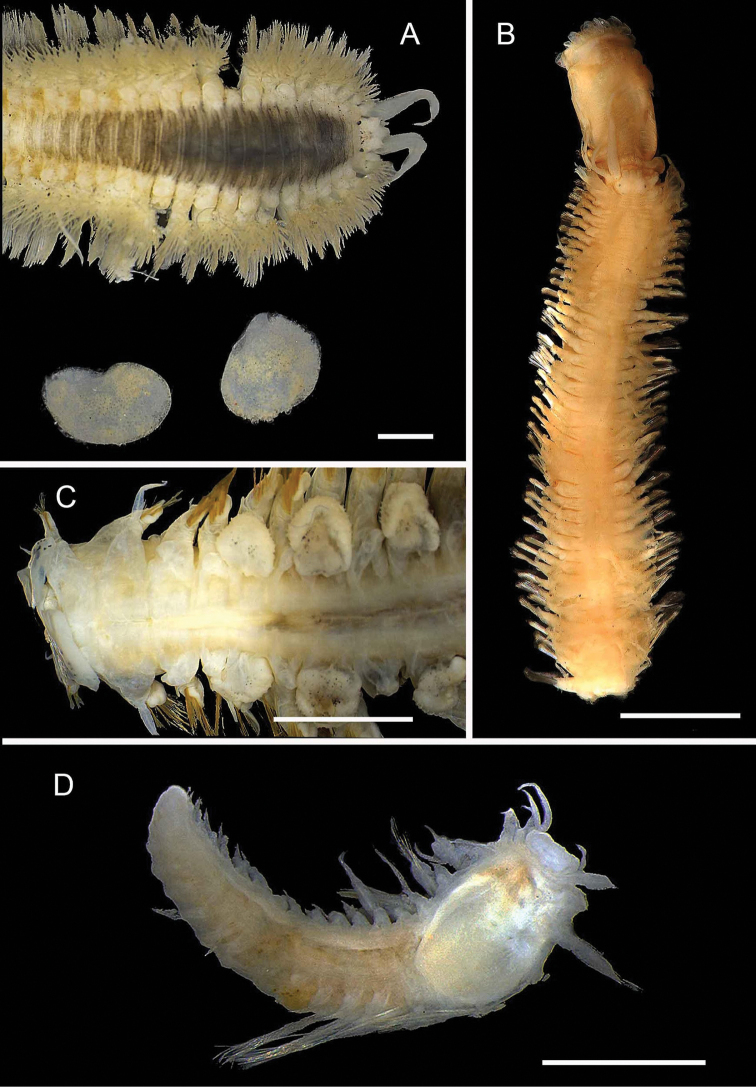
Polynoidae 2 **A***Harmothoe* sp. 5 anterior end, dorsal view, and elytra (AM W.52581) **B***Lepidasthenia* indet. dorsal view, pharynx extended (AM W.51580) **C**Macellicephalinae sp. 5 anterior end, dorsal view (AM W.52014) **D***Polaruschakov* sp. dorsal view (AM W.52580). Scale bars: 5 mm.

##### Harmothoe

Taxon classificationAnimaliaPhyllodocidaPolynoidae

indet.

###### Diagnosis.

Single specimen, broken, with 37 segments, missing elytrae. Fifteen pairs of elytrophores on 2, 4, 5, 7, 9, 11, 13, 15, 17, 19, 21, 23, 26, 29, 32. Palps smooth. Prostomium with prominent cephalic peaks. Two pairs widely spaced large eyes, anterior ones laterally located, posterior ones also laterally placed. Median antenna with large ceratophore, style papillated and longer than palps, lateral antenna short, inserted ventrally, bases separated. Lateral antennae styles papillated, short, approximately half length of prostomium. Prostomium pigmented (brown). Tentacular segment with notochaetae. Dorsal cirri styles papillate, tapering. Notochaetae slightly thicker than neurochaetae. Notochaetal bundles held erect but not meeting dorsally. Notochaetae with rows of spines to tip. Neurochaetae slenderer, with rows of spines, medially inflated, and fine bidentate bare tips (secondary tooth small). Neuropodial acicular lobe with small distal digitiform lobe. Notopodial lobe with elongate acicular lobe.

###### Remarks.

As elytra are all missing, this specimen could not be identified to species. It does not resemble *Harmothoe* sp. 5 of this survey or any of the *Harmothoe* species found in the GAB surveys.

###### Records.

1 specimen. Suppl. material 1: op. 121 (AM).

##### Lepidasthenia

Taxon classificationAnimaliaPhyllodocidaPolynoidae

indet.

Fig. 23B

###### Diagnosis.

Single specimen incomplete with at least 46 segments and 19 pairs of elytra. Elytra thin, colourless, fragile, without macrotubercles, on segments 2, 4, 5, 7, 9, 11, 13, 15, 17, 19, 21, 23, 26, 29, 32, 36, 39, 42, 45+. Nuchal flap absent. Facial tubercle present as small round flap ventral to antennae. Lateral antennae and median antenna terminally inserted. Two pairs of eyes present. Notochaetae absent, notopodia reduced to acicular lobe on neuropodia. Neurochaetae slender and of two types: long, thinner ones with spinous rows and fine blunt tips; additional wider, falcigerous ones with spinous rows subdistally on inflated region and larger tooth below distal tooth (tip) which is bifid or bidentate. Neuropodial prechaetal lobe slightly longer than postchaetal lobes. Papillae present on surface of neuropodium ventral to ventral cirrus.

###### Remarks.

There are 42 species of *Lepidasthenia* worldwide (Read and Fauchald 2020), and several have been reported from Australian waters, but this specimen displays differences such as lack of nuchal flap (cf. *Lepidasthenia michaelseni* Augener, 1913) and lack of notochaetae as well as two types of neurochaetae (cf. *Lepidasthenia australiensis* (Augener, 1927). Further complete material is needed for identification to species.

###### Records.

1 specimen. Suppl. material 1: op. 80 (AM).

##### Macellicephala

Taxon classificationAnimaliaPhyllodocidaPolynoidae

sp. 1

###### Diagnosis.

Small-bodied, < 10 mm in length, with 18 segments, nine pairs elytrophores, on 2, 4, 5, 7, 9, 11, 13, 15, 17. Elytra all missing. Prostomium bilobed, lateral antennae and frontal filaments absent, median antenna elongate with large ceratophore; palps smooth, long, reaching to at least segment 6. Eyes absent, facial tubercle absent. Tentaculophores achaetous, tentacular styles long, smooth. Parapodia sub-biramous; notopodia reduced with elongate acicular lobe and few blunt-tipped notochaetae with many faint rows of low teeth (many specimens with notochaetae missing); neuropodia with elongated acicular lobe and long flattened neurochaetae with acutely pointed straight tips and serrations along both sides. Notochaetae slightly slenderer than neurochaetae. Dorsal tubercles indistinct. Dorsal cirrophores elongate. Ventral cirri attached mid-parapodium from segment 3. Body smooth, without papillae. Posteriorly, ventral keel absent; anus opens dorsally. Pharynx often dark purple, seen through the body wall anteriorly, with two pairs of smooth jaws and nine pairs of terminal papillae.

###### Remarks.

These specimens most resemble *M. laubieri* Reyss, 1971, described from the Mediterranean in 2665 m, because of the combination of species characters such as the long length of palps and tentacular cirri, lack of frontal filaments and facial tubercle, inconspicuous dorsal tubercles, the form of the noto- and neurochaetae, and lack of papillae on the body. However, because of the geographical distance of these specimens from the type locality of *M. laubieri*, we do not assign the name.

###### Records.

14 specimens. Suppl. material 1: ops. 23, 40, 45, 66, 76, 87, 103 (AM).

##### Macellicephalinae

Taxon classificationAnimaliaPhyllodocidaPolynoidae

gen. sp. 1

###### Diagnosis.

Small specimens with < 17 segments and eight pairs of large elytrophores, elytra all missing. Palps long, smooth; median antenna present, long, with short ceratophore, inserted posteriorly on prostomium; lateral antennae and frontal filaments absent. Facial tubercle absent, upper lip trilobed. Tentaculophores with strongly projecting acicular lobes, chaetae missing or absent, and long styles. Dorsal tubercles with large branchial-like cirriform processes, dorsal cirri styles elongate, attached subdistally on notopodia. Ventral cirri inserted medially on neuropodia from segment 3. Parapodia biramous, notopodia subequal to neuropodia. Notochaetae stout, curved and serrate on convex side, neurochaetae slenderer, with distal part flattened and serrated along both lateral margins. Posterior end without ventral keel, rounded. Pharynx not everted, not dissected due to fragility of specimens, thus unknown.

###### Remarks.

There are five genera within the Macellicephalinae that possess branchial-like cirriform dorsal tubercles: *Bathyfauvelia* Pettibone, 1976, *Bathycatalina* Pettibone, 1976, *Bathybahamas* Pettibone, 1985, *Vampiropolynoe* Marcus & Hourdez, 2002, and *Yodanoe* Bonifácio & Menot, 2018. However, only *Bathybahamas* and *Yodanoe* have only eight pairs of elytra. *Yodanoe* possesses notopodia much shorter than neuropodia, whereas these specimens have noto- and neuropodia subequal in length. *Bathybahamas* is a monotypic genus, with *B. charleneae* Pettibone, 1985 described from off the Bahamas at a depth of 2066 m, but it possesses two types of neurochaetae, and 18 body segments, which these specimens do not.

###### Records.

6 specimens. Suppl. material 1: ops. 31, 79, 98 (AM).

##### Macellicephalinae

Taxon classificationAnimaliaPhyllodocidaPolynoidae

gen. sp. 5

Fig. 23C

###### Diagnosis.

Large bodied specimen, complete, 33 mm long, 13 mm wide (including parapodia) for 26 segments. Facial tubercle present. Pharynx not everted, but jaws observed via dissection: two pairs of triangular jaws with four or five teeth per jaw; unknown number of pharyngeal papillae. Prostomium bilobed, lobes rounded anteriorly, and posteriorly, median antenna missing or absent, lateral antennae and frontal filaments absent, eyes absent. Palps smooth, short, with reduced palpophores. Tentacular segment fused to prostomium, with tentaculophores situated lateral to palps, both dorsal and ventral styles similar in length and form to palps, achaetous. Large dorsal papillated swollen structures present on (non-elytrigerous) segments 6, 8, 10, located between dorsum and base of cirrophores, possibly reproductive. Dorsal cirri long, longer than parapodia; ventral cirri on segment 2 larger than following segments, becoming small and filiform from segment 3 to posterior segments, inserted subdistally on neuropodia. Parapodia with notopodia reduced to small elongate acicular lobe on anterior face, with slender notochaetae emerging basally, chaetae with rows of fine spines and filiform tips; neuropodia large with elongate pre-chaetal lobe and rounded postchaetal bract-like lobe, neurochaetae much more stout than notochaetae, golden, lanceolate with rows of spines along two sides. Only six pairs of elytrophores distinct, on segments 2, 4, 5, 7, 9, and 11; thereafter difficult to discern due to swollen dorsal bases of parapodia. Pair of thick elytra present on first chaetiger (segment 2), elongate-reniform, covering dorsum, with some dark pigment spots and small marginal papillae; a single large elytra present on segment 11, thin, translucent, round to oval, without pigment or marginal papillae, not covering dorsum. Dorsal tubercles large, and from segment 12, basal swellings present dorsally on every parapodium, with two pairs of ridges running anteriorly and posteriorly along parapodium from dorsum towards dorsal cirrus; these swollen bases also papillated from segment 21. Ventral keel absent. Pygidium rounded, anus dorsal.

###### Remarks.

Due to the lack of lateral antennae, this specimen is assigned to Macellicephalinae, but does not appear to bear resemblance to any of the 37 currently valid Macellicephalinae genera (Read and Fauchald 2020), due to the combination of the form of the neuropodial lobes and notopodial lobes, lack of branchiae, ridged and swollen dorsal tubercles (similar to those of *Lepidonotopodium* spp. but otherwise dissimilar to that genus), lack of frontal filaments, and the presence of serrated jaws.

###### Records.

1 specimen. Suppl. material 1: op. 79 (AM).

##### Macellicephalinae

Taxon classificationAnimaliaPhyllodocidaPolynoidae

indet.

###### Diagnosis.

Small specimens from 3–8 mm in length, with 12–23 segments, and 6–11 pairs of elytrophores. Most somewhat damaged, thus difficult to identify. Possibly seven morphologically different species. Median antenna present or absent, lateral antennae absent, frontal filaments present or absent, facial tubercle absent or present, palps long or short (one specimen with thick, leaf-shaped palps), tentaculophores achaetous. Parapodia with subequal noto- and neuropodial lobes, or notopodial lobes reduced; notochaetae missing, present or absent, neurochaetae either similar thickness to, more robust than, or thinner than, notochaetae; most neurochaetae distally flattened, concave with rows of serrations along both sides. Dorsal tubercles large, or indistinct. Jaws present; pharynx with terminal papillae. Branchial and reproductive structures absent. Ventral keel absent.

###### Remarks.

Numerous specimens were from Brenke sledge samples, most damaged. Approximately seven species are present.

###### Records.

30 specimens. Suppl. material 1: ops. 16, 23, 31, 33, 40, 54, 79, 87, 89, 96, 110, 134 (AM).

##### Polaruschakov

Taxon classificationAnimaliaPhyllodocidaPolynoidae

sp.

Fig. 23D

###### Diagnosis.

Several small specimens, ~ 5 mm long, 1.5 mm wide, some damaged posteriorly, some with palps and many neuropodia missing, with < 21 segments. All elytra missing, nine pairs of small elytrophores present on segments 2, 4, 5, 7, 9, 11, 13, 15, 17. Pharynx with two pairs of smooth jaws (denticles absent), seven pairs of distal papillae present, none larger than others. Prostomium bilobed, median antenna, lateral antennae and frontal filaments all absent, eyes absent (or unpigmented), tentacular segment with long tentacular cirri, achaetous. Palps short, smooth, reaching only to chaetiger 3 or chaetiger 4. Segment 2 with long ventral (buccal) cirrus similar in length to tentacular cirri. Segment 3 with long dorsal cirrus, subsequent ones (mostly) missing. Ventral cirri inserted medially on neuropodia, shorter than neuroacicular lobes. Parapodia sub-biramous, with elongate preacicular neuropodial lobe, notopodia inserted on anterodorsal face of neuropodia, conical and much shorter than neuropodia, aciculae penetrating epidermis. Notochaetae long, slender with transverse rows of fine spines along shaft and with blunt tips. Neurochaetae all flattened, coarsely serrated along both margins, tips pointed. Last three or four posteriormost chaetigers reduced. Swollen dorsal structures may be present on some specimens.

###### Remarks.

These specimens agree with the emended genus diagnosis by Bonifácio and Menot (2018) for *Polaruschakov* Pettibone, 1976, because of the absence of all antennae combined with smooth jaws (or with a single small secondary marginal tooth) and the absence of flattened scale-like structures on segment 6, but, because of their small size, damaged bodies and posterior ends and missing elytra, they could not be identified to species. There are five species in this genus which has only been reported from deep Arctic waters, off the Mediterranean (in Pettibone 1976) and in abyssal waters of the equatorial eastern Pacific Ocean off Mexico (Bonifácio and Menot 2018).

###### Records.

58 specimens. Suppl. material 1: op. 16, 31, 54, 66, 76, 79, 96, 98, 103, 110, 134 (AM). 2 specimens. Suppl. material 1: op. 16 (NHMUK).

##### Polynoidae

Taxon classificationAnimaliaPhyllodocidaPolynoidae

indet.

###### Remarks.

Specimens were identified to family level only, and some others were unidentifiable due to damage, from Brenke sledge samples.

###### Records.

12 specimens. Suppl. material 1: ops. 9, 11, 16, 41, 55, 65, 119 (AM). 1 specimen. Suppl. material 1: op. 16 (NHMUK).

#### Family Protodrilidae Hatschek, 1888

D. Ramos

Protodrilids are interstitial annelids that possess two anterior palps, but lack parapodia, chaetae and other appendages. The family is composed of 38 species in six genera (Martinez et al. 2015; Read and Fauchald 2020). Protodrilids are described mostly from shallow interstitial environments, although one species was abundant on whale bones at depths of 200–260 m (Sato-Okoshi et al. 2015). There only seven records of the family in Australia, two species from one genus *Protodrilus submersus* von Nordheim, 1989 and *Protodrilus jagersteni* von Nordheim, 1989 (from Lord Howe Island and Lizard Island respectively), and specimens assigned to the genera *Claudrilus*, *Megadrilus* (both Lord Howe Island) and *Meiodrilus* (North East Cay, Saumarez Reef) but without species designations (Martinez et al. 2015). Of these, the deepest was sampled from 15 m. The protodrilid specimens recorded in this study came from a whale fall collected at upper bathyal depths (1000 m), this is the deepest documented occurrence of the family to date.

##### Protodrilus cf. puniceus

Taxon classificationAnimaliaPhyllodocidaProtodrilidae

Sato-Okoshi, Okoshi & Fujiwara, 2015

Fig. 24A, B, C

###### Diagnosis.

Round prostomium with two terminal palps. No eyes. Thick bands of cilia around mouth, continuing ventrally along length of body. Slender filiform body, less than 5 mm long when preserved. Pygidium with two lateral lobes and a median cluster of cilia. Colour in ethanol white.

###### Remarks.

*Protodrilus puniceus* is the only species of *Protodrilus* reported from whale fall communities, all other species are distributed in sandy, intertidal areas (Sato-Okoshi et al. 2015). It was described from a sperm whale carcass deposited at a depth of 219–254 m just off Cape Namomisaki, Kyushu Island, Japan. Observed specimens were similarly collected from a whale fall community but at 1000 m.

###### Records.

> 100 specimens. Suppl. material 1: op. 100 (AM). 12 specimens Suppl. material 1. op. 100 (NHMUK).

**Figure 24.**
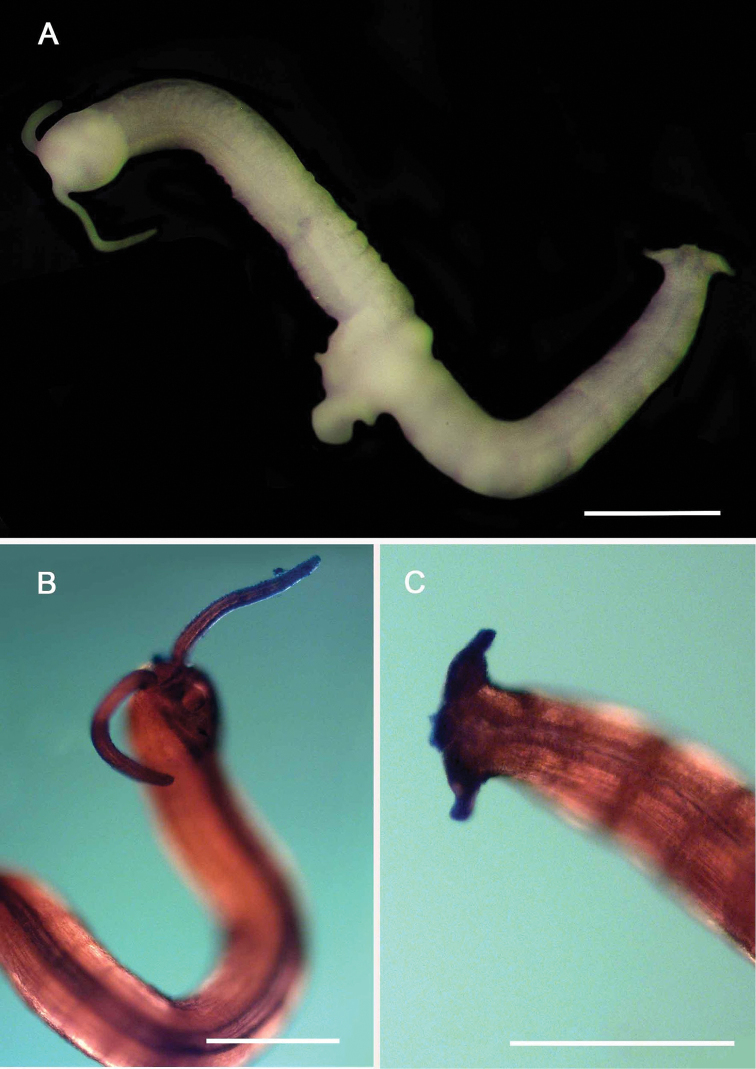
Protodrilidae**A**Protodrilus cf. puniceus**B** same, ventral side **C** same, pygidium. Scale bars: 250 µm.

#### Family Sabellariidae Johnston, 1865

E. K. Kupriyanova, J. Zhang

Sabellariids are filter feeding annelids, which inhabit tubes made of sand and shell fragments cemented together. The family Sabellariidae is composed of 12 genera and 132 species (Read and Fauchald 2020). Most sabellariids live in intertidal or subtidal habitats, sometimes building reefs, thus the common name ‘honey-comb worms’ (Kirtley 1994; Hutchings et al. 2012). The genera *Bathysabellaria*, *Gesaia*, *Phalacrostemma* and *Tetreres* are often found in bathyal and abyssal localities (Lechapt and Kirtley 1996; Hutchings et al. 2012). Five genera and 11 species have been recorded from Australian waters, but only *Phalacrostemma maloga* Hutchings, Capa & Peart, 2012 and *Tetreres terribilis* Hutchings, Capa & Peart, 2012 are known from bathyal depths (Hutchings et al. 2012). Material from the present study was used to describe new species *Gesaia csiro* Zhang, Hutchings, Burghardt & Kupriyanova, 2020 and *Phalacrostemma timoharai* Zhang, Hutchings, Burghardt & Kupriyanova, 2020, while a single specimen of *Phalacrostemma* was too damaged to be formally described as a new species (Capa and Hutchings 2019c; Zhang et al. 2020).

##### Gesaia csiro

Taxon classificationAnimaliaPhyllodocidaSabellariidae

Zhang, Hutchings, Burghardt & Kupriyanova, 2020

Fig. 25A, B

###### Diagnosis.

Operculum completely divided into two elongate free lobes. Twenty-two pairs of outer paleae, their blades with frayed thecae and rolled inward tips. Two pairs of inner opercular paleae on dorsal margin of opercular lobes, with straight cylindrical blades with smooth margins. Six pairs of long conical papillae spirally arranged around opercular lobes. One pair of nuchal hooks without limbation. Three pairs of tentacular filaments along margins of buccal cavity. Buccal flaps absent. One pair of long palps extending beyond operculum. Thoracic segment 1 with neuropodial cirri. Thoracic segment 2 with one pair of triangular lateral lobe. Eleven pairs of dorsal branchiae on chaetigers 2–12. Four parathoracic chaetigers (3–6) bearing notopodia with robust lanceolate chaetae interspersed with fine capillaries and neuropodia bearing thin lanceolate chaetae interspersed with fine capillaries. Cauda long and smooth with three pairs of anal appendages.

###### Remarks.

Type locality is Central Eastern MP, eastern Australia, 4414–4436 m. The genus *Gesaia* is recorded from eastern Australian waters for the first time. Seven specimens of *Gesaia* sp. 1 were recorded from three stations in the GAB (932–1836 m) (MacIntosh et al. 2018: additional file 2), molecular data are needed to confirm if the species in this study are the same as from the GAB.

###### Records.

208 specimens. Suppl. material 1: ops. 88, 89, 128 (AM).

**Figure 25.**
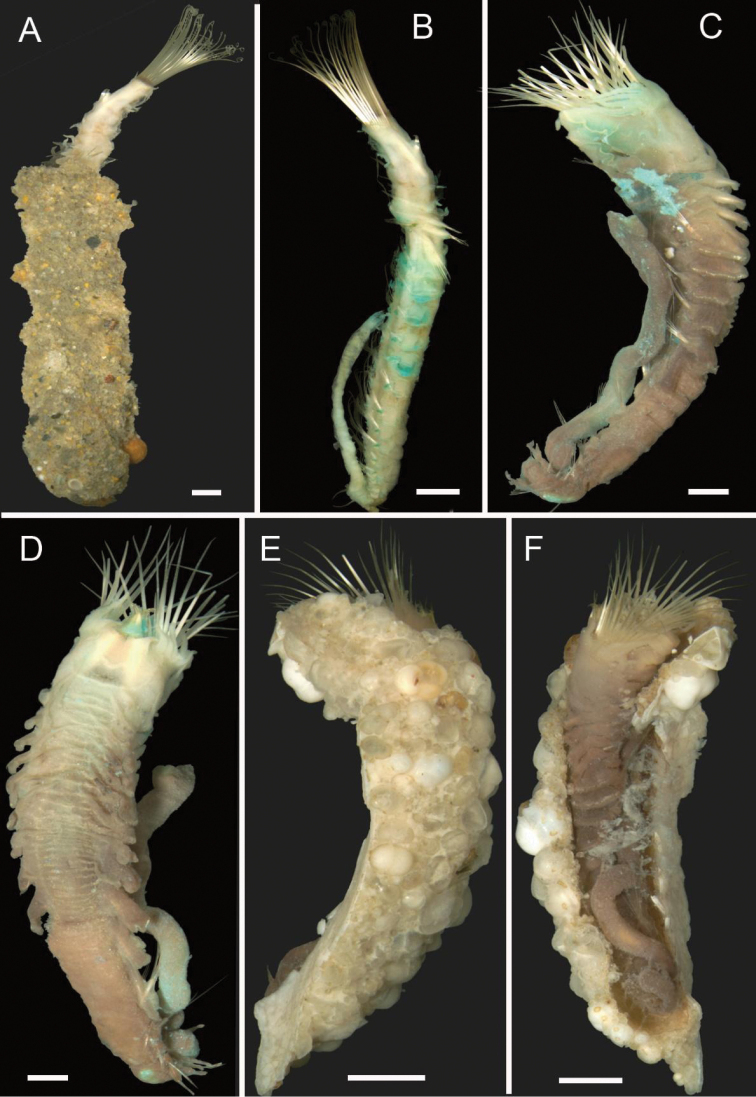
Sabellariidae**A***Gesaia csiro* holotype (AM W.49506), anterior end with tube **B***G. csiro*, entire body, lateral view **C***Phalacrostemma timoharai* holotype (AM W.50674), ventro-lateral view **D***P. timoharai*, lateral view **E***P. timoharai*, tube **F***P. timoharai*, entire body in tube, lateral view. Scale bars: 1 mm (**A, B, E, F**); 0.5 mm (**C, D**).

##### Gesaia

Taxon classificationAnimaliaPhyllodocidaSabellariidae

sp.

###### Remarks.

The specimen is identified to genus, further investigation required to determine if it is the same species as *Gesaia csiro*.

###### Records.

1 specimen. Suppl. material 1: op. 86 (NHMUK).

##### Phalacrostemma timoharai

Taxon classificationAnimaliaPhyllodocidaSabellariidae

Zhang, Hutchings, Burghardt & Kupriyanova, 2020

Fig. 25C–F

###### Diagnosis.

Opercular lobes completely fused to each other. 18–22 pairs of outer paleae, their blades straight with ornamented thecae. Two pairs of inner opercular paleae on dorsal margin of opercular lobes, with straight cylindrical or slightly flattened blades with smooth margins. Eight pairs of robust long conical papillae around operculum. Four pairs of nuchal hooks with limbs on concave margin. Tentacular filaments absent along margins of buccal cavity. Pair of buccal flaps present. One pair of palps similar in length to operculum. Thoracic segment 1 with two (or one) pair of neuropodial cirri. Thoracic segment 2 (chaetiger 2) with one pair of triangular lateral lobes. Nine pairs of dorsal branchiae on chaetigers 2–10. Four parathoracic chaetigers (3–6) bearing notopodia with robust and retractile lanceolate chaetae interspersed with capillaries and neuropodia with fine lanceolate chaetae interspersed with fine capillaries. Cauda smooth, anal appendages absent.

###### Remarks.

Type locality is Coral Sea MP, eastern Australia, 1013–1093 m. *Phalacrostemma timoharai* is characterized by having 18–22 pairs of outer paleae, two pairs of neuropodial cirri on thoracic segment 1 and one pair of lateral lobes on thoracic segment 2.

###### Records.

4 specimens. Suppl. material 1: ops. 104, 121 (AM).

##### Phalacrostemma sp. nov.

Taxon classificationAnimaliaPhyllodocidaSabellariidae

###### Diagnosis.

Opercular lobes completely fused to each other. 12 pairs of (broken) outer golden paleae with pointed tips and compact thecae with straight margins. One pair of inner paleae, their blades smooth, amber-coloured with tapering tips. Eight pairs of robust and tapering opercular papillae, not extending to tip of outer paleae. Two pairs of flattened nuchal hooks, with poorly developed limbs on concave side. Tentacular filaments absent. Pair of buccal flaps present. One pair of short and robust palps, not extending to operculum. Thoracic segment 1 with one pair of long and tapering neuropodial cirri. Thoracic segment 2 with one pair of broad triangular lateral lobes. Eight pairs of dorsal branchiae on chaetigers 2–9. Four parathoracic chaetigers (3–6) bearing notopodia with robust non-retractile lanceolate chaetae interspersed with fine capillaries and neuropodia with thin lanceolate chaetae interspersed with fine short capillaries. Cauda lost.

###### Remarks.

This single specimen from 1761–1770 m is different from the specimens of *P. timoharai* as it has only 12 pairs of outer paleae (but many are broken), only one pair of inner paleae, two pairs of nuchal hooks and non-retractile lanceolate notopodial chaetae on parathoracic segments 3–6 (see Zhang et al. 2020: fig. 10F). The specimen clearly belongs to a new species which was also confirmed by molecular data, but is too damaged to be described formally as a new species.

###### Records.

1 specimen. Suppl. material 1: op. 128 (AM).

#### Family Sabellidae Latreille, 1825

A. Murray

Sabellids are sedentary filter-feeding annelids inhabiting tubes composed of pure mucus or agglutinated sand grains. The family Sabellidae is composed of ~ 40 genera, and > 400 species (Read and Fauchald 2020; Capa et al. 2019b). They are found in many habitats from fresh to marine waters and from intertidal to abyssal depths. Some species belonging to *Chone*, *Euchone*, *Fabrisabella*, *Jasmineira*, *Perkinsiana*, *Potamethus* and *Potaspina* are found more in deep waters in soft sediments (Hartman 1969, 1978; Fauchald 1972; Ruff and Brown 1989; Capa 2007; Tovar-Hernández 2008; Tovar-Hernández et al. 2012; Capa et al. 2013). However, deep-water sabellids are poorly studied in Australian waters, with only a few previously described species reported off eastern Australia by McIntosh (1885) and Benham (1916), and only a few species described from depths > 100 m recently by Capa (2007). Other species have been described from Antarctic waters by various British, German, Swedish and New Zealand expeditions (e.g., Pixell 1913; Hartman 1966b, 1967a, 1978; Hartmann-Schröder and Rosenfeldt 1989, 1991; Knox and Cameron 1998; Tovar-Hernández et al. 2012). In this study we report at least six species from five genera.

##### Sabellidae

Taxon classificationAnimaliaPhyllodocidaSabellidae

gen. sp. 1

Fig. 26A

###### Diagnosis.

Small-bodied species. Several very small specimens (1.5 mm long including crown). Eight thoracic and 8–14 abdominal chaetigers, branchial crown with three pairs of radioles. Radioles with flanges, long pinnules and at least one pair of long ventral radiolar appendages present, dorsal lips present. Posterior peristomial ring collar present, with entire dorsal margin (no mid-dorsal gap), ventrally slightly higher with short mid-ventral incision. Glandular ridge present on chaetiger 2, inconspicuous. Thoracic notochaetae include superior elongate narrowly-hooded chaetae and an inferior row of bayonet chaetae only (paleate chaetae absent). Thoracic uncini acicular with long curving shaft and slight subdistal swelling, with rows of different-sized teeth above main fang, companion chaetae absent. Abdominal neurochaetae narrowly-hooded; abdominal uncini avicular with rasp-shaped teeth above main fang, short neck, quadrangular breast, handle absent. Pygidium without eyespots due to preservation. Pygidial cirrus absent. Anal flanges and depressions absent.

###### Remarks.

Due to small size of specimens, examination of uncinal teeth was difficult, and a positive identification to genus was not able to be confirmed. Specimens were stained with methyl blue and photographed to show staining pattern. Some characters of the genus *Amphicorina* could be observed: eight thoracic and at least eight abdominal chaetigers; three pairs of radioles with few pinnules, long ventral pinnular appendages present; glandular girdle on chaetiger 2; thoracic notochaetae either elongate and narrowly-hooded or small thin bayonet chaetae (with paleate chaetae absent entirely), thoracic uncini acicular with rows of teeth over main fang (though presence of one larger proximal tooth was not observable); abdominal neurochaetae narrowly-hooded, uncini rasp-shaped; posterior anal depression absent. The collar features are, however, more consistent with *Chone* or *Jasmineira*, though the chaetal features are more consistent with *Amphicorina*. However, *Amphicorina* is more typically recorded from shallow and nearshore waters than deep and has not been reported previously from abyssal depths.

###### Records.

6 specimens. Suppl. material 1: ops. 42, 68 (AM).

**Figure 26.**
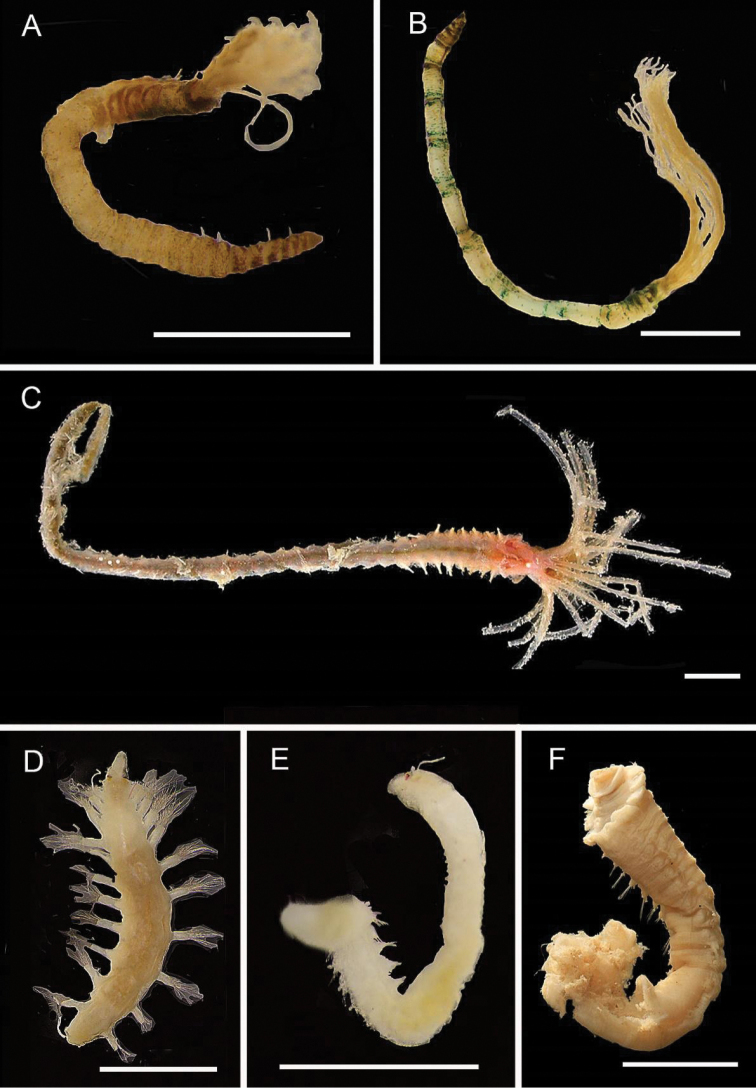
Sabellidae, Syllidae, Terebellidae. Sabellidae**A**Sabellidae sp. 1 (AM W.52133) **B**Sabellidae sp. 2 (AM W.52214) **C**Potamethus cf. scotiae (AM W.51585). Syllidae**D***Anguillosyllis* sp. (AM W.52625) **E**Exogone cf. heterosetosa (AM W.52329) **F**Terebellidae (sensu stricto) (AM W.50426). Scale bars: 1 mm (**A, B, D, E**); 5 mm (**C, F**).

##### Sabellidae

Taxon classificationAnimaliaPhyllodocidaSabellidae

gen. sp. 2

Fig. 26B

###### Diagnosis.

One small gravid specimen, body 0.8 mm long including crown, damaged by poor preservation (plus one half branchial crown without a body). Eight thoracic chaetigers, and seven abdominal chaetigers, 3 (4?) pairs radioles with long pinnules. Branchial crown membrane present, dorsal lip with radiolar appendage, dorsal pinnular appendages present, ventral pinnular appendages present. Anterior peristomial ring dorsally exposed, posterior ring somewhat developed into a higher ventral lobe, with wide mid-dorsal gap. Glandular ridge present on chaetiger 2. Paleate thoracic chaetae apparently absent in segments where chaetae still intact, narrowly-hooded chaetae present (bayonet chaetae not seen). Thoracic uncini acicular, with long curved handles and rows of teeth (sizes not determinable) above main fang. Abdominal uncini avicular with rows of teeth above main fang, breast square or rectangular, handles absent. Posterior anal depression and flanges all absent.

###### Remarks.

This specimen displays some features of the genus *Chone* such as the number of radiole pairs, presence of branchial membrane and pinnular appendages, collar lobation, and the form of the uncini. However, the diagnostic paleate thoracic chaetae are apparently lacking, thus, this is a tentative identification, and may be due to the poor condition and size of the specimen. Specimen was stained with methyl blue and photographed to show staining pattern. Further specimens are required for confirmation.

###### Records.

1 specimen. Suppl. material 1: op. 42 (AM).

##### Jasmineira

Taxon classificationAnimaliaPhyllodocidaSabellidae

sp. 2

###### Diagnosis.

Medium to large body size. Branchial crown with 21 pairs of radioles, lobes involute ventrally. Collar without lateral incisions (not four-lobed), dorsally high with pockets and fused to faecal groove, ventrally high with small midventral incision, lappets small. Dorsal lips with long radiolar appendages, length of ~ 2–3 thoracic segments. Glandular girdle present on chaetiger 2. Thoracic superior notochaetae narrowly-hooded, inferior chaetae paleate with long mucro, bayonet chaetae present. Thoracic uncini long-handled, acicular with sub-distal swelling on shaft, companion chaetae absent. Abdominal neurochaetae elongate, narrowly-hooded; uncini avicular with elongate neck, small breast, short-handled.

###### Remarks.

One large specimen is in three pieces with most radioles split off at abscission zone, but present in sample; another large specimen is too degraded by poor preservation to distinguish anything other than chaetae. These are different to the species found in the 2017 GAB survey (recorded as ‘*Jasmineira* sp.’; MacIntosh et al. 2018: additional file 2), which has a four-lobed collar.

###### Records.

2 specimens. Suppl. material 1: ops 31, 115 (AM).

##### Jasmineira

Taxon classificationAnimaliaPhyllodocidaSabellidae

sp. 3

###### Diagnosis.

One of two specimens badly damaged, thus identification difficult, tube stuck to branchial crown and body which broke during examination. Other specimen very small with all radioles broken off at abscission zone, mouth features undistinguishable, collar low dorsally with deep pockets, fused to faecal groove mid-dorsally, laterally entire, ventrally high with short mid-ventral incision (lappets small). Both specimens with *Jasmineira* features: eight thoracic chaetigers, thoracic uncini acicular and long-handled, companion chaetae absent; thoracic inferior chaetae paleate (though more broadly-hooded type A - but with long mucro of Capa and Murray (2015), as shaft continues through hood to tip), bayonet chaetae present; abdomen with elongate, narrowly-hooded neurochaetae; abdominal uncini avicular with long neck and rows of small teeth over main fang.

###### Remarks.

These specimens are different to ‘*Jasmineira* sp. 2’ from op. 115, which has paleate inferior thoracic chaetae with shorter mucro. They are somewhat similar to specimen (also damaged) found in the 2017 GAB survey, recorded as ‘*Jasmineira* sp.’ (MacIntosh et al. 2018: additional file 2).

###### Records.

2 specimens. Suppl. material 1: op. 40, 78 (AM).

##### Perkinsiana

Taxon classificationAnimaliaPhyllodocidaSabellidae

sp.

###### Diagnosis.

Single small incomplete specimen, only thorax and one abdominal segment remaining. Eight thoracic segments, six pairs of radioles with long pinnules, radiolar eyes absent. Basal flanges absent. Ventral lamellae present and external. First segment enlarged. Ventral shields prominent, in contact with thoracic neuropodial tori. Dorsal lips with short radiolar appendage. Thoracic inferior chaetae two rows of paleate chaetae, bayonet chaetae absent. Thoracic uncini avicular with long necks, medium-length handles and many rows of teeth above main fang. Companion chaetae present, with long, roughly symmetrical hoods. Chaetigers 7–9 with uncini and companion chaetae, spines absent. Abdominal uncini avicular with long necks and short handles, similar to thoracic uncini.

###### Remarks.

Specimen is poorly preserved, the collar region is damaged, and its details are unclear. The specimen conforms most to the genus *Perkinsiana* because of the types of chaetae and the elongation of the first segment but identification to species level is difficult due to lack of abdomen and poor preservation of collar. It is possibly the same species as the larger of two specimens recorded as ‘Sabellidae sp. 3’ in the 2015 GAB survey (MacIntosh et al. 2018: additional file 2), which was examined and although also greatly damaged anteriorly, was found to possess five pairs of long radioles with long pinnules, external ventral lamellae, and thoracic paleate chaetae, long-handled avicular uncini and companion chaetae similar to the specimen described herein.

###### Records.

1 specimen. Suppl. material 1: op.101 (AM).

##### Potamethus cf. scotiae

Taxon classificationAnimaliaPhyllodocidaSabellidae

(Pixell, 1913)

Fig. 26C

###### Diagnosis.

Eight thoracic and numerous abdominal segments. Branchial crown with 6–9 pairs radioles. Wide ventral ‘flange’ on ventralmost radioles; ventral sacs large, external to crown; ventral shields present on body. Collar with dorsal lamellae and dorsal pockets, prolonged ventrally with large ventral lappets, oblique laterally. Peristomial ring elongate, exposed above collar. Thoracic notochaetae including superior narrowly-hooded chaetae and two inferior rows of paleate chaetae; thoracic uncini avicular with extremely long handles (> 10 × distance of main fang to breast); companion chaetae present, with similarly long handles. Abdominal neurochaetae of two types: short, broadly-hooded with long tips and longer elongate narrowly-hooded chaetae; abdominal uncini avicular with long handles/shafts, but with breast reduced to narrow swelling at curvature. Ventral surface glandular, thoracic shields prominent. Tubes muddy with fine transverse striations.

###### Remarks.

Four large fragmented specimens were removed from tubes. There are currently no *Potamethus* species reported from Australia, however, there are museum records of *Potamethus* collected from deep water east of Tasmania in 1986 (Murray, pers. obs.), and more recently from the GAB surveys in 2015 and 2017 (MacIntosh et al. 2018: additional file 2). Worldwide, there are 11 nominal species, all described from deep waters, and types would need to be examined to determine to which species these specimens belong, or if it is new. Based on descriptions from the literature, the specimens bear greatest resemblance to *P. scotiae* (Pixell, 1913) from Antarctic waters.

###### Records.

4 specimens. Suppl. material 1: op. 53, 122 (AM).

##### Potamethus

Taxon classificationAnimaliaPhyllodocidaSabellidae

sp.

###### Remarks.

Identification is to genus only, based on presence of dorsal lamellae joining dorsal collar, and presence of extremely long-handled thoracic uncini and companion chaetae, paleate inferior thoracic chaetae, and broadly-hooded superior thoracic chaetae. Possibly is the same as above specimens of Potamethus cf. scotiae, but specimens were small, incomplete and/or degraded too much from poor preservation whilst in their tubes.

###### Records.

2 specimens. Suppl. material 1: op. 53, 121 (AM).

#### Family Scalibregmatidae Malmgren, 1867

J.A. Blake

Scalibregmatids, sometimes called maggot worms, are characterized by anteriorly swollen, short bodies. They are active burrowers and subsurface deposit feeders, which never form tubes. The family is composed of 16 genera and ~ 72 species (Blake 2015, 2019c; Read and Fauchald 2020). In Australia, six named species in five genera (*Asclerocheilus*, *Hyboscolex*, *Oligobregma*, *Pseudoscalibregma* and *Scalibregma*) have been reported (http://www.ala.org.au). In the present study samples contained at least four genera and six species; at least four new to science. New species include *Asclerocheilus* (one), *Scalibregmides* (one), *Pseudoscalibregma* (one) and *Oligobregma* (one).

##### Asclerocheilus

Taxon classificationAnimaliaPhyllodocidaScalibregmatidae

sp. nov. 1

###### Diagnosis.

Large specimens, heavy yellow spines in noto- and neuropodia of chaetiger 3.

###### Records.

5 specimens. Suppl. material 1: ops. 53, 65 (AM).

##### Oligobregma

Taxon classificationAnimaliaPhyllodocidaScalibregmatidae

sp. nov. 1

Fig. 27D, E

###### Diagnosis.

The most abundant species in the collections. Similar to *Oligobregma mucronata* Blake, 2015, from upper slope depths, Antarctica.

###### Records.

193 specimens. Suppl. material 1: ops. 5, 9, 16, 23, 31, 33, 54, 79, 89, 103, 110 (AM).

**Figure 27.**
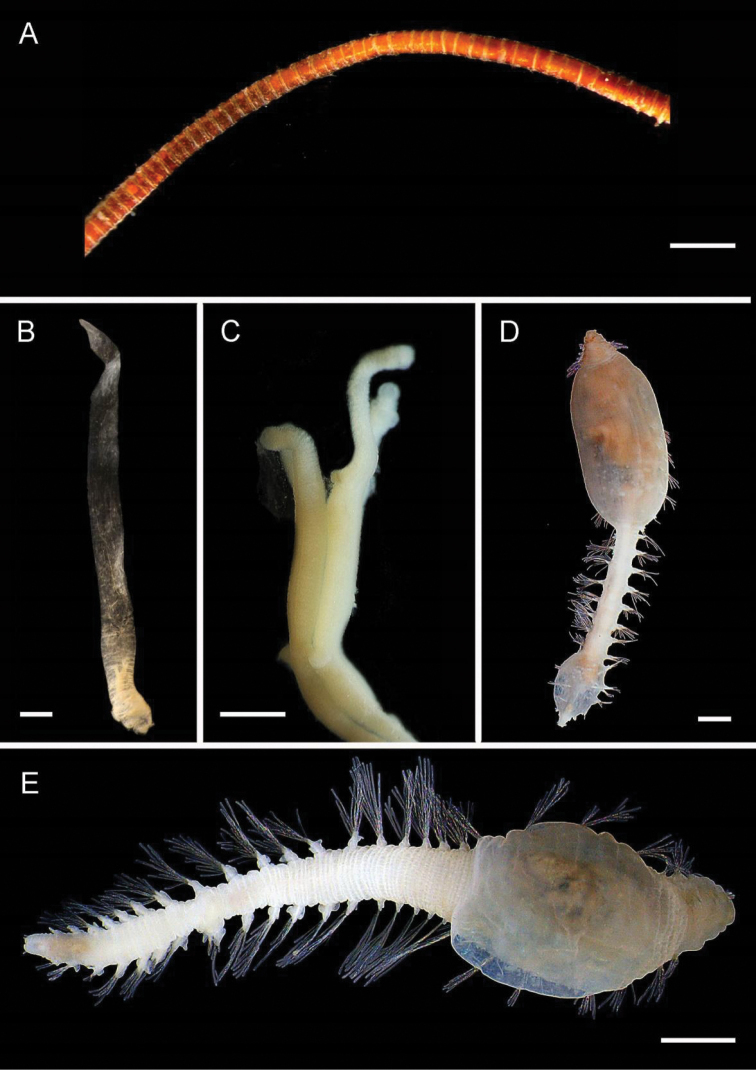
Scalibregmatidae and Siboglinidae. Siboglinidae**A** Frenulate tube (op. 11) **B***Osedax* sp. nov., preserved female specimen inside tube **C***Osedax* sp. nov., detail of palps of preserved female specimen. Scalibregmatidae**D***Oligobregma* sp. nov. 1 (AM W.52686) **E***Oligobregma* sp. nov. 1 (AM W.52698). Scale bars: 2 mm (**A, B**); 500 µm (**C**); 1 mm (**D, E**).

##### Oligobregma spp.

Taxon classificationAnimaliaPhyllodocidaScalibregmatidae

###### Records.

3 specimens. Suppl. material 1: ops. 11, 27 (AM).

##### Pseudoscalibregma

Taxon classificationAnimaliaPhyllodocidaScalibregmatidae

sp. nov. 1

###### Records.

5 specimens. Suppl. material 1: ops. 11, 54, 76 (AM).

##### Scalibregmides

Taxon classificationAnimaliaPhyllodocidaScalibregmatidae

sp. nov. 1

###### Remarks.

Both previously known species of the genus *Scalibregmides* were described from shallow water off South America: *Scalibregmides chilensis* Hartmann-Schröder, 1965 and *Scalibregmides peruanus* Blake, 1981. The current specimen represents the third known species from the genus and the first from deep water.

###### Records.

3 specimens. Suppl. material 1: ops. 11, 33 (AM).

##### Scalibregmatidae gen. spp.

Taxon classificationAnimaliaPhyllodocidaScalibregmatidae

###### Remarks.

Brenke sledge samples were identified to family level only.

###### Records.

27 specimens. Suppl. material 1: ops. 5, 9, 16, 23, 31, 40, 45, 54, 66, 76, 79, 98, 103, 110 (NHMUK). 9 specimens. Suppl. material 1: ops. 9, 22, 32, 96 (AM).

#### Family Serpulidae Rafinesque, 1815

E. K. Kupriyanova

The family Serpulidae (including Spirorbinae) is a group of sedentary annelids inhabiting self-secreted calcareous tubes. The family is composed of ~ 70 genera and > 500 species (Read and Fauchald 2020). These animals are most common and abundant in subtidal and shelf locations, but can occur from intertidal to hadal depths (Kupriyanova et al. 2010; Kupriyanova et al. 2011; Kupriyanova et al. 2014). Serpulids from bathyal and abyssal depths belong to the genera *Bathyvermilia*, *Bathyditrupa*, *Filogranula*, *Hyalopomatus*, *Laminatubus*, *Neovermilia*, *Spirodiscus*, *Protis*, *Vitreotubus* and *Zibrovermilia* (Kupriyanova et al. 2011; Kupriyanova and Ippolitov 2015), but only representatives of *Bathyditrupa*, *Bathyvermilia*, *Hyalopomatus*, and *Protis* are typical abyssal taxa also penetrating into the upper hadal zone (Kupriyanova et al. 2010; Kupriyanova et al. 2011). Two bathyal species, *Laminatubus alvini* ten Hove & Zibrowius, 1986 and *Protis hydrothermica* ten Hove & Zibrowius, 1986 are commonly found in hydrothermal vent and cold seep communities, and Kupriyanova et al. (2010) also reported *Hyalopomatus mironovi* Kupriyanova, 1993a and *Protis* sp. from hydrothermal vents of North Fiji. The shallow-water serpulid fauna of Australia is reasonably well documented, with 45 genera and ~ 180 species recorded from Australian waters, but only four deep-sea species have been recently reported by MacIntosh et al. (2018). In this study > 900 specimens belonging to ~ 13 species were recovered from the Australian lower bathyal and abyssal environment, most of them new to science.

##### Bathyvermilia challengeri

Taxon classificationAnimaliaPhyllodocidaSerpulidae

Zibrowius, 1973

Fig. 28A

###### Diagnosis.

Tubes with characteristic sculpture of numerous transverse ridges close to each other.

###### Remarks.

Only empty tubes were collected. The original records of this species came from three RV ‘Challenger’ stations in the North and South Pacific Ocean taken at 4246–5719 m (Zibrowius 1973).

###### Records.

2 tubes. Suppl. material 1: op. 90 (AM).

**Figure 28.**
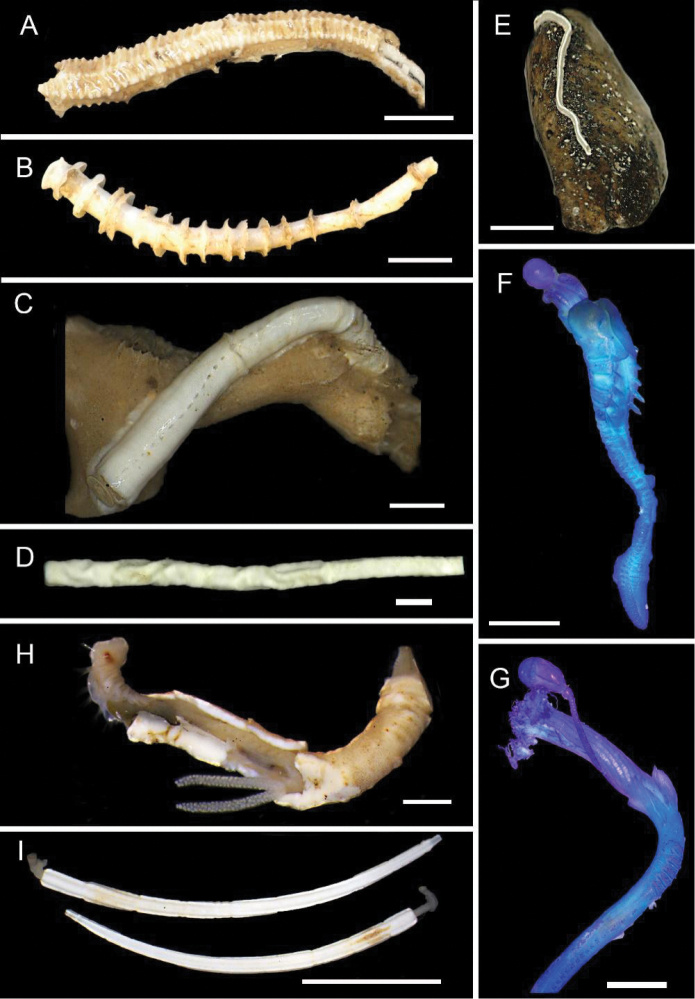
Serpulidae**A***Bathyvermilia challengeri*, tube only (AM W.49722) **B**Bathyvermilia cf. kupriyanovae, tube only (AM W.49707) **C***Bathyvermilia* sp. nov. 3, specimen in white shiny tube (AM W.49494) **D***Hyalopomatus dieteri*, tube only (AM W.49689) **E***Bathyvermilia* sp. nov. 4, specimen in tube with high keel (AM W.49448) **F***Hyalopomatus* sp. nov. 2, specimen removed from the tube (AM W. 49727) **G***Hyalopomatus* sp. nov. 3, specimen removed from the tube (AM W. 49494) **H***Protis* sp. 3, specimen partly removed tube, with egg sacks (AM W.49682) **I***Spirodiscus* sp. nov., specimens in tubes (AM W.49511). Scale bars: 2 mm (**A, C**); 10 mm (**B, E**); 1 mm (**D, F, H**); 0.5 mm (**G**); 2 mm (**I**).

##### Bathyvermilia cf. kupriyanovae

Taxon classificationAnimaliaPhyllodocidaSerpulidae

Bastida-Zavala, 2008

Fig. 28B

###### Diagnosis.

Large white tubes with numerous wide peristomes.

###### Remarks.

Only empty tubes were collected. *Bathyvermilia kupriyanovae* Bastida-Zavala, 2008 and *B. zibrowiusi* Kupriyanova, 1993b both have large tubes with wide flaring peristomes. The other characters separating these two species are structures of thoracic membranes and the opercula. The tubes collected from the Australian eastern abyss are tentatively attributed here to *B. kupriyanovae* as they appear to have wider peristomes than those of *B. zibrowiusi*.

###### Records.

4 tubes. Suppl. material 1: ops. 11, 56 (AM).

##### Bathyvermilia

Taxon classificationAnimaliaPhyllodocidaSerpulidae

sp. nov. 3

Fig. 28C

###### Diagnosis.

White tubes with shiny smooth surface, surface with slight keel made of small denticles and small peristomes. Six thoracic chaetigerous segments plus simple collar chaetae. Opercular peduncle slightly annulated, constriction separates from operculum conical covered with white calcareous endplate.

###### Remarks.

Tubes were mostly found attached to deep-sea corals. SEM and molecular data are needed to confirm this preliminary identification.

###### Records.

21 specimens. Suppl. material 1: ops. 65, 80, 100, 104 (AM).

##### Bathyvermilia

Taxon classificationAnimaliaPhyllodocidaSerpulidae

sp. nov. 4

Fig. 28E

###### Diagnosis.

White tubes attached to substrate throughout their length, triangular in cross-section with high smooth keels. Five thoracic chaetigerous segments plus simple collar chaetae. Peduncle smooth thin, slightly thickened distally, separated by a distinct constriction from elongated conical operculum, covered with distinct brownish flat chitinous endplate. *Apomatus* chaetae present. Abdominal chaetae short, with flat triangular denticulate blade. Uncini with pointed anterior fang.

###### Remarks.

This species differs from other species of *Bathyvermilia* by having only six thoracic chaetigerous segments (which is typical for representatives of the genus *Hyalopomatus*) and triangular in cross-section tube without peristomes. It was the most abundant serpulid collected during the voyage.

###### Records.

661 specimens. Suppl. material 1: ops. 4, 14, 56, 78, 97, 99, 102, 109, 128, 135 (AM).

##### Bathyvermilia

Taxon classificationAnimaliaPhyllodocidaSerpulidae

sp. 5

###### Diagnosis.

Pinkish tubes with rugose surface, with poorly developed transverse ridges. Operculum unknown. Six thoracic chaetigerous segments plus simple collar chaetae. Thoracic membranes ending at chaetiger 3. *Apomatus* chaetae present. Thoracic uncini saw-shaped with pointed anterior fang.

###### Remarks.

Species is distinct because of its pinkish tube.

###### Records.

6 specimens. Suppl. material 1: ops. 88, 97, 99 (AM).

##### Hyalopomatus dieteri

Taxon classificationAnimaliaPhyllodocidaSerpulidae

Kupriyanova & Ippolitov, 2015

Fig. 28D

###### Diagnosis.

Very typical straight thick-walled quadrangular in cross-section tube with rouned edges.

###### Remarks.

Only one empty tube was collected. The species was originally described off New Caledonia, 1820–1980 m.

###### Records.

1 tube. Suppl. material 1: op. 128 (AM).

##### Hyalopomatus

Taxon classificationAnimaliaPhyllodocidaSerpulidae

sp. nov. 2

Fig. 28F

###### Diagnosis.

White smooth tubes completely attached to substrate, without external sculpture, except for several indistinct transverse ridges. Nearly globular, only slightly elongated semi-transparent, undifferentiated operculum. Peduncle of the same width as radioles, smooth, without distinct constriction. Thoracic membranes with rounded edges ending right after chaetiger 2. Collar trilobed, ventral lobe larger than lateral ones, covering radiolar lobes and half of radioles. Five thoracic chaetigers plus collar chaetae bundle including special fin-and-blade chaetae. Uncini rasp-shaped with very characteristic for the genus anterior peg made of two (thoracic) or three or four (abdominal) rounded lobes with shallow incision(s) in between. *Apomatus* chaetae absent. Abdominal chaetae unknown.

###### Records.

1 specimen. Suppl. material 1: op. 100 (AM).

##### Hyalopomatus

Taxon classificationAnimaliaPhyllodocidaSerpulidae

sp. nov. 3

Fig. 28G

###### Diagnosis.

Smooth white tube. Elongated operculum with distinctly differentiated endplate. Peduncle smooth, of same thickness as radioles, annulated distally, separated from operculum by a constriction. Five thoracic chaetigerous segments plus minute simple collar chaetae. Thoracic membranes short, ending at chaetiger 3. Collar high, trilobed, ventral lobe larger than lateral ones. Uncini rasp-shaped with very characteristic for the genus anterior peg made of two or more rounded lobes with shallow incision(s) in between. *Apomatus* chaetae absent. Abdominal chaetae in posterior chaetigers only, capillary with tip made of two rows of denticles.

###### Records.

3 specimens. Suppl. material 1: ops. 86, 97, 104 (AM).

##### Protis

Taxon classificationAnimaliaPhyllodocidaSerpulidae

sp. 1

###### Diagnosis.

Smooth white relatively thick tubes lacking sculpture. Six thoracic chaetigerous segments plus collar chaetae. Operculum, if present, globular transparent on normal pinnulated radioles.

###### Remarks.

SEM and molecular data are needed to confirm this preliminary identification.

###### Records.

48 specimens. Suppl. material 1: ops. 35, 86, 88, 97, 99, 100, 128 (AM).

##### Protis

Taxon classificationAnimaliaPhyllodocidaSerpulidae

sp. 2

###### Diagnosis.

Smooth white relatively thin tubes lacking sculpture. Six thoracic chaetigerous segments plus collar chaetae. Operculum, if present, elongated transparent undifferentiated vesicle on normal pinnulated radioles.

###### Remarks.

SEM and molecular data are needed to confirm this preliminary identification.

###### Records.

22 specimens. Suppl. material 1: ops. 43, 44, 56, 65, 67, 78, 90, 99, 101 (AM).

##### Protis

Taxon classificationAnimaliaPhyllodocidaSerpulidae

sp. 3

Fig. 28H

###### Diagnosis.

Rugose greyish, relatively thick-walled tubes. Six thoracic chaetigerous segments plus collar chaetae. Operculum, if present, globular transparent vesicle on normal pinnulated radioles.

###### Remarks.

One specimen is with two elongated egg sacks attached to the abdomen. SEM and molecular data are needed to confirm this preliminary identification.

###### Records.

10 specimens. Suppl. material 1: ops. 69, 86, 88, 90, 104, 128 (AM).

##### Spirodiscus sp. nov.

Taxon classificationAnimaliaPhyllodocidaSerpulidae

Fig. 28I

###### Diagnosis.

Tube less than 1 cm long, very characteristic, thin tusk-shaped and unattached. Tubes fluted with eight ridges (octagonal in cross-section) anteriorly, but tetragonal in cross-section posteriorly. Pinnulated peduncles, thick relative to normal radioles. Opercula cup-shaped with concave chitinous endplates. Five thoracic chaetigerous segments, including simple collar chaetae. *Apomatus* chaetae absent. Thoracic uncini saw-to-rasp-shaped with wide pegs divided into two lobes, abdominal uncini rasp-shaped. Abdominal chaetae short, with flat triangular denticulate blade.

###### Remarks.

The species is morphologically similar to *Spirodiscus groenlandicus* (McIntosh, 1877) known from the North Atlantic Ocean and Southern Indian Ocean but differs by the tube morphology. Specimens were reasonable common in samples collected by Brenke sledge.

###### Records.

126 specimens. Suppl. material 1: ops. 66, 79, 98, 103, 110 (AM).

##### Serpulidae

Taxon classificationAnimaliaPhyllodocidaSerpulidae

gen. sp.

###### Diagnosis.

Tubes with 4–5 keels and typical transverse sculpture making honey-comb appearance.

###### Remarks.

Tube sculpture slightly resemble that of *Metavermilia arctica* Kupriyanova, 1993c.

###### Records.

3 tubes. Suppl. material 1: op. 100 (AM).

#### Family Siboglinidae Caullery, 1914

M. Georgieva

The siboglinids are highly modified in comparison to other annelids, as they do not have a mouth, gut, or anus, but instead host symbiotic bacteria within a specialised organ known as the trophosome. All siboglinids are also tube-dwelling as adults, with the robustness of the tube made by each species varying among the family. They comprise 32 genera that form four monophyletic lineages, namely the vestimentiferans (21 currently described species), *Sclerolinum* (seven species), *Osedax* (26 species) and the frenulates (143 species) (Read and Fauchald 2020). The siboglinids occur mostly in deep waters, although can occasionally also be found in the photic zone in polar regions (e.g., Southward 1962). Members of the genus *Osedax* gain their nutrition exclusively from vertebrate falls (most commonly whale falls), while vestimentiferans, *Sclerolinum* and frenulates occupy environments such as hydrothermal vents, methane seeps, reducing sediments, sunken plant remains and occasionally also whale falls. With the exception of *Osedax*, siboglinids host chemolithoautotrophic Gammaproteobacteria that fix carbon dioxide, while the Oceanospirillales symbionts of *Osedax* are heterotrophic, and both they and the host depend on vertebrate bones for nutrition (Goffredi et al. 2007). Siboglinids are not well known from Australian waters, however they have been described from surrounding regions such as from off northern New Zealand (vestimentiferans; McCowin et al. 2019), as well as from Indonesia (frenulates; Southward 2000). Frenulates from Australian waters have also been observed in the collections of Australian museums, but these are not yet formally described. There are also additional reports of *Osedax* from off South Australia (G. Rouse pers. comm.), which suggest that at least two of the monophyletic siboglinid lineages are present in Australian waters. We report the presence of Siboglinidae tubes and one species of *Osedax*.

##### Siboglinidae gen. spp.

Taxon classificationAnimaliaPhyllodocidaSiboglinidae

Fig. 27A

###### Remarks.

Frenulate tubes, possibly including live-collected worms, were also collected from seven stations (437–4013 m) in the GAB (MacIntosh et al. 2018: additional file 2).

###### Records.

11 specimens of frenulate tube pieces. Suppl. material 1: op. 11 (NHMUK).

##### Osedax sp. nov.

Taxon classificationAnimaliaPhyllodocidaSiboglinidae

Fig. 27B, C

###### Diagnosis.

Siboglinid found colonising fin whale fall. Female living within tube and with ‘root’ structures embedded into bones. Females with crown of four palps fused for much of their length, without obvious pinnules but with distinct blood vessels in live specimens. Trunk short in relation to the length of the palps, ovisac was not observed. Tube: anterior thin, semi-transparent and appearing closed at the tip, posterior tougher and creased. Colour in ethanol pale yellow.

###### Remarks.

Genetic data confirm that these specimens are a new species that falls within the same clade as other nude palp *Osedax* species (Georgieva et al. in prep) associated with whale falls.

###### Records.

More than 20 specimens. Suppl. material 1: op. 100 (NHMUK) (whale bones, MV).

#### Family Sigalionidae Kinberg, 1856

A. Murray

Sigalionids are a family of scale worms with elongate, narrow bodies and usually with a larger number of segments than in the Polynoidae. There are currently considered to be five subfamilies: Sigalioninae, Pelogeniinae, Pholoinae, Pisioninae, and Sthenelanellinae (Gonzalez et al. 2018; Eibye-Jacobsen et al. 2020), all characterised by the presence of some kind of compound neurochaetae. There are currently ~ 252 accepted species in 32 genera (Eibye-Jacobsen et al. 2020). They occur worldwide from intertidal to depths of < 4000 m (Pettibone 1989; Wehe 2007; Eibye-Jacobsen et al. 2020) but are rarely present in large numbers. Some Sigalioninae and Pholoinae species are exclusive to the deep sea (Gonzalez et al. 2018; Eibye-Jacobsen et al. 2020), particularly those from the genera *Neoleanira*, *Pholoides* and *Pholoe* (Pettibone 1970; Pettibone 1992; Wehe 2007; Ravara and Cunha 2016). In Australia there are 12 genera and 15 named species recorded (http://www.ala.org.au). In this study we report at least five species (two of which are new) from four genera.

##### Leanira sp. nov.

Taxon classificationAnimaliaPhyllodocidaSigalionidae

Fig. 29A, B

###### Diagnosis.

Mostly incomplete specimens, at least 28 mm long, 3 mm wide, for 70 segments (or 26 mm long, 4.5 mm wide for 40 segments). Prostomium sometimes with dark pigmentation or a few spots. Elytra smooth on surface, small and round anteriorly, becoming larger and more ovate (kidney-shaped) posteriorly, almost covering dorsum, lacking lateral indentations. Median antenna short, subulate, without auricles, lateral antennae located on inner dorsal side of tentacular segment. Eyes absent. Palps long, palpal sheaths present. Labial lobes present on lateral lips, bulbous. Dorsal cirri absent from segment 3. Neurochaetae all compound spinigers with entire tips, canaliculate. Clavate stylodes present on parapodia. Branchiae starting at ~ chaetiger 30.

###### Remarks.

This species has also been collected from depths of < 920 m along the east Australia coast during cruises by the FRV ‘Kapala’ (1980) and RV ‘Franklin’ (1988) (Hutchings et al. in prep.). It bears some resemblance to *L. quatrefagesi* Kinberg, 1856 (only recorded from intertidal areas in the South Pacific and Southern Oceans) and *L. hystricis* Ehlers, 1874 (recorded from 900–2600 m in North Atlantic waters) because of the presence of labial lobes, the lack of neurochaetae, the lack of lateral indentations of the elytra and the absence of segmental papillae (Hutchings et al. in prep.).

###### Records.

17 specimens. Suppl. material 1: ops. 22, 23, 40, 45, 54, 56.

**Figure 29.**
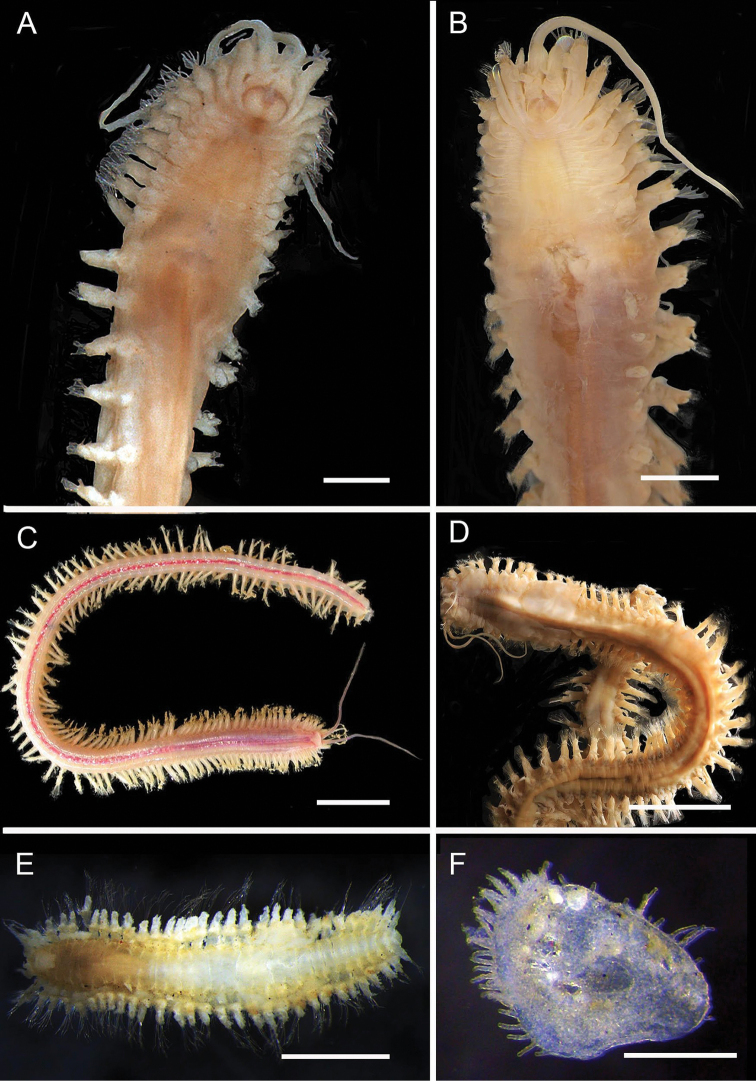
Sigalionidae**A***Leanira* sp. nov. (AM W.52617) **B***Leanira* sp. nov. (AM W.52620) **C***Neoleanira* sp. nov. (AM W.49501) **D***Neoleanira* sp. nov. (AM W.52612) **E***Pholoe* sp. (AM W.52160) **F***Pholoides* sp. (AM W.52616). Scale bars: 1 mm (**A, B, E**); 1 cm (**C, D**); 0.1 mm (**F**).

##### Neoleanira sp. nov.

Taxon classificationAnimaliaPhyllodocidaSigalionidae

Fig. 29C, D

###### Diagnosis.

Eyes absent. Elytra with smooth margins (except for first elytra bearing row of small papillae on anterior margin), lateral ‘pockets’ present. Lateral antennae long, inner tentacular sheath present next to palpal sheath; long dorsal cirri present on segment 3; small auricles on median antenna ceratophore. Neurochaetae all compound canaliculate spinigers. Stylodes present on noto- and neuropodia.

###### Remarks.

This is the same species that has been previously recorded off the east coast of Australia in depths of 815–1075 m, during cruises by the RV ‘Franklin’ (1988) and RV ‘Tangaroa’ (1982) (Hutchings et al. in prep.).

###### Records.

7 specimens. Suppl. material 1: op. 80.

##### Pholoe

Taxon classificationAnimaliaPhyllodocidaSigalionidae

sp.

Fig. 29E

###### Diagnosis.

Many small specimens ≤ 4 mm in length and 1 mm in width, easily fragmented, < 32 chaetigers. Body compressed dorsolaterally. Dorsal cirri and branchiae absent. Prostomium oval, bilobed anteriorly, eyes absent. Median antenna present on ceratophore in anterior notch, small lateral antennae present between median antenna and tentaculophores, slightly ventral to median antenna ceratophore (often hidden). Palps stout, ventrolateral to tentaculophores, at least 2.5 × longer than tentaculophore cirri. Tentaculophores achaetous, with dorsal and ventral cirri, with styles similar to median antenna, digitiform, tapering. Parapodia biramous, notopodia much shorter than neuropodia. Neuropodia with stylodes. Notochaetae thin, capillary, spinulose, curved. Neurochaetae all falcigers with medium-length finely-spinulose blades and with fine serrations subdistally on shafts. Elytra delicate, without concentric rings, with few simple elongate papillae submarginally and on surface, becoming longer on more posterior elytra, elytra covering dorsum.

###### Remarks.

There are currently five described species of *Pholoe* lacking eyes. This species resembles *P. petersenae* Ravara & Cunha, 2016, though that species was described from the NE Atlantic (Gulf of Cadiz) in depths of 1000–2000 m, and also *P. courtneyae* Blake, 1995, which was described from the Californian continental slope in depths of 900–1880 m; these three species are anoculate, and lateral antennae are present, though small and often not visible dorsally.

###### Records.

312 specimens. Suppl. material 1: ops. 9, 16, 23, 31, 33, 40, 42, 45, 54, 55, 66, 79, 89, 96, 98, 110.

##### Pholoides

Taxon classificationAnimaliaPhyllodocidaSigalionidae

sp.

Fig. 29F

###### Diagnosis.

Specimen small, incomplete, 24 chaetigers and only seven pairs of elytra remaining. Elytra with concentric rings, simple marginal and submarginal papillae, and sand grains attached to surface. Single median antenna attached anteriorly on prostomium. Lateral antennae absent. Tentacular segment with single tentacular cirrus, similar to antenna, chaetae present. Two minute pairs of prostomial eyes, each pair very close, but not overlapping. Notochaetae spinulose capillaries; neurochaetae short-bladed compound falcigers with unidentate tips, most with smooth blades and some with smooth shafts, some in anterior chaetigers with faintly serrated longer blades and subdistally serrated shafts. Ventrum papillate.

###### Remarks.

*Pholoides mendeleevi* Averincev, 1978 was described from southern Australian waters in depths of < 730 m, and that author’s illustrations bear some resemblance to this specimen. Other *Pholoides* specimens have also been collected from eastern Australia, mostly in Bass Strait at a depth of 120 m during a cruise by the RV ‘Tangaroa’ in 1981, which may represent a new species (Hutchings et al. in prep.). However, the identification here is tentative as the incomplete specimen cannot be ascribed to either species.

###### Records.

1 specimen. Suppl. material 1: op. 100.

##### Sigalioninae

Taxon classificationAnimaliaPhyllodocidaSigalionidae

sp. 1

###### Diagnosis.

Most specimens incomplete and missing elytra. Largest complete specimen 13 mm long, 1 mm wide, for 68 chaetigers. Prostomium with single long median antenna with short ceratophore, auricles absent. Eyes absent. Lateral antennae absent from all specimens (or missing). Palps long, smooth, reaching to at least chaetiger 14, with short inner palpal sheaths. Tentacular segment fused to prostomium, with two pairs of tapering cirri, dorsal cirri as long as median antenna, ventral ones short; tentaculophores with spinulose capillary chaetae. Labial lobes on lateral lips not observed. Parapodia without long bracts or stylodes. Dorsal cirri absent from chaetiger 3. Branchiae not observed. Notochaetae all capillary, smooth or spinulose, very long posteriorly. Neurochaetae mostly all compound spinigers with canaliculate blades, ventralmost chaetae shorter, canaliculate, and with blunt tips. Most elytra missing, remaining ones small, round, thin, translucent, without marginal or other papillae, not overlapping dorsally. Ventral cirri subulate.

###### Remarks.

These specimens appear to lack lateral antennae completely, and have canaliculate spinigerous compound neurochaetae only, though some in the ventralmost position possess blunt tips. There are few genera of Sigalionidae that lack lateral antennae altogether: *Mayella* Hartmann-Schröder, 1959, known from a single specimen collected intertidally in El Salvador, which Eibye-Jacobsen et al. (2020) suggest is a juvenile polynoid, and most genera and species of Pholoinae, all of which possess short falcigerous neurochaetae. These specimens strongly resemble a sigalionin species because of the type of chaetae. They may represent juveniles of *Leanira* sp. which were present in the same samples.

###### Records.

70 specimens. Suppl. material 1: ops. 9, 16, 23, 31, 40, 42, 45, 54, 55, 76, 79, 96, 110, 134 (AM).

##### Sigalionidae gen. spp.

Taxon classificationAnimaliaPhyllodocidaSigalionidae

###### Remarks.

Specimens from Brenke sledge were identified to family only, specimen from beam trawl were too damaged to identify (op. 80).

###### Records.

5 specimens. Suppl. material 1: op. 16 (NHMUK); op. 80 (1 specimen too damaged to identify, AM).

#### Family Sphaerodoridae Malmgren, 1867

M. Capa

Sphaerodorids are typically benthic annelids that are characterised by the presence of conspicuous epithelial tubercles arranged in more or less distinct rows (longitudinal and/or transverse) and a thick cuticle without collagen (e.g., Ruderman 1911; Reimers 1933; Hausen 2005; Filippova et al. 2010; Capa et al. 2014, 2016; Capa and Bakken 2015). The family includes ~ 110–120 nominal species reported worldwide, from intertidal to abyssal depths (Capa et al. 2014, 2016). The monophyly of the group has been assessed recently and is evidenced by their well-defined external morphology (e.g., Capa et al. 2016). As a result of a major revision of the group, there are currently eight accepted genera (Capa et al. 2018, 2019c): *Clavodorum* Hartman & Fauchald, 1971, *Commensodorum* Fauchald, 1974, *Euritmia* Sardá-Borroy, 1987, *Geminofilum* Capa, Nygren, Parapar, Bakken, Meißner & Moreira, 2019c, *Sphaerephesia* Fauchald, 1972, *Sphaerodoridium* Lützen, 1961, *Sphaerodoropsis* Hartman & Fauchald, 1971 and *Sphaerodorum* Örsted, 1843. Ten species have been reported from Australian waters (Capa and Bakken 2015), most of them were collected in shallow waters (< 80 m), except for *Sphaerodorum australiensis* (Hartmann-Schröder, 1982) reported from around Australia < 400 m deep, *Sphaerephesia longofalcigera* (Capa & Bakken, 2015) collected at ~ 400 m deep, north of Perth, WA, and *Sphaerephesia* sp. (as *Sphaerodoropsis* sp.) collected at ~ 700 m around the Two Rocks region, WA (Capa and Bakken 2015).

##### Clavodorum cf. longipes

Taxon classificationAnimaliaPhyllodocidaSphaerodoridae

Fauchald, 1974

Fig. 30A–C

###### Diagnosis.

Body short and ovoid (~ 2 mm, 22 chaetigers), dorsum strongly convex. Head with seven appendages; palps and lateral antennae digitiform, ~ 6–7 × as long as wide, with two or three digitiform basal papillae each. Median antenna as long as paired appendages, without basal papillae. Tentacular cirri similar in shape to digitiform head additional papillae. Antenniform papillae absent. Macrotubercles stalked, smooth, without terminal papilla; arranged in more or less clear longitudinal rows, one transverse row per segment, with six macrotubercles each. Additional dorsal papillae absent. Ventrum with 4–6 papillae per segment, arranged in two longitudinal bands near the base of parapodia. Parapodia with conical ventral cirri, not surpassing the tip of acicular lobe; lacking parapodial papillae. All chaetae compound, with blades 5–9 × as long as wide in mid-body segments.

###### Records.

3 specimens. Suppl. material 1: ops. 42, 45 (AM).

**Figure 30.**
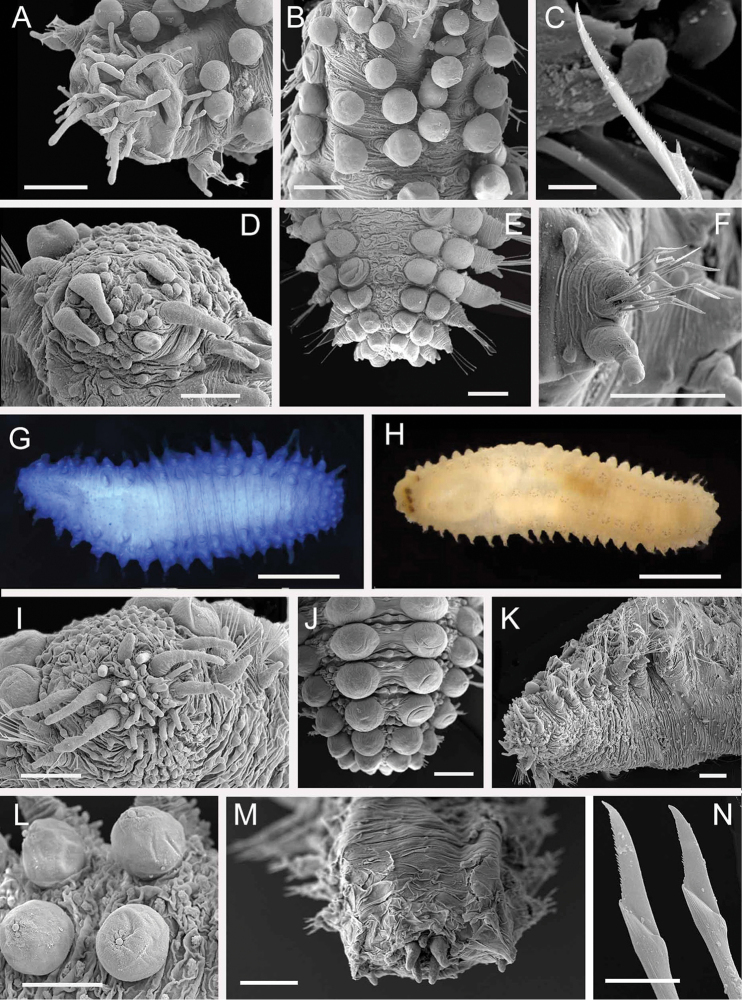
Sphaerodoridae**A–C**Clavodorum cf. longipes**D–G***Sphaerephesia* sp. nov. 1 (**D**, stained with methyl blue) **H–J***Sphaerephesia* sp. nov. 3 **K, L***Sphaerephesia* sp. nov. 5 **M, N***Sphaerodorum* sp. Scale bars: 100 µm (**A, B, D, E, I–M**); 1 mm (**G, H**); 10 µm (**C, N**).

##### Geminofilum

Taxon classificationAnimaliaPhyllodocidaSphaerodoridae

sp. 1

###### Diagnosis.

Body short (1.5 mm), sub-cylindrical, strongly converse dorsally (sub-circular in cross section), with a dark purple-brown pigment in preserved specimen, and whitish macrotubercles. Head invaginated, appendices not observed. Macrotubercles sessile, hemispherical, arranged in two transverse rows per segment, with eight and nine tubercles each. Additional dorsal epithelial papillae absent. Scarce ventral papillae in mid-body segments, but not clearly observed. Parapodia with cylindrical ventral cirri, reaching the tip of acicular lobe. One spherical parapodial papillae, at the base of parapodia (?). All chaetae compound (five or six per parapodium), with blades 4–5 × as long as wide in mid-body segments.

###### Remarks.

This is a possible new species, but material is too damaged to confirm.

###### Records.

1 specimen. Suppl. material 1: op. 45 (AM).

##### Geminofilum

Taxon classificationAnimaliaPhyllodocidaSphaerodoridae

sp. nov. 2

###### Diagnosis.

Body elongated (6 mm, 30 chaetigers), sub-cylindrical, strongly converse dorsally (sub-circular in cross section); lacking any pigmentation pattern (preserved specimen). Head with at least four digitiform appendages, ~ 6 × as long as wide, without spurs or basal papillae; antenniform papillae not distinguished. Small spherical, sessile and smooth tubercles, scattered over body surface (in four irregular transverse rows, and > 40 per segment, transverse rows above parapodia with ~ 14 larger tubercles). Ventrum with ~ four irregular transverse rows of papillae. Parapodia with ventral cirri, surpassing the tip of acicular lobe and ~ eight papillae. Approximately ten compound chaetae per parapodium, with blades 5–6 × as long as wide in mid-body segments.

###### Records.

1 specimen. Suppl. material 1: op. 87 (AM).

##### Sphaerephesia

Taxon classificationAnimaliaPhyllodocidaSphaerodoridae

sp. nov. 1

Fig. 30D–G

###### Diagnosis.

Body ellipsoid (~ 3–5 mm, 15–20 chaetigers), flattened dorsoventrally wider than high; some preserved specimens with yellowish macrotubercles. Head with seven appendages, smooth, lacking basal papillae or spurs; paired appendages ~ 3 × as long as wide, bottle-shaped; median antenna slightly smaller. Antenniform papillae absent. Four longitudinal rows of dorsal macrotubercles, lateral rows closer to each other, one transverse row per segment. Macrotubercles sessile, hemispherical, and with a pointy distal end. Additional dorsal papillae hemispherical, ~ 15 per segment, arranged in four irregular transverse rows. Ventral papillae ~ 20 in mid-segments, arranged in four transverse rows. Parapodia with digitiform ventral cirri, reaching the tip of acicular lobe. Two or three spherical parapodial papillae. All chaetae compound, with blades 4–5 × as long as wide in mid-body segments.

###### Records.

51 specimens. Suppl. material 1: ops. 9, 16, 31, 33, 40, 45, 55; 66, 76, 79 (AM).

##### Sphaerephesia

Taxon classificationAnimaliaPhyllodocidaSphaerodoridae

sp. nov. 3

Fig. 30H–J

###### Diagnosis.

Body ellipsoid (~ 1–5 mm, 20–28 chaetigers), flattened dorsoventrally wider than high. Some preserved specimens with small dark pigment spots in dorsal macrotubercles. Head with seven appendages, smooth, lacking basal papillae or spurs; ~ 5 × as long as wide, bottle shaped; median antenna slightly smaller. Antenniform papillae present, shorter and thinner than median antennae. Four longitudinal rows of dorsal macrotubercles, one transverse row per segment. Macrotubercles sessile, pear-shaped. Additional dorsal papillae hemispherical, ~ 40 per segment, arranged in ~ four irregular transverse rows in mid-body segments. Ventral papillae ~ 20–30 in mid-body segments, arranged in more or less clear transverse rows. Parapodia stout, with prominent acicular lobe and bottle-shaped ventral cirri, reaching the tip of acicular lobe. more than ten parapodial papillae, spherical. All chaetae compound (> ten), with blades ~ 7–10 × as long as wide in mid-body segments.

###### Records.

30 specimens. Suppl. material 1: ops. 9, 16, 23, 31, 33, 42, 45, 46, 54, 55, 79 (AM).

##### Sphaerephesia

Taxon classificationAnimaliaPhyllodocidaSphaerodoridae

sp. nov. 5

Fig. 30K, L

###### Diagnosis.

Body ellipsoid (3 mm, 23 chaetigers), with convex dorsum. Head with seven appendages, conical, smooth, lacking basal papillae or spurs; ~ 3–4 × as long as wide; median antenna shorter, digitiform. Antenniform papillae present; additional digitiform papillae covering the head. Four longitudinal rows of dorsal macrotubercles, one transverse row per segment; lateral rows closer to each other. Macrotubercles sessile, pear-shaped and with terminal papillae. Additional ellipsoid dorsal papillae, ~ 20 per between dorsal most macrotubercles, arranged in four or five irregular transverse rows. Ventral papillae, ~ 40 in mid-body segments, arranged in four or five irregular transverse rows. Parapodia with conical ventral cirri, not surpassing the tip of acicular lobe; and > 20 spherical parapodial papillae. All chaetae (> ten) compound, with blades > 15 × longer than wide in mid-body segments.

###### Re﻿marks.

Identification as ‘sp. nov.’ is tentative, further analysis is needed to confirm.

###### Records.

2 specimens. Suppl. material 1: op. 42 (AM).

##### Sphaerodorum

Taxon classificationAnimaliaPhyllodocidaSphaerodoridae

sp.

Fig. 30M, N

###### Diagnosis.

Body long and slender, subquadrangular in cross section. Head with seven appendages, smooth, lacking basal papillae or spurs; ~ 3 × as long as wide; median antenna and tentacular cirri shorter. Antenniform papillae absent. Two longitudinal rows of dorsal macrotubercles, one pair per segment; sessile, with terminal papillae. Two longitudinal rows of microtubercles, one pair per segment, running parallel between macrotubercles. Additional dorsal papillae faint in studied material. Ventral papillae not observed. Parapodia with less than six spherical papillae. All chaetae semi-compound with blades ~ 5 × as long as wide, in mid-body chaetigers; hooks in first chaetiger not observed.

###### Records.

2 specimens. Suppl. material 1: op 9, 16 (AM).

##### Sphaerodoridae gen. spp.

Taxon classificationAnimaliaPhyllodocidaSphaerodoridae

###### Remarks.

Brenke sledge samples were identified to family level.

###### Reco﻿rds.

3 specimens. Suppl. material 1: op. 16 (NHMUK). 6 specimens. Suppl. material 1: ops. 9, 16, 100 (AM).

#### Family Spionidae Grube, 1850

K. Meißner

Spionidae are benthic annelids which possess a pair of elongate, prehensile grooved palps extending from the head. Spionidae is a large group of ~ 600 species grouped into 38 genera (Blake et al. 2019; Read and Fauchald 2020). The taxonomy of Spionidae from shallow waters around Australia is well studied although the fauna from less accessible regions is not well represented. Spionidae are common in all benthic marine habitats from the intertidal to the deep waters. Spionid genera typically, but not exclusively, reported from the deep sea are *Prionospio* (and related taxa), *Laonice* and *Spiophanes*. In Australian deep waters (> 200 m) seven deep-sea species, *Laonice insolita* Greaves, Meißner & Wilson, 2011, *Laonice pectinata* Greaves, Meißner & Wilson, 2011, *Paraprionospio coora* Wilson, 1990, *Paraprionospio oceanensis* Yokoyama, 2007, *Spiophanes dubitalis* Meißner & Hutchings, 2003, *Spiophanes japonicum* Imajima, 1991 and *Spiophanes wigleyi* Pettibone, 1962 have been reported. The deepest record of a spionid (Spionidae sp.) in Australia was at 4799 m from the Indian Ocean off Geraldton-Exmouth coast (http://www.ala.org.au). Spionidae were abundant and diverse in the more extensive infaunal samples taken in the GAB (MacIntosh et al. 2018: additional file 2). In those voyages 13 OTUs from 43 stations (138–3064 m) were recorded, with *Microspio* and *Prionospio* being the best represented genera. Here we report at least nine species from six genera. At least one species is new to science.

##### Aurospio cf. dibranchiata

Taxon classificationAnimaliaPhyllodocidaSpionidae

Maciolek, 1981

Fig. 31A

###### Diagnosis.

Prostomium round anteriorly, elongated posteriorly (keel), extending to middle or the end of chaetiger 1, without appendages. Prostomial peaks and eyes absent. Peristomium fused to first chaetiger, with golden pigments dorsally along posterior margin of the prostomium. Dorsal crests and interparapodial pouches absent. Cirriform branchiae on chaetigers 3 and 4, small, often partially hidden by parapodial dorsal lamellae to which they are fused basally. Dorsal lamellae large and foliaceous from chaetigers 2–6, smaller and round thereafter. Particularly long capillaries present in anterior chaetigers. Multidentate long-shafted hooded hooks present in noto- and neuropodia, in neuropodia starting on chaetigers 10, much later in notopodia according to original description. Sabre chaetae from chaetiger 10.

###### Remarks.

Diagnostic characters are not consistently observable in all specimens due to their poor condition (all anterior fragments, longest anterior fragment with 19 chaetigers). Notopodial hooks were not present in examined specimens (all short anterior fragments). In some specimens a few branchiae were still present and the start of sabre chaetae and neuropodial hooks on chaetigers 10 could be observed. We here tentatively identify the examined specimens as Aurospio cf. dibranchiata Maciolek, 1981. The species is known to occur in deep waters of the Atlantic and central Pacific Oceans, but has not been reported yet from near Australia.

###### Records.

8 specimens. Suppl. material 1: ops. 16, 27, 31, 42, 79 (AM). 10 specimens. Suppl. material 1: ops. 23, 27, 31, 76 (NHMUK).

**Figure 31.**
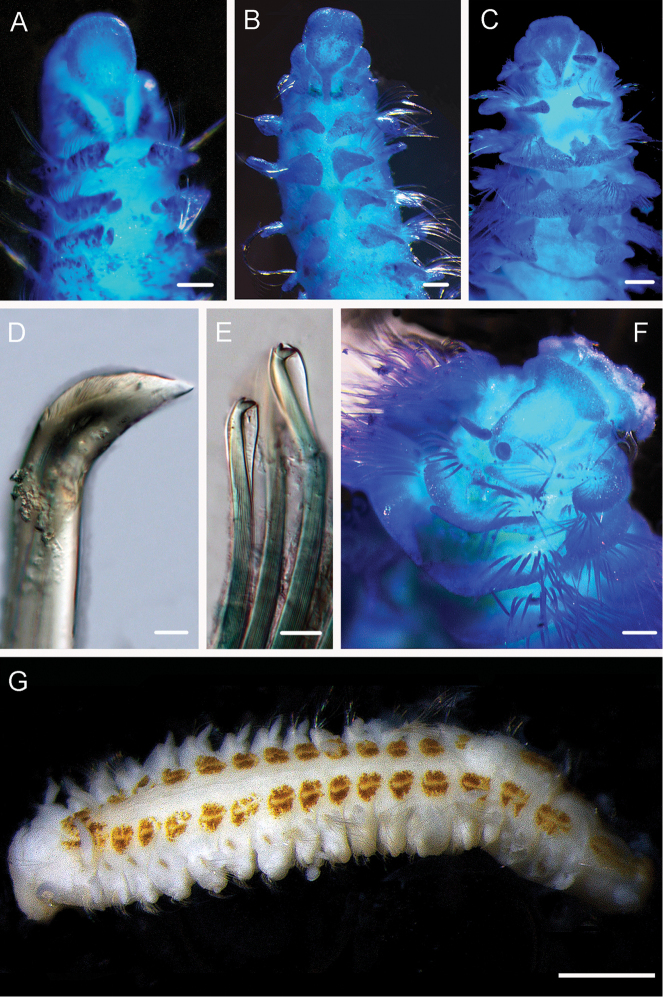
Spionidae**A**Aurospio cf. dibranchiata: anterior end, dorsal view (AM W.52242) **B***Aurospio* sp. nov. 1, anterior end, dorsal view (AM W.52240) **C**Prionospio cf. amarsupiata, anterior end, dorsal view (AM W.52221) **D***Dipolydora notialis*, heavy spine with crest of bristles on convex side from chaetiger 5 (AM W.52245) **E***Dipolydora notialis*, bidentate hooded hooks with smooth, curved shafts without constriction from posterior chaetiger (AM W.52245) **F**Laonice cf. blakei: anterior end, dorsal-oblique view (AM W.52226) **G***Spiophanes anoculata*, posteriorly incomplete specimen in dorsal-oblique view, metameric dorsal ciliated organ bordered by pigment of dark orange or ochre colour (AM W.52222). Scale bars: 100 µm (**A, B, C**); 1 µm (**D, E**); 200 µm (**F**); 500 µm (**G**).

##### Aurospio

Taxon classificationAnimaliaPhyllodocidaSpionidae

sp. nov. 1

Fig. 31B

###### Diagnosis.

Prostomium round anteriorly, extending into a short caruncle to the end of chaetiger 1, without appendages. Eyes absent. Peristomium moderately developed and separated from first segment. First chaetiger dorsally with yellowish pigment lateral to the caruncle. Branchiae present on chaetigers (2, potential branchial scars) 3 and 4, club-shaped to cirriform, smaller than notopodial lamellae and not fused to it. Dorsal crests and interparapodial pouches absent. Parapodial lamellae on chaetiger 1 small, tapered in notopodia, rounded in neuropodia; from chaetigers 2–6 notopodial lamellae large, subtriangular and foliaceous, smaller and rounded thereafter; neuropodial lamellae at same chaetigers wide and foliaceous, thereafter low, wider than long, rounded. Long capillaries present in anterior chaetigers; multidentate long-shafted hooded hooks from chaetigers 15 in neuropodia, apical teeth in a row above main fang. Notopodial hooks not present. Sabre chaetae first present together with neuropodial hooks from chaetiger 15.

###### Remarks.

The specimen is an anterior fragment in rather poor condition. It might belong to an undescribed species of *Aurospio* with neuropodial hooks and sabre chaetae from chaetiger 15.

###### Records.

1 specimen. Suppl. material 1: op. 79 (AM).

##### Prionospio spp.

Taxon classificationAnimaliaPhyllodocidaSpionidae

###### Remarks.

Specimens are identified as *Prionospio* based on the shape of the prostomium (anteriorly rounded, posteriorly extending into a short caruncle), presence of branchiae or branchial scars not earlier than chaetiger 2, and the presence of low dorsal crests. Moreover, sabre chaetae and hooded hooks were usually present. However, most specimens are in poor condition and characters essential for their identification to species level are lost, e.g., mostly anterior fragments, only few branchiae preserved. Other characters observed are very variable. Several species are present according to observed characters (see Suppl. material 2) but remain unidentified.

###### Records.

8 specimens. Suppl. material 1: ops. 33, 40, 79, 110 (AM).

##### Prionospio cf. amarsupiata

Taxon classificationAnimaliaPhyllodocidaSpionidae

Neal & Altamira in Paterson, Neal, Altamira, Soto, Smith, Menot, Billett, Cunha, Marchais-Laguionie and Glover 2016

Fig. 31C

###### Diagnosis.

Prostomium longer than wide, inverse bottle-shaped, slightly rounded anteriorly, elongated posteriorly into short caruncle extending to the end of chaetiger 1, prostomium without appendages. Eyes absent. Peristomium moderately developed and separated from first chaetiger; with yellow pigment lateral to the caruncle as semi-circular ridges (possibly position of nuchal organs). Branchiae mostly lost but scars of lost branchiae seem apparent on chaetigers 2–4, potentially also chaetigers 5 with branchial scars; branchiae on chaetigers 3 and 4 cirriform, shorter than notopodial lamellae and not fused to it. Interparapodial pouches not observed. Notopodial lamellae on chaetigers 1–5 lanceolate, small on chaetigers 1, afterwards increasing in size until chaetiger 4, smaller again on chaetigers 5, largest usually on chaetigers 3 and 4. Neuropodial lamellae small and rounded on first chaetiger, from chaetiger 2 semi-circular, in hook-bearing chaetigers reduced in size and not well preserved in examined material. Chaetae of three types: capillaries, hooded hooks, sabre chaetae. Anterior chaetae until chaetiger 18–20 all capillaries with thin sheaths, in notopodia arranged in up to three rows, in two rows in neuropodia. From chaetiger 18–20 stout granulated sabre chaetae in inferior position. From chaetigers 19 or 20 capillaries without sheaths, and neuropodial hooks; hooks long-shafted, hooded, stout, with ~ seven apical teeth above main fang, sometimes appearing acicular with distally bent tip. Notopodial hooks not present in examined material (all anterior fragments of fewer than 30 chaetigers).

###### Remarks.

We refer to our specimens as P. cf. amarsupiata since branchial scars on chaetigers 2–5 seem to be present. Specimens with long branchiae on 2^nd^ and 5^th^ chaetigers, as shown in the original description, could not be found.

###### Records.

4 specimens. Suppl. material 1: ops. 23, 33, 54, 66 (AM).

##### Dipolydora notialis

Taxon classificationAnimaliaPhyllodocidaSpionidae

(Blake & Kudenov, 1978)

Fig. 31D, E

###### Diagnosis.

Specimens all short anterior fragments, moderately preserved. Prostomium narrow, rounded anteriorly, caruncle not well preserved in present material; occipital tentacle not observed. Chaetiger 1 with capillaries in noto- and neuropodia. Chaetiger 5 moderately modified; modified heavy spines of one type arranged in a curved row, heavy spines with bent tip and crest of bristles on convex side, arranged together with thin companion chaetae; dorsal fascicle of geniculate chaetae and of neuropodial capillaries present. Bidentate hooded hooks with smooth, curved shafts without constriction start in neuropodia of chaetiger 7. Branchiae from chaetiger 7, continuing to the end of fragments. Gizzard-like structure in anterior part of the digestive tract not very distinct.

###### Remarks.

The morphology of specimens examined is generally in good accordance with the original description of *Polydora notialis* by Blake and Kudenov (1978), now referred to *Dipolydora*. The number of heavy spines is greater in the here examined specimens (eight spines in one row opposed to four or five spines cited in the original description). However, this character is not regarded an important diagnostic character in the taxonomic literature dealing with this species or genus.

###### Records.

7 specimens. Suppl. material 1: ops. 4, 67, 70, 80, 100 (AM).

##### Laonice cf. blakei

Taxon classificationAnimaliaPhyllodocidaSpionidae

Sikorski & Jirkov in Sikorski, Jirkov and Tzetlin 1988

Fig. 31F

###### Diagnosis.

Specimens all anterior fragments, almost all in very poor condition, usually very short, only two specimens with > 20 chaetigers. Prostomium bell-shaped, anteriorly broadly rounded; small cirriform occipital tentacle present at posterior end; eyes absent. Nuchal organ if discernible with yellow pigment, extending to ~ chaetiger 8 in best preserved specimens. Peristomium moderately developed and not fused to prostomium. Branchiae from chaetiger 2, cirriform, separate from dorsal lamellae (mostly lost in present material). Interparapodial pouches present from between chaetigers 3 and 4. Parapodial lamellae broad, particularly foliaceous in notopodia of the anterior mid-body. Capillaries arranged in two or three rows in anterior notopodia, in neuropodia in two rows. Sabre chaetae first present from chaetigers 10–13, appearing first as up to five capillaries in inferiormost position, in hook-bearing chaetigers usually as one or two stout granulated chaetae. Hooded hooks first observed in neuropodia of chaetigers 17–19, numerous (numbering 15), with four apical teeth above main fang; in notopodia hooks absent. Dorsal crests not observed. Pygidium unknown.

###### Remarks.

The morphology of specimens examined is in accordance with diagnostic characters for *L. blakei*. Important characters are the start of interparapodial pouches between chaetigers 3 and 4, prostomium not fused to the peristomium, the start of sabre chaetae not before chaetigers 10, and of neuropodial multidentate hooded hooks from about chaetigers 20. However, most of the specimens from IN2017_V03 were in very poor condition and not all diagnostic characters could be observed in each specimen. *Laonice blakei* is known from deep waters of the Atlantic Ocean and Nordic Seas but has not been reported before from Australian waters or the Pacific Ocean in general. Considering this we refer to our specimens as L. cf. blakei.

###### Records.

11 specimens. Suppl. material 1: ops. 9, 31, 33, 42, 55, 76, 79 (AM).

##### Spiophanes anoculata

Taxon classificationAnimaliaPhyllodocidaSpionidae

Hartman, 1960

Fig. 31G

###### Diagnosis.

Prostomium broad anteriorly, bell-shaped, with short but distinct anterolateral horns, posteriorly short straight extension with papilliform occipital antenna. Eyes absent in material from the present study but four minute, deeply embedded, red eyes sometimes present in material from the east Pacific. Dorsal ciliated organs as continuous ciliated grooves to the end of chaetiger 2, thereafter as segmental dorsal ciliated grooves interrupted by segmental furrows, after chaetiger 18 or later changing again to continuous double lines (missing in IN2017_V03 specimens); ciliated grooves bordered by pigment of dark orange or ochre colour. Chaetal spreader of ‘0+1’ type, present on chaetigers 5–8, opening of glandular organs on chaetigers 9–14 as simple vertical slits. Parapodial lamellae not well preserved in most specimens. Chaetiger 1 bearing stout, crook-like chaeta in neuropodium. Notochaetae mostly simple capillaries and capillaries with narrow sheath arranged in a tuft, neurochaetae capillaries with sheaths arranged in two or three rows, stout capillaries in anterior and middle body region; from chaetigers 15 neuropodia with quadridentate hooded hooks; stout granulated sabre chaetae starting on chaetigers 4, very long in anterior chaetigers. Ventrolateral intersegmental pouches absent.

###### Remarks.

Specimens are in good agreement with former descriptions, with the most conspicuous character being the metameric dorsal ciliated organs. See Meißner (2005) and Blake (1996b) for details of parapodial lamellae, chaetal arrangement, and details of chaetae. However, the description by Blake (1996b) deviates from Australian material and also from former descriptions of specimens from the NE Pacific Ocean (Meißner 2005) in that Blake describes continuous ciliated grooves reaching the end of chaetigers 3 rather than 2, and sabre chaetae to start on chaetigers 15 instead of chaetiger 4.

###### Records.

5 specimens. Suppl. material 1: ops. 27, 40, 54, 79 (AM).

##### Spiophanes cf. viriosus

Taxon classificationAnimaliaPhyllodocidaSpionidae

Meißner & Hutchings, 2003

###### Remarks.

The specimen is only a middle fragment in poor condition, without prostomium and posterior end. Due to this we abstain from a more detailed description. However, based on pigment observable in parapodia of the middle body region, chaetal spreaders which are not of the ‘0+1’ type but possibly of the ‘2+3 type’ present in chaetigers 5–7, and glandular openings in chaetiger 8 being absent the fragment might be tentatively referred to *Spiophanes viriosus* Meißner & Hutchings, 2003.

*Spiophanes viriosus* was originally described from coastal waters in Queensland, Australia.

###### Records.

1 specimen, middle fragment. Suppl. material 1: op. 11 (AM).

##### Spionidae gen. spp.

Taxon classificationAnimaliaPhyllodocidaSpionidae

###### Remarks.

Specimens from Brenke sledge samples were incomplete and could not be identified.

###### Records.

24 specimens. Suppl. material 1: ops. 5, 9, 23, 27, 31, 33, 40, 42, 45, 54, 66, 79, 96, 98, 110, 134 (NHMUK).

#### Family Sternaspidae Carus, 1863

M. Georgieva

Commonly known as mud owls, Sternaspidae are distinctive round-bodied or peanut-shaped worms are easily recognized by their characteristic and often colourful ventro-caudal shield (Drennan et al. 2019). Currently, Sternaspidae is comprised of 42 species in three genera, with the largest genus, *Sternaspis* Otto, 1820, containing 32 species (Read and Fauchald 2020). They have a global distribution and live buried in soft sediment at depths varying from the intertidal zone to 4400 m. Three named species from two genera *Caulleryaspis* Sendall & Salazar-Vallejo, 2013 and *Sternaspis* have been reported from Australian waters (http://www.ala.org.au). In this study, we report two species from the genus *Sternaspis*.

##### Sternaspis

Taxon classificationAnimaliaPhyllodocidaSternaspidae

sp.

Fig. 32A, B

###### Diagnosis.

Body ~ 5 mm long and < 2 mm wide. Segments between introvert and rest of body highly cinched, with body 0.7 mm wide at narrowest point. Differing colouration between introvert and abdomen apparent. Ventro-caudal shield a bright orange colour, ribbed and concentrically ringed.

###### Remarks.

These specimens may also represent Sternaspis cf. annenkovae, but further investigation is required to confirm this.

###### Records.

3 specimens. Suppl. material 1: ops. 35, 40 (AM).

**Figure 32.**
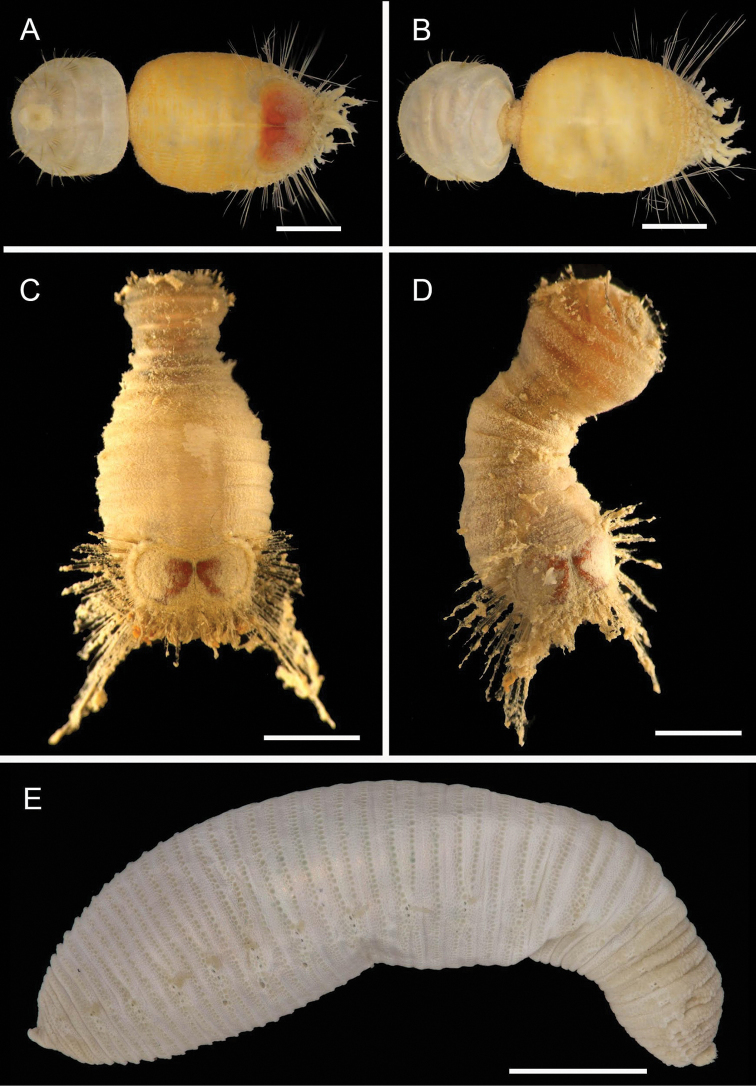
Sternaspidae. Travisiidae**A***Sternaspis* sp., ventral view (op. 40) **B***Sternaspis* sp., dorsal view (op. 40) **C**Sternaspis cf. annenkovae, ventral view (op. 40) **D**Sternaspis cf. annenkovae, ventral view (op. 40) **E***Travisia* sp. 1 (AM W.52547). Scale bars: 1 mm (**A, B**); 3 mm (**C, D**); 5 mm (**E**).

##### Sternaspis cf. annenkovae

Taxon classificationAnimaliaPhyllodocidaSternaspidae

Salazar-Vallejo & Buzhinskaja, 2013

Fig. 32C, D

###### Diagnosis.

Body ~ 11.5 mm long and 5 mm wide. Segments between introvert and rest of body appearing cinched. Body covered in fine papillae largest and densest on segments 7 and 8. Ventro-caudal shield ribbed and concentrically ringed.

###### Remarks.

For further details see Drennan et al. (2019).

###### Records.

2 specimens. Suppl. material 1 op. 40 (NHMUK).

#### Family Syllidae Grube, 1850

A. Murray

Syllidae is a family of small to medium-sized (2–3 mm to 14 cm) annelids distinguished by the presence of a muscular region of the anterior digestive tract known as the proventricle, which may be seen through the dorsal body wall. Syllids are a diverse and abundant group, with currently, 74 genera and ~ 700 species (San Martin and Aguado 2019), that inhabit most marine environments, but they are more scarce in the deep sea, with ~ 90 species recorded from this environment, though greater numbers are expected to be discovered with the increasing exploration of deep and abyssal depths (Desbruyères and Segonzac 1997; Barroso et al. 2017; San Martin and Aguado 2019). Syllids in the deep sea tend to be smaller than those in shallow waters and may be recognised by the distinctive and often conspicuous proventricle of the anterior gut. They are often collected from deep-sea samples as fragments only. In Australia, syllids have been well studied with 54 genera and ~ 260 named species reported (http://www.ala.org.au). This study reports five species from four genera, one species possibly new.

##### Anguillosyllis

Taxon classificationAnimaliaPhyllodocidaSyllidae

sp.

Fig. 26D

###### Diagnosis.

Specimens small, < 3.5 mm in length, 11 chaetigers, prostomium short, wider than long, with pair of oval pigmented nuchal organs posteriorly, eyes absent. Palps narrow, elongate, longer than prostomial length, fused for almost full length, with tip distally notched. Lateral antennae short, cirriform, wrinkled (not ovate), median antenna missing. Pharyngeal tooth absent, nine or ten terminal papillae around pharynx rim. Proventricle extending through segments 3–4, with an indistinct number of muscle bands (12–15?). Single pair of papilla-like tentacular cirri on peristomium. Dorsal cirri long, filiform, wrinkled, coiling; few remaining, absent or missing from chaetiger 2. Ventral cirri digitiform, short, inserted somewhat distally (more than midway) on parapodia. Parapodia elongate with distally rounded posterior lobes, retractile elongate postchaetal lobes not obvious (all retracted?), but presumably represented by a small dorsal papilla-like protuberance. Parapodial glands not evident. Chaetae all compound, long-bladed spinigerous chaetae and shorter-bladed falcigers with finely spinulose blades and unidentate tips. Emergent aciculae and simple chaetae not observed. Posterior end truncated, damaged on most specimens, with at least one pair of long lateral pygidial cirri present, but ventromedial pygidial cirri missing on all specimens.

###### Remarks.

Recently, a revision of the *Anguillosyllis* species from deep-water locations was published by Maciolek (2020). This author increased the number of nominal species described from four to 20 (Read and Fauchald 2020) and determined that the number of chaetigers was a specific character. Accordingly, the only described species which possess a combination of 11 chaetigers, palps fused for most of their length as well as elongate, bladed, spinigerous, compound chaetae are the type species for the genus, *A. capensis* Day, 1963, *A. capensis* sensu Böggemann and Purschke (2005), and *A. capensis* sensu Böggemann (2009). These Australian specimens most resemble a description by Böggemann (2009) of specimens from the abyssal SE Atlantic Ocean (recorded from 3950–5655 m depth off western Africa), which Maciolek (2020, in Remarks for the genus diagnosis, p.15) considers to be two different species as well as different to *A. capensis* Day, 1963. Böggemann’s (2009) and Böggemann and Purschke’s (2005) west African specimens possess dorsal cirri on chaetiger 2 as well as simple chaetae in superior and inferior positions in some specimens, which our Australian specimens appear to lack.

For these Australian specimens, because we cannot determine the relative extent of the posterior parapodial lobes which are retracted completely (presumably, or are absent entirely), and because all dorsal cirri are missing from chaetiger 2 (or may be completely absent), it is not possible to determine whether these specimens are the same as one of the two species (described as one) of Böggemann (2009). It does not conform to any other nominal species’ descriptions and is likely to be new.

###### Records.

16 specimens. Suppl. material 1: ops 9, 16, 31, 33, 54, 79 (NHMUK). 11 specimens. Suppl. material 1: ops 16, 31, 33, 42, 54, 79 (AM).

##### Exogone cf. heterosetosa

Taxon classificationAnimaliaPhyllodocidaSyllidae

McIntosh, 1885

Fig. 26E

###### Diagnosis.

Specimen incomplete, 3 mm long, 0.25 mm wide for 29 segments. Palps fused for full length, curled ventrally. Two pairs eyes. Three antennae, median antenna longer than combined length of prostomium and palps, lateral antennae shorter than palps. Proventricle through 3–4 chaetigers. Single pair of papillae-like tentacular cirri; dorsal cirri similar to tentacular cirri, slightly longer, absent on chaetiger 2. Parapodia uniramous with compound chaetae and a single dorsal simple chaeta per parapodium from chaetiger 1; compound chaetae mostly short-bladed heterogomph bidentate falcigers with secondary tooth larger than distal one, and short marginal spines, plus a single spiniger-like compound chaeta per parapodium, shafts distally spinose, blades elongate, enlarged basally (triangular) and tapering to fine indistinctly bidentate tips; single aciculum per parapodium, distally rounded.

###### Remarks.

This specimen most resembles *E. heterosetosa* McIntosh, 1885, according to the original description and the subsequent redescription by San Martin (2005) and comments by Barroso et al. (2017), but differs from it by the more elongate, basally-expanded blades of the spiniger-like compound chaetae. The known distribution of *E. heterosetosa* is subantarctic seas, and it has been recorded from Australian coasts from shallow waters < 600 m depth according to San Martin (2005).

###### Records.

1 specimen. Suppl. material 1: op. 40 (AM).

##### Syllis

Taxon classificationAnimaliaPhyllodocidaSyllidae

sp. 1

###### Diagnosis.

Palps free to base. Two pairs of eyes, prostomium broad. Pharynx everted, single anterior tooth present, ten soft papillae around rim. Pharynx extending to chaetiger 8, proventriculus extends through another 9–10 segments. Body large anteriorly, tapering posteriorly. Parapodia all short, ventral cirri short, dorsal cirri all articulate, long, thin, some curled, alternating lengths after ~ chaetiger 10, some articles longer in posterior dorsal cirri; dorsal cirri present on chaetiger 2. Chaetae in anterior segments with at least one large aciculum per chaetiger, projecting tip curved in anterior chaetigers, tip straight in mid-body and curved in posterior chaetigers, plus a few other thinner, straight aciculae (tapering tips) in mid- and posterior body chaetigers; chaetae all compound falcigers, at least ten per chaetiger: very short, finely serrated blades with bidentate tips; anteriorly, chaetal blades fine with small subdistal tooth, almost unidentate; posteriorly, teeth almost subequal; some shafts of falcigers with acute asymmetric extension; pseudocompound falcigers absent; simple dorsal chaetae present in posterior chaetigers, slender, slightly curving and distally minutely bifid.

###### Remarks.

Specimen were in sponge collected with echinoderms. It is not *S. sclerolaema* Ehlers, 1901, which has pseudocompound chaetae.

###### Records.

1 specimen. Suppl. material 1: op. 69 (AM).

##### Syllis

Taxon classificationAnimaliaPhyllodocidaSyllidae

sp. 2

###### Diagnosis.

Body pigment absent. Palps free to base. Pharyngeal tooth present, ten soft papillae around rim of pharynx; two pairs of eyes. Dorsal cirri alternating long and short with > 20 articles. Parapodia with compound falcigers only, with medium to short serrated bidentate blades; aciculae pointed. Pseudocompound and spiniger-like chaetae absent. Dorsal simple chaetae only present posteriorly, with minute bifid tips.

###### Remarks.

This species is not the same as those collected in the GAB samples e.g., NMV F242523 – RE2017_C01, which has very short dorsal cirri, and NMV F242524 – RE2017_C01, which lacks eyes and has larger dorsal cirri which are straight, not curled.

###### Records.

1 specimen. Suppl. material 1: op. 13 (AM).

##### Syllinae

Taxon classificationAnimaliaPhyllodocidaSyllidae

indet.

###### Diagnosis.

Specimen incomplete, damaged, ~ 10 mm long, 0.8 mm wide for 69 chaetigers, all antennae and chaetal blades missing. Palps free to base, two pairs of eyes, plus a minute pair of anterior eyespots. Pharynx everted with a single dorsal tooth just below papillated rim. A few anterior dorsal cirri remaining, with short thin articles. Parapodia uniramous, with heterogomph compound chaetae, blades unknown.

###### Remarks.

This specimen is too damaged to be identified. It does not appear to be the same as *Syllis* sp. 2, which has longer dorsal cirri and which was collected at the same operation.

###### Records.

1 specimen. Suppl. material 1: op. 13 (AM).

#### Family Terebellidae Johnston, 1846 emended Nogueira, Fitzhugh & Hutchings, 2013

P. Hutchings

Terebellids have multiple long grooved palps which extend out from the worm, thus giving them the name spaghetti worms. The body has a distinct thorax and abdomen defined by the distribution of noto- and neuropodia, and usually two or three pairs of branched or tufted branchiae. The family currently contains 73 genera and > 675 species (Hutchings et al. 2021). The family is found from the intertidal to deep waters. There are currently 26 genera and 80 named species reported from Australian waters (http://www.ala.org.au). All Australian species have been described from shallow water, except *Pista torquata* Hutchings, 2007, which has a type locality of the Tasman Sea 610 m depth and was reported from the GAB at 1340–1320 m (Hutchings 2007). The present study reports three genera and four species, one species is likely new to science.

##### Amphitrite

Taxon classificationAnimaliaPhyllodocidaTerebellidae

sp.

###### Diagnosis.

One pair (?) of poorly branched branchiae and lateral lobes.

###### Records.

5 specimens. Suppl. material 1: op. 80 (AM).

##### Loimia

Taxon classificationAnimaliaPhyllodocidaTerebellidae

sp.

###### Diagnosis.

Lateral lobes present on segment 1, and also present on segment 3 and sometimes on segment 4, three pairs of arborescent branchiae on segments 2–17, pairs of notopodia with smooth tipped winged capillaries from segment 4. Neuropodia from segment 5, short handled uncini with high, pectinate crests, partially intercalated to completely separated double rows back to back from segment 11 until termination of notopodia. Abdominal neuropodia with uncini arranged in single rows.

###### Records.

1 damaged specimen. Suppl. material 1: op. 6 (AM).

##### Pista

Taxon classificationAnimaliaPhyllodocidaTerebellidae

sp.

###### Diagnosis.

Glandular lobes on segments 2–4 of variable sizes and positions, and segment 1 reduced dorsally with pair of glandular lobes. Branchiae arborescent, pectinate or plumose from segment 2, typically two pairs on segments 2 and 3, rarely a single pair or three pairs. Seventeen pairs of smooth tipped winged notochaetae from segment 4. Neuropodia from segment 5, as long handled avicular uncini at least on anterior segments arranged in single rows, then arranged in double rows until end of thorax, reverting to single rows to pygidium.

###### Remarks.

Specimens of *Pista* were in poor condition. They probably represent at least two species, but branchiae and lateral lobes, critical characters to distinguish species, are damaged and in many cases incomplete, including posterior thorax. Molecular data may help in distinguishing between species.

###### Records.

23 specimens. Suppl. material 1: ops. 4, 22, 30, 35, 56, 67 (AM).

##### Terebellidae

Taxon classificationAnimaliaPhyllodocidaTerebellidae

gen. nov. sp. nov. 1

###### Diagnosis.

Fourteen pairs of notopodia, neuropodia begining before end of notopodia, abranchiate genus.

###### Remarks.

Potentially new genus and species.

###### Records.

1 specimen. Suppl. material 1: op. 22 (AM).

##### Terebellidae gen. spp.

Taxon classificationAnimaliaPhyllodocidaTerebellidae

Fig. 26F

###### Remarks.

Specimens were too damaged for further identification.

###### Records.

16 specimens Suppl. material 1: ops. 4, 6, 22, 53, 90, 100, 104 (AM).

#### Family Travisiidae Hartmann-Schröder, 1971

L. Avery, R. S. Wilson

The family Travisiidae is characterised by a short, thick, grub-like body tapered at both ends. Travisiidae contains a single genus, *Travisia*, with three accepted species. *Travisia* specimens are not usually numerous in benthic samples, but the genus is well represented in abyssal and bathyal environments. Twenty species are recorded from depths of 250 m or greater (Blake and Maciolek 2019b) and eight species are only found in bathyal depths (2000 m or greater). Four named species of *Travisia* have been reported from Australian waters (http://www.ala.org.au). Here we report one species.

##### Travisia

Taxon classificationAnimaliaPhyllodocidaTravisiidae

sp. 1

Fig. 32E

###### Diagnosis.

Body of 22–25 chaetigers. Prostomium conical, longer than maximum width. Chaetae present from segment 2, one achaetous posterior segment (smallest specimens with chaetae only visible on anterior segments 2–5). Mouth located between chaetigers 1 and 2. Segment 1 uniannulate; anterior and posterior segments, starting at segment 2 triannulate (no obvious differentiation between anterior and posterior regions). Branchiae present, first on chaetiger 3–6, continue for 8–11 chaetigers. Branchiae much shorter than body diameter. Branchiae absent on specimens less than ~ 9.5 mm long, but present on an increasing number of segments on the largest specimens collected. Epidermal papillae are low and sparse at the anterior margin of each segment, becoming larger towards the posterior margin of each segment. Notopodial and neuropodial lobes commencing on chaetiger 3 (in small specimens either absent or difficult to distinguish from adjacent epidermal papillae). Parapodial lobes continuous with an encircling row of papillae, remaining epidermis of each segment low tessellation. Interramal pores first present chaetiger 1, last on chaetiger 20. Pre-pygidial 8–12 segments forming deep lateral grooves within which parapodia and chaetae located (only on the largest specimens). Pygidial tube with six or seven blunt lobes equal in length to the last two chaetigers. The last six dorsal posterior chaetigers crenulated.

###### Remarks.

Initially the smallest specimens were treated as a distinct OTU (in these the chaetae are sparse, papillae are less distinct and branchiae and parapodial lappets are not observable) but it seems more likely that this represents size-related variation. Other than having branchiae, *Travisia* sp. 1 is strikingly similar to abranchiate species *Travisia glandulosa* McIntosh, 1879 (e.g., see Wiklund et al. 2019: fig. 31D) and *Travisia gravieri* McIntosh, 1908 (see Kirkegaard 1996). As noted above, branchiae are reduced and difficult to observe, or apparently absent in several small specimens of *Travisia* sp. 1 but branchiae have never been reported in *T. glandulosa* or *T. gravieri*. *T. glandulosa* appears to have a disjunct distribution at abyssal depths, with isolated groups of records at ~ 60°N and 60°S in the Atlantic, plus several isolated records in the Kermadec and Sunda Trenches. *Travisia gravieri* is also widely reported in the North Atlantic at abyssal and bathyal depths in addition to a single record off Angola in the South Atlantic; however, the Angola specimen was only 4×1.5 mm (Kirkegaard 1996) and we were not able to observe branchiae in specimens of *Travisia* sp. 1 from this study of similar size. It seems that *T. glandulosa*, *T. gravieri*, and *Travisia* sp. 1 may belong to a single species or species complex but re-evaluation of these taxa is beyond the scope of this study.

Among species with branchiae, only four other species along with *Travisia* sp. 1 have branchiae commencing at chaetiger 3 (*Travisia carnea* Verrill, 1873; *Travisia filamentosa* León-González, 1998; *Travisia hobsonae* Santos, 1977 and *Travisia profundi* Chamberlin, 1919) but none of these have all chaetigers triannulate. *T. profundi* is similar in having 12 chaetigers with branchiae (*Travisia* sp. 1 has 8–11 chaetigers with branchiae), but in *T. profundi* there is a transition to biannulate and uniannulate posterior chaetigers, and ten or 11 anal lobes compared with six or seven in *Travisia* sp. 1. This species differs from the two *Travisia* OTUs reported from 141–375 m in the GAB (MacIntosh et al. 2018: additional file 2).

###### Records.

6 specimens. Suppl. material 1: ops. 4, 16, 31, 54, 56 (AM).

##### Travisia

Taxon classificationAnimaliaPhyllodocidaTravisiidae

sp.

###### Remarks.

Material is represented by immature unidentifiable specimens.

###### Records.

1 specimen. Suppl. material 1: op. 79 (AM). 1 specimen. Suppl. material 1: op. 16 (NHMUK).

#### Subclass Echiura Sedgwick, 1898

**Order Echiuroidea**

**Suborder Bonelliida**

##### Family Bonelliidae Lacaze-Duthiers, 1858

P.-W. Hsueh

The family is characterised by the presence of sexual dimorphism which is not seen in all other families of Echiura. The female is small to medium in size with sac-like trunk and with truncate or bifid proboscis. The male is usually small, planarian-like or nematiform, often parasitic in or on the female (Stephen and Edmonds 1972). Thirty genera and 78 species are currently known (Read and Fauchald 2020). Of these species, seven are reported from Australia: *Archibonellia michaelseni* Fischer, 1919; *Metabonellia haswelli* (Johnston & Tiegs, 1920); *Protobonellia papillosum* Murina, 1978; *Pseudobonellia biuterina* Johnston & Tiegs, 1919; *Sluiterina album* Murina, 1978; *Vitjazema ultraabyssalis* Zenkevitch, 1958, and *Zenkevitchiola brevirostris* Murina, 1978 (Edmonds 1987). The present study reports *Alomasoma* Zenkevitch, 1958, and *Maxmuelleria* Bock, 1942 for the first time from Australia.

###### Alomasoma

Taxon classificationAnimaliaEchiuroideaBonelliidae

sp. nov. 1

Fig. 33A–C

####### Diagnosis.

**Figure 33.**
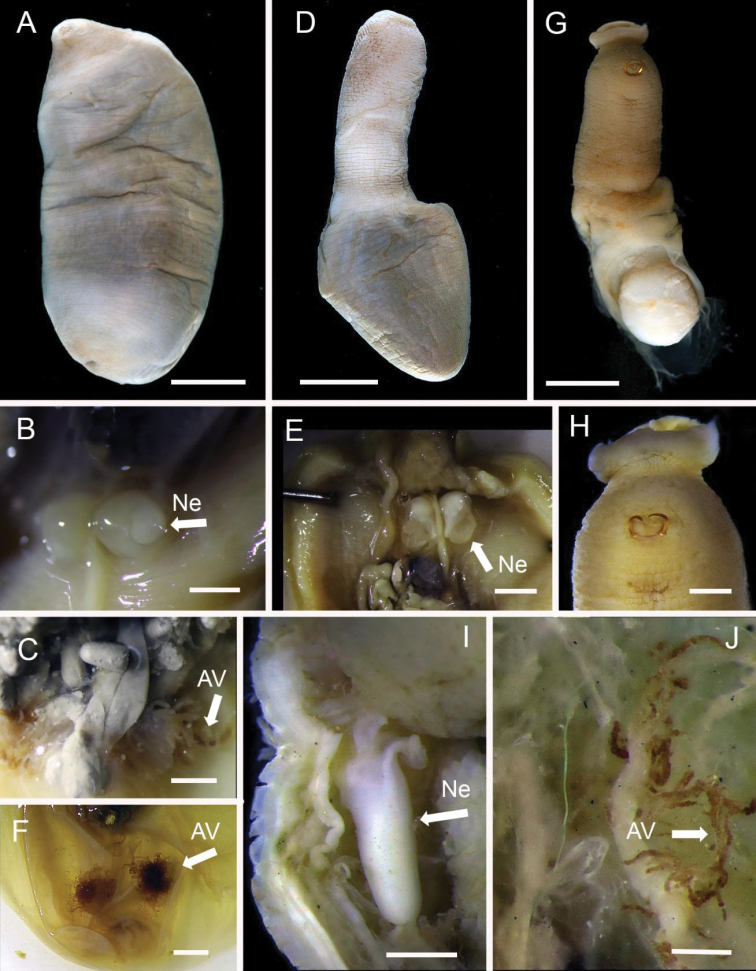
Echiura*Alomasoma* sp. nov. 1 (AM W.49662) **A** whole animal **B** nephridia **C** anal vesicles. *Alomasoma* sp. nov. 2 (AM W.49662) **D** whole animal **E** nephridia **F** anal vesicles. *Maxmuelleria* sp. nov. (AM W.49668) **G** whole animal **H** close-up of anterior part **I** right nephridium **J** anal vesicles. Abbreviations: AV, anal vesicles; Ne, nephridium. Scale bars: 10 mm (**A, D, G**); 2 mm (**B, C, E, F, H, I**); 1 mm (**J**).

Specimen 56 mm in length, body cylindrical, with trace of proboscis, body wall thin (Fig. 33A); ventral chaetae absent; two nephridia with separate pores, nephrostome basally, globular, without stalk, not bifid (Fig. 33B); anal vesicles broom-like (Fig. 33C).

####### Records.

1 specimen. Suppl. material 1: op. 99 (AM).

###### Alomasoma

Taxon classificationAnimaliaEchiuroideaBonelliidae

sp. nov. 2

Fig. 33D–F

####### Diagnosis.

Specimen 47 mm in length, body pear-shaped, with trace of proboscis, body wall thin (Fig. 33D); ventral chaetae absent; two nephridia with separate pores, nephrostome basally with a long stalk, not bifid (Fig. 33E); anal vesicles broom-like (Fig. 33F).

####### Records.

1 specimen. Suppl. material 1: op. 99 (AM).

###### Maxmuelleria sp. nov.

Taxon classificationAnimaliaEchiuroideaBonelliidae

Fig. 33G–J

####### Diagnosis.

Specimens medium in size, ranging from 31 to 40 mm in length. Specimens with either none, one or two ventral chaetae remaining. Present description based on one specimen (AM W.49668; 35 mm in length). Proboscis truncate, no special cup (Fig. 33G, H); two ventral chaetae (Fig. 33H); no anal rosette; two nephridia with separate pores, nephrostome basally, not bifid (Fig. 33I); anal vesicles a long stalk with alternated branches, each branch arborescent (Fig. 33J).

####### Records.

3 specimens. Suppl. material 1: op. 65 (AM).

###### Bonelliidae gen. spp.

Taxon classificationAnimaliaEchiuroideaBonelliidae

Fig. 34A–G

####### Records.

8 specimens. Suppl. material 1: op. 15, 35, 43, 65, 97, 104 (AM).

**Figure 34.**
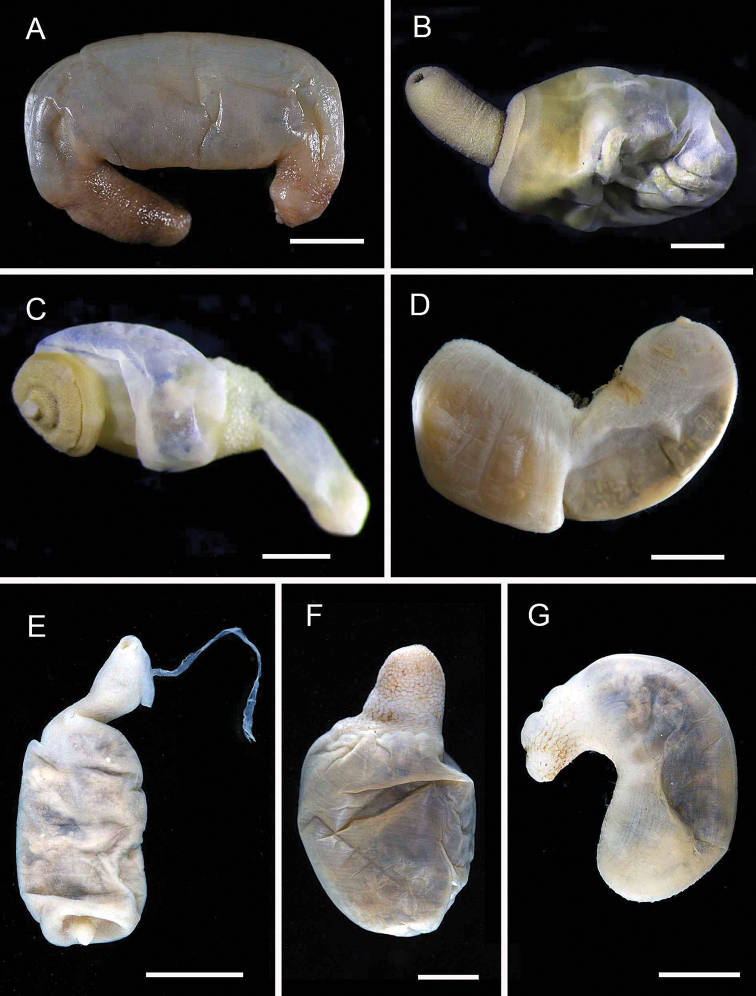
Echiura whole animals of Bonelliidae spp. **A**AM W.49516 **B**AM W.49541 **C**AM W.49541 **D**AM W.49663 **E**AM W.49664 **F**AM W.49665 **G**AM W.49666. Scale bars: 10 mm (**A, D, E**); 2 mm (**B**); 1 mm (**C**); 5 mm (**F, G**).

#### Class Sipuncula

A. Schulze

Formerly considered a distinct phylum, Sipuncula are now regarded as a branch within the annelid radiation (Weigert et al. 2014; Weigert et al. 2016). Sipunculan classification was recently revised based on phylogenetic and phylogenomic studies (Kawauchi et al. 2012; Lemer et al. 2015). Approximately 150 sipunculan species currently recognized (Cutler 1994; Schulze et al. 2019) are organized into six families. The actual number of species is likely much higher based on recent molecular studies indicating that cryptic species are commonplace (Schulze et al. 2012).

In contrast to the ‘typical’ annelid body plan, the sipunculan body is unsegmented. It consists of a trunk region and a retractable introvert, generally with a crown of tentacles at its anterior end. Recurved, proteinaceous hooks are often present along the introvert. The anus is usually located dorsally at the anterior end of the trunk. Nephridiopores (usually two) open at a similar level as the anus on the ventral side. Internally, one two four introvert retractor muscles present.

Sipunculans are generally cryptic in their lifestyle, but can reach high densities in some habitats. They range from the intertidal zone to depths of > 7000 m (Saiz Salinas et al. 2018). Cutler (1977) who examined the material from the ‘Galathea’ expedition, reported 43 species collected below 400 m depth, some of them from southeastern Australia. Saiz Salinas et al. (2018) reviewed literature on deep-sea sipunculans from below 2000 m and listed 51 species. Few of these represented records from southeastern Australia within that depth range. MacIntosh et al. (2018: additional file 2) listed > 1000 specimens from 25 stations (depth range 388–3884 m) in the GAB; together with the present material these collections, when formally described, will add significantly to knowledge of the diversity of abyssal sipunculans.

##### Sipuncula

Taxon classificationAnimaliaSipunculiformes

fam. gen. spp.

Fig. 35D

###### Remarks.

Many specimens not identified beyond phylum level.

###### Records.

101 specimens. Suppl. material 1: ops. 4, 6, 22, 32, 33, 43, 53, 54, 69, 88, 90, 100, 104 (AM). 2 specimens. Suppl. material 1: ops. 11, 100 (NHMUK).

**Figure 35.**
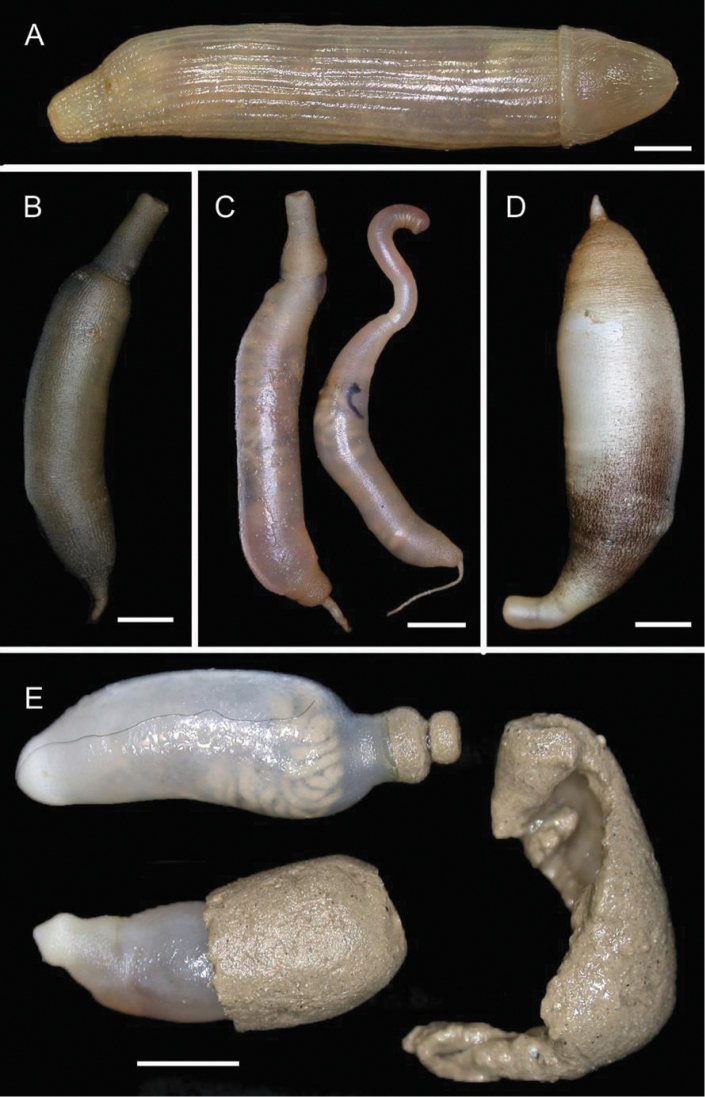
Sipuncula**A***Sipunculus* sp. (AM W.49645), lateral view **B***Golfingia muricaudata* (AM W.49656), lateral view **C***Golfingia muricaudata* (AM W.49657) **D**Sipuncula gen. sp. (op. 35), lateral view **E***Phascolion lutense* (AM W.49601), top: specimen removed from sediment tube; bottom: specimen partially removed from tube; right: sediment tube. Scale bars: 5 mm.

##### Family Sipunculidae

Relatively large sipunculans (usually > 5 cm), with an introvert shorter than the trunk; no introvert hooks. Tentacles arranged in a circle surrounding the mouth. Body wall with externally visible bands of longitudinal and circular musculature crossing each other, giving the impression of rectangular ‘mini-pillows’. One genus and four species are known from the deep sea (> 2000 m) (Saiz Salinas et al. 2018). In Australian waters 11 species from two genera (*Siphonosoma* and *Sipunculus*) have been reported (http://www.ala.org.au). We report at least one species from *Sipunculus*.

###### Sipunculus spp.

Taxon classificationAnimaliaSipunculiformesSipunculidae

Fig. 35A

####### Diagnosis.

Four stem-like tentacles, with the ventral pair smaller than the dorsal pair. Nephridiopores located slightly anterior to the anus.

####### Records.

25 specimens. Suppl. material 1: ops. 4, 80, 104 (AM).

##### Family Golfingiidae Stephen & Edmonds, 1972

Small to large-sized worms (max 200 mm); introvert length similar to trunk length or shorter. Tentacles encircling mouth. Hooks, if present, simple, scattered, not sharply curved and often deciduous. Trunk wall externally smooth or covered with small papillae. The family is well represented in the deep sea (> 2000 m) with 36 species and six genera (Saiz Salinas et al. 2018). In Australian waters six species from two genera (*Golfingia* and *Nephasoma*) have been reported (http://www.ala.org.au). We report at least seven species from four genera.

###### Golfingiidae

Taxon classificationAnimaliaGolfingiiformesGolfingiidae

gen. sp.

####### Records.

1 specimen. Suppl. material 1. op. 42 (AM).

###### Golfingia

Taxon classificationAnimaliaPhyllodocidaGolfingiidae

sp.

####### Diagnosis.

Small to medium worms (usually < 30 mm). Introvert hooks, if present, small (< 40 μm); nephridial pores anterior to anus. Body wall smooth.

####### Remarks.

Further identification is uncertain.

####### Records.

6 specimens. Suppl. material 1: ops. 6, 30, 45, 101 (AM).

###### Golfingia (Golfingia) muricaudata

Taxon classificationAnimaliaGolfingiiformesGolfingiidae

(Southern, 1913)

Fig. 35B, C

####### Diagnosis.

Presence of a distinctive caudal appendage (< ~ 30% of the trunk length); introvert shorter than trunk.

####### Records.

34 specimens. Suppl. material 1: ops. 11, 22, 35, 43, 44, 65, 70, 86, 99 (AM).

###### Phascolion spp.

Taxon classificationAnimaliaGolfingiiformesPhascoliidae

####### Diagnosis.

Small to medium-sized worms (< ~ 50 mm), commonly inhabiting abandoned gastropod or scaphopod shells, polychaete tubes or foraminiferan tests. Trunk usually with unique holdfast papillae. Single nephridium, usually located posterior to the anus.

####### Remarks.

Specimens are not identified to species.

####### Records.

25 specimens. Suppl. material 1: ops. 31, 44, 56, 100, 121, 128 (AM).

###### Phascolion (Montuga) lutense

Taxon classificationAnimaliaGolfingiiformesPhascoliidae

Selenka, 1885

Fig. 35E

####### Diagnosis.

Trunk smooth, except for densely packed papillae at the anterior end. No holdfast papillae. Trunk with characteristic grey anterior ‘cap’. Inhabiting soft clay tubes instead of hard shells.

####### Remarks.

This is the first report of this species from Australian waters.

####### Records.

4 specimens. Suppl. material 1: ops. 6, 67 (AM).

###### Phascolion (Isomya) cf.hedraeum

Taxon classificationAnimaliaPhyllodocidaPhascoliidae

Selenka & De Man, 1883

####### Diagnosis.

Inhabiting sediment tube; round holdfast papillae with hardened borders anteriorly.

####### Records.

1 specimen. Suppl. material 1: op. 89 (AM).

###### Onchnesoma

Taxon classificationAnimaliaGolfingiiformesPhascoliidae

sp.

####### Diagnosis.

Small worms (< 10 mm). Introvert always longer than trunk. Anus on distal end of introvert. No introvert hooks. Single nephridium.

####### Remarks.

Specimens are not identified to species. This is the first report of this genus from Australian waters.

####### Records.

4 specimens. Suppl. material 1: ops. 31, 119 (AM).

###### Thysanocardia cf. catharinae

Taxon classificationAnimaliaGolfingiiformesPhascoliidae

(Grube, 1868)

####### Diagnosis.

Up to 70 mm trunk length. Complex tentacular arrangement in ‘festoons’ (double rows extending along the distal introvert) and around the nuchal organ. Tentacles arranged in 14–16 festoons, each with < 40 tentacles.

####### Remarks.

This is the first record of this genus from Australian waters.

####### Records.

9 specimens. Suppl. material 1: ops. 80, 104 (AM).

##### Family Phascolosomatidae Stephen & Edmonds, 1972

Small to medium-sized worms. Tentacles surrounding the nuchal organ in a semi-circle. Introvert hooks usually present, recurved and organized in circles, typically with internal structures visible under transmitted light. Four introvert retractor muscles. Two genera and eight species are known from the deep sea (> 2000 m) (Saiz Salinas et al. 2018). In Australian waters, 15 species from two genera (*Apionsoma* and *Phascolosoma*) have been reported.

###### ? Apionsoma

Taxon classificationAnimaliaGolfingiiformesPhascolosomatidae

sp.

####### Diagnosis.

Small specimens (usually > 20 mm) with an introvert much longer than trunk. Introvert hooks arranged in rings with accessory basal spinelets. Nephridia bilobed.

####### Remarks.

Identification is uncertain.

####### Records.

15 specimens. Suppl. material 1: po. 6 (AM).

### Analysis of annelid biodiversity

We report > 6000 annelid specimens (box core – 57 specimens, Brenke sledge – 2481, beam trawl – 3470) (Suppl. material 1). Of these, 4714 specimens (78.5%) represented 214 putative species from 50 families. The remaining 1294 specimens were too small or damaged to be assigned to a morphospecies at this time and they were assigned to a family or genus only. Small-bodied representatives of eight families, namely Acrocirridae, Cirratulidae, Euphrosinidae, Fabriciidae, Flabelligeridae, Goniadidae, Lacydoniidae, and Pilargidae, were found exclusively in Brenke sledge and box core material. Only 27 species (624 specimens, 13% of the specimens identifiable to morphospecies) were attributed to known taxa and given valid names, while the remaining 187 (4090 specimens, 87% of the specimens identifiable to morphospecies) were determined in open nomenclature following Sigovini et al. (2016) and were assigned qualifiers sp. nov. (55 species, including six already described from this material), cf. (45), or sp. (87) (Suppl. material 1). The 55 species confidently confirmed by taxonomic experts in the respective families to be new (sp. nov.) will be formally described in the near future. The remaining 132 species (or some of them) assigned cf. and sp. qualifiers may also be new to science; however, the specimens require further study (e.g., molecular data or scanning electron microscopy) to determine the status with confidence. Preliminary data also suggest that complete specimens from the families Eunicidae, Orbiniidae, and Terebellidae could be described within three new genera at a later date. Thus, the material can contain a minimum of 55 species new to science and at least three new genera.

Sixteen of the species collected from the eastern abyss were also reported from stations at the GAB. Of these 16, seven (Nereididae: *Nicon maculata*, Polynoidae: *Bathyeliasona nigra*, *Eunoe abyssorum*, Aphroditidae: *Aphrodita goolmarris*, *Laetmonice benthaliana*, *Laetmonice yarramba*, Goniadidae: *Bathyglycinde profunda*) were named species. The remaining nine undescribed species were confirmed by specialists who had compared both sets of material (restricted to the families Ampharetidae, Aphroditidae, Fabriciidae, Nereididae, Onuphidae, Polynoidae, and Sabellidae).

The most species-rich family was the Polynoidae (> 17 species) followed by Onuphidae (13 species), Serpulidae (12 species), Acrocirridae (10 species) and Maldanidae (10 species). Total species richness of annelids was similar between lower bathyal and abyssal depths (163 and 160 species respectively) and decreased to mid-bathyal depths (70 species). Total species richness was highest at Bass Strait (90 species), Byron Bay (82 species) and Jervis MP (81 species) whilst the Moreton Bay (30 species) and off Fraser Island had the lowest richness (41 species).

## Discussion

This is the first comprehensive report of annelids from the eastern Australian lower bathyal and abyssal region. Our results indicate a higher number of families and species (50 and 214 respectively) than was reported from the recent deep-water survey of the Great Australian Bight (GAB) at 200 to 5000 m depth (42 annelid families, 179 species) (MacIntosh et al. 2018), and from the south western continental margin of Western Australia (12 families, 57 species) where samples were collected from lower shelf and upper bathyal depths at 100, 500, and 1000 m depth contours (Poore et al. 2015). Indeed, more specimens were collected from eastern Australia (Annelida*n* > 6000) than from the GAB (Annelida*n* = 2364) and western Australia (polychaete *n* = 660) combined. Unfortunately, these results are not directly comparable as different sampling gear was used in each study (eastern Australia 54 successful samples using box core, Brenke sledge and beam trawl; GAB 304 samples using eight gear types including beam trawl, benthic sledge, multicorer and ROV pushcore; western Australia 135 grab samples). Our study presents the largest dataset to date on deep-water annelids from Australia. As a result, it adds important records of occurrence and distribution of species and provides a springboard for future taxonomic studies.

Prior to the 2017 survey, only eight annelid species were described from the eastern continental margin of Australia from below 1000 m and only two of them (*Parapolyeunoa flynni* and *Aglaophamus profundus*) from the targeted depths in this study, below 2000 m (Table 1). The high proportion of undescribed (new to science) species, at least 55 and up to 187 species (< 87% of specimens identified to morphospecies), found in this study is unsurprising given that this was the first systematic survey of the region and so few annelids had been previously described from abyssal depths in Australia. The high number of new annelid species is typical for previously un-sampled deep-sea environments. Recent deep-sea investigations have reported varying percentages of annelid (polychaetes) species new to science, for example, 33% in abyssal SE Atlantic (Böggemann 2009), ~ 40% in NE Pacific (Méndez 2006), > 40% from the slope to upper trench in NW Pacific (Alalykina 2018), 55% on the shelf/slope of the Southern Ocean (Neal et al. 2018), ~ 70% in SW Atlantic (Neal et al. 2020), and even up to 90% (higher than in the present study) in the abyssal Pacific (Glover et al. 2002).

While so far only six species have been formally described from this survey material (pectinariids *Petta investigatoris* and *P. williamsonae*, sabellariids *Gesaia csiro* and *Phalacrostemma timoharai* and melinnids *Melinnopsis gardelli* and *Melinnopsis chadwicki*) taxonomic studies are ongoing. The 24 new species currently being described by co-authors of this study include one acoetid of the genus *Panthalis* (Murray), one glycerid (Böggemann), two lumbrinerids (Borisova and Budaeva), four maldanids of the genera *Boguea*, *Chirimia* and *Notoproctus* (Kongsrud), four onuphids of the genus *Nothria* (Paxton and Budaeva), four serpulids of the genera *Bathyvermilia*, *Hyalopomatus* and *Spirodiscus* (Kupriyanova), four sphaerodorids of the genera *Geminofilum* and *Sphaerephesia* (Capa), and four scalibregmatids of the genera *Asclerocheilus*, *Oligobregma*, *Pseudoscalibregma* and *Scalibregmides* (Blake). At least one new species of Siboglinidae (genus *Osedax*) and several species of Dorvilleidae (genus *Ophryotrocha*) were recovered from a whale carcass collected from op. 100 (Georgieva). It is anticipated that the large international effort in species identification by taxonomic specialists in each annelid group will eventually result in the description of all the new annelids from the expedition. Once this taxonomic work is complete, we can test relevant biogeographical hypotheses, in particular we will be able to address the question of species connectivity along the eastern continental margin across to the deep the GAB.

Sixteen morphospecies reported from this study were also recorded from the GAB in southern Australia (MacIntosh et al. 2018) suggesting that at least some species ranges can span across both the eastern and southern margin of Australia. This agrees with the traditional view that deep-sea species have larger geographical ranges than shallow-water species (Ekman 1953), a view that is supported by some recent studies (e.g., McClain and Hardy 2010; Higgs and Attrill 2015). Indeed, geographical range is generally thought to increase with increasing depth (e.g., Etter and Rex 1990; Allen and Sanders 1996). Depth has been long known to have a strong structuring influence on deep-sea species distributions (e.g., Ekman 1953; France and Kocher 1996; Zardus et al. 2006). High species turnover occurs between the shelf break and 1000 m marking a transition between shelf and slope fauna, and between 2000 and 3000 m the transition between slope and abyssal fauna (Carney 2005; Brown and Thatje 2014) and thus, boundaries of biogeographical provinces are typically delineated by depth. Modelling shows that at lower bathyal depths (800–3000 m) the southern Australian fauna is distinct from the eastern fauna, while at abyssal depths (3500–6500 m) species are distributed across the Indian Ocean to the eastern margin of Australia (UNESCO 2009; Watling et al. 2013). Thus, we expect abyssal, but not bathyal fauna, to range from eastern Australia to the GAB. Interestingly, when these biogeographical hypotheses are tested against fine-resolution data from ophiuroids, the bathyal zone fauna (0–2000 m) is continuous from southeastern to southwestern Australia (O’Hara et al. 2011) suggesting the predicted biogeographical provinces around Australia may not hold true for all taxa.

Existing molecular data on the annelid material collected during ‘Sampling the Abyss’ voyage support annelid species connectivity along the eastern Australian margin from southern Queensland to Tasmania. Analysis on DNA sequence data confirmed that specimens of pectinariid *Petta investigatoris* were collected < 491 km apart from Bass Strait to Jervis MP, NSW (Zhang et al. 2019) and the ampharetids *Melinnopsis gardelli* occurred at Freycinet MP and Coral Sea MP (distance 2064 km), while *Melinnopsis chadwicki* was found from the Hunter Marine Park to the Coral Sea Marine Park (996 km) (Gunton et al. 2020). The latter two melinnid species were found to have distinct bathymetric ranges: *M. gardelli* was recorded from 2520–2821 m and *M. chadwicki* from 1006–1257 m depth (Gunton et al. 2020). While annelid species are found within distinct depth ranges along the eastern Australian margin, whether these species ranges extend to the GAB is less clear due to lack of genetic studies.

From 16 morphospecies reported from the present study and the GAB, the two nominal morphospecies with the widest reported distributions in our data were the aphroditid *Laetmonice yarramba* Hutchings & McRae, 1993 and the goniadid *Bathyglycinde profunda* (Hartman and Fauchald 1971). These species were reported from both the northernmost (Coral Sea MP and off Fraser Island) and southernmost (Flinders MP and Freycinet MP) stations.

*Laetmonice yarramba*, originally described from 60–102 m off the coast of NSW, was reported across a distance of more than 1800 km from Freycinet MP (op. 4) to off Fraser Island (op. 115) with a total depth range of 3868 m (Suppl. material 1). This species was also recorded from 21 stations at the GAB from 189–3884 m (MacIntosh et al. 2018). While the geographic range from eastern Australia to the GAB is likely, the apparent broad bathymetric range from shelf to abyssal depths (60–3884 m) requires further investigation as this putative depth range would extend vertically across shelf, slope and abyssal faunal zones. Genetic evidence suggests some deep-water annelids have ‘broad’ (> 2500 m sensu Glazier and Etter 2014) depth ranges, e.g., the maldanid *Nicomache lokii* range 3668 m (Eilertsen et al. 2018), but bathymetric ranges of other deep-sea annelid species are more restricted e.g., 1300 m for the spionid *Laonice weddellia* (see Brasier et al. 2017). According to the census of abyssal polychaetes (> 2000 m), ~ 62–78% of species within a family had bathymetric ranges smaller than 1000 m (Paterson et al. 2009). Thus, the broad (> 3000 m) depth range of *L. yarramba* along with its reported wide geographic range and morphological variability (Hutchings and McRae 1993 and herein) suggests that the nominal species is a species complex and molecular data are needed to test this hypothesis.

*Bathyglycinde profunda* (Hartman & Fauchald, 1971) was originally described from the equatorial region off northeast South America (4825 m). This taxon in the present study was recorded from Flinders MP to Coral Sea MP (ops. 16 to 134), a distance of 1920 km and depth range 2093–4280 m. The same morphospecies has been also reported from northwest Atlantic Ocean (2862–5023 m) by Hartman and Fauchald (1971), from the outer shelf and continental slope off Brazil (325–508 m) by Rizzo and Amaral (2004), from the Clarion-Clipperton Zone in the Pacific by Janssen et al. (2019) and the GAB by MacIntosh et al. (2018). Whether the species in this study is the same as the one described from South America requires further genetic evidence as distribution of such a species would fall into multiple biogeographical provinces as defined by UNESCO (2009) and Watling et al. (2013). Recent molecular studies have provided contrasting results on annelid species range size previously based on morphological data. Genetic evidence exists for abyssal annelids with broad geographic ranges (4150 km, see Schüller and Hutchings 2012) and as well as for bipolar distributions of deep-sea species from chemosynthetic environments (16000 km, Georgieva et al. 2015; Eilertsen et al. 2018). Yet, some deep-sea annelids previously believed to have broad distributions based on morphology alone were found to include multiple species as a result of molecular studies (e.g., Stiller et al. 2013). As with *L. yarramba*, the distribution of *B. profunda* is likely to be more restricted than morphology suggests, and molecular data are needed to test these broad species ranges.

Once more data on the extent of annelid species range sizes from this study become available, we will be able to not only address the degree of genetic connectivity between the GAB and eastern Australian margin, but also to examine underlying biogeographical the patterns across annelids. In particular, we will be able to address the question whether the tropical to temperate transition between 30–40°S reported for deep-sea ophiuroids (O’Hara et al. 2011, 2014) and megafauna collected during the same cruise ‘Sampling the Abyss’ (O’Hara et al. 2020b) is also observed in annelid fauna.

## Conclusions

We report 214 annelid species from the eastern Australian margin, at least 55 of which are new to science. Prior to 2017, only two annelid species were described from the region below 2000 m, consequently this work vastly increases our knowledge of deep-water annelids and provides critical baseline data on an important group of benthic invertebrates from a virtually unknown region of the world’s ocean. This is important as comprehensive taxonomically-consistent deep-water datasets that cover large areas in Australia are rare (O’Hara 2008; Alderslade et al. 2014; Althaus et al. 2017; MacIntosh et al. 2018; Williams et al. 2018; Farrelly and Ahyong 2019; Ekins et al. 2020; O’Hara et al. 2020a, b, c), in particular for smaller benthic invertebrates (Poore et al. 2015), which highlights the uniqueness of this dataset.

The strong feature of this study is that specific annelids groups were sent to taxonomic specialists around the world giving us more confidence in the species identifications. Furthermore, because all material from this study is deposited or will soon be in properly curated museum collections (AM, MV, NHMUK) and a significant proportion has been preserved for molecular studies, these valuable samples are easily accessible and can be used for answering important questions on taxonomy and species ranges along the eastern Australian margin including seven deep-water Marine Parks.

## Appendix 1.

Note added in proofs: Microphthalmidae Hartmann-Schröder, 1971 was accepted as a valid family in the World Register of Marine Species on 27 June 2020. [Read G, Fauchald K [Ed.] (2021) World Polychaeta database. Microphthalmidae Hartmann-Schröder, 1971. Accessed through: World Register of Marine Species at: http://www.marinespecies.org/aphia.php?p=taxdetails&id=322549 on 2021-01-25].

## Supplementary materials

Supplementary material 1 Annelid identifications from IN2017_V03_Darwin Core format Data type occurrences Explanation note species occurrences along with museum registration numbers. https://binary.pensoft.net/file/513304This dataset is made available under the Open Database License (http://opendatacommons.org/licenses/odbl/1.0/). The Open Database License (ODbL) is a license agreement intended to allow users to freely share, modify, and use this Dataset while maintaining this same freedom for others, provided that the original source and author(s) are credited.Laetitia M. Gunton, Elena K. Kupriyanova, Tom Alvestad, Lynda Avery, James A. Blake, Olga Biriukova, Markus Böggemann, Polina Borisova, Nataliya Budaeva, Ingo Burghardt, Maria Capa, Magdalena N. Georgieva, Christopher J. Glasby, Pan-Wen Hsueh, Pat Hutchings, Naoto Jimi, Jon A. Kongsrud, Joachim Langeneck, Karin Meißner, Anna Murray, Mark Nikolic, Hannelore Paxton, Dino Ramos, Anja Schulze, Robert Sobczyk, Charlotte Watson, Helena Wiklund, Robin S. Wilson, Anna Zhadan, Jinghuai Zhang

Supplementary material 2 Spionidae characters Data type morphological Explanation note Comparison of morphological characters of *Prionospio* species from IN2017_V03. https://binary.pensoft.net/file/513305This dataset is made available under the Open Database License (http://opendatacommons.org/licenses/odbl/1.0/). The Open Database License (ODbL) is a license agreement intended to allow users to freely share, modify, and use this Dataset while maintaining this same freedom for others, provided that the original source and author(s) are credited.Karin Meißner
